# Prehabilitation for Patients Undergoing Orthopedic Surgery: A Systematic Review and Meta-analysis

**DOI:** 10.1001/jamanetworkopen.2023.8050

**Published:** 2023-04-13

**Authors:** Anuj Punnoose, Leica S. Claydon-Mueller, Ori Weiss, Jufen Zhang, Alison Rushton, Vikas Khanduja

**Affiliations:** 1 Young Adult Hip Service, Physiotherapy Department, Addenbrooke’s–Cambridge University Hospitals NHS (National Health Service) Trust, Cambridge, United Kingdom; 2 School of Allied Health, Anglia Ruskin University, Chelmsford and Cambridge, United Kingdom; 3 Department of Orthopedics, Meir Medical Centre, Kfar-Saba, Israel; 4 School of Medicine, Faculty of Health, Education, Medicine and Social Care, Anglia Ruskin University, Chelmsford, United Kingdom; 5 School of Physical Therapy, Faculty of Health Sciences, Western University, London, Ontario, Canada; 6 Young Adult Hip Service, Department of Trauma and Orthopedics, Addenbrooke’s–Cambridge University Hospital NHS Foundation Trust, Cambridge, United Kingdom

## Abstract

**Importance:**

Prehabilitation programs for patients undergoing orthopedic surgery have been gaining popularity in recent years. However, the current literature has produced varying results.

**Objective:**

To evaluate whether prehabilitation is associated with improved preoperative and postoperative outcomes compared with usual care for patients undergoing orthopedic surgery.

**Data Sources:**

Bibliographic databases (MEDLINE, CINAHL [Cumulative Index to Nursing and Allied Health Literature], AMED [Allied and Complementary Medicine], Embase, PEDRO [Physiotherapy Evidence Database], and Cochrane Central Register of Controlled Trials) were searched for published trials, and the Institute for Scientific Information Web of Science, System for Information on Grey Literature in Europe, and European clinical trials registry were searched for unpublished trials from January 1, 2000, to June 30, 2022.

**Study Selection:**

Randomized clinical trials (RCTs) comparing prehabilitation with standard care for any orthopedic surgical procedure were included.

**Data Extraction and Synthesis:**

Two independent reviewers screened trials. Data were pooled using a random-effects model. Recommendations were determined using the Grading of Recommendations Assessment, Development and Evaluation system and the study was conducted according to the Preferred Reporting Items for Systematic Reviews and Meta-Analyses (PRISMA) reporting guideline.

**Main Outcomes and Measures:**

Pain, function, muscle strength, and health-related quality of life (HRQOL).

**Results:**

Forty-eight unique trials involving 3570 unique participants (2196 women [61.5%]; mean [SD] age, 64.1 [9.1] years) were analyzed. Preoperatively, moderate-certainty evidence favoring prehabilitation was reported for patients undergoing total knee replacement (TKR) for function (standardized mean difference [SMD], −0.70 [95% CI, −1.08 to −0.32]) and muscle strength and flexion (SMD, 1.00 [95% CI, 0.23-1.77]) and for patients undergoing total hip replacement (THR) for HRQOL on the 36-item Short Form Health Survey (weighted mean difference [WMD], 7.35 [95% CI, 3.15-11.54]) and muscle strength and abduction (SMD, 1.03 [95% CI, 0.03-2.02]). High-certainty evidence was reported for patients undergoing lumbar surgery for back pain (WMD, –8.20 [95% CI, −8.85 to −7.55]) and moderate-certainty evidence for HRQOL (SMD, 0.46 [95% CI, 0.13-0.78]). Postoperatively, moderate-certainty evidence favoring prehabilitation was reported for function at 6 weeks in patients undergoing TKR (SMD, −0.51 [95% CI, −0.85 to −0.17]) and at 6 months in those undergoing lumbar surgery (SMD, −2.35 [95% CI, −3.92 to −0.79]). Other differences in outcomes favoring prehabilitation were of low to very low quality of evidence.

**Conclusions and Relevance:**

In this systematic review and meta-analysis of RCTs, moderate-certainty evidence supported prehabilitation over usual care in improving preoperative function and strength in TKR and HRQOL and muscle strength in THR, high-certainty evidence in reducing back pain, and moderate-certainty evidence in improving HRQOL in lumbar surgery. Postoperatively, moderate-certainty evidence supported prehabilitation for function following TKR at 6 weeks and lumbar surgery at 6 months. Prehabilitation showed promising results for other outcomes, although high risk of bias and heterogeneity affected overall quality of evidence. Additional RCTs with a low risk of bias investigating preoperative and postoperative outcomes for all orthopedic surgical procedures are required.

## Key Points

**Question:**

Is prehabilitation associated with improved outcomes in patients undergoing orthopedic surgery?

**Findings:**

In this systematic review and meta-analysis of 48 unique trials involving 3570 unique patients, the prehabilitation intervention significantly improved function, health-related quality of life, muscle strength, and back pain prior to surgery in patients undergoing orthopedic procedures compared with usual care (without prehabilitation). Postoperatively, prehabilitation improved function in the short to medium term in comparison with usual care.

**Meaning:**

These findings suggest that prehabilitation is associated with improving some outcomes for patients undergoing orthopedic surgery both preoperatively and postoperatively.

## Teaser

This systemic review and meta-analysis assesses the association of prehabilitation with preoperative and postoperative outcomes for patients undergoing orthopedic surgical procedures.

## Introduction

An estimated 310 million surgical procedures are performed worldwide every year.^1^ Musculoskeletal disease is the biggest contributor to global disability.^2^ This alongside an aging population has placed an unprecedented demand on surgical services, including orthopedics, leading to increasing waiting lists and further deconditioning of an aged population.^3^ Furthermore, undergoing a major surgical intervention can increase catabolism and oxygen demand, leading to a decline in one’s physical function.^4^ Waiting for a surgical procedure therefore provides a window of opportunity to optimize and influence the preoperative muscle strength, function, and health-related quality of life (HRQOL) of a patient, which are often considered predictive factors associated with postoperative outcomes in the population undergoing orthopedic surgery.^5,6,7,8^ This is termed *prehabilitation*.^9^

Although prehabilitation has been reported in the literature since the 1940s, the role of prehabilitation in improving postoperative outcomes has only been researched from the year 2000.^10,11^ Systematic reviews investigating the benefits of prehabilitation have reported varying conclusions. Cabilan et al^12^ found prehabilitation of more than 500 minutes in patients undergoing hip or knee replacement is only associated with improvements in reducing acute rehabilitation admissions (odds ratio, 0.51 [95% CI, 0.28-0.93]). Moreover, trials included in the review were published before March 2013 and excluded day cases.

A similar study by Wang et al^13^ included 22 trials investigating the effectiveness of prehabilitation for patients undergoing total hip (THR) and total knee (TKR) replacement. The authors reported slight improvement in postoperative pain (at 4 weeks) and concluded the effects were small and only in the short term and therefore clinically insignificant. A more recent systematic review by Widmer et al^14^ on prehabilitation for patients undergoing THR reported prehabilitation to be superior to usual care in several functional performance measures. However, the authors were unable to perform meta-analysis due to the heterogeneity of the outcome measures, which was a major limitation. Therefore, the purpose of the present study was to conduct a comprehensive, up-to-date systematic review and meta-analysis to determine whether prehabilitation is associated with an improvement in outcomes for patients undergoing any orthopedic surgical procedures, including day cases, compared with usual care (no prehabilitation). The secondary objective was to explore the components and dosage of prehabilitation.

## Methods

### Design

A systematic review was conducted according to the Preferred Reporting Items for Systematic Reviews and Meta-Analyses (PRISMA) reporting guideline. The study protocol was registered with PROSPERO (CRD42019123268) and published.^15^

### Eligibility Criteria

#### Trial Design

We searched for randomized clinical trials (RCTs) comparing prehabilitation, including multimodal interventions (eg, exercises with and without pain management), with usual care for adult participants (aged >18 years) undergoing an orthopedic surgical procedure. Eligible trials were published from January 1, 2000, to June 30, 2022. Prehabilitation interventions included exercises, pain management, other adjunct therapies such as acupuncture or electrical stimulation. Pain, muscle strength, function, HRQOL, and disease- and/or joint-specific outcomes were assessed. Additional outcome measures included anxiety and depression, range of motion, measures of functional performance and health economic measures. Non–English language trials were excluded at the full-text review stage.^16^

### Information Sources

A comprehensive independent search by 2 reviewers (A.P. and O.W.) was performed in MEDLINE (OVID), CINAHL (Cumulative Index to Nursing and Allied Health Literature; EBSCO), AMED (Allied and Complementary Medicine; OVID), Embase, PEDRO (Physiotherapy Evidence Database), and Cochrane Register of Controlled Trials for trials published from 2000 to June 2022. The Institute for Scientific Information Web of Science, System for Information on Grey Literature in Europe, and European clinical trials registry were searched for ongoing and unpublished trials. Key orthopedic journals (*The Bone and Joint Journal*, *International Orthopedics*, and *Journal of Orthopedics and Traumatology*) were hand searched. Reference lists of included trials were screened. Trial authors were contacted where data were unavailable. The full search strategy used is found in eTable 1 in Supplement 1.

### Selection Process

Titles and abstracts (stage 1) and full-text trials (stage 2) were independently screened by 2 reviewers (A.P. and O.W.). Where consensus could not be obtained by discussion, the third and fourth reviewers (A.R. and V.K.) were consulted.

### Data Collection Process and Items

Two reviewers (A.P. and O.W.) extracted data independently using a standardized form. A third reviewer (L.S.C.-M.) independently checked data for consistency and accuracy. Data extracted included sample characteristics, sample size, duration, prehabilitation delivery (home, face-to-face, or virtual), components of the prehabilitation program, and results summary.

### Risk of Bias

Risk of bias for each RCT was independently assessed by 2 reviewers (A.P. and J.S.) using the Cochrane risk of bias tool, version 2.0.^17^ Where consensus could not be obtained by discussion, a third reviewer (V.K.) was consulted.

### Data Synthesis

Meta-analysis was performed using Review Manager software, version 5.4 (Cochrane). Due to the variability in interventions and population demographics, a random-effects model was used.^18^ For discrete outcomes, relative risk (eg, complication rate) and 95% CIs were calculated. For continuous outcomes, (eg, pain score), weighted mean differences (WMD) and 95% CI were calculated after conversion to a scale from 0 to 100, in which a higher score indicated a worse outcome. Units of measurement were standardized to metric (eg, pounds to kilograms, calculated by multiplying by 0.45). Where scales were different, or conversion was not possible, data were pooled using standardized mean difference (SMD). Pain scores during walking were preferred to pain at rest. Muscle strength reported on the affected side was used. Where SE was reported instead of SD, SD was calculated using the formula SD = SE × √*n*. If change scores were reported, the follow-up scores were calculated as the sum of change scores and baseline scores.^19^ Data were pooled into 5 periods: preoperatively after prehabilitation and 6 weeks, 3 months, 6 months, and 12 months or more postoperatively. In instances where 2 follow-up end points fell into the same period, the later scores were used (eg, for 4-week and 6-week postoperative end points, those at 6 weeks were used).

### GRADE Certainty Assessment

The Grading of Recommendations Assessment, Development and Evaluation system (GRADE) and software (GRADE Pro GDT) were used to rate each outcome.^20,21^ The overall certainty of evidence was categorized into 3 levels: high, moderate, and low or very low based on the 5 GRADE domains.^22^ Publication bias was detected using funnel plots if data were pooled from 10 or more trials.^23^

## Results

### Study Selection

**Figure 1.**
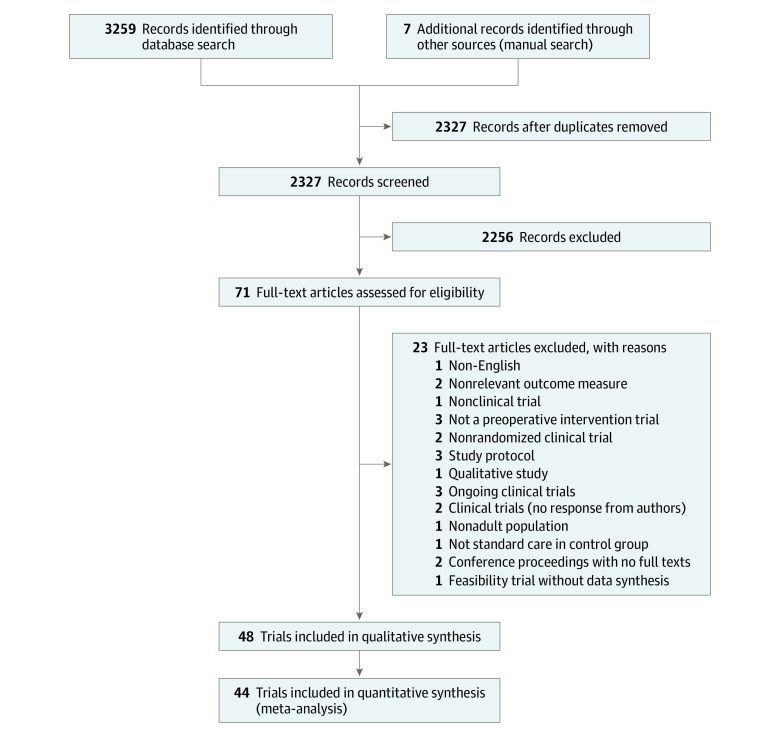
Study Flow Diagram

Database searches identified 3259 citations (Figure 1). Seven additional articles were identified through hand searching. After removal of duplicates, 2327 articles remained. Upon screening of the title and abstract, 2256 were excluded as they did not meet the eligibility criteria. The remaining 71 full texts were reviewed and 21 were excluded.^24,25,26,27,28,29,30,31,32,33,34,35,36,37,38,39,40,41,42,43,44^ Additionally, authors of 2 conference proceedings were contacted on 2 separate occasions with no response and therefore were excluded at the full text stage.^45,46^ Five follow-up trials were considered with their primary trials to avoid publication bias.^47,48,49,50,51^

### Study Characteristics

Full details of included trials, their population characteristics and results are detailed in eTable 2 in Supplement 1. Forty-eight trials (46 published^52,53,54,55,56,57,58,59,60,61,62,63,64,65,66,67,68,69,70,71,72,73,74,75,76,77,78,79,80,81,82,83,84,85,86,87,88,89,90,91,92,93,94,95,96,97^ and 2 trials from the Clinical Trials Registry^98,99^) containing 3570 patients (2196 women [61.5%] and 1374 men [38.5%]; mean [SD] age, 64.1 [9.1] years) were included. Data reported on the Clinical Trials Registry were used for one trial^99^ because published data reported on the postoperative period only.^100^ The author of the other unpublished study provided data upon request.^98^ Twenty-eight trials (58%)^34,52,55,56,57,61,62,66,69,71,72,75,76,77,80,84,85,86,87,89,90,92,93,94,95,96,98,99^ evaluated the effectiveness of prehabilitation for TKR alone, 7 for THR alone,^53,63,64,67,68,79,91^ 6 for THR and TKR combined,^54,58,59,60,82,88^ 5 for lumbar surgery,^51,73,78,81,97^ 1 for anterior cruciate ligament reconstruction,^83^ and 1 for femoroacetabular impingement syndrome.^65^

### Type of Interventions and Dosage and Components of Prehabilitation Interventions

Details of the interventions, dosage, and adherence are detailed in eTable 3 in Supplement 1. Thirty-nine trials (81%)^52,55,56,57,58,59,60,61,62,63,64,65,66,67,68,69,70,71,72,74,75,76,77,78,79,82,83,84,85,86,87,88,90,91,94,95,96,97,98^ used a variety of exercise interventions such as strengthening, balance, proprioception, or aquatic training. Of these, 10 trials (21%) used exercises in combination with preoperative education,^34,52,53,59,64,65,69,72,80,94^ 2 (4%) with acupuncture,^85,90^ and 3 (6%) with neuromuscular electrical stimulation.^89,93,99^ Only 5 trials (10%) were multimodal in nature.^53,59,85,90,97^ Thirty-nine trials (81%)^52,53,54,55,56,57,60,61,63,64,65,66,67,69,70,71,72,73,74,75,76,77,78,82,83,84,85,86,87,88,89,90,91,92,93,94,96,97,98,99^ implemented prehabilitation for a duration of at least 4 weeks. Thirty-seven trials (77%)^52,55,56,57,58,60,61,62,63,64,65,66,67,68,71,72,73,74,75,76,77,78,79,82,83,84,86,87,88,89,91,92,93,94,95,96,97,98,99^ reported at least 2 sessions of prehabilitation per week, and 26 (54%)^52,53,57,60,61,62,63,67,70,73,74,75,76,77,81,82,83,84,85,88,90,92,94,95,96,97^ implemented supervised prehabilitation sessions either as an individual or as a group in clinic or at home. Three trials delivered prehabilitation virtually (ie, via teleprehabilitation^60,94^ and telephone^80^). Adherence was only reported in 21 trials (44%),^52,60,61,67,68,70,74,75,76,78,79,80,81,82,83,84,86,88,89,91,97^ with 19 trials^52,60,61,67,68,70,74,75,76,78,79,80,82,83,84,86,88,89,91^ reporting adherence as moderate (>70%).

### Risk of Bias

Risk of bias assessment is provided in eFigures 1 and 2 in Supplement 1. There was selective reporting in 33 trials,^52,53,54,55,56,57,59,62,63,64,66,68,69,70,71,72,75,76,77,79,81,82,83,86,87,89,90,91,92,93,96,98,99^ insufficient details on attrition in 8 trials,^53,59,61,70,72,75,91,98^ inadequate explanation of allocation concealment in 24 trials,^53,54,55,56,57,63,64,65,69,71,72,75,79,82,86,87,89,91,92,93,95,96,98,99^ lack of assessor blinding in 22 trials,^53,54,55,56,62,66,67,69,71,72,75,76,79,81,83,86,92,93,95,97,98,99^ and inadequate randomization in 9 trials.^69,71,75,76,86,92,93,98,99^ Only 10 trials (21%) had low overall risk of bias.^51,58,60,73,78,80,84,85,88,94^

### Results of Syntheses and Certainty of Evidence

The GRADE summary of findings for all outcome measures for each orthopedic surgical procedure is provided in eTables 4 to 7 in Supplement 1. Publication bias was only considered for trials involving TKR.

### Association of Prehabilitation With Primary Outcomes

#### Preoperative Phase (After Prehabilitation)

**Figure 2.**
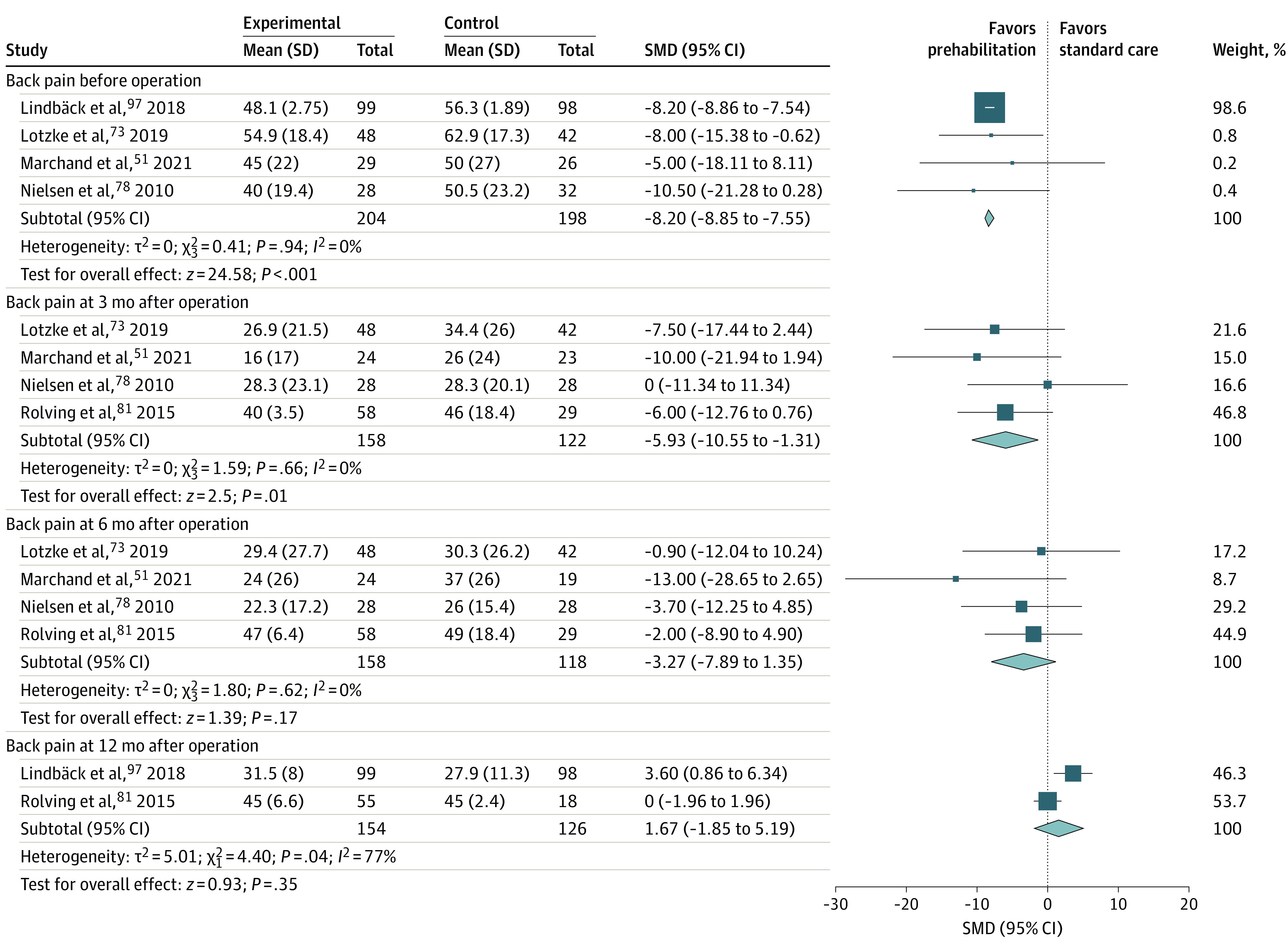
Forest Plot of Mean Differences in Back Pain Before and After Lumbar Surgery The size of the squares is proportional to the weight of each study. Horizontal lines indicate the 95% CI of each study; diamond, the pooled estimate with 95% CI; and vertical line the line of no effect. SMD indicates standardized mean difference.

**Figure 3.**
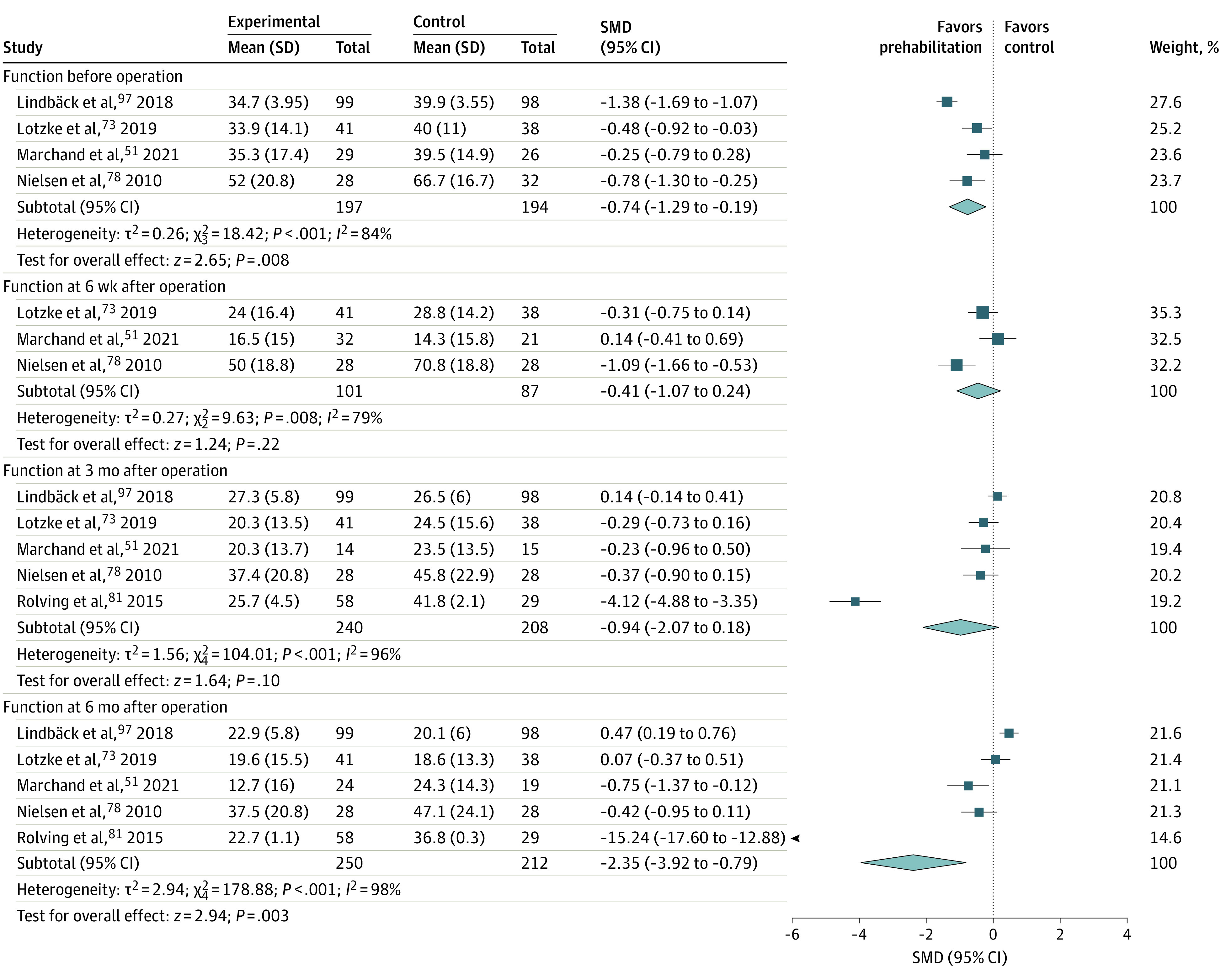
Forest Plot of Standardized Mean Differences in Function Before and After Lumbar Surgery The size of the squares is proportional to the weight of each study. Horizontal lines indicate the 95% CI of each study; diamond, the pooled estimate with 95% CI; and vertical line, the line of no effect. SMD indicates standardized mean difference.

Reduction in preoperative pain following prehabilitation was statistically significant for THR (8 trials [n = 340]^53,60,63,67,68,79,82,91^; SMD, −0.47 [95% CI, −0.69 to −0.25]; GRADE low [eFigure 3 in Supplement 1]), TKR (18 trials [n = 1138]^52,57,60,61,69,70,76,82,84,85,86,87,89,90,92,93,94,98^; SMD, −0.58 [95% CI, −0.88 to −0.28]; GRADE low [eFigure 4 in Supplement 1]), and lumbar surgery for back pain (4 trials [n = 402]^51,73,78,97^; mean difference, –8.20 [95% CI, −8.85 to −7.55]; GRADE high [Figure 2]). Function was also noted to improve significantly following prehabilitation for THR (8 trials [n = 359]^60,63,64,67,68,79,82,91^; SMD, −0.54 [95% CI, −0.78 to −0.28]; GRADE low [eFigure 5 in Supplement 1]),TKR (14 trials [n = 575]^52,57,60,61,62,70,71,76,82,84,89,92,93,94^; SMD, −0.70 [95% CI, −1.08 to −0.32]; GRADE moderate) [eFigure 6 in Supplement 1]), and lumbar surgery (4 trials [n = 391]^51,73,78,97^; SMD, −0.74 [95% CI, −1.11 to −0.69]; GRADE low [Figure 3]).

Improvement in HRQOL was statistically significant in THR using the 36-item Short Form Health Survey (2 trials [n = 40]^60,63^; mean difference, 7.35 [95% CI, 3.15-11.54]; GRADE moderate [eFigure 7 in Supplement 1]) and lumbar surgery (2 trials [n = 149]^73,78^; SMD, 0.46 [95% CI, 0.13-0.78]; GRADE moderate [eFigure 8 in Supplement 1]). Improvements in muscle strength were also reported to be statistically significant for hip abductors in patients undergoing THR (2 trials [n = 107]^63,88^; SMD, 1.03 [95% CI, 0.03- 2.02]; GRADE moderate [eFigure 9 in Supplement 1]), knee flexor strength in patients undergoing TKR (7 trials [n = 349]^52,57,70,72,84,86,92^; SMD, 1.00 [95% CI, 0.23-1.77]; GRADE moderate [eFigure 10 in Supplement 1]), and knee extensor strength (13 trials [n = 632]^52,57,61,70,72,84,86,88,92,93,94,95,98^; SMD, 0.72 [95% CI, 0.28-1.15]; GRADE low [eFigure 11 in Supplement 1]). Other meta-analyses completed at follow-up time points to 1 year reported no significant differences (eFigures 3, 4, 8, and 12-16 in Supplement 1).

#### Postoperative Phase (After Surgery)

Reduction in back pain at 3 months in the prehabilitation group was noted to be statistically significant in patients undergoing lumbar surgery (4 trials [n = 280]^51,73,78,81^; mean difference, −5.93 [95% CI, −10.55 to −1.31]; GRADE low) [Figure 2]). Function significantly improved for TKR at 6 weeks (10 trials [n = 451]^61,66,70,75,76,84,88,89,93,94^; SMD, −0.51 [95% CI, −0.85 to −0.17]; GRADE moderate [eFigure 6 in Supplement 1]) and at 3 months (17 trials [n = 1086]^52,55,57,58,70,71,75,76,77,80,82,84,88,89,92,93,99^; SMD, −0.29 [95% CI, −0.51 to −0.08]; GRADE low [eFigure 6 in Supplement 1]), for THR at 3 months (6 trials [n = 310]^58,63,64,67,82,88^; SMD, −0.38 [95% CI, −0.62 to −0.14]; GRADE low [eFigure 5 in Supplement 1]) and at 12 months (3 trials [n = 160]^53,64,67^; SMD, −0.34 [95% CI, −0.65 to −0.02]; GRADE low [eFigure 5 in Supplement 1]), and for lumbar surgery where isolated differences are reported at 6 months (5 trials [n = 448]^51,73,78,81,97^; SMD, −2.35 [95% CI, −3.92 to −0.79]; GRADE moderate [Figure 3]). A significant difference was also reported in HRQOL only for TKR at 6 weeks (6 trials [n = 330]^61,70,75,84,88,93^; WMD, 5.66 [95% CI, 2.04-9.27]; GRADE low [eFigure 17 in Supplement 1]) and 3 months (4 trials [n = 177]^52,57,76,89^; WMD, 1.89 [95% CI, 0.64-3.14]; GRADE low [eFigure 18 in Supplement 1]). Prehabilitation also improved muscle strength for TKR to 6 weeks for flexor strength (2 trials [n = 69]^72,84^; SMD, 0.72 [95% CI, 0.23-1.21]; GRADE low [eFigure 19 in Supplement 1]) and for extensor strength (8 trials [n = 347]^61,72,76,84,87,93,94,99^; SMD, 0.45 [95% CI, 0.06-0.84]; GRADE low [eFigure 20 in Supplement 1]). Meta-analyses for all other primary outcomes completed at follow-up time points to 1 year reported no significant differences (eFigures 21-29 in Supplement 1).

### Association of Prehabilitation With Secondary Outcomes

Prehabilitation and its association with secondary outcomes are described in eResults in Supplement 1. Meta-analysis for all secondary outcomes is demonstrated in eFigures 30 to 44 in Supplement 1.

## Discussion

This is the first systematic review with meta-analysis of RCTs, to our knowledge, to provide level I evidence suggesting that prehabilitation programs are associated with improvements in preoperative outcomes for patients undergoing all orthopedic surgical procedures. Specifically, there is moderate-certainty evidence for function, knee flexor strength, and 6-minute walk test performance for TKR, abduction strength for THR, and HRQOL for THR and lumbar surgery and high-certainty evidence for lumbar surgery and back pain. For the outcomes outlined, effects of prehabilitation were moderate to large or met the minimal clinically important difference for that measure (eTables 4-7 in Supplement 1), which should improve postoperative outcomes.

However, the quality of evidence was inconsistent at various time points postoperatively, with moderate-certainty evidence favoring prehabilitation only for function at 6 weeks in TKR and at 6 months in lumbar surgery. Although prehabilitation showed statistically significant differences over usual care in other outcome measures (pain, range of motion, and functional performance measures like the timed up and go and stair tests), the overall quality of the evidence was low to very low. This supports the need for RCTs with low risk of bias (selective reporting, assessor blinding, concealment) including these outcome measures to further research prehabilitation for orthopedic surgical procedures. The evidence favoring prehabilitation at various outcome assessment points is summarized in the Table.

**Table.** Summary of Evidence Favoring Prehabilitation

Outcome assessment point	Certainty of evidence	Procedure	Statistically significant outcomes favoring prehabilitation
Preoperative	High	Lumbar surgery	Back pain
	Moderate	TKR	Function
			Knee flexor strength
			6MWT
		THR	HRQOL
			Hip Abductor strength
		Lumbar surgery	HRQOL
	Low	TKR	Pain
			Knee extensor strength
			Knee flexion ROM
			TUG
			Stair test
		THR	Pain
			Function
			HRQOL
		Lumbar surgery	Function
6 wk Postoperative	Moderate	TKR	Function
	Low	TKR	Function
			HRQOL
			Knee flexor ROM
			Knee flexor strength
			Knee extensor strength
			TUG
3 mo Postoperative	Low	TKR	Function
			HRQOL
			Stair test
		THR	Function
		Lumbar surgery	Back pain
6 mo Postoperative	Moderate	Lumbar surgery	Function
12 mo Postoperative	Low	THR	Function

Abbreviations: 6MWT, 6-minute walk test; HRQOL, Health-Related Quality of Life; ROM, range of motion; THR, total hip replacement; TKR, total knee replacement; TUG, timed up and go test.

Numerous factors such as preoperative muscle strength, function, and HRQOL are predictive factors associated with postoperative function in the population undergoing orthopedic surgery.^5,6,7,8^ Poor fitness and deconditioning could negatively influence postoperative outcomes.^101^ Additionally, postponement of elective operations due to the pandemic have led to increased waiting times, poorer access to support services, and heightened anxiety leading to further chronicity of symptoms and deconditioning of the patients.^102^ During this period, prehabilitation interventions may play a major role in optimizing patients prior to undergoing surgical procedures. Although there was no evidence of a correlation of prehabilitation in improving anxiety or depression in our review, there is a growing body of evidence on the role of psychological factors and their influence on surgical outcomes.^103^ Future prehabilitation programs should look at being more multimodal in nature and include psychological interventions alongside exercises that may address factors such as anxiety and depression prior to surgical intervention.

Although prehabilitation was associated with improvement in various outcome domains preoperatively, the quality of evidence for postoperative outcomes was low to very low. Variables such as types of surgical procedure, postoperative pain management, and variability in rehabilitation services on discharge from the acute services can influence postoperative recovery and therefore cannot be attributable to prehabilitation alone.^104,105,106^ Standardizing these factors may provide a clearer understanding of the association of prehabilitation with postoperative outcomes.

In this systematic review, adherence was reported only in 21 trials (44%)^52,60,61,67,68,70,74,75,76,78,79,80,81,82,83,84,86,88,89,91,97^ and was shown as moderate (>70% adherence in 19 of 21 trials^52,60,61,67,68,70,74,75,76,78,79,80,82,83,84,86,88,89,91^). One major factor that could contribute to low adherence could be increased pain while performing the exercises. Forty-one trials (85%)^52,53,54,55,56,57,58,59,60,61,62,63,64,66,67,68,69,70,71,72,75,76,77,79,80,82,84,85,86,87,88,89,90,91,92,93,94,95,96,98,99^ in this review included patients with degenerative joint diseases awaiting joint replacement surgery. Exercises loading the joints can induce further discomfort and could result in low adherence to the exercise program that can affect treatment outcomes, particularly in older people with long-term conditions such as osteoarthritis.^107^ Trials researching exercise regimens with reduced joint mechanical load such as aquatic therapy and neuromuscular stimulation (eTable 3 in Supplement 1) have shown some promising results as adjuncts in prehabilitation programs.^108,109,110^ Additionally, adherence to self-directed exercises outside the treatment period is crucial in maintaining effects of rehabilitation in the longer term.^107^ Low self-efficacy and motivation and lack of support post discharge may result in reduced exercise compliance and reduce the positive benefits gained preoperatively.^111^

Both supervised and unsupervised sessions are used by trials included in this review. Previous trials have shown the benefits of exercises supervised by qualified physical therapists either at home or in a clinic setting.^112,113^ Supervision allows greater adaptations and improved compliance with exercises, especially in older adults.^113,114^ However, availability of transport and costs are major barriers to compliance when treatment is delivered at clinics or hospitals.^115^ Therefore, use of technology such telerehabilitation may play a role. Telerehabilitation has shown similar results compared with traditional physical therapy in patients undergoing lower limb arthroplasties.^116,117^ Therefore, careful consideration of all the above is needed to aid decision making on the method of intervention delivery in future trials.

Thirty-seven trials (77%)^52,55,56,57,58,60,61,62,63,64,65,66,67,68,71,72,73,74,75,76,77,78,79,82,83,84,86,87,88,89,91,92,93,94,95,96,97,98,99^ included in this review delivered prehabilitation twice a week over at least 4 weeks. This finding is in accordance with a recent umbrella review of 55 systematic reviews on prehabilitation for patients undergoing major surgical procedures^118^ and physiological literature to indicate 4 to 6 weeks of strength training is required to induce neurological and morphological muscular adaptations.^119^

### Strengths and Limitations

This comprehensive review used rigorous methods in accordance with international reporting guidance. However, most of the trials included in this review were on joint replacements and lumbar surgical procedures. Studies on other procedures were sparse, and therefore results from this review may not be applicable to other surgical procedures. Although there is a strong drive toward performing arthroplasties as day cases, this review did not identify any trials on day case procedures.^120,121^ Included trials showed moderate-to-high risk of bias and heterogeneity in the meta-analysis, which commonly reduced certainty of evidence recommendations to low or very low. Moderate- and high-certainty evidence (eg, Figure 2 and eFigures 6 and 22 in Supplement 1) have instances of high heterogeneity that may be attributable to different trial characteristics such as varying prehabilitation interventions.

## Conclusions

In this systematic review and meta-analysis of RCTs, prehabilitation was associated with moderate improvement in several preoperative outcomes (function, knee flexor and hip abductor strength, HRQOL, 6-minute walk test) among patients undergoing all orthopedic procedures and was also associated with a reduction in back pain among patients undergoing lumbar surgery. However, the evidence was inconsistent and the quality of evidence for postoperative outcomes was low to very low. A minimum duration of 4 to 6 weeks and 2 sessions per week may be recommended for patients undergoing orthopedic surgery. Prehabilitation programs with a combination of supervised and unsupervised sessions can be safely administered with minimal risks. Additional RCTs with a low risk of bias investigating preoperative and postoperative outcomes for all orthopedic surgical procedures are required.

## Notes

Supplement 1. **eTable 1.** Search Strategy **eTable 2.** Study Characteristics and Summary Results **eTable 3.** Details of Interventions, Dosage, and Compliance Rates **eFigure 1.** Risk of Bias for Individual Trials **eFigure 2.** Risk of Bias Summary Graph **eTable 4.** GRADE Summary of Findings: TKR **eTable 5.** GRADE Summary of Findings: THR **eTable 6.** GRADE Summary of Findings: Lumbar Surgery **eTable 7.** GRADE Summary of Findings: THR and TKR **eFigure 3.** Pain: Preoperative and 3 Months and 6 Months Postoperative for THR **eFigure 4.** Pain: Preoperative and 3 Months, 6 Months, and 12 Months Postoperative for Total Knee Replacement Surgery (TKR) **eFigure 5.** Function: Preoperative and 3 Months, 6 Months, and 12 Months Postoperative for THR **eFigure 6.** Function: Preoperative and 3 Months, 6 Months, and 12 Months Postoperative for TKR **eFigure 7.** Health-Related Quality of Life (HRQOL): Preoperative for THR on SF-36 **eFigure 8.** HRQOL: Preoperative and 3 Months Postoperative for Spinal Surgery **eFigure 9.** Isometric Hip Abductor Strength: Preoperative for THR **eFigure 10.** Knee Flexor Strength: Preoperative for TKR **eFigure 11.** Knee Extensor Strength: Preoperative for TKR **eFigure 12.** Leg Pain: Preoperative and 3 Months, 6 Months, and 12 Months Postoperative for Spinal Surgery **eFigure 13.** Anxiety and Depression on Hospital Anxiety and Depression Scale (HADS): Preoperative and 3 Months Postoperative for TKR **eFigure 14.** Depression: Preoperative and 3 Months Postoperative for Spinal Surgery **eFigure 15.** HRQOL: Preoperative for TKR on SF-36 **eFigure 16.** Isometric Hip Extensor Strength: Preoperative for THR **eFigure 17.** HRQOL on KOOS: 6 Weeks Postoperative for TKR **eFigure 18.** HRQOL: 3 Months Postoperative for TKR on SF-36 **eFigure 19.** Knee Flexor Strength: 6 Weeks Postoperative for TKR **eFigure 20.** Knee Extensor Strength: 6 Weeks Postoperative **eFigure 21.** Pain on HOOS: 6 Weeks Postoperative for THR **eFigure 22.** Function: 6 Months and 12 Months for TKR **eFigure 23.** Function on HOOS: 6 Weeks Postoperative for THR **eFigure 24.** HRQOL on HOOS: 6 Weeks Postoperative for THR **eFigure 25.** HRQOL: 3 Months Postoperative for THR **eFigure 26.** Knee Flexor Strength: 3 Months Postoperative **eFigure 27.** Knee Extensor Strength: 3 Months Postoperative **eFigure 28.** Knee Flexor Strength: 12 Months Postoperative for TRK **eFigure 29.** Knee Extensor Strength: 12 Months Postoperative for TRK **eResults.** Association of Prehabilitation With Secondary Outcomes **eFigure 30.** Active Knee Flexion Range of Motion (ROM): Preoperative and 6 Weeks, 3 Months, and 12 Months Postoperative for TKR **eFigure 31.** 6-Minute Walk Test (6MWT): Preoperative and 6 Weeks, 3 Months, and 12 Months Postoperative for TKR **eFigure 32.** TUG: Preoperative and 6 Weeks, 3 Months, and 12 Months Postoperative for TKR **eFigure 33.** Stair Test: Preoperative and 6 Weeks and 3 Months Postoperative for TKR **eFigure 34.** Active Knee Extension ROM: Preoperative and 6 Weeks, 3 Months, and 12 Months Postoperative for TKR **eFigure 35.** 6MWT: Preoperative for THR **eFigure 36.** 30-Second Chair Rise Test: Preoperative and 6 Weeks and 3 Months Postoperative for TKR **eFigure 37.** Functional Reach: Preoperative and 6 Weeks Postoperative for TKR **eFigure 38.** Timed Up and Go Test (TUG): Preoperative and 6 Weeks Posterative for THR **eFigure 39.** Total Length of Stay for THR and TKR **eFigure 40.** Length of Stay for Spinal Surgical Procedures **eFigure 41.** Health Care Costs for TKR **eFigure 42.** Readmission Rates for TKR **eFigure 43.** Total Complication Rates for THR and TKR **eFigure 44.** Complication Rates for Spinal Surgery
eReferences

Supplement 2.
Data Sharing Statement
