# Supplementary material for: Prehabilitation for Patients Undergoing Orthopedic Surgery: A Systematic Review and Meta-analysis
Source: JAMA Netw Open. 2023 Apr 13;6(4):e238050. doi: 10.1001/jamanetworkopen.2023.8050 (PMC10102876; doi:10.1001/jamanetworkopen.2023.8050)
Supplement: Supplement 1. — eTable 1. Search Strategy eTable 2. Study Characteristics and Summary Results eTable 3. Details of Interventions, Dosage, and Compliance Rates eFigure 1. Risk of Bias for Individual Trials eFigure 2. Risk of Bias Summary Graph eTable 4. GRADE Summary of Findings: TKR eTable 5. GRADE Summary of Findings: THR eTable 6. GRADE Summary of Findings: Lumbar Surgery eTable 7. GRADE Summary of Findings: THR and TKR eFigure 3. Pain: Preoperative and 3 Months and 6 Months Postoperative for THR eFigure 4. Pain: Preoperative and 3 Months, 6 Months, and 12 Months Postoperative for Total Knee Replacement Surgery (TKR) eFigure 5. Function: Preoperative and 3 Months, 6 Months, and 12 Months Postoperative for THR eFigure 6. Function: Preoperative and 3 Months, 6 Months, and 12 Months Postoperative for TKR eFigure 7. Health-Related Quality of Life (HRQOL): Preoperative for THR on SF-36 eFigure 8. HRQOL: Preoperative and 3 Months Postoperative for Spinal Surgery eFigure 9. Isometric Hip Abductor Strength: Preoperative for THR eFigure 10. Knee Flexor Strength: Preoperative for TKR eFigure 11. Knee Extensor Strength: Preoperative for TKR eFigure 12. Leg Pain: Preoperative and 3 Months, 6 Months, and 12 Months Postoperative for Spinal Surgery eFigure 13. Anxiety and Depression on Hospital Anxiety and Depression Scale (HADS): Preoperative and 3 Months Postoperative for TKR eFigure 14. Depression: Preoperative and 3 Months Postoperative for Spinal Surgery eFigure 15. HRQOL: Preoperative for TKR on SF-36 eFigure 16. Isometric Hip Extensor Strength: Preoperative for THR eFigure 17. HRQOL on KOOS: 6 Weeks Postoperative for TKR eFigure 18. HRQOL: 3 Months Postoperative for TKR on SF-36 eFigure 19. Knee Flexor Strength: 6 Weeks Postoperative for TKR eFigure 20. Knee Extensor Strength: 6 Weeks Postoperative eFigure 21. Pain on HOOS: 6 Weeks Postoperative for THR eFigure 22. Function: 6 Months and 12 Months for TKR eFigure 23. Function on HOOS: 6 Weeks Postoperative for THR eFigure 24. HRQOL on HO [file jamanetwopen-e238050-s001.pdf]

Supplementary Online Content

Punnoose A, Claydon-Mueller LS, Weiss O, Zhang J, Rushton A, Khanduja V. Prehabilitation for patients undergoing orthopedic surgery: a systematic review and meta-analysis. *JAMA Netw Open*. 2023;6(4):e238050. doi:10.1001/jamanetworkopen.2023.8050

eTable 1. Search Strategy
eTable 2. Study Characteristics and Summary Results
eTable 3. Details of Interventions, Dosage, and Compliance Rates
eFigure 1. Risk of Bias for Individual Trials
eFigure 2. Risk of Bias Summary Graph
eTable 4. GRADE Summary of Findings: TKR
eTable 5. GRADE Summary of Findings: THR
eTable 6. GRADE Summary of Findings: Lumbar Surgery
eTable 7. GRADE Summary of Findings: THR and TKR
eFigure 3. Pain: Preoperative and 3 Months and 6 Months Postoperative for THR
eFigure 4. Pain: Preoperative and 3 Months, 6 Months, and 12 Months Postoperative for Total Knee Replacement Surgery (TKR)
eFigure 5. Function: Preoperative and 3 Months, 6 Months, and 12 Months Postoperative for THR
eFigure 6. Function: Preoperative and 3 Months, 6 Months, and 12 Months Postoperative for TKR
eFigure 7. Health-Related Quality of Life (HRQOL): Preoperative for THR on SF-36
eFigure 8. HRQOL: Preoperative and 3 Months Postoperative for Spinal Surgery
eFigure 9. Isometric Hip Abductor Strength: Preoperative for THR
eFigure 10. Knee Flexor Strength: Preoperative for TKR
eFigure 11. Knee Extensor Strength: Preoperative for TKR
eFigure 12. Leg Pain: Preoperative and 3 Months, 6 Months, and 12 Months Postoperative for Spinal Surgery
eFigure 13. Anxiety and Depression on Hospital Anxiety and Depression Scale (HADS): Preoperative and 3 Months Postoperative for TKR
eFigure 14. Depression: Preoperative and 3 Months Postoperative for Spinal Surgery
eFigure 15. HRQOL: Preoperative for TKR on SF-36
eFigure 16. Isometric Hip Extensor Strength: Preoperative for THR
eFigure 17. HRQOL on KOOS: 6 Weeks Postoperative for TKR
eFigure 18. HRQOL: 3 Months Postoperative for TKR on SF-36
eFigure 19. Knee Flexor Strength: 6 Weeks Postoperative for TKR
eFigure 20. Knee Extensor Strength: 6 Weeks Postoperative
eFigure 21. Pain on HOOS: 6 Weeks Postoperative for THR
eFigure 22. Function: 6 Months and 12 Months for TKR
eFigure 23. Function on HOOS: 6 Weeks Postoperative for THR
eFigure 24. HRQOL on HOOS: 6 Weeks Postoperative for THR
eFigure 25. HRQOL: 3 Months Postoperative for THR
eFigure 26. Knee Flexor Strength: 3 Months Postoperative
eFigure 27. Knee Extensor Strength: 3 Months Postoperative
eFigure 28. Knee Flexor Strength: 12 Months Postoperative for TRK
eFigure 29. Knee Extensor Strength: 12 Months Postoperative for TRK
eResults. Association of Prehabilitation With Secondary Outcomes
eFigure 30. Active Knee Flexion Range of Motion (ROM): Preoperative and 6 Weeks, 3 Months, and 12 Months Postoperative for TKR
eFigure 31. 6-Minute Walk Test (6MWT): Preoperative and 6 Weeks, 3 Months, and 12 Months Postoperative for TKR
eFigure 32. TUG: Preoperative and 6 Weeks, 3 Months, and 12 Months Postoperative for TKR
eFigure 33. Stair Test: Preoperative and 6 Weeks and 3 Months Postoperative for TKR
eFigure 34. Active Knee Extension ROM: Preoperative and 6 Weeks, 3 Months, and 12 Months Postoperative for TKR
eFigure 35. 6MWT: Preoperative for THR
eFigure 36. 30-Second Chair Rise Test: Preoperative and 6 Weeks and 3 Months Postoperative for TKR

© 2023 Punnoose A et al. *JAMA Network Open*.

eFigure 37. Functional Reach: Preoperative and 6 Weeks Postoperative for TKR
eFigure 38. Timed Up and Go Test (TUG): Preoperative and 6 Weeks Posterative for THR
eFigure 39. Total Length of Stay for THR and TKR
eFigure 40. Length of Stay for Spinal Surgical Procedures
eFigure 41. Health Care Costs for TKR
eFigure 42. Readmission Rates for TKR
eFigure 43. Total Complication Rates for THR and TKR
eFigure 44. Complication Rates for Spinal Surgery
eReferences

This supplementary material has been provided by the authors to give readers additional information about their work.

eTable 1. Search Strategy

MEDLINE (Ovid)

#	**Search terms**	**No. of studies**
1	((Rehab* or re-hab* or Physiotherap* or physical therap*) adj3 (Prior or before or ahead or pre-op* or preop* or pre-surger* or presurger*)).ti,ab,kw,kf. or ((exp Physical Therapy Modalities/ or exp Exercise Therapy/ or exp Rehabilitation/) and (exp Preoperative Care/ or exp Preoperative Period/))	4342
2	(Prehabilit* or Pre-habilit* or Pre habilitat*).ti,ab,kw,kf. or exp Preoperative Exercise	1411
3	1 OR 3	5535
4	randomized controlled trial/ or Random Allocation/ or clinical trial/ or (clinical adj trial$).ti,ab. or ((singl$ or doubl$ or treb$ or tripl$) adj (blind$3 or mask$3)).ti,ab. or randomly allocated.ti,ab. or (allocated adj2 random$).ti,ab. or trial*.ti,ab. or randomized controlled trial.pt. or controlled clinical trial.pt.	1935421
5	3 AND 4	1359
6	limit 5 to yr="2000 -Current"	1232

EMBASE

#	**Search terms**	**No. of studies**
1	((Rehab* or re-hab* or Physiotherap* or physical therap*) adj3 (Prior or before or ahead or pre-op* or preop* or pre-surger* or presurger*)).ti,ab. or ((exp *physiotherapy/ or exp *kinesiotherapy/ or exp *Rehabilitation/) and (exp *Preoperative Care/ or exp *Preoperative Period/ or exp *preoperative treatment/))	4664
2	(Prehabilit* or Pre-habilit* or Pre habilitat*).ti,ab. or exp *Preoperative Exercise/	2025
3	1 OR 2	6498
4	*randomized controlled trial/ or *randomization/ or *clinical trial/ or (clinical adj trial$).ti,ab. or ((singl$ or doubl$ or treb$ or tripl$) adj (blind$3 or mask$3)).ti,ab. or (allocat* adj2 random$).ti,ab. or trial*.ti,ab.	1925482
5	3 AND 4	1139
6	limit 5 to yr="2000 -Current"	1110
7	limit 6 to embase	591

AMED

#	**Search terms**	**No. of studies**
1	((Rehab* or re-hab* or Physiotherap* or physical therap*) adj3 (Prior or before or ahead or pre-op* or preop* or pre-surger* or presurger*)).ti,ab. or ((exp physical therapy speciality/ or exp Rehabilitation/ or exp exercise therapy/) and (exp preoperative care/ or exp Preoperative Period/))	301
2	(Prehabilit* or Pre-habilit* or Pre habilitat*).ti,ab.	41
3	1 OR 2	332
4	exp Clinical trials/ or exp Randomized controlled trials/ or exp random allocation/ or (clinical adj trial$).ti,ab. or ((singl$ or doubl$ or treb$ or tripl$) adj (blind$3 or mask$3)).ti,ab. or randomly allocated.ti,ab. or (allocated adj2 random$).ti,ab. or trial*.ti,ab.	24644
5	3 AND 4	74
6	limit 5 to yr="2000 -Current"	70

© 2023 Punnoose A et al. *JAMA Network Open*.

PEDRO

#	**Search terms**	**No. of studies**
1	Pre-habiliation – title/abstract	72
2	Physiotherapy and preoperative – title/abstract	61
3	Exercise therapy and preoperative – title/abstract	28
4	Exercise therapy and presurgery – title/abstract	0
5	Exercise therapy and pre-surgery – title/abstract	17
6	Physiotherapy and pre-surgery – title/abstract	46
	Total	197

© 2023 Punnoose A et al. *JAMA Network Open*.

CINAHL

#	Query	Limiters/Expanders	Last Run Via	Results
S5	S3 AND S4	Expanders - Apply equivalent subjects<br>Search modes - Boolean/Phrase	Interface - EBSCOhost Research Databases<br>Search Screen - Advanced Search<br>Database - CINAHL	238
S4	TI ((“clinical trial*”) or ((singl* or doubl* or treb* or tripl*) n1 (blind* or mask*) or “randomly allocated” or (allocated n2 random*) or trial*) or AB((“clinical trial*”) or ((singl* or doubl* or treb* or tripl*) n1 (blind* or mask*) or “randomly allocated” or (allocated n2 random*) or trial*) or (MH "Randomized Controlled Trials+") OR (MH "Clinical Trials+") OR (MH "Therapeutic Trials+")	Expanders - Apply equivalent subjects<br>Search modes - Boolean/Phrase	Interface - EBSCOhost Research Databases<br>Search Screen - Advanced Search<br>Database - CINAHL	592,293
S3	S1 OR S2	Expanders - Apply equivalent subjects<br>Search modes - Boolean/Phrase	Interface - EBSCOhost Research Databases<br>Search Screen - Advanced Search<br>Database - CINAHL	975
S2	TI (Prehabilit* or Pre-habilit* or Pre habilitat*) or AB(Prehabilit* or Pre-habilit* or Pre habilitat*) or (MH "Prehabilitation")	Expanders - Apply equivalent subjects<br>Search modes - Boolean/Phrase	Interface - EBSCOhost Research Databases<br>Search Screen - Advanced Search<br>Database - CINAHL	701
S1	TI((Rehab* or re-hab* or Physiotherap* or physical therap*) n3 (Prior or before or ahead or pre-op* or preop* or pre-surger* or presurger*)) or AB((Rehab* or re-hab* or Physiotherap* or physical therap*) n3 (Prior or before or ahead or pre-op* or preop* or pre-surger* or presurger*)) or or (((MH "Physical Therapy+") OR (MH "Therapeutic Exercise+") OR (MH "Rehabilitation+")) and ((MH "Preoperative Care") OR (MH "Preoperative Period+")))	Expanders - Apply equivalent subjects<br>Search modes - Boolean/Phrase	Interface - EBSCOhost Research Databases<br>Search Screen - Advanced Search<br>Database - CINAHL	294

© 2023 Punnoose A et al. *JAMA Network Open*.

COCHRANE CENTRAL

#	**Search terms**	**No. of studies**
1	((Rehab* or re-hab* or Physiotherap* or physical therap*) near (Prior or before or ahead or pre-op* or preop* or pre-surger* or presurger*)):ti,ab,kw	27747
2	MeSH descriptor: [Physical Therapy Modalities] explode all trees	30119
3	MeSH descriptor: [Exercise Therapy] explode all trees	16536
4	MeSH descriptor: [Rehabilitation] explode all trees	41493
5	#2 or #3 or #4	43307
6	MeSH descriptor: [Preoperative Care] explode all trees	6162
7	MeSH descriptor: [Preoperative Period] explode all trees	359
8	#6 or #7	6488
9	#5 and #8	333
10	(Prehabilit* or Pre-habilit* or Pre habilitat*):ti,ab,kw	551
11	(Prehabilit* or Pre-habilit* or Pre habilitat*):ti,ab,kw	45
12	#10 or #11	558
13	#1 or #9 or #12 with Publication Year from 2000 to 2022, in Trials	25305
14	(orthop?ed* or arthroplas* or discectom* or diskectom* or laminoplast* or laminec*):ti,ab,kw	28314
15	MeSH descriptor: [Orthopedic Procedures] explode all trees	14436
16	MeSH descriptor: [Arthroplasty] explode all trees	5866
17	MeSH descriptor: [Anterior Cruciate Ligament Reconstruction] explode all trees	520
18	MeSH descriptor: [Femoroacetabular Impingement] explode all trees	91
19	(Femoroacetabular Impingement):ti,ab,kw	93
20	((acl or anterior cruciate ligament*) near (surg* or operat* or proced* or reconstruct*)):ti,ab,kw	6318
21	{or #14-#20}	39429
22	#13 and #21	931

© 2023 Punnoose A et al. *JAMA Network Open*.

eTable 2. Study Characteristics and Summary Results

Author, Year, Location, Type of Surgery, [Risk of Bias]	Number of participants, sex and mean age.	Intervention vs Usual care Mode.	Results: Between group comparison Intervention, Usual care, Mean ± SD (unless reported otherwise)	Adverse events
An,[1]2021, Korea, TKR, [Low]	60<br>20 Intervention 1<br>20 Intervention 2<br>20 Usual care<br>0 Males<br>60 Females<br>70years	Tele-rehabilitation vs education vs usual care; Unimodal	**Pre-op (Intervention 1, Intervention 2, Control)**<br>**WOMAC:** Pain: 11.00±1.14,11.53±2.57,12.67±1.6,Stiffness: 4.38±1.03, 4.38±1.03, 4.82±1.23, 4.56±0.85, Function: 40.67±4.81,40.67±4.81,47.94±4.52, 48.16±6.34;<br>**Muscle strength (ISK):** Quads: 53.071±9.71, 42.77±9.72, 37.94±15.61;<br>**ROM:** Knee flexion:114.41±6.26,110.52±8.84, 107.18±10.23;<br>**TUG (sec):**10.61±1.26,12.16±1.6, 13.34±2.13<br>**6-weeks post-op**<br>**WOMAC:**<br>Pain:3.50±1.33, 6.00±1.00, 7.05±2.55,Stiffness:1.56±0.51, 2.23±0.44, 2.95±0.83, Function: 21.83±4.31,27.88 ±4.47,31.89±6.16;<br>**Ms. strength (ISK):** Quads: 37.34±5.12,31.01±6.88,23.32±5.15;<br>**ROM:** Knee flexion: 138.32±1.79, 131.70±3.18, 127.91±6.73; **TUG (sec):** 9.81±1.06,10.45 ±0.67,11.65±1.85	NR
Beaupre,[2]2004, Canada, TKR, [Some Concerns]	131<br>65 Pre-op exercise and education<br>66 Usual care<br>70 Males<br>61 Females<br>67 years	Pre-op exercise and education vs usual care Unimodal	**Pre-op**<br>**WOMAC**: Pain: 48±13, 49±17; Stiffness: 45±19, 44±18; Function: 50±14, 51±17<br>**Muscle strength affected leg(lb):** Quadriceps: 22±8,24±11, Hamstring: 16±6, 17±7<br>**Knee ROM** (Flex+Ext): 109±12,105±18<br>**SF-36**: Physical Functioning: 32±18, 31±18; Role Physical: 23±33, 16±28; Body Pain: 40±15, 39±14;<br>General Health: 73±18,77±20; Mental Health:73±16, 78±18; Vitality: 49±21, 54±18; Social Functioning: 66±28, 69±23; Role Emotional: 66±42, 74±39<br>**3 months post-op**<br>**WOMAC**: Pain: 74±18,73±14; Stiffness: 62±17,61±18; Function: 73±17,73±15;<br>**Muscle strength affected leg** (lb): Quadriceps: 27±10,27±8; Hamstring: 18±7,18±6<br>**Total Knee ROM (Flex+Ext)**<br>93±16, 93±15<br>**SF-36**: Physical Functioning: 43±20,49±22; Role Physical: 26±35,28±37; Body Pain: 55±21,56±18; General Health: 75±15,75±15; Mental Health: 75±15,75±15; Vitality: 57±19 60±18; Social Functioning: 70±21,75±24; Role Emotional: 70±42,73±39<br>**6 months post-op**	Intervention<br>2 PE<br>3 DVT<br>3 Infection<br>3 MUA<br>Usual care<br>2 PE<br>6 DVT<br>3 Infection<br>2 MUA

© 2023 Punnoose A et al. *JAMA Network Open*.

Author, Year, Location, Type of Surgery, [Risk of Bias]	Number of participants, sex and mean age.	Intervention vs Usual care Mode.	Results: Between group comparison Intervention, Usual care, Mean ± SD (unless reported otherwise)	Adverse events
			**WOMAC:** Pain: 80±15,75±15; Stiffness: 82±13,80±16; Function: 78±15,74±15<br>**Muscle strength affected leg (lb):**<br>Quadriceps: 29±9,28±9; Hamstring: 19±6,20±7<br>**Total Knee ROM (Flex+Ext):**<br>95±14,96±17<br>**SF-36:** Physical Functioning: 52±18,48±24; Role Physical: 34±35,42±40; Body Pain: 59±20,57±17; General Health: 74±14,75±18; Mental Health: 80±13,80±18; Vitality: 59±18,63±19; Social Functioning: 80±21,76±24 ; Role Emotional: 68±39,79±38<br>**1 year post-op**<br>**WOMAC:** Pain: 82±13,80±16;<br>Stiffness: 67±18,71±21; Function: 77±14,77±16<br>**Muscle strength affected leg (lb):**<br>Quadriceps: 30±10,29±8; Hamstring: 21±8,21±6<br>**Total Knee ROM (Flex+Ext)**<br>99±16,103±16<br>**SF-36:** Physical Functioning: 53±22,58±25; Role Physical: 44±38,56±42; Body Pain: 69±22,70±22; General Health: 73±16,76±18; Mental Health: 81±15,85±12; Vitality: 57±20,65±19; Social Functioning: 84±22,85±0; Role Emotional: 69±38,87±26<br>**Total LOS(days):**<br>10.2±4.5,11.7±5.2<br>**Healthcare costs:**<br>$1369±274, $1366±1415; **Readmission rate:** 11 in 1 year- 3 for MUA due to poor ROM (2 usual care and 1 intervention)	
Berge,[3]2004, UK, THR, [High]	40<br>19 Intervention<br>21 Usual care<br>12 Males<br>28 Females<br>71.3 years	Pain management program and education vs usual care<br>Multimodal	**Pre-op**<br>**Pain:** 4.47±1.97,6.65±2.97;**AIMS(Total)**: Means data unavailable; Walking dist (metres): 194±67,161±98<br>**12 months post-op:**<br>**Pain:** 2.36±3.09,3.20±3.117;<br>**AIMS(Total):**42.89,49.15; Walking dist (metres): 220±91,209±131	NR
Bergin,[4]2014, USA, THR &TKR, [High]	106<br>50 Intervention<br>56 Usual care<br>45 Males<br>61 Females	Incentive spirometry vs usual care<br>Unimodal	Results reported as Mean(95%CI)<br>**Pain at return to baseline IS** volume: TKR: 2.90(1.86,3.95), 3.65(2.44,4.86); THR: 2.44(1.12, 3.76),2.41 (1.25, 3.58) p=0.55<br>**Combined LOS in days** (Mean±SD): 2.5±0.6,2.7±0.6, p=0.122	Intervention<br>1 post-op pulmonary<br>26 non-post-op pulmonary<br>Usual care

© 2023 Punnoose A et al. *JAMA Network Open*.

Author, Year, Location, Type of Surgery, [Risk of Bias]	Number of participants, sex and mean age.	Intervention vs Usual care Mode.	Results: Between group comparison Intervention, Usual care, Mean ± SD (unless reported otherwise)	Adverse events
	63.8 years			3 post-op pulmonary<br>34 non-post-op pulmonary
Brown,[5]2012, USA, TKR; [Some concerns]	32<br>17 Intervention<br>15 Usual care<br>Other data NR	Pre-op exercise vs OA(normal population) vs usual care; Unimodal	Results reported as Mean±SD for OA, Prehab, Usual Care<br>**3 months post-op**<br>**SF-36:** Physical Functioning: 39.14±11.82,66.4±20.7,39.3±30.2; Role Physical: 41.36±11.87, 75.6±19.1,64.3±28.3; Body Pain: 39.67±9.91, 59.9±19.1, 47.5±22.8, General Health: 40.99±11.51, 73.2±21.2,62.4±23.5; Vitality:44.09±10.56,60.2±12.9,59.8±31.0; Social Functioning: 42.78±12.75,84.1±17.7,66.1±37.3; Role Emotional: 43.5±12.85,95.4±10.7,79.8±30.4; Mental Health: 45.46±11.68,81.4±13.1,81.4±20.8	NR
Brown,[6]2014, USA, TKR, [Some Concerns]	31<br>17 Intervention<br>15 Usual care<br>9 Males<br>22 Females<br>63 years	Pre-op exercise vs usual care; Unimodal	Data was extracted from figures.<br>**Pre-op**<br>SEE: 6.7,6.0; OEE:1.6,1.9; 1-week post-op: SEE:6.8,5.9; OEE:NR;<br>**2 weeks post-op**<br>SEE:6.8,6.5; OEE:1.5,1.7; SEE: Non-significant between group difference reported OEE: Non-significant between group difference reported	Intervention 1 infection<br>1 cold<br>3 extended inpatient stay<br>Usual Care<br>2 infection<br>4 extended inpatient stay
Calatayud,[7]2017, Denmark, TKR, [Some Concerns]	44<br>22 Intervention<br>22 Usual Care<br>7 Males<br>37 Females<br>66.7 years	Pre-op exercise vs usual care; Unimodal	Mean(95%CI),[Mean between group difference(95%CI)<br>**Pre-op**<br>**WOMAC:** 40.0(38.6,41.4), 58.6 (57.1,60.1), [18.6 (16.7 to 20.5)];<br>**SF-36(Physical):** 49.0(47.9, 50.1), 40.2(39.1,41.3),[-8.8 (-10.2 to -7.4)]; **WOMAC Pain:** 6.8(6.4,7.2),10.3(9.9 ,10.7),[3.5 (2.9 to 4.0)];<br>**Muscle strength affected leg (kgs):** Knee Flex:17.6(17.1,18.2), 8.2 (7.6,8.8) [-9.4 (-10.1 to -8.7)]; Knee Ext: 37.8(34.7,40.9),22.0(18.8, 25.2), [-15.8 (-19.5 to -12.2)],Hip Abd:13.4(3.0,13.8),7.1 (6.7,7.5), [-6.3 (-6.9 to -5.8)]; **ROM:** Flexion: 114.4 (110.9,117.8), 102.8 (99.3,106.3),Extension: 6.6 (5.6,7.6), 14.9 (13.9,16.0);	0 Intervention<br>3 Usual Care

© 2023 Punnoose A et al. *JAMA Network Open*.

Author, Year, Location, Type of Surgery, [Risk of Bias]	Number of participants, sex and mean age.	Intervention vs Usual care Mode.	Results: Between group comparison Intervention, Usual care, Mean ± SD (unless reported otherwise)	Adverse events
			**TUG(sec):** 6.7(6.4,7.1), 9.0(8.7,9.4), [2.3 (1.8 to 2.7)], **Stair Climbing(sec):**7.2(6.6,7.9),11.4(10.8,12.1),[4.2 (3.4 to 5.0)];<br>**1 -month post-op**<br>**WOMAC:** 28.4(27.0,29.8),42.4(40.9,43.8), [14.0 (12.1 to 15.9)];<br>**SF-36(Physical):**<br>51.4(50.3,52.4), 46.9(45.8, 48.0), [-4.4(-5.8 to -3.0];<br>**WOMAC Pain:**<br>4.0(3.6, 4.4),5.1(4.7,5.5), [1.1 (0.6 to 1.6)]<br>**Muscle strength affected leg (kgs):**<br>Knee Flex: 8.7(8.1,9.3),3.9 (3.3,4.4), [-4.8 (-5.5 to -4.1)];<br>Knee Ext: 8.9 (5.8,12.0),7.7(4.5,10.9), [-1.2 (-4.9 to 2.5)]; Hip Abd: 7.7 (7.3,8.1),4.8(4.3,5.2), [-2.9 (-3.5 to -2.4)]; **ROM:** Flexion: 88.8 (85.4,92.3), 82.3 (78.8,85.8),Extension: 11.1 (10.1,12.2), 16.9 (15.9,17.9); **TUG(sec):** 7.3(6.9,7.6), 9.4(9.0,9.7), [2.1 (1.7 to 2.6)]; **Stair Climbing(sec):** 9.1(8.4,9.7), 12.7(12.1,13.4), [3.6 (2.8 to 4.5)<br>**3 months post-op**<br>**WOMAC:**<br>25.0(23.5, 26.4), 30.7(29.2,32.2), [5.8 (3.9 to 7.6)]; **SF-36(Physical):** 55.7 (54.6,56.8), 53.0(51.9,54.1), [-2.7 (-4.1 to -1.3)]; **WOMAC Pain:** 2.9(2.5,3.3), 3.8(3.4,4.2), [0.9 (0.4 to 1.5)]; **Muscle strength affected leg (kgs):** Knee Flex:9.4(8.8,9.9), 4.4(3.8,5.0), [-5.0 (-5.7 to -4.3)], Knee Ext: 22.8(19.7,25.9),14.3(11.1,17.5), [-8.5 (-12.1 to -4.8)]; Hip Abd: 7.8(7.4,8.2), 5.0(4.5,5.4), [-2.8 (-3.4 to -2.3)]; **ROM:** Flexion: 101.2 (97.8,104.7), 96.4 (92.9,99.9), Extension: 8.2 (7.2,9.3), 13.9 (12.8,14.9); **TUG(sec):** 7.0(6.7,7.3), 8.7 (8.3,9.1),[1.7 (1.3 to 2.1)]; **Stair Climbing(sec):** 7.9 (7.2,8.5),12.1(11.5,12.8), [4.2 (3.4 to 5.1)]; **LOS(days):** Reduction of 1.95 days in the intervention group.	
Cavill,[8]2016, Australia, THR&TKR, [Low]	64<br>32 Intervention<br>32 Usual care<br>31 Males<br>33 Females<br>63years	Pre-op exercise vs usual care; Unimodal	**8 weeks post-op**<br>**THR**<br>**EQ-5D Utility:** 0.69±0.23,0.64±0.23; **EQ-5D VAS:**78.3±19.0,65.9±15.1;<br>**PSFS**: 5.0±2.0,5.5±2.6; **TUG(sec):** 8.9±1.5,17.7±16.9;<br>**N (%) requiring IP Rehab**: 1(11%),3(27%); **LOS(days):** 6.2±1.8,7.2±3.8;<br>**TKR**<br>**EQ-5D Utility:** 0.62±0.27,0.70±0.12, **EQ-5D VAS:** 66.8±22.7,73.2±15.7; **PSFS:** 4.7±2.1,5.5±2.3; **TUG(sec):** 11.8±6.8,10.5±5.2<br>**ROM:** Flex:110.8±10.6, 98.2±11.1, Extension: 12.9±8.5, 13.3±6.4; **N(%) requiring IP Rehab**: 4(21%),1(5%); **LOS(days):** 7.3±2.6,6.7±1.9	NR

© 2023 Punnoose A et al. *JAMA Network Open*.

Author, Year, Location, Type of Surgery, [Risk of Bias]	Number of participants, sex and mean age.	Intervention vs Usual care Mode.	Results: Between group comparison Intervention, Usual care, Mean ± SD (unless reported otherwise)	Adverse events
Crowe,[9]2003, Canada, THR &TKR, [Some Concerns]	133<br>65 Intervention<br>68 Usual care<br>27 Males<br>106 Females<br>68.9years	Pre-op exercise and education and counselling vs usual care; Multimodal	Combined (THR&TKR) results reported<br>**LOS(days)**: 6.55 ± 4.2, 10.50 ± 14.2<br>**Functional Milestones(days)**: Independent bed transfers: 4.71 ± 3.4, 5.87 ± 3.2, Walk 30m: 5.27 ± 5.9, 6.75 ± 7.5, Climb stairs: 5.83 ± 6.6, 6.38 ± 3.4, Achieving required ROM: 5.12±5.4, 6.54±3.5	Intervention<br>3 confused<br>0 hip dislocation<br>2 infection<br>1 cardiac<br>1 other<br>Usual care<br>5 confused<br>2 hip dislocation<br>5 infection<br>3 cardiac<br>1 other
Doiron-Cadrin,[10] 2019,Canada,THR&TKR, [Low]	34<br>12 Intervention 1<br>11 Intevention 2<br>11 Usual care<br>9 Males<br>25 Females<br>65.3 years	In-person Preop ex. vs Tele-Prehab vs usual care; Unimodal	Results reported as Mean±SD for Tele-Prehab, In-person Prehab,usual care<br>**Pre-op**<br>**THR**<br>**LEFS:** 56.5 ± 16.5, 48.8 ± 19.2, 63.8 ± 16.1; **WOMAC:** Pain: 9.0 ± 3.1, 8.0 ± 5.0, 9.0 ± 2.9, **Function**: 29.0 ± 7.3, 16.7 ± 13.4, 31.8 ± 7.9; SF-36: **Physical:** 38.2 ± 6.3,35.5 ± 8.2, 33.9 ± 8.8, **Mental:** 52.8 ± 8.2 42.8 ± 10.7, 35.7 ± 11.6; **TUG(sec):** 7.7 ± 1.1,9.8 ± 3.1, 10.0 ± 2.3;**SPW(sec):** 29.4 ± 5.8, 33.6 ± 7.1, 35.0 ± 6.8; **ST(sec):** 9.6 ± 2.5, 14.4 ± 6.4, 13.2 ± 4.8; **GRCS** (%): 67%, 33%, 0%<br>**TKR**<br>**LEFS:** 69.7 ± 11.0, 52.0 ± 10.3, 50.3 ± 13.8; **WOMAC:** Pain: 6.5 ± 4.7, 7.6 ± 1.5,9.8 ± 4.8; **Function:** 23.5 ± 7.9, 25.8 ± 7.7,37.2 ± 13.6; SF-36: **Physical:** 38.4 ± 5.4, 34.1 ± 5.3,35.4 ± 6.0, **Mental**: 41.3 ± 10.5, 43.0 ± 12.3,33.6 ± 8.1; **TUG(sec):** 9.4 ± 1.3,11.5 ± 2.7,13.2 ± 5.4; **SPW(sec):** 37.2 ± 5.7,39.0 ± 7.3, 47.5 ± 14.1; ST(sec): 15.6 ± 7.6, 23.4 ± 10.9, 28.6 ± 18.5; **GRCS(%):**100%,80%,0%	NR
Dominguez-Navarro,[11]2020, Spain, TKR, [Some Concerns]	82<br>28 Intervention 1<br>28 Intervention 2<br>26 Usual care<br>41 Males<br>41 Females<br>70.4 years	Strength Training vs Strength and Balance training vs usual care; Unimodal	Results reported as Mean±SD for intervention 1, intervention 2, usual care<br>**Pre-op**<br>**Berg Balance:** 49.9±3.3,50.9±2.8,48.3±3.4;<br>**KOOS:** ADL:62.4±10.1,61.1±10.8,51.3±11.7; Symptoms:71±13.7,72±10.6,61.4±13.7, Pain: 61.8±9.9,57.6±11.1,49.3±13, QoL:38.3±14.1,35±15,28.9±13.3;<br>**ROM:**Flex:112.1±11.5,110.9±10.6,104.1±10.5, Ext: -3±3.5,-4.7±4.2,-4.3±5.1<br>**Ms. Strength (N):**Knee ext: 141.9±42, 152.3±40.8,107.2±33.1;<br>**TUG (sec):**13.4±7.6,11.9±2.6,15.7±6.1;	NR

© 2023 Punnoose A et al. *JAMA Network Open*.

Author, Year, Location, Type of Surgery, [Risk of Bias]	Number of participants, sex and mean age.	Intervention vs Usual care Mode.	Results: Between group comparison Intervention, Usual care, Mean ± SD (unless reported otherwise)	Adverse events
			**Functional reach(cm):** 30.8±5.4,30.9±5.1,28±5.8;<br>**Single leg stands (sec):** 7.8±8.7,10.3±9.5,7.5±7.9<br>**2 weeks post-op**<br>**Berg Balance:** 48.4±3.1, 49.4±3.4,47.6±3.3;<br>**KOOS:**<br>ADL:60.11±10.7,64.56±8.9,59.24±14.3,Symptoms:79.9±6.7,82.9±7.3,79.9±6.7,Pain:64±11,65.2±12.5,61.6±14.1,QoL:43.5±12.3,47.2±16.8,40.5±14.1;<br>**ROM:** Flex: 93.3±9.5, 95.2±12.4,93.5±9.4,Ext: -4.7±2.1, -5.8±4.9, -6.6±3.5<br>**Ms Strength (N):** Knee ext: 74.22±24.5,77.39±31.1,71.52±19.4;<br>**TUG(sec):**16.9±8,17±5.8,19.4±7.4;<br>**Functional reach(cm):** 30±4.3,30±5.3,28±5.3;<br>**Single leg stand (s):** NR<br>**6 weeks post-op**<br>**Berg Balance:** 51.4±2.2, 51.7±3.1,50.1±2.9;<br>**KOOS:** ADL:73.3±16.4, 77.6±9, 75.7±9.1,Symptoms: 88.7±7.9, 87.2±7.3,85.9±7, Pain: 79.1±17.5, 74.3±17.5,77.3±12, QoL: 56±8.1, 57.6±14/55.4±17.9;<br>**ROM:** Flex:103.2±10.4, 105.2±9.1,101.6±9.1,Ext:-4.2±4.1,-3.0±4.7,-3.6±2.9;<br>**Ms. Strength (N):** Knee ext: 135.7±53.3,118.7±37.9,118.5±34<br>**TUG(sec):**13.1±6.8,12.4±3.7,13.7±3.8;<br>**Functional reach(cm):** 31.6±4.8, 32.3±5.8,29.9±5.2;<br>**Single leg stands** (s): 9.8±9.7, 8.9±8.7,8±7.8;<br>**1 yr post-op**<br>**Berg Balance:** 52±1.7,52.1±2.1,50.7±2.2;<br>**KOOS:** ADL:88.1±6.8,88.2±5.5,87.8±4.6, Symptoms: 93.4±7.4, 96.7±2.9, 91.4±9.9, Pain:90.2±5.7,92.2±4.4,88.7±7.8; QoL:71.4±8.9,67.9±11.2, 67.6±9.2;<br>**ROM:** Flex:105.1±10.2, 104.8±8.7, 102.9±8.5, Ext:-2.2±2.3,-1.6±2.2,-0.1±3.3;<br>**Muscle Strength (Newton):**<br>Knee ext: 158.3±67.2, 142.8±38.9, 128.3± 32.7;<br>**TUG(sec):** 11.1±3.1, 11.1±2.8, 12.1±2.9;<br>**Functional reach(cm):** 32.7±4.8, 32.3±4.9,29.2±4.6;<br>**Single leg stands (s):** 12.7±10.2, 10.3±9.2,5.1±4.7	
Evgeniadis,[12] 2000, Greece, TKR, [High]	62<br>21 Intervention 1<br>19 Intervention 2<br>22 Usual care	Pre-op exercise vs Post-op exercise	**Pre-op** (Pre-op exercise, postop exercise, usual care)<br>**SF-36:**<br>Physical Functioning: 19.91± 2.77, 17.78±2.99,19.67 ± 3.4,<br>Role Physical: 5.09 ±1.37, 4.84±1.13,4.83 ±1.2,	NR

© 2023 Punnoose A et al. *JAMA Network Open*.

Author, Year, Location, Type of Surgery, [Risk of Bias]	Number of participants, sex and mean age.	Intervention vs Usual care Mode.	Results: Between group comparison Intervention, Usual care, Mean ± SD (unless reported otherwise)	Adverse events
	11 Males<br>42 Females<br>68.6 years	vs usual care; Unimodal	Body Pain: 8.36 ±0.92, 9.3±1.1,8.42 ±1.73<br>General Health: 17.64 ±1.86, 15.67±1.8, 17.5±2.23,<br>Vitality: 14.27± 1.56,15.1±1.45, 14.92 ±3.18<br>Social Functioning: 6.34 ±0.53, 6.2±0.97,6.08 ±0.8<br>Role Emotional: 4.36± 1.43, 4.2±1.09, 4.33 ±1.44<br>Mental Health: 18.55 ±1.69, 17.79±2.21, 19.33 ±2.5<br>Health Change: 3.73± 0.78, 3.22±0.67,3.92± 0.51<br>**ROM:** Flex:112.5±9.11,109.3±12.4, Ext:-2.46±2.46,-3.87±3.93;<br>**ILAS(Total):** 1.9 ±3.8, 0.7±0.65,0.21± 0.89<br>**3 days post-op**<br>**ILAS**(Total): 29.5 ±2.9, 28.2±2.4,28.9 ±3.3<br>**2 weeks post-op**<br>**ROM:**Flex:65.9±6.36,70.25±11.3; Ext:-5.45±3.8, -6.5±3.83; **ILAS(Total)**: 19.7 ±2.45, 20.5±1.2, 20.3 ±1.97<br>**6 weeks post-op**<br>**ILAS(Total):** 9.82 ±0.98, 9.16±0.93,10.08 ±1.16<br>**10 weeks post-op**<br>**ROM:** Flex:73.3±6.87, 76.02±10.3, Ext:-7.45±5.56,-7±3.95<br>**ILAS(Total):** 4.65± 0.58, 2.79±0.64,4.87 ±0.73<br>**14 weeks post-op**<br>**ROM:** Flex:80.73±6.7, 80.42±10.2, Ext: -5.7±4.27, -6.42±3.6<br>**ILAS(Total):** 0.31 ±0.49, 0.14±0.39, 0.38 ±0.56	
Ferrara,[13]2008, Italy, THR, [Some Concerns]	23<br>11 Intervention<br>12 Usual care<br>9 Males<br>14 Females<br>63 years	Pre-op exercise vs usual care; Unimodal	**1 day Pre-op**<br>**Barthel Index:**<br>84.50t±6.7, 75.06±16.2<br>**SF-36:** Physical: 34.4 ±4.05, 27.3±10.3, Mental: 51.1 ±11.2, 40.9 ±11.6<br>**WOMAC** Function: 33.7±13.8,43.5±9.5, Stiffness: 4.82±1.88,4.58 ±1.62, Pain: 8.0±3.8, 11.0±3.6<br>**HHS:** 43.6±15.7, 34.9 ±15.5<br>**Pain:**5.5±2.2, 7.3±2.0<br>**Muscle strength affected leg (BMRC scale):** Quads: 4.50±0.59, 4.25±0.45, Hip Abd: 4.68±0.46,3.90±0.46<br>**ROM:** Abd: 31.8±110.55, 32.08±11.95 External rot: 22.27±7.86,14.58±7.82<br>**2 weeks post-op**<br>**Pain on VAS (Median)**	NR

© 2023 Punnoose A et al. *JAMA Network Open*.

Author, Year, Location, Type of Surgery, [Risk of Bias]	Number of participants, sex and mean age.	Intervention vs Usual care Mode.	Results: Between group comparison Intervention, Usual care, Mean ± SD (unless reported otherwise)	Adverse events
			Data extracted from figures: 3.75 (IQR=4), 5.0 (IQR=1).<br>**4 weeks post-op**<br>**Pain on VAS-(Median)**<br>Data extracted from figures: 0.5 (IQR=2), 3.5(IQR=1.5).<br>**12 weeks post-op**<br>**Barthel Index:**<br>95.0±4.08, 91.8±2.52<br>SF-36 Physical: 46.6 ±8.95, 52.09±8.11; Mental: 53.10±6.65,51.36±9.06;<br>**WOMAC** Function**:**18.3±12.06, 28.5±10.01 Stiffness: 1.00±1.33, 1.3±1.56, Pain: 1.7±2.35, 2.2±1.75; HHS: 69.47±7.49, 65.2±16.4;<br>**Pain:** 0.3±0.48, 1.27 ±1.00<br>**Muscle strength affected leg (BMRC scale):** Quads: 5.0±0.0, 5.0±0.0,Hip Abd: 5.0±0.0, 5.0±0.0<br>**ROM:** Abd: 43.00±6.32, 39.09±4.36, External rot: 33.50±4.11, 33.64±4.52	
Franz,[14]2022, Germany, TKR, [High]	30<br>10 Intervention<br>10 Intervention<br>10 Usual care<br>20 Males<br>10 Females<br>64yrs	Prehabiliation and BFR (Intervention 1) vs Prehabilitation and Sham BFR (Intervention 2) vs usual care	**Results reported as Means ± SD for Intervention1, Intervention 2, Usual care**<br><br>**Pre-op (5 days prior to op)**<br>**Muscle strength(kg):** Quads: 31.5±10.4, 16.0±10.2, 20.3 ±7.1, Hams: 19.1±4.8, 9.8±3.6, 11.0±2.9; **KOOS:** Symptoms: 60.8 ± 3.7, 48.6 ± 9.5, 51.7 ± 4.7, Pain: 57.6 ± 3.4, 49.8 ± 5.2, 47.6 ± 5.4, ADL: 57.9 ± 3.7, 52.8 ± 3.4, 49.4 ± 6.0, Sports & Rec: 29.5 ± 3.7, 24.0 ± 3.9, 25.0 ± 4.1, QOL: 40.0 ± 3.2, 30.9 ± 5.2, 25.6 ± 10.0; **6MWT:** 456 ± 58, 450±70, 390±80; **CRT:** 12±3, 12.00 ± 2.49, 10.5±3.5; **ROM:** Flex: 116.0±10.7, 112.4 ±3.1, 117.0±14.4, Ext: 2.70±2.41, 2.30 ±2.83, 2.60±1.90<br>**3 months post-op**<br>**Muscle Strength(kg):** Quads: 22.1±10.4, 11.1±9.5, 15.0±6.3, Hams: 14.6±5.3, 6.4 ±4.6, 8.8 ±2.7; **KOOS:** Symptoms: 47.2±3.0, 46.1 ± 7.7, 45.0 ± 4.5, Pain: 67.8 ± 3.5, 61.7 ± 6.8, 60.8 ± 6.9, ADL: 63.7 ± 5.1, 59.0 ± 3.5, 58.7 ± 4.5, Sports & Rec: 31.0 ± 5.7, 25.0 ±4.1, 23.1±3.5,QOL: 52.5 ± 6.8, 44.4 ± 4.6, 45.6 ± 9.2; **6MWT:** 426 ± 73, 420±60, 370±70; **CRT:**10.90±2.77, 10.0±2.98, 10.25±3.75; **ROM:** Flex: 117.9 ±6.0, 113.3 ±10.1,114.7±7.1<br>Ext: 1.50 ±3.24, 2.00±1.63, 3.30 ±2.98<br>**6 months post-op**<br>**Muscle Strength(kg):** Quads: 30.8±11.1, 15.3 ±9.4,18.5 ±7.0, Hams:19.6±4.1, 9.0 ±4.3, 11.0±1.7; **KOOS:** Symptoms: 67.1 ± 3.6,65.2 ± 9.0,63.4 ± 5.1, Pain: 70.0 ± 4.7,76.2 ± 3.6,71.1 ± 7.9,ADL: 71.9 ±3.1,67.8 ± 3.1,65.6 ± 4.6, Sports & Rec: 38.5	None

© 2023 Punnoose A et al. *JAMA Network Open*.

Author, Year, Location, Type of Surgery, [Risk of Bias]	Number of participants, sex and mean age.	Intervention vs Usual care Mode.	Results: Between group comparison Intervention, Usual care, Mean ± SD (unless reported otherwise)	Adverse events
			± 3.4,29.5 ± 6.0,32.0 ± 6.7, QOL: 55.0 ±4.0,44.4 ± 4.6,54.4 ± 8.99; **6MWT:** 464 ± 58, 445±90, 430±70; **CRT:**13.3±2.31, 11.30±2.5, 11.75±3.25; **ROM:** Flex: 119.5±6.6,115.3 7.9, 122.0±8.5,Ext: 0.70±1.57, 1.20 ±1.14/ 2.00±2.58	
Gocen,[15]2004, Turkey, THR, [High]	60 Intervention<br>30 Control<br>31 Males<br>28 Females<br>51 years	Pre-op exercise and education vs usual care<br>Unimodal	**1-day Pre-op**<br>**HHS:** 51.48 ±18.32, 45.30 ±12.98<br>**At Discharge**<br>**HHS:** 64.46±6.92, 59.36±6.82<br>**Pain(at rest):** 0.28±0.59, 0.40±0.93<br>**Pain(during activity):** 1.24±1.60, 1.30±1.73<br>**Functional Milestones(days):**<br>Walking: 2.07±0.20,2.20±0.41, Climbing stairs: 6.17 ±1.69, 7.37 ±1.02, Bed transfer: 2.93 ±0.59, 3.33±0.71, Toilet transfer: 4.24±0.51, 5.07±1 .28<br>**12 weeks post-op**<br>**HHS:** 85.30±11.78, 78.70±9.41<br>**2 years post-op**<br>**HHS**: 97.14±4.32, 95.66 ±6.08	Intervention<br>1 infection<br>Usual care<br>1 infection
Grant,[16]2017, UK, Hip Arthroscopy, [High]	18<br>9 Intervention<br>9 Usual care<br>6 Males<br>12 Females<br>39.5 years	Pre-op ex vs usual care, Unimodal	Results reported as Median (IQR)<br>**2 weeks Pre-op**<br>**Muscle Strength affected leg (lbs):**Hip Abd: 20.9 (15.6 to 31.3), 13.6 (8.5 to 14.7),Hip Add: 19.3 (15.9 to 29.3),12.4 (8.2 to 14.8), Hip Flex: 32.9 (28.8 to 51.2), 18.9 (10.5 to 27.4), Hip Ext Rot: 19.0 (13.7 to 22.3), 13.9 (9.3 to 15.0), Knee Ext: 49.0 (34.2 to 88.5), 25.1 (18.4 to 36.4);<br>**NAHS:** 56.3 (51.3 to 76.3), 48.8 (36.6 to 64.4);<br>**EQ-5D-5L:** 75.0 (71.3 to 80.0), 55.0 (42.5 to 67.5);<br>**12 weeks post-op**<br>**Muscle Strength affected leg (lbs)** Hip Abd: 20.9 (18.3 to 31.3), 18.8 (14.7 to 23.3) Hip Add: 18.8 (16.4 to 28.3), 17.2 (13.7 to 20.3), Hip Flex: 36.1 (22.0 to 51.6), 25.5 (20.3 to 34.5), Hip Ext Rot: 18.4 (14.3 to 31.5), 15.6 (15.2 to 20.0), Knee Ext: 56.4(39.5 to 104.5),38.7(30.8 to 63.3);<br>**NAHS:** 91.3 (78.8 to 94.7), 85.0 (79.7 to 88.8)<br>**EQ-5D-5L:** 85.0 (81.3 to 89.5), 82.5 (75.0 to 85.0)	NR

© 2023 Punnoose A et al. *JAMA Network Open*.

Author, Year, Location, Type of Surgery, [Risk of Bias]	Number of participants, sex and mean age.	Intervention vs Usual care Mode.	Results: Between group comparison Intervention, Usual care, Mean ± SD (unless reported otherwise)	Adverse events
Gstoettner,[17]<br>2011, Austria, TKR, [High]	38<br>18 Intervention<br>20 Usual care<br>8 Males<br>30 Females<br>72.8 years	Pre-op exercise vs usual care Unimodal	**1-day Pre-op (Follow-up for Intervention Group only)**<br>**Gait speed(sec):** 60m:49.4±13.6, NR, Stairs up: 26.6±8.8, NR, Stair down: 22.7 ±8.2, NR;<br>**WOMAC:** Pain: 3.1 ±1.3,NR, Stiffness:1.7±1.4,NR, Function: 2.2 ±1.2,NR;<br>**KSS (Total):** 74.5±15.2, NR; **KSS(Function):** 80.3 ±18.1, NR; **Stability Index:**MLSI: 1.6±0.5, NR, APSI: 1.9 ±0.6, NR, OSI: 2.3±0.7, NR;<br>**6 weeks Post-op**<br>Gait Speed(sec):60m: 56.8 ±17.7, 51.8±9.9 Stairs up: 33.8 ±13.8, 30.0±8.4, Stair down: 34.6 ±17.4, 30.4±10.2; **WOMAC:** Pain: 1.3±1.1, 0.98±0.99 Stiffness:1.5±1.5, 1.1±1.5, Function: 1.2±1.2, 1.9±1.0; **KSS (Total):** 82.5±19.2, 80.6±17.5, p=0.831; **KSS(Function):** 74.3±14.6, 73.9±5.9<br>**Stability Index:** MLSI: 1.6±0.7, 1.9±0.6, APSI: 1.7 ±0.4, 2.3 ±0.6, OSI: 2.2 ±0.7, 2.9 ±0.7	0 Intervention<br>Usual care<br>1 DVT
Hermann,[18]<br>2015, Denmark, THR, [High]	80<br>40 Intervention<br>40 Usual care<br>28 Males<br>52 Females<br>70.4years	Pre-op exercise vs usual care Unimodal	Results reported as Mean±SD, Effect Size [95% CI]<br>**Pre-op**<br>**HOOS ADL**: 59.9±17.1, 48.7±13.9, 0.8[0.3,1.3], **Pain:** 55.4±16.9, 45.9±14.1,0.6[0.2,1.1], **Symptoms:** 56.6±19.8, 45.4±16.7,0.6 [0.2,1.1],<br>**Sports& Recreation:** 38.5±18.9, 28.6±15.4,0.6 [0.2,1.1], **Hip related QOL:** 38.8±17.2, 31.2±13.9,0.4 [0.0,0.8];<br>**Muscle strength affected leg(Watt/kg):**<br>Hip Ext: 1.9±0.7, 1.4±0.7, 0.6[0.2,1.1],<br>**Muscle strength unaffected leg(Watt/kg):**<br>Hip Ext: 2.2±0.8,1.7±0.8, 0.5[0.1,1.0]<br>**Results reported as Means between group difference (95%CI) p-value**<br>**3 months post-op**<br>**HOOS:** ADL: 5.6 (-1.1,12.3) 0.106, Pain: 2.3 (-4.6,9.3) 0.51<br>Symptoms: 4.1 (-3.4,11.6) 0.28<br>Sports& Recreation: 10.5 (1.4,19.6) 0.023, Hip related QOL: 3.4 (-5.4,12.2) 0.44;<br>**Functional performance:**<br>Stair ascending (steps/sec): 1.2 (0.2,2.2) 0.021, Stair descending (steps/sec): 1.0 (-0.2,2.3) 0.114, Chair rise (s): 2.6 (1.0,4.3) 0.002, Gait 25m, normal speed(m/s): 1.4 (-0.02,2.8) 0.054, Gait 25m, max speed(m/s): 1.5 (0.2,2.7) 0.020;<br>**Muscle strength (Nm/kg)** Knee Ext affected: 0.19 (0.08,0.30) 0.001, Knee Ext unaffected: 0.25 (0.12,0.37) <0.001, Hip Ext affected: 0.13 (-0.04,0.29) 0.141, Hip Ext unaffected: 0.13 (-0.04,0.29) 0.141<br>**6 months post-op**	None

© 2023 Punnoose A et al. *JAMA Network Open*.

Author, Year, Location, Type of Surgery, [Risk of Bias]	Number of participants, sex and mean age.	Intervention vs Usual care Mode.	Results: Between group comparison Intervention, Usual care, Mean ± SD (unless reported otherwise)	Adverse events
			**HOOS:** ADL: 3.9 (-3.2, 11) 0.28, Pain: 5.1 (-2.2,12.4) 0.174,Symptoms: 1.0 (-6.9,8.9) 0.81Sports& Recreation: 2.3 (-7.2, 11.8) 0.64, Hip related QOL: 5.8 (-3.4,15.1) 0.22;<br>**9 months post-op**<br>**HOOS:** ADL: 1.8 (-5.7,9.3) 0.64, Pain: 3.5 (-4.2,11.2) 0.37<br>Symptoms: _1.2 (-9.5,7.1) 0.77<br>Sports& Recreation: 1.7 (-8.2,11.7) 0.74, Hip related QOL: 3.1 (-6.6,12.8) 0.53<br>**12 months post-op**<br>**HOOS:** ADL: 2.6 (-4.2,9.8) 0.44, Pain: 0.5 (-6.7,7.7) 0.89<br>Symptoms: _4.9 (-12.7,2.8) 0.21,Sports& Recreation: 6.2 (-3.2,15.6) 0.20, Hip related QOL: 0.2 (-8.9,9.3) 0.97;<br>**Functional performance:**<br>Stair ascending (steps/sec): 1.3 (0.3,2.3) 0.011<br>Stair descending (steps/sec): 1.6 (0.3,2.9) 0.017, Chair rise (s): 0.7 (-1.0,4.3) 0.41, Gait 25m, normal speed(m/s): 1.4 (-0.07,2.8) 0.062, Gait 25m, max speed(m/s): 0.9 (-0.4,2.2) 0.17;<br>**Muscle strength (Nm/kg)**<br>Knee Ext affected: 0.10 (-0.02,0.22) 0.088, Knee Ext unaffected: 0.09 (-0.05,0.22) 0.23, Hip Ext affected: 0.15 (-0.03,0.34) 0.104, Hip Ext unaffected: 0.16 (-0.00,0.31) 0.054	
Hoogeboom,[19] 2010, Netherlands, THR, [Some Concerns]	21<br>10 Intervention<br>11 Usual care<br>7 Males<br>14 Females<br>76 years	Pre-op exercise vs usual care; Unimodal	Results reported as Means± SD,[Adjusted Mean difference between groups (95% CI)]<br>**Pre-op**<br>**HOOS:** Pain: 55.3±12.0, 49.3±17.0,[7.3 (-5.1,19.7)], Symptoms: 56.5±14.2, 59.0±15.6,[-0.6 (-12.2,11.0)],<br>ADL: 51.1±10.5, 52.3±21.2,[-0.6 (-11.9,10.6)], Sports &Rec: 25.0±14.4,32.5±20.2,[4.6 (-14.0, 4.9)], Quality of Life: 36.3±15.8,43.3±15.4,[-1.2 (-13.0,10.7)];<br>**Pain:** 37.7±18.0,49.3±24.7[-8.6 (-27.3,10.2)];<br>**Functional performance**:<br>6MWT(metres):<br>363.0±126.3,342.7±133.7 [5.3 (-43.7,54.3)], TUG(sec): 9.6±2.8,13.4±11.5,[-4.4 (-9.3,0.5)], CRT(sec): 15.0±5.8,17.4±5.9,[-2.9 (-6.2,0.4)], PWC-170: 103.6±74.5,120.7±63.3[-17.9 (-92.6,56.9)], Hand Grip Strength(N):243.7±92.2,283.5±83.3[-12.7 (-47.3,21.9)];	Intervention<br>1 Fractured femur peri-op<br>1 Low saturation<br>0 Usual care

© 2023 Punnoose A et al. *JAMA Network Open*.

Author, Year, Location, Type of Surgery, [Risk of Bias]	Number of participants, sex and mean age.	Intervention vs Usual care Mode.	Results: Between group comparison Intervention, Usual care, Mean ± SD (unless reported otherwise)	Adverse events
			**Post-op LOS(days)**<br>Median 6 days both groups	
Huang,[20] 2012, Taiwan, TKR, [High]	243<br>126 Intervention<br>117 Usual care<br>73 Males<br>170 Females<br>70 years	Pre-op home exercise education vs usual care; Unimodal	**Pre-op**<br>**Pain using VAS:** 6.4±1.0, 6.5±1.0;<br>**ROM:**Flex:92±7,91±7, Ext: NR<br>**Day 1 post-op**<br>**Pain:**<br>4.5±1.3,4.4. ±1.2,<br>**ROM:** Flex:30±11, 30±12 Ext: NR<br>**Day of Discharge(Day5):**<br>Pain: 2.4±0.7,2.5±0.6<br>ROM: Flex:76±22, 74±20 Ext: NR<br>**Functional recovery (ambulation%):** 85.7%,81.2%<br>**LOS(days)** 7± 1, 8± 1(95%CI:-0.795 to-0.044; **Medical expenditure** 123726±5204,125838±4428)	Intervention<br>1.6% Infection<br>4% DVT<br>Usual care<br>0.9% Infection<br>2.6% DVT
Huber,[21] 2015, Switzerland, TKR, [High]	45<br>22 Intervention<br>23 Usual care<br>24 Males<br>21 Females<br>69.8 years	Pre-op exercise+ education vs education only	Results reported as Mean between group difference(95%CI)<br>Intervention, usual care<br>**1-week Pre-op**<br>**Chair Stand Test(sec)**: -1.5 (-5.3, 2.2);<br>**KOOS**:ADL: 1.3 (-10.1, 12.8), Pain: -2.3 (-12.4, 7.9);<br>**Muscle strength-affected leg(n):** Knee ext: 8.8 (-40.0, 57.8); Knee flex: 23.2 (-0.1, 46.5);<br>**20m Walk Test(sec):** -0.6 (-2.0, 0.9); **TUG(sec):** 0.2 (-1.5, 2.0); SF-36: Physical Functioning: 7.1 (-5.3, 19.5), Role Physical: -10.8 (-39.9, 18.3), Body Pain: 4.9 (-7.2, 17.0), General Health: 3.3 (-5.9, 12.4), Mental Health: 2.6 (-6.6, 11.7), Vitality: -2.3 (-13.9, 9.3), Social Functioning: 5.0 (-7.1, 17.2), Role Emotional: -10.8 (-34.3, 12.7);<br>**EQ-5D:** Mobility: 0.1 (-0.3, 0.4)<br>Self-Care: -0.0 (-0.2, 2.2)<br>Usual activities: -0.0 (-0.4, 0.4)<br>Pain/Discomfort: -0.1 (-0.4, 0.2), Anxiety/Depression: -0.1 (-0.3, 0.3); EQ-VAS: 7.1 (-2.5, 16.7);<br>**ROM:** Flex: 1.9 (−4.4, 8.2); Ext: 0.8 (−2.4, 4.0)<br>**6 weeks post-op**<br>**Chair Stand Test(sec):** Not assessed; **KOOS:** ADL: -2.0 (-13.3, 9.3), Pain: -6.4 (-16.5, 3.6); **Muscle strength(n):**	Intervention<br>1 pain<br>0 Usual care

© 2023 Punnoose A et al. *JAMA Network Open*.

Author, Year, Location, Type of Surgery, [Risk of Bias]	Number of participants, sex and mean age.	Intervention vs Usual care Mode.	Results: Between group comparison Intervention, Usual care, Mean ± SD (unless reported otherwise)	Adverse events
			Knee ext: NR Knee flex: NR, **20m Walk Test(sec):**Not assessed<br>**TUG(sec**): Not assessed;<br>**SF-36:**Physical Functioning: 0.5 (-11.8, 12.7), Role Physical: -5.2 (-34.2, 23.9), Body Pain: 2.8 (-9.2, 14.9), General Health: 3.4 (-5.7, 12.5), Mental Health: -1.6 (-10.7, 7.5), Vitality: -1.0 (-12.5, 10.5), Social Functioning: 2.4 (-9.7, 14.4) , Role Emotional: -11.1 (-34.5, 12.3)<br>**EQ-5D:** Mobility: 0.0 (-0.3, 0.4), Self-Care: 0.1 (-0.2, 0.3),<br>Usual activities: -0.1 (-0.5, 0.2), Pain/Discomfort: 0.1 (-0.3, 0.4),<br>Anxiety/Depression: 0.0 (-0.2, 0.2), EQ-VAS: 2.1 (-7.4, 11.6);<br>**ROM:** Flex: NR, Ext: NR<br>**3 months post-op**<br>**Chair Stand Test(sec)**: 2.0 (-1.8, 5.8); **KOOS:** ADL: -4.9 (-16.3, 6.5), Pain: -3.3 (-13.5, 6.8); **Muscle strength (n):** Knee Ext: -3.5 (-52.7, 45.6); Knee Flex: -12.7 (-36.2, 10.8);<br>**20m Walk Test(sec)**: -0.5 (-2.0, 1.0)<br>**TUG(sec):** 1.6 (-0.1, 3.3)<br>**SF-36:** Physical Functioning: -6.6 (-8.5, 17.5), Role Physical: -3.2 (-32.2, 25.9), Body Pain: -3.4 (-15.5, 8.7), General Health: -2.8 (-12.0, 6.3)Mental Health: -3.0 (-12.2, 6.1); Vitality: -8.3 (-20.0, 3.3)<br>Social Functioning: -1.6 (-13.7, 10.5), Role Emotional: -10.2 (-34.0, 13.5);<br>**EQ-5D:** Mobility: -0.1 (-0.4, 0.2); Self-Care: 0.1 (-0.1, 0.3); Usual activities: 0.1 (-0.1, 0.3); Pain/Discomfort: -0.0 (-0.5, 0.3), Anxiety/Depression: -0.0 (-0.3, 0.3), EQ-VAS: 1.2 (-8.4, 10.8); **ROM:**Flex: −3.9 (−10.2, 2.4), Ext: 1.4 (−1.8, 4.5)<br>**3-12 months post-op**<br>Chair Stand Test(sec): Not assessed **KOOS**:ADL: 3.6 (-8.5, 15.7), Pain: 2.3 (-8.5, 13.0); **Muscle strength (n):** Knee Ext: NR, Knee Flexion: NR; **20m Walk Test(sec):** Not assessed<br>**TUG(sec):** Not assessed<br>**SF-36:** Physical Functioning: 4.5 (-8.5, 17.5), Role Physical: -1.1 (-31.7, 29.5), Body Pain: 4.9 (-7.8, 17.7), General Health: 2.5 (-7.2, 12.1)<br>Mental Health: 0.4 (-9.2, 10.0), Vitality: 3.3 (-8.9, 15.5),<br>Social Functioning: -0.7 (-13.4, 12.1), Role Emotional: -9.3 (-34.2,15.7),<br>**EQ-5D:** Mobility: 0.1 (-0.3, 0.4); Self-Care: 0.0 (-0.2, 0.2), Usual activities: -0.1 (-0.4, 0.3), Pain/Discomfort: -0.1 (-0.4, 0.3), Anxiety/Depression: 0.1 (-0.2, 0.3), EQ-VAS: -0.1 (-10.2, 10.0);<br>**ROM:** Flex: NR,Ext: NR	

© 2023 Punnoose A et al. *JAMA Network Open*.

Author, Year, Location, Type of Surgery, [Risk of Bias]	Number of participants, sex and mean age.	Intervention vs Usual care Mode.	Results: Between group comparison Intervention, Usual care, Mean ± SD (unless reported otherwise)	Adverse events
Jahic,[22]2018, Bosnia and Herzegovina, TKR, [Some Concerns]	20<br>10 Intervention<br>10 Usual care<br>6 Males<br>14 Females<br>59 years	Pre-op exercise vs usual care; Unimodal	**Pre-op**<br>**KSS:** 46.4±8.00, 35.70±5.58; **FS:** 40.50±7.25, 29.50±7.25;<br>**After Surgery**<br>**KSS:**66.70±6.83,47.90±6.89,; **FS:** 42.50±7.91, 39.50±7.25,<br>**3 months post-op**<br>**KSS:** 76.70±6.83, 57.90±7.05; **FS**: 68.00±2.58, 67.50±2.64<br>**6 months post-op**<br>KSS: 79.10±6.97, 57.90±7.05,; **FS:**77.50±2.64, 77.50±2.64,<br>**1-year post-op**<br>**KSS:** 80.20±7.07, 75.30±4.88; **FS:** 92.00±4.22, 90.00±0.00	NR
Kim,[23]2021, USA, TKR, [Some concerns]	43<br>20 Intervention<br>23 Usual care<br>14 Males<br>19 Females<br>67.1yrs	Aquatic therapy vs usual care Unimodal	**Pre-op**<br>**WOMAC:** Pain: 7.3 ± 0.5, 9.3 ± 0.4, Stiffness: 3.3 ± 0.3, 4.5 ± 0.2, Function: 27.6 ± 1.6, 27.6 ± 1.6; **SPPB:** Balance: 3.6 ± 0.2, 3.6 ± 0.2, Gait speed: 3.4 ± 0.2, 3.1 ± 0.2, Repeated chair stand: 2.6 ± 0.2, 1.9 ± 0.2; **Depression:** 1.7 ± 0.4, 2.9 ± 0.4<br>**4-6 weeks Post-op**<br>**WOMAC:** Pain: 6.4 ± 0.7, 8.2 ± 0.7, Stiffness: 3.5 ± 0.2, 4.0 ± 0.2, Function: 18.7 ± 2.7, 26.8 ± 2.7<br>**SPPB:** Balance: 3.8 ± 0.2, 3.6 ± 0.2, Gait speed: 2.8 ± 0.2, 2.6 ± 0.2, Repeated chair stands: 1.9 ± 0.3, 1.2 ± 0.3<br>**Depression:** 2.3 ± 0.5, 2.8 ± 0.5	Odd ratio1.5 (95%CI 0.21-11.2)<br>Further data unavailable
Leeuwen,[24]2014, Netherlands, TKR, [High]	22<br>11 Intervention<br>11 Usual care<br>12 Males<br>10 Females<br>69-73years	Pre-op exercise and education vs usual care Unimodal	**Pre-op**<br>**WOMAC**:65±20,67±8<br>**Ms. Strength affected side**<br>**MVT ext:**111±50,121±50; **Doublet Torque:** 50±16,48±17; **Voluntary activation(%):** 78±15,85±8; **MVT Flexion**: 43±19,50±24; **Ms. Strength unaffected side MVT- Ext**:123 ± 47, 139 ± 57, **Doublet Torque**: 52 ± 14, 50 ± 16, **Voluntary activation(%):** 78 ± 15, 85 ± 10, **MVT Flex**: 47 ± 26, 55 ± 30;<br>**Functional Tasks: Chair Stand Test (sec):** 11.3±2.1,11.4±1.8, **Stair Climb Test(sec):**11.6±3.4,12.4±3.3 **6MWT(metres):**471±92,493±55<br>**6 weeks post-op**<br>**WOMAC**: 70±16,79±11	NR

© 2023 Punnoose A et al. *JAMA Network Open*.

Author, Year, Location, Type of Surgery, [Risk of Bias]	Number of participants, sex and mean age.	Intervention vs Usual care Mode.	Results: Between group comparison Intervention, Usual care, Mean ± SD (unless reported otherwise)	Adverse events
			**Ms. Strength affected side MVT Ext**: 63±30,70±35 **Doublet Torque:** 34±10,35±13 **Voluntary activation (%):** 79±9,84±4, **MVT Flexion:** 37±18,36±16<br>**Ms. Strength unaffected side MVT Ext**: 116 ± 44, 128 ± 65<br>**Doublet Torque**: 50 ± 14, 50 ± 17, **Voluntary activation(%):**80 ± 13, 88 ± 6, **MVT Flex**: 47 ± 27, 55 ± 30<br>**Functional Tasks:**<br>**Chair Stand Test (sec):**13.3±3.4,12.5±2.5, **Stair Climb Test (sec):** 20.9±10.8, 17.6±7.5, **6MWT (metres):** 380±109,440±87<br>**12 weeks post-op**<br>**WOMAC:** 83±15,93±4<br>**Ms. Strength affected side MVT Ext**:76±34,97±40<br>**DoubletTorque**:39±12,39±14; **Voluntary activation(%):** 80±10,90±8, **MVT Flex**:42±17,50±23, **Ms. Strength unaffected side MVT Ext:**118 ± 43, 138 ± 56<br>**Doublet Torque**: 51 ± 16, 50 ± 13, **Voluntary activation(%):** 83 ± 11, 91 ± 6; **MVT Flexion:** 47 ± 26,55 ± 26; **Functional Tasks**: **Chair Stand Test (sec)**:11.8±1.8,10.8±1.5, Stair Climb Test(sec): 12.8±3.4,14.1±0, **6MWT(metres):** 456±62, 513±97	
Lindback,[25]2017<br>,<br>Sweden,<br>Lumbar surgery,<br>[Some Concerns]	197<br>98 Intervention<br>99 Usual care<br>92 Males<br>105 Females<br>60 years	Pre-op exercise+ CBT vs usual care; Multimodal	Reported as Mean Change between groups Intervention, usual care (95%CI)<br>**Pre-op**<br>**ODI:** -3.3 (-6.2,-0.4)<br>**Pain:** Back Pain: -6.0 (-11.8,-0.3), Leg Pain: -4.6 (-10.8,1.5)<br>**EQ5D:** 0.078 (0.0,0.1)<br>EQVAS: 4.6 (0.4,8.8)<br>**SF-36 Physical**: -1.5 (-0.5,3.5); **SF-36 Mental**: 0.3 (-1.7,2.4);<br>**SEE:** 8.7 (2.2,15.2)<br>**Gait Speed(m/sec):**<br>Normal: 0.07 (0.02,0.12); Fast: 0.21(0.07,0.26)<br>**Muscle Strength (kg):** Quads(Rt): 2.80 (0.74,4.87) Quads(Lt): 3.28 (1.30 to 5.20)<br>Results below reported as n(%): **ODI (Item4):**<br>Pain does not prevent walking:13(20%), 8(10%),<br>Walking distance 1km: 32(47%), 27(36%), Walking distance 500m: 13(20%), 21(28%), Walking distance 100m:9(13%),11(15%)<br>In need of crutches:0(0%), 8(11%), Mostly bedridden: 0(0%),0(0%)	NR

© 2023 Punnoose A et al. *JAMA Network Open*.

Author, Year, Location, Type of Surgery, [Risk of Bias]	Number of participants, sex and mean age.	Intervention vs Usual care Mode.	Results: Between group comparison Intervention, Usual care, Mean ± SD (unless reported otherwise)	Adverse events
			Self-rated walking distance:<br><100m: 4(5%),7(9%), 100-500m:25(35%),32(41%)<br>0.5-1km:11(16%),22(29%)<br>>1 km:31(44%),16(21%)<br>**3 months Post-op**<br>ODI: 1.6 (-2.9,6.1)<br>EQ5D:0.004 (-0.1,0.1)<br>EQVAS: 0.8 (-4.5,6.1)<br>SES:-6.0 (-15.1,3.2)<br>**1 -year post-op**<br>**ODI:** 4.0 (-0.5,8.5)<br>**Pain:** Back Pain: 4.7 (-2.4,11.8), Leg Pain: 2.9 (-5.4,11.2)<br>**EQ5D:** -0.030 (-0.1,0.0) EQVAS: -4.5 (-10.3,1.2), **SF-36 Physical:** -1.4 (-4.8,1.9), **SF-36 Mental**: -1.6 (-4.0,0.8), **SES:** -6.9 (-16.2,2.3)<br>**PGIC (UC usual care, IG Intervention group):**<br>Worse- 2% UC, 13% IG<br>No Change-41% UC, 38% IG<br>Improved: 17% UC, 49% IG	
Lotzke,[26]2019, Sweden, Lumbar Surgery, [Low]	118<br>59 Intervention<br>59 Usual care<br>55 Males<br>63 Females<br>45 years	CBT vs usual care<br>Unimodal	Between group mean difference not reported<br>Results reported as between group Effect Size (95%CI)<br>**Pre-op**<br>**ODI:** 0.13 (-0.26,0.51); **Pain:** Back Pain: 0.21(-0.18,0.59), Leg Pain: -0.09(-0.47,0.30); **PCS**: 0.29(-0.11,0.69); **SEE:** 0.04(-0.35,0.44); **EQ-5D**: 0.57(0.16,0.98); **EQVAS:** 0.02(-0.37,0.41); **PSFS**: 0.23(-0.19,0.65)<br>**3 weeks post-op**<br>**ODI:** -0.08(-0.47,0.31)<br>**Pain:** Back Pain: -0.22(-0.62,0.17), Leg Pain: 0.00(-0.39,0.40); **PCS:** -0.03(-0.43,0.36); **SEE:** 0.25(-0.16,0.65); **EQ-5D:** -0.18(-0.57,0.22); EQVAS: -0.18(-0.58,0.22); **PSFS**:0.15(-0.27,0.57)<br>**8 weeks post-op**<br>**ODI:** 0.12(-0.27,0.50); **Pain:** Back Pain: 0.24(-0.16,0.63)<br>Leg Pain: 0.01(-0.39,0.40); **PCS:** 0.27(-0.12,0.67); **SEE:** -0.05(-0.45,0.34); **EQ-5D:**0.23(-0.16,0.63), EQVAS:-0.05(-0.44,0.35); **PSFS:**0.35(-0.06,0.76)<br>**3 months post-op**<br>**ODI:** 0.04(-0.34,0.43) **Pain:** Back Pain: 0 (-0.38,0.39), Leg Pain: -0.04(-0.43,0.35); **PCS:** -0.08(-0.47,0.30); **SEE**: 0.04(-0.35,0.43); **EQ-5D**:-0.03(-	NR

© 2023 Punnoose A et al. *JAMA Network Open*.

**Author, Year, Location, Type of Surgery, [Risk of Bias]**	**Number of participants, sex and mean age.**	**Intervention vs Usual care Mode.**	**Results: Between group comparison Intervention, Usual care, Mean ± SD (unless reported otherwise)**	**Adverse events**
			0.42,0.35), EQVAS:0.01(-0.38,0.4); **PSFS:**0.30(-0.10,0.70); **TUG:** 0.19(-0.21,0.59); **5min walk**: 0.05(-0.35,0.45); **1 min Stair climbing**: 0.02(-0.38,0.42)<br>**6 months post-op**<br>**ODI:** -0.16(-0.55,0.23)<br>Pain: Back Pain: -0.10(-0.49,0.29), Leg Pain: -0.08(-0.47,0.31); **PCS:** 0.02(-0.37,0.42); **SEE:** 0.06 (0.34,0.46); **EQ-5D**-0.25 (-0.64, 0.14), EQVAS-0.12(-0.52,0.27); **PSFS:** 0.17(-0.25,0.59); **TUG**: 0.05(-0.35,0.46); **5min walk**: -0.9(-1.7,-0.1); **Stair climbing**: -0.18(-0.59,0.23)	
Marchand,[27]2022, Canada, Spinal surgery, [Low]	68<br>35 Intervention<br>33 Usual care<br>40 Males<br>28 Females<br>69 yrs	Exercises vs usual care<br>Unimodal	**Pre-op**<br>**Leg pain:** 5.5 ± 2.3, 6.5 ± 2.4;<br>**Back Pain:** 4.5 ± 2.2, 5.0 ± 2.7; **LSS disability:** 35.0 ± 7.9, 37.5 ± 6.5; **Back disability:** 35.3 ± 17.4, 39.5 ± 14.9; **Kinesiophobia:** 44.2 ± 7.7, 47.6 ± 8.0; **Depression**: 4.3 ± 5.3, 5.3 ± 5.2; **Trunk Ms**: Flex(Nm): 53.2 ± 24.8, 42.1 ± 26.1,Ext (Nm): 57.0 ± 61.0, 26.3 ± 26.7<br>Lumbar extensor endurance (sec)**:** 65.7 ± 65.6, 17.9 ± 35.8<br>**ROM:** Lumbar flex: 68.6 ± 27.2, 62.3 ± 25.8 , Lumbar Ext: 16.8 ± 6.9, 12.7 ± 7.1, Left lateral flexion: 14.1 ± 8.8, 10.6 ± 5.3, Right lateral flex: 13.2 ± 8.2, 11.2 ± 5.5;<br>**Walking capacity(sec):**<br>Time to 1st symptoms: 158.8 ± 102.4, 79.4 ± 87.4, Total ambulation time: 231.7 ± 92.2, 146.3 ± 104.7; **TUG (sec):** 7.5 ± 1.9, 9.7 ± 2.6; **Sit to Stand (reps):** 12.1 ± 3.6, 8.4 ± 2.2<br>**PGIC:** 2.9±1.3, 4.5 ± 1.0<br>Improvement in symptoms: 69% (intervention), 11.5% (usual care)<br>Worsening of symptoms: 13%(intervention), 46% (usual care)<br>**6 weeks post-op**<br>**Leg pain:** 5.5 ± 2.3, 6.5 ± 2.4; **Back Pain:** 2.1 ± 2.2, 2.8 ± 2.7; **LSS disability**: 29.6 ± 8.5, 28.5 ± 10.6; **Back disability**: 16.5 ± 15.0, 14.3 ± 15.8; **Kinesiophobia**: 38.2 ± 8.9, 38.9 ± 9.3; **Depression**: 2.0 ± 3.4, 1.8 ± 3.1;<br>**Trunk ms. strength:**<br>Flex: 54.4 ± 28.5, 50.1 ± 27.8,Ext: 57.0 ± 61.0, 26.3 ± 26.7 , Ext endurance(sec)**:** 69.5 ± 59.2, 17.9 ± 35.8; **ROM:**Lumbar flex: 68.8 ± 17.6, 72.9 ± 19.4, Lumbar Ext: 16.8 ± 6.9, 12.7 ± 7.1, Left lateral flex: 13.5 ± 5.7, 12.8 ± 5.4, Right lateral flex: 14.4 ± 6.6, 15.3 ± 7.9; **Walking capacity(sec):**<br>Time to 1st symptoms: 223.1 ± 106.5, 199.6 ± 122.8; Total ambulation time: 262.2 ± 78.5, 262.0 ± 78.1; **TUG(sec)**: 6.7 ± 1.3, 8.0 ± 2.7; **Sit to stand(reps)**: 11.8 ± 2.2, 12.0 ± 2.1	Intervention<br>0 intra-operative complications<br>Usual care<br>2 intra-operative complications

© 2023 Punnoose A et al. *JAMA Network Open*.

**Author, Year, Location, Type of Surgery, [Risk of Bias]**	**Number of participants, sex and mean age.**	**Intervention vs Usual care Mode.**	**Results: Between group comparison Intervention, Usual care, Mean ± SD (unless reported otherwise)**	**Adverse events**
			**3 months post-op**<br>**Leg pain:** 2.9 ± 3.1, 2.2 ± 2.3; **Back Pain:** 1.6 ± 1.7, 2.6 ± 2.4 ;**LSS disability**: 37.9 ± 10.2, 39.2 ± 14.4; **Back disability**: 15.8 ± 13.6, 22.0 ± 13.9; **Kinesiophobia**: 37.9 ± 7.8, 41.3 ± 7.3; **Depression**: 2.7 ± 3.5, 3.9 ± 4.6<br>**6 months post-op**<br>**Leg pain:** 2.4 ± 2.8, 3.1 ± 2.5 **Back Pain:** 2.4 ± 2.6, 3.7 ± 2.6; **LSS disability**: 30.5 ± 10.1, 45.0 ± 21.6; **Back disability**: 12.7 ± 16.0, 24.3 ± 14.3; **Kinesiophobia**: 37.6 ± 7.6, 40.4 ± 10.7; **Depression**: 1.7 ± 2.0, 3.4 ± 3.3; **EQ-5D:**No data available; **LOS(days):** 4.1 ± 3.2, 4.5 ± 2.0	
Mat-Eismail,[28] 2016, Malaysia, TKR, [High]	50<br>24 Intervention<br>26 Usual care<br>7 Males<br>43 Females<br>64.6years	Pre-op exercises vs usual care Unimodal	Results reported as Mean(95%CI)<br>**Pre-op:** Data unclear<br>**6 weeks post-op**<br>**KOOS**: **Symptoms**:88.22(82.99,93.45),88.84(82.99,93.45), **Pain:** 86.94 (81.06,92.82), 82.95(77.30,88.60), **ADL:** 81.58(75.37,87.780),74.52(68.56,80.48), **Sports & Rec:** 39.58(34.35,44.82),32.12(27.09,37.14), **QOL**: 55.49(49.93,61.05), 51.76 (46.42,57.10) **ROM: Flex**:94.58(88.05, 101.12), 89.23 (82.95,95.51)<br>**3 months post-op**<br>**KOOS: Symptoms:** 96.63(92.36,100.90),90.21(86.10,94.31), Pain:94.35 (90.49,98.23),88.06(84.34,91.77), **ADL**:89.50 (85.04,93.97), 80.56(76.27,84.84), Sports and Rec:42.71(37.14,48.27),34.81(29.46,40.15), **QOL:** 64.08(57.08,71.08),57.57(50.84,64.29); **ROM:** Flex:108.75(104.68, 112.82), 102.31(98.4, 106.22)	Intervention<br>2 Infection<br>Usual care<br>2 infection<br>1 fracture<br>1 contracture
McKay,[29] 2012, Canada, TKR, [High]	22<br>10 Intervention<br>12 Usual care<br>10 Males<br>12 Females<br>62 years	Pre-op exercises vs sham Unimodal	**Pre-op**<br>**WOMAC:** Pain: 8.70±3.77, 9.00 ±4.41, Function: 28.50± 12.57, 30.50 ±13.68; **SF-36 Physical:** 29.66±7.99, 25.61± 5.77; **SF-36 Mental:** 52.76±7.79, 42.28±15.28<br>**Arthritis Self Efficacy Scale**: 141.70±26.3, 141.08 ±33.84<br>**Quads Strength affected leg(Nm/Kg):**1.03± 0.57, 0.81±0.52; **50 feet Walking Test(sec):** 11.38±5.95,12.63± 3.51; **Stair Test(sec):** 26.86±24.89, 23.28±11.70<br>**6 weeks post-op**<br>**WOMAC** Pain:5.60±2.72, 4.92±4.5, Function: 18.10±11.85, 19.17±15.01<br>**SF-36 Physical:**31.79±8.25, 29.80±6.71; **SF-36 Mental**: 49.35±10.47, 46.68±15.97;<br>**Arthritis Self Efficacy Scale:**	1 Intervention<br>0 Sham<br>Further data unavailable

© 2023 Punnoose A et al. *JAMA Network Open*.

Author, Year, Location, Type of Surgery, [Risk of Bias]	Number of participants, sex and mean age.	Intervention vs Usual care Mode.	Results: Between group comparison Intervention, Usual care, Mean ± SD (unless reported otherwise)	Adverse events
			159.20± 31.82, 158.08±25.54<br>**Quads Strength affected** leg(Nm/Kg):0.60±0.39, 0.57±0.29; **50 feet Walking Test(sec);**14.23±7.55, 13.11±3.30; **Stair Test(sec):** 30.53±24.85, 26.72±12.05<br>**12 weeks post-op**<br>**WOMAC** Pain: 4.40±3.20, 3.58±4.40, Function:13.10±11.56, 14.33±15.42;<br>**SF-36 Physical**:41.25±10.06, 34.83±9.78; **SF-36 Mental**:48.02±17.45, 51.46±16.37; **Arthritis Self Efficacy Scale**:178.10±19.60, 166.58±25.99; **Quads Strength affected leg (Nm/Kg)**: 0.77± 0.56, 0.74± 0.35; **50 feet Walking Test(sec)**: 11.80±5.66, 11.82±2.97; **Stair Test(sec):** 26.99±26.73, 22.18±10.98	
Mitchell,[30]2005, UK, TKR, [Some Concerns]	114<br>57 Intervention<br>57 Usual care<br>24 Males<br>33 Females<br>70 years	Pre-op+ Post-op home ex vs usual care Unimodal	Results reported as Mean±SD, Regression Coefficient (95%CI)<br>**12 weeks post-op**<br>**WOMAC: Physical Function** 24.9±13.4,26.4±14.9, -1.0 (-5.9,3.8);<br>**Pain**: 6.8±3.7,6.9±4.3, -0.5 (-2.0,1.0), **Stiffness** 3.5±1.4,3.6±2.1,-0.2 (-0.9, 0.4);<br>**SF-36:**<br>**General Health:** 61.0±23.4, 61.0±22.9,-0.2 (-7.0, 6.7), **Mental Health** 68.0±20.4,71.2±20.0, -2.9 (-9.3, 3.5), **Bodily Pain** 46.6±20.6,48.5±26.8,-3.4 (-12.0 5.2), **Physical Function** 41.6±22.2,43.3±27.6, 2.5(-6.3 11.3)**Role Emotional** 48.0±46.7), 45.6±44.8,4.1 (-10.9, 19.0), **Role Physical** 27.6±37.1,23.2±36.2,7.8(-5.6, 21.2) **Social functioning**, 64.1±26.6,60.8±33.1,6.7 (-3.4 16.7) **Energy**: 50.7±19.5,48.2±23.7, 3.4(-3.5,10.3)<br>**LOS (days):** 9.2±3.4, 9.6±3.0; **Healthcare Costs (Total):** £5376±916, £5372±832;<br>**SF-6D(QALYS):** 0.57±0.09, 0.56±0.12, 0.002 (-0.034,0.039)	NR
NCT03044028 [31] USA,TKR, [High]	66<br>22 Pre-op and post-op NMES<br>22 Post-op NMES<br>22 Usual care<br>11 Males<br>35 Females<br>64.9 years	Pre-op and Post-op NMES vs Post-op NMES only vs usual care Unimodal	Results reported as Mean change±SD from baseline for Pre+Post NMES, Post NMES, usual care<br>**6 weeks post-op**<br>Ms. strength: Quads: 3.35±20.70/15.95±14.61/-5.48±16.53<br>**12 weeks post-op**<br>**Pain:** 2.00±2.92, 1.80±2.04, 1.18±2.36<br>**KOOS: Physical function:**	0 Pre-op and Post-op NMES<br>0 Post-op NMES<br>Usual care<br>1 GI disorder

© 2023 Punnoose A et al. *JAMA Network Open*.

Author, Year, Location, Type of Surgery, [Risk of Bias]	Number of participants, sex and mean age.	Intervention vs Usual care Mode.	Results: Between group comparison Intervention, Usual care, Mean ± SD (unless reported otherwise)	Adverse events
			33.43 ±14.76, 20.39±12.70, 26.62±9.81, **Pain**: 72.22±19.64,78.89±16.57, 80.38 ±15.43; **ROM:** Between group difference reported<br>0.50±13.22, 6.67±15.4, -1.50±12.93<br>**LOS in days (Mean difference between groups):**<br>1.83±0.72, 1.75±0.45, 1.68±0.78<br>**Number of patients discharged to extended care facility**: 0(0%),0(0%), 1(4.5%)<br>**Readmission rates:** 0(0%),0(0%),1(4.5%)<br>No. of Op PT visits:<br>12.86±4.95,16.25 ±7.29, 11.67±5.06	
NCT01844934,[32] USA, TKR, [High]	43<br>9 Intervention<br>10 Usual care<br>NR Males<br>NR Females<br>NR years	Pre-op ex vs usual care; Unimodal	**Pre-op**<br>**UCLA activity score**: 4.78± 1.86,4.50 ±1.43;<br>**6MWT(feet):**1450.89±306.69,1266.90 ±257.32;<br>**30 sec sit to stand (n):** 14.89±6.07, 16.80±8.23;<br>**Pain after sit to stand**: 4.28±2.55,2.4±1.58;<br>**Gait speed (sec):** 8.76±1.14, 10.31±1.65;<br>**Gait speed pain:** 3.17±2.81,2.15±1.20; **Ms strength:** Quads: 3.56±41.91, 52.70±33.04; **ROM:** Flex: 129.44±10.43,116.50±12.83<br>Ext: -3.22±5.24, -3.50±7.04<br>**4 weeks post-op**<br>**UCLA activity score:** 3.11±0.60,3.90±1.37<br>**6 MWT(feet):** 998.56±351.65,1006.20±303.19;<br>**30 sec sit to stand (n):** 11.99±5.51,13.20±6.56;<br>**Pain after sit to stand:** 2.39±2.67,2.75±2.03;<br>**Gait speed (sec):** 12.66±3.27, 13.95±6.41;<br>**Gait speed pain:** 2.72±2.0,1.95±1.72;<br>**Ms strength:** Quads: 40.99±20.26, 39.99±32.62<br>**ROM:** Flex: 103.22±14.44, 105.20±14.16; Extension: -8.56±7.97, -6.0±10.10<br>**14 weeks post-op**<br>**UCLA activity score:** 5.67±1.41,5.10±2.38;<br>**6MWT(feet):**1456.56±365.1,1387.60±301.93;<br>**30 sec sit to stand (n):** 16.00±6.38, 17.50±8.63;<br>**Pain after sit to stand:** 2.56±1.96,0.90±1.91<br>**Gait speed (sec):** 8.81±1.43, 9.59±1.42;<br>**Gait speed pain:** 1.39±1.32,0.40±0.97;<br>**Muscle strength:** Quads:70.16±34.89, 66.82±31.04;<br>**ROM:**Flex: 117±15.39, 118±12.33 ext: -3.67±4.36, -3.80±6.16	NR

© 2023 Punnoose A et al. *JAMA Network Open*.

Author, Year, Location, Type of Surgery, [Risk of Bias]	Number of participants, sex and mean age.	Intervention vs Usual care Mode.	Results: Between group comparison Intervention, Usual care, Mean ± SD (unless reported otherwise)	Adverse events
Nielsen,[33]2010, Denmark, Lumbar Surgery, [Some Concerns]	60<br>38 Intervention<br>35 Usual care<br>24 Males<br>36 Females<br>50 years	Pre-op exercises vs usual care<br>Unimodal	Results reported as Median (IQR)<br>**Pre-op**<br>**RMDQ**: 14 (1–21), 17 (7–23); **Pain:** Back Pain: 41 (0–78),53 (0–96), Radiating Pain: 41 (0–78),53 (0–96); **TUG(sec):** 9 (6–31),11 (7–27)<br>**Sit to Stand(sec):** 14(8–22),18 (8–31); **HRQol-15D**: 0.85(0.74–0.99),0.82(0.65–0.92)<br>**At Discharge**<br>**RMDQ**:NR;<br>**Pain: Back Pain:** 31 (2–77) 21 (0–85); **Radiating Pain**:NR<br>**TUG(sec):** 14 (8–26),13 (8–35); **Sit to Stand(sec):**NR;<br>**HRQol-15D**: 0.83(0.62–1.00),0.79(0.48–0.94)<br>**1-month post-op**<br>**RMDQ**: 12 (3–21), 17 (1–23); **Pain: Back Pain**: 17 (0–50),31(0–87), Radiating Pain: 17 (0–50),31 (0–87); **TUG (sec):** 8 (5–31),9(5–18)<br>**Sit to Stand(sec)**: 12 (8–28) 14 (5–32); **HRQol-15D:** 0.88(0.74–0.99),0.86(0.66–0.96)<br>**3-month post-op**<br>**RMDQ**: 8 (0–20),11 (0–22); **Pain: Back Pain:** 10 (0–93),16 (0–81), **Radiating Pain:** 10 (0–93),16 (0–81)<br>**TUG (sec):** 8 (5–17),9 (5–14)<br>**Sit to Stand(sec):** 11 (8–23),12 (8–33); **HRQoL-15D**: 0.90(0.73–1.00),0.89(0.62–1.00)<br>**6-months post-op**<br>**RMDQ: 8 (0–20),11 (0–23); Pain**: Back Pain: 10 (0–69) 21 (0–62), Radiating Pain: 10 (0–69), 21 (0–62); **TUG(sec):** 8 (5–18),8(5–13)<br>**Sit to Stand(sec):** 12 (7–20),13 (7–27); **HRQol-15D**: 0.91(0.73–1.00)0.90,(0.69–1.00); **LOS(days):** 5 (4–9),7 (5–15)	3 Intervention<br>1 Usual care<br>Further data unavailable
Oosting,[34]2012, Netherlands, THR, [Some Concerns]	30<br>15 Intervention<br>15 Usual care<br>6 Males<br>6 Females<br>76years	Pre-op exercises vs usual care	Results reported as Means± SD, [Adjusted Mean difference between groups (95% CI)]<br>**Pre-op**<br>**TUG (sec):** 14.9±7.6, 17.2±13.8,[2.9 (-0.9,6.6)]; **CRT(sec):** 33.5±11.2, 34.5±14.8,[3.9 (-6.7,14.4)]; **6MWT(meters):** 288±88, 296±113,[-41 (-74,-8);<br>**Pain:** 5.9±2.1, 6.0±1.6,[0.0 (-1.1,1.2)]; **PSC-Q**: 5.5±1.9, 6.9±2.0,[1.1 (-0.3,2.4)]<br>**HOOS**: ADL: 65.2±10.6, 68.7±14.9,[3.6 (-4.9,12.1)], **Pain:** 66.8±9.2, 67.8±12.2,[3.8 (-3.1,10.7)], **Symptoms:**	Intervention<br>1 cardiac<br>4 wound infection<br>1 orthopaedic<br>1 sensory loss<br>1 shingles<br>Usual care<br>2 cardiac<br>3 wound

© 2023 Punnoose A et al. *JAMA Network Open*.

Author, Year, Location, Type of Surgery, [Risk of Bias]	Number of participants, sex and mean age.	Intervention vs Usual care Mode.	Results: Between group comparison Intervention, Usual care, Mean ± SD (unless reported otherwise)	Adverse events
			62.9±12.2, 64.0±18.3,[4.2 (-2.0,10.4)], **Sports and Recreation:**86.8±16.6, 83.2±13.5,[2.1 (-15.0,10.9)], QOL: 77.0±10.1, 80.4±11.1,[-0.8 (-7.9,6.3)], **LAPAQ(min/d):**<br>552±199, 476±379,[-68 (-254,119)]<br>**At discharge**<br>**LOS:** 5.1±1.0, 5.4±2.1<br>**6 weeks post-op**<br>TUG(sec): 13.4±5.5, 13.8±11.1,[-0.3 (-2.7,2.0)]; **CRT(sec):** 35.4±23.7,31.4±6.9,[5.8(-0.0,11.6)]; **6MWT(meters):** 282±84, 339±69,[9 (-39,58)];**Pain:** 1.9±1.4, 2.1±2.0,[0.2 (-1.2,1.5)]; **PSC-Q**: 3.8±2.6, 3.4±1.9,[-0.2 (-2.1,1.8)]<br>**HOOS:** ADL: 36.5±10.8, 40.0±13.3,[4.4 (-7.2,16.0)]<br>Pain: 34.5±8.6, 37.3±16.8,[3.6 (-7.5,15.0)], **Symptoms:**<br>44.9±15.4, 42.7±10.8,[-0.7 (-10.7,9.3)], **Sports and Recreation:** 52.5±18.7, 48.0±21.0,[-4.2 (-25.1,16.6)]<br>QOL: 53.2±16.4, 49.2±16.1, [-9.4 (-25.3,6.6)]; **LAPAQ(min/d):** 425±288, 507±429,[55 (-266,376)]	1 orthopaedic<br>2 cognitive impairment<br>1 renal<br>1 pressure sores<br>1 bowel obstruction
Riddle,[35]2019, USA, TKR, [Low]	402<br>137 Intervention1<br>135 Intervention 2<br>130 Usual care<br>135 Males<br>267 Females<br>63.2 years	Pre and Post-op CBT vs Education. only<br>vs usual care; Unimodal	Results reported as Mean (95%CI) for CBT, education only, usual care<br>**2 months post-op**<br>**WOMAC:** Pain:6.4(5.5,7.3), 6.1(5.2,7.0), 6.1(5.3,7.0), Physical: 22.5(19.2,25.7), 19.5(16.3 to 22.7), 21.2 (18.1,24.4), Pain: 3.1 (2.6 to 3.7), 3.1 (2.5 to 3.7), 3.4 (2.8 to 4.0), PCS: 9.3(6.5,12.1), 9.8(7.0,12.6), 9.5(6.8,12.3); **SPBB:** NR; 6MWT (metres):NR; GRCS:2.8(2.3,3.3),2.9(2.4,3.5, 2.9(2.3,3.4)<br>**6 months post-op**<br>**WOMAC;** Pain:4.1(3.2,5.0), 3.8(2.9,4.7), 4.4(3.6,5.3)<br>Physical:15.2(12.0,18.4), 14.7(11.5,17.9), 15.4 (12.3 to 18.6), Pain: 2.2 (1.6 to 2.8), 2.2(1.6,2.8), 2.3(1.8,2.9)<br>**PCS:** 6.9(4.1,9.7), 6.3(3.5,9.1), 7.2(4.5,10.0); **SPBB:**NR<br>**6 MWT(metres):**NR; GRCS:3.0(2.5,3.6), 3.7(3.1,4.2), 3.6(3.0,4.1)<br>**12 months post-op**<br>**WOMAC**<br>Pain: 3.3(2.5,4.2), 3.0(2.1,3.8), 2.9(2.0,3.8), Physical:12.2(9.0,15.4), 11.7(8.6,14.9), 10.5 (7.4,13.6)<br>Pain:1.8(1.2,2.4), 2.0(1.3,2.6), 1.7(1.1,2.2); **PCS:** 6.8(4.0,9.6), 7.2(4.4,10.0), 6.1 (3.4,8.9)<br>**SPBB**: 8.4 (7.6,9.1), 8.0 (7.2,8.7), 8.6 (7.8, 9.4)<br>**6 MWT(metres):** 366 (341,391), 337 (313,362), 363(340,387)	CBT<br>3 DVT<br>2 Infection<br>7 Readmission<br>7 MUA<br>2 Death<br>2 Distress<br>3 Revision<br>Ed. only<br>2 DVT<br>1 Infection<br>7 Readmissio1 UTI<br>7 MUA<br>1 Distress<br>3 Revision<br>Usual care<br>3 DVT<br>1 Infection<br>8 Readmission<br>3 MUA

© 2023 Punnoose A et al. *JAMA Network Open*.

Author, Year, Location, Type of Surgery, [Risk of Bias]	Number of participants, sex and mean age.	Intervention vs Usual care Mode.	Results: Between group comparison Intervention, Usual care, Mean ± SD (unless reported otherwise)	Adverse events
			**GRCS:** 3.6(3.1,4.2), 3.8(3.3,4.3),4.1 (3.6,4.6)	1 Revision
Risso,[36]2022, UK, TKR, [High]	21<br>9 Intervention<br>12 Usual care<br>13 Males<br>9 Females<br>71.1 years	Neuro-muscular exercises. vs usual care; Unimodal	**Pre-op (1 week before surgery)**<br>**EMD-** Rectus femoris: 33.5±6.0, 43.6±7.5, Vastus lateralis: 33.9±6.4,45.3±7.6;<br>**Msstrength Quads** (N): 211.9± 62.8, 171.7±44.9<br>**Day of surgery (Pre-op):**<br>**EMD-** Rectus femoris: 38.4± 7.1, 45.3±7.1, Vastus lateralis: 38.9 ±8.3, 46.8±7.4<br>**Ms strength Quads** (N): 207.1±66.3, 172.8±43.7	NR
Rolving,[37]2015, Denmark, Lumbar Surgery, [High]	90<br>59 Intervention<br>31 Usual care<br>39 Males<br>51 Females<br>49.5years	Pre and Post-op CBT vs usual care Unimodal	Results reported as Median Change in Scores (IQR)from baseline<br>**3 months post-op**<br>**ODI:** -15 (-26,-4), 1 (-14, 8)<br>**CSQ:** -5.0 (-10,2.0) ,-2.0 (-8.0,0.0)<br>**FABQ**: -1.0 (-3.0,1.0),0.0 (-5.0,3.0)<br>**LBP Rating Scale**<br>Back Pain:-3.0 (-4.3,-1.3),-2.6(-4.3,-0.3) Leg Pain:-3.2(-5.3,-1.3),-2.3(-4.7,-0.3)<br>**6 months post-op**<br>**ODI:** - 18(- 24,- 7),- 4 (- 16, 4)<br>**CSQ:** - 7.5(- 12,0.0) ,- 2.0(- 5.0,3.0)<br>**FABQ:** - 3.0(- 5.0, 1.0),0.0(- 2.0, 3.0)<br>**LBP Rating Scale**<br>Back Pain: - 2.3(- 4.0,- 1.7),- 2.3(- 4.7,-0.7) Leg Pain:- 2.8 (- 5.0,- 1.3), - 2.0 (-5.7,-0.3)<br>**1-year post-op**<br>**ODI:** - 14 (- 26,- 5),- 6 (-26,4), **CSQ:-** 5.0(- 10.0,0.0),- 5.5 (-11.0,-1.0), FABQ: -3.0 (- 7.0,0.0),- 2.5, (- 6.5,1.0);<br>**LBP Rating Scale**<br>Back Pain: - 2.5 (- 4.3,- 1.0),- 2.7(- 5.0,- 0.3); Leg Pain: - 2.8 (- 4.7,- 1.3),1.3(- 6.0, - 0.3)	Intervention<br>2 pain<br>0 Usual care
Rooks,[38]2006, USA, THR &TKR, [Some Concerns]	108<br>54 Intervention 54 Usual care<br>48 Males<br>60 Females<br>64.5years	Pre-op exercise vs usual care Unimodal	**THR**<br>**Pre-op**<br>**WOMAC** Pain: 7.8± 4.1, 9.9 ± 2.9, Function: 26.9 ±11.9, 33.7±10.9;<br>**SF-36**: Physical Function: 40.4 ±23.4, 30.3±17.1, Pain: 49.5±19.4,37.7±17.9, Role Physical: 44.6±37.5, 32.1±39.0<br>**Ms strength(kg)-Leg press**:102± 49,103±43; **TUG(sec):**11.35±2.35,11.3±2.25;<br>**Functional Reach(cm):** 30.6±6.6, 31.5±7.1<br>**8 weeks post-op**	Intervention<br>0 in THR<br>3 in TKR<br>Usual care<br>4 in THR<br>4 in TKR<br>No further data unavailable

© 2023 Punnoose A et al. *JAMA Network Open*.

Author, Year, Location, Type of Surgery, [Risk of Bias]	Number of participants, sex and mean age.	Intervention vs Usual care Mode.	Results: Between group comparison Intervention, Usual care, Mean ± SD (unless reported otherwise)	Adverse events
			**WOMAC** Pain: 2.6±2.6,2.7±2.0, Function: 12.8±9.0,12.9±8.0;<br>**SF-36** Physical Function: 57.6±22.0,55.1± 22.4, Pain: 71.4±20.1,70.8±21.2, Role Physical: 48.2±41.9, 47.2±42.4<br>**Msstrength(kg)-Leg press**: 80±32,106±51; TUG(sec): 11.53±2.42,10.9±2.83;<br>**Functional Reach(cm):** 30.7±6.9, 32.8±6.1<br>**26 weeks post-op**<br>WOMAC Pain: 1.1±1.7, 1.0±1.2, Function: 5.4±5.8, 5.3±5.4,<br>**SF-36**: Physical Function: 81.7±18.1, 76.6±18.6,Pain:79.6±21.2, 77.4±16.3,Role Physical: 83.0±35.2, 86.5±24.4<br>**Ms strength(kg)-Leg press**: 99±37, 117±51, TUG(sec): 9.76±1.29, 9.41±1.46;<br>**Functional Reach(cm):** 33.5±5.2, 31.4±7.1<br>**TKR**<br>**Pre-op**<br>**WOMAC:** Pain: 7.3±0.7, 7.5±5.0, Function: 27.7±11.6, 25.0±11.9;<br>**SF-36** Physical Function: 34.0± 21.5,40.2± 19.4, Pain: 42.1±16.6, 56.7±21.4, Role Physical: 20.8± 33.4, 58.9±41.2;<br>**Ms strength(kg)-Leg press:** 116± 37,102±46; **TUG(sec):** 12.15±2.65, 10.38±1.77; **Functional Reach(cm):** 26.7±7.6, 28.0±6.4<br>**8 weeks post-op**<br>**WOMAC** Pain: 4.7±2.4, 5.0±3.4, Function: 16.3±7.1, 15.3±11.4;<br>**SF-36** Physical Function: 49.9±15.0,53.1±26.3,Pain: 59.8±16.4, 68.1±16.6, Role Physical: 48.7±38.6, 49.4±39.8<br>**Ms strength(kg)-Leg press**: 99±47, 86±43; **TUG(sec):** 12.03±2.05,12.40±3.22;<br>**Functional Reach(cm):** 27.9±6.4, 26.7 ±7.6<br>**26 weeks post-op**<br>**WOMAC:** Pain: 2.4±2.7, 2.3±2.0, Function: 9.9±9.0, 1.4±11.9;<br>**SF-36:** Physical Function: 68.0±19.8, 66.1±26.6, Pain: 71.2±19.3, 68.1±25.1, Role Physical: 71.1±36.4, 58.3±46.9<br>**Ms strength(kg)-Leg press:** 129±42,111±49; **TUG(sec):** 10.08±1.76, 11.66±4.47;<br>**Functional Reach(cm):** 29.2±7.6, 27.9± 7.6;<br>**Combined THR &TKR at Discharge**<br>(IG=Intervention, UC=Usual care):<br>Discharged Home: Intervention(n=24), Usual care (n=20); Walk >50m: 27 in IG,21 in UC	

© 2023 Punnoose A et al. *JAMA Network Open*.

Author, Year, Location, Type of Surgery, [Risk of Bias]	Number of participants, sex and mean age.	Intervention vs Usual care Mode.	Results: Between group comparison Intervention, Usual care, Mean ± SD (unless reported otherwise)	Adverse events
Savkin,[39] 2021, Turkey, TKR, [High]	40<br>20 Intervention<br>20 Usual care<br>37 Males<br>3 Females<br>64yrs	NMES vs usual care Unimodal	**Pre-op**<br>**ROM:** Flex: 109.55±8.29, 105.05± 10.81, Ext: -13.45±5.47, -14.8 ±8.32<br>**Quads strength:** 103.66±14.39,95.08±22.9<br>**KOOS:** Symptoms: 55.54± 15.26, 41.61± 19.05, Pain: 53.06 ±17.93, 37.08± 15.35, ADL: 61.47±15.89, 42.65± 11.31, Sports & Rec: 4±15.61, 3.25±7.12, QOL: 33.75±20.22, 23.13±21.47;<br>**WOMAC (Total):** 39.6 ±13.74, 57.25±11.28; **CST:** 9.75±1.86, 9.8±2.14;<br>**40m walk test:** 39.7± 11.54, 45.24±12.44;<br>**Stair climb test:** 23.99±10.82, 28.61± 8.3<br>**4 weeks post-op**<br>**ROM:** Flex: 116.25±8.05, 112.15±6.52, Ext: -12.5±6.15,-12.2±5.62; **Quads strength:** 93.41±24.69, 95.84±19.96<br>**KOOS:** Symptoms: 76.61±11.67, 73.39±14.35, Pain: 73.06±17.2, 66.53± 17.67, ADL: 83.68±13.22, 73.53±17.76, Sports & Rec:18.25±15.41, 18±12.4, QOL: 62.81±23.95, 54.06± 26.54;<br>**WOMAC (Total):** 19.95±12.17, 24.35± 13.16 **CST:** 10± 2.22, 9.95±2.14<br>**40m walk test:** 38.07±8.94, 41.91±10.15; **Stair climb test:**24.36±9.41, 25.41±7.33<br>**12 weeks post-op**<br>**ROM:** Flex: 117.1±8.48, 116±7.81; Ext: -9.05±5.27,-9.25±5.88; **Quads strength:** 96.34 ±25.75, 98.01±22.79<br>**KOOS:** Symptoms: 81.61±12.49, 77.68±11.98, Pain: 83.89±15.2, 86.39± 12.96, ADL: 89.85±12.35, 87.21±11.19, Sports & Rec: 30.5± 20.89, 23.75±21.82, QOL: 73.75± 23.61,68.44±26.4<br>**WOMAC (Total):** 12.3±10.15, 15.25± 12.17; **CST:** 11.4±2.14, 11.6±2.3; **40m walk test:** 34.66±8.41, 38.92±7.42; **Stair climb test:**18.88±6.59, 22.43±7.35	NR
Shaarani,[40] 2013, Ireland, ACL reconstruction, [High]	20<br>11 Intervention<br>9 Usual care<br>20 Males<br>0 Females<br>29 years	Pre-op exercise vs usual care Unimodal	Data partly extracted from figures.<br>**Pre-op**<br>**Single leg Hop Test** (cms): 183.1±15.55, 156.0±42.98 **Total Modified Cincinnati Score:** 76.5, 70.0 **Ms Strength affected leg:** Quads:150.0,135.0, Hamstrings:110.0,97.0,<br>**12 weeks post-op**<br>Single leg Hop Test(cms): 144.91±15.52,113.33±25.54; **Total Mod Cincinnati Score:** 85.3,77.6,<br>**Muscle strength affected leg** Quads:100.0,90.0 Hamstring:98.0, 80.0<br>**Return to Sport (weeks):**	NR

© 2023 Punnoose A et al. *JAMA Network Open*.

Author, Year, Location, Type of Surgery, [Risk of Bias]	Number of participants, sex and mean age.	Intervention vs Usual care Mode.	Results: Between group comparison Intervention, Usual care, Mean ± SD (unless reported otherwise)	Adverse events
			34.8±4.14,42.5±10.46	
Skoffer,[41] 2016, Denmark, TKR, [Low]	59<br>30 Intervention<br>29 Usual care<br>23 Males<br>36 Females<br>70years	Pre-op exercise vs usual care Unimodal	**Pre-op**<br>**Functional Performance:**<br>CST(Reps): 13.3±5.1, 11.1±2.9, TUG(sec): 9.1±2.6, 9.3±3.0,10m WT(sec): 7.3±1.6, 8.0±2.0, 6MWT(metres): 434±101, 427±76<br>**Knee Ms strength(Nm/Kg)-Affected leg**: ISK flexion: 0.6±0.3, 0.5±0.2, ISK extension: 0.9±0.4, 0.9±0.4, ISM flexion: 0.8±0.3, 0.6±0.2, ISM extension: 1.2±0.4, 1.0±0.4; **Knee Ms. Strength(Nm/Kg)-Unaffected leg:** ISK flexion: NR, ISK extension: NR, ISM flexion: 0.7±0.3, 0.7±0.3, ISM extension: 1.4±0.4, 1.3±0.5;<br>**Average Pain(past 14 days):** 3.4±2.5, 5.0±1.9<br>**KOOS:** Pain: 59.2±15.7, 55.2±18.5, Symptoms: 66.0±14.9, 59.2±21.2, ADL: 64.1±16.1, 60.6±15.9, Sports/Rec: 29.5±17.4, 19.8±19.5, QoL: 38.2±15.0, 34.1±17.1; **HRQoL:** 71.6±20.7, 64.9±15.7**; ROM:** Flex: 120.76±10.8, 120.86±8.8, Ext: 5.76±3.8, 6.66±3.2<br>**1-week post-op**<br>**Functional Performance:**<br>CST(Reps): 4.4±5.1, 2.2±3.5,TUG(sec): 14.8±5.2, 17.0±5.6,<br>10m WT(sec): 12.5±4.9, 14.4±5.6, 6MWT(metres): 258±93, 226±82<br>**Knee Ms. Strength(Nm/Kg)-Affected Leg:** NR<br>**Knee Ms. Strength(Nm/Kg)-Unaffected leg:** ISK flexion: NR, ISK extension: NR,ISM flexion: NR, ISM extension: NR<br>**Average Pain (past 14 days):** 4.2±2.5, 5.3±2.2<br>**KOOS:** Pain: 53.9±15.2, 47.7±16.8, Symptoms: 52.5±17.9, 48.1±12.5, ADL: 56.2±16.1, 50.1±21.1 Sports/Rec: 18.4±17.1, 11.7±22.3, QoL: 38.4±15.6, 35.3±16.8, **HRQoL:** 69.7±18.6, 59.2±19.6; **ROM:** Flex: 85.16±13.2, 86.46±19.9, Ext: 7.76±3.2, 7.26±3.4<br>**6 weeks post-op**<br>**Functional Performance:**<br>CST(Reps): 13.3±5.0, 9.6±4.4 TUG(sec): 8.3±2.3,10.0±2.4<br>10MWT(metre): 7.6±1.8, 8.6±1.6, 6 MWT: 424±103, 376±83; **Knee Ms.Strength** (**Nm/Kg)-Affected leg**:<br>ISK flexion: 0.5±0.2, 0.4±0.2	0 Intervention<br>0 Usual care

© 2023 Punnoose A et al. *JAMA Network Open*.

Author, Year, Location, Type of Surgery, [Risk of Bias]	Number of participants, sex and mean age.	Intervention vs Usual care Mode.	Results: Between group comparison Intervention, Usual care, Mean ± SD (unless reported otherwise)	Adverse events
			ISK extension: 0.7±0.2, 0.6±0.2; ISM flexion: 0.7±0.3, 0.5±0.2; ISM extension: 0.9±0.3, 0.6±0.3<br>**Knee Ms. Strength(Nm/Kg)-Unaffected leg:** ISK flexion: NR, ISK extension:NR<br>ISM flexion: 0.7±0.2, 0.6±0.2<br>ISM extension: 1.4±0.4, 1.2±0.4<br>**Average Pain(past 14 days):**<br>2.3±1.7, 2.1±1.2<br>**KOOS:** Pain: 70.9±16.7, 67.3±13.0, Symptoms: 66.7±16.7, 64.0±12.3, ADL: 76.1±13.8, 70.6±11.4<br>Sports/Rec: 36.4±24.3, 18.9±19.2, QoL: 56.3±20.6, 50.0±13.3; **HRQoL**: 83.2±14.0, 67.2±23.8; **ROM:** Flex: 108.66±11.0, 106.16±13.8, Extension: 5.26±2.9, 5.26±3.2<br>**12 weeks post-op**<br>**Functional Performance:**<br>CST(Reps): 14.7±4.7, 11.0±4.4, TUG(sec): 7.9±2.3, 8.9±2.1, 10MWT(metre): 7.1±1.5, 7.7±1.2, 6 MWT: 449±94, 433±74; **Knee Ms.Strength(Nm/Kg)-Affected leg:** ISK flexion: 0.5±0.2, 0.4±0.2, ISK extension: 0.9±0.3, 0.7±0.2, ISM flexion: 0.7±0.3, 0.6±0.2<br>ISM extension: 1.0±0.3, 0.8±0.3; **Knee Ms. Strength (Nm/Kg)-Unaffected leg:** ISK flexion:NR, ISK extension:NR, ISM flexion: 0.7±0.2, 0.6±0.2, ISM extension: 1.4±0.4,1.3±0.4;<br>**Average Pain (past 14 days):**<br>1.4±1.6, 1.5±1.1<br>**KOOS:** Pain: 78.1±16.3, 79.9±14.2, Symptoms: 72.8±16.4, 71.9±11.4, ADL: 82.9±11.7, 78.2±12.9, Sports/Rec: 50.2±28.4, 40.0±22.5, QoL: 66.2±18.9, 61.9±16.6; **HRQoL**: 86.7±10.5, 76.4±20.1; **ROM:** Flex: 113.06±14.8, 112.56±7.81,<br>Extension: 3.36±2.8, 4.36±2.4<br>**1 year post-op**<br>**Functional Performance:**<br>CST(Reps): 14.7±3.8, 13.1±3.1, TUG(sec): 7.5±2.2, 7.7±6.6, 10m WT(sec): 6.7±1.3, 7.0±1.1, 6MWT(metres): 479±92, 474±74; **Knee Ms. Strength (Nm/Kg)-Affected leg**: ISK flexion: 0.9±0.2, 0.8±0.3, ISK extension: 1.4±0.3,1.3±0.4<br>**Knee Ms. Strength(Nm/Kg)-Unaffected leg:** Flexion: 0.8±0.2,0.7±0.3**,** Extension: 1.6±0.4, 1.5±0.5<br>**Average Pain(past 14 days):**<br>1.1±1.4,0.6±0.7	

© 2023 Punnoose A et al. *JAMA Network Open*.

Author, Year, Location, Type of Surgery, [Risk of Bias]	Number of participants, sex and mean age.	Intervention vs Usual care Mode.	Results: Between group comparison Intervention, Usual care, Mean ± SD (unless reported otherwise)	Adverse events
			KOOS: Pain: 89.9±13.2, 89.0±10.1, Symptoms: 86.5±13.1,83.4±14.5, ADL: 87.6±12.3,84.4±11.8, Sports/Rec: 59.5±27.5, 55.0±18.4, QoL: 78.6±19.1, 73.4±15.2; **HRQoL:** 89.1±10.9, 76.8±22.3; **ROM:** Flex: 117.3±8.4, 116.4±19.8, Extension: 1.8±2.3, 2.4±2.8	
Soni,[42]2012 UK,TKR, [Low]	56<br>28 Intervention<br>28 Usual care<br>56 Males<br>0 Females<br>67.5 years	Pre-op Acupuncture and exercises vs usual care; Multimodal	**12 weeks pre-op**<br>**OKS:** 21±21.1, 19.8±12.2;<br>**Pain:** 6.0±2.7, 5.8±3.0,<br>**50m timed walk(sec):** 61.7±34.3, 61.0±33.0<br>**3 months post-op:**<br>**OKS:** 25.1±10.6, 27.4±10.0; **Pain:** 4.7±2.8, 3.9±3.0;<br>**50m timed walk(sec):** 64.1±44.7,55.0±18.4	NR
Swank,[43]2011, USA, TKR , [High]	71<br>35 Intervention<br>36 Usual care<br>25 Males<br>46 Females<br>63years	Pre-op exercises vs usual care Unimodal	Results reported as Mean±SD (95%CI)<br>**Pre-op**<br>**Muscle strength affected side:**Knee Flex: 32.3±3.7(31.1,33.5),27.1±3.8(25.9,28.3), Knee Ext: 60.0±5.4(58.3,61.7),50.7±5.5(48.9,52.5)<br>**Muscle strength unaffected side**: Knee Flex: 39.8±4.2(38.5,41.2),36.0±4.2(34.6,37.4), Knee Ext: 78.9±7.2(76.6,81.2),78.4±7.4(76.0,80.8); **Functional Tasks**<br>6 MWT(metres): 392.8±15.2(387.9,397.7), 363.3±15.6(358.2,368.4); 30 Sec Sit to Stand (reps): 11.5±0.65(11.3,11.7), 9.6± 0.68(9.4±9.8)<br>Ascend Stairs (1st flight) in sec: 11.0±1.3(10.6,11.4), 11.1±1.3 (10.7,11.5), Ascend stairs (2nd flight) in sec:11.1±1.2(10.7,11.5),12.5±1.2(12.1,12.9), Descend Stairs(1st Flight) in sec:12.0±1.9(11.4,12.6),10.9±2.0(10.3,11.6), Descend Stairs(2nd Flight)in sec:11.0±1.3(10.6,11.4),12.2±1.3(11.8,12.6); **Pain after Functional Tasks:**<br>6 MWT: 4.3±0.38(4.2,4.4), 5.1±0.42(5.0,5.2)<br>Sit to Stand: 5.3±0.44(5.2,5.4), 4.7±0.43(4.6,4.8) Stair ascend:5.9±0.45(5.8,6.1),4.6±0.66(4.5,4.8), Stair descend: 6.1±0.44(6.0,6.2), 4.8±0.44(4.7,5.0)	NR
Topp,[44]2009, USA, TKR, [Some Concerns]	54<br>26 Intervention<br>28 Usual care<br>17 Males<br>37 Females	Pre-op exercises vs usual care; Unimodal	Results reported as Mean± SEM<br>**Pre-op**<br>**Knee Ms strength affected side:** Max Ext (Torque/Body Weight): 56.5±16.16, 54.02±5.94<br>**Knee Muscle strength unaffected side:**	NR

© 2023 Punnoose A et al. *JAMA Network Open*.

Author, Year, Location, Type of Surgery, [Risk of Bias]	Number of participants, sex and mean age.	Intervention vs Usual care Mode.	Results: Between group comparison Intervention, Usual care, Mean ± SD (unless reported otherwise)	Adverse events
	64years		Max ext (Torque/Body Weight):<br>79.5±9.03, 82.74±8.70, Strength Asymmetry<br>(non-surgical minus surgical side): 22.98±6.14, 28.72±5.92<br>**Functional Tasks &[Pain after the tasks]:**6MWT:<br>1282±59[4.77±0.45],1185.18±56[6.80±0.43], 30 sec Sit to Stand reps:12.08±0.83 [4.03±0.46], 9.82±0.80 [4.91±0.45], Stair ascend:<br>10.63±1.12[4.34±0.51],10.36±1.08 [5.54±0.50], Stair descend:10.39±1.26 [4.58±0.51],10.45±1.19[5.65± 0.48]<br>**1 month post-op**<br>**Knee Muscle strength affected side:**Max ext(Torque/Body Weight):43.15±3.63, 44.41±3.50<br>**Knee Muscle strength unaffected side**:Max ext(Torque/Body Weight):<br>83.92±9.48, 95.45±9.13,<br>Strength Asymmetry<br>(non-surgical minus surgical side): 40.77±8.31, 51.04±8.00<br>**Functional Tasks[Pain after the tasks]:**6MWT: 1191±51 [2.17±0.37],1166.71±49[2.36±0.35],30 sec Sit to Stand reps:<br>11.46±.69[2.20 ± 0.39], 10.36±.67 [2.04 ± 0.37], Stair ascend: 11.98±1.36[2.03 ± 0.37], 10.39±1.31[2.14 ± 0.35],<br>Stair descend: 13.21±1.62 [1.83 ± 0.37], 11.79±1.53[2.43 ± 0.35]<br>**3 months post-op:**<br>**Knee Muscle strength affected side:** Max Ext(Torque/Body Weight): 62.27 ± 5.00, 60.74 ± 4.81<br>**Knee Ms.strength unaffected side:** Max ext(Torque/Body Weight):<br>90.26±9.14,94.73±8.81,Strength Asymmetry<br>(non-surgical minus surgical side): 28.00±6.93,33.98±6.67<br>**Functional Tasks[Pain after the tasks]:** 6MWT:<br>1337 ± 58[1.53 ± 0.34], 1365 ± 56[1.38 ± 0.33], 30 sec Sit to Stand reps:12.87 ± .82[1.62 ± 0.29], 11.25 ± .79[1.06 ± 0.28],<br>Stair ascend: 8.44 ±0.81[1.33 ± 0.31],7.45±0.77[1.26 ± 0.30],<br>Stair descend: 8.60 ± 1.12 [1.42 ± 0.37],8.06 ±1.06[1.45 ± 0.35]	
Villadsen,[45] 2013, Denmark, THR &TKR, [Low]	165<br>84 Intervention 81 Usual care<br>73 Males<br>92 Females	Pre-op exercises vs usual care Unimodal	Results reported as Mean change between group (95%CI)<br>**THR**<br>**Pre-op**	Intervention<br>1 pain<br>Usual care<br>1 infection THR and 1 in TKR

© 2023 Punnoose A et al. *JAMA Network Open*.

Author, Year, Location, Type of Surgery, [Risk of Bias]	Number of participants, sex and mean age.	Intervention vs Usual care Mode.	Results: Between group comparison Intervention, Usual care, Mean ± SD (unless reported otherwise)	Adverse events
	67.4years		HOOS: ADL: -10.9 (-15.9,-5.8), Pain: -8.4 (-12.8,-4.0), Symptoms: -3.2(-8.1,1.7), Sports and Recreation: -8.0 (-13.8,-2.2), QoL: -4.9 (-10.1,0.3), **Functional measures:**<br>5 chair stands(sec): 1.9 (0.5,3.4), 20m self-chosen pace (sec): 1.4 (0.1,2.7); 20m max pace (sec): 1.6 (0.3,3.0); **Ms strength (Watts):**Hip extension: -7.9(-20,4.8), Hip abd: -3.9 (-9.4,1.7)<br>**6 weeks post-op**<br>**HOOS:** ADL: -3.0 (-10.1,4.1), Pain: -3.0 (-10.3,4.4), Symptoms: -1.2 (-6.7,4.3), Sports and Recreation: 0.3(-6.9,7.6), QoL: 0.7 (-9.1,10.5); **EQ5D**: -5.1 (-13.2,3.0); EQ5DVAS: 0.06 (-0.13,0.00)<br>**3 months post-op**<br>HOOS: ADL: -3.2 (- 10.3, 3.9), Pain: -3.7 (-11.0, 3.7), Symptoms: -3.1 (-10.4,4.1), Sports and Recreation: -5.2 (-14.9,4.6) QoL: -2.6 (-10.7,5.5) **EQ5D:** -0.02 (-0.09,0.04), EQ5DVAS: -2.0 (-9.7,5.7) 0.61<br>**TKR**<br>**Pre-op**<br>**KOOS:** ADL: -3.5 (-8.8,1.8), Pain: -2.2 (-6.7,2.4),<br>Symptoms: -4.4(-9.4,0.7), Sports and Recreation: -1.1 (-7.1, 5.0), QoL: -6.3 (-11.6,-1.0)<br>**Functional measures:**<br>chair stands(sec): 1.9 (0.5,3.3), 20m self chosen pace (sec): 0.4 (-0.8,1.7),20m max pace (sec): 0.1 (-1.2,1.4);<br>**Muscle strength (Watts):** Knee ext: -11.6 (-25.6,2.5)<br>**6 weeks post-op**<br>**KOOS:** ADL: -5.6 (-12.9,1.8), Pain: -8.0 (-15.5,-0.4), Symptoms: -2.7 (-10.2,4.7), Sports and Recreation:-0.8 (-10.8,9.3), QoL: -5.0 (-13.2, 3.2), EQ5D: -0.03 (-0.09,0.04). EQ5DVAS: -8.8 (-16.4,-1.2)<br>**3 months post-op**<br>**KOOS** ADL: -5.6 (-12.9,1.8), Pain: -5.5 (-13.0,2.9), Symptoms: -6.0 (-13.4,1.5), Sports and Recreation: -5.6 (-15.6,4.5), QoL: -4.6 (-12.9,3.6); **EQ5D**: -0.06 (-0.13,0.01),EQ5DVAS: 2.8 (-4.8,10.4); **Ms strength**:<br>Hip Ext(15%) and Hip Abd(35%) improvement.<br>**Combined(THR&TKR) results reported at 12 months post-op:** Results reported as Mean(SE),[Mean between group difference (95%CI),Effect Size]<br>**12 months post-op**<br>**HOOS/KOOS**	

© 2023 Punnoose A et al. *JAMA Network Open*.

Author, Year, Location, Type of Surgery, [Risk of Bias]	Number of participants, sex and mean age.	Intervention vs Usual care Mode.	Results: Between group comparison Intervention, Usual care, Mean ± SD (unless reported otherwise)	Adverse events
			ADL: 35.9 (2.1), 32.1 (2.4), [3.80 (-2.45 to 10.07),0.24], Pain: 41.2 (2.2), 37.1 (2.4)-[4.13 (-2.33 to 10.60),0.29], Symptoms: 33.9 (2.7), 32.2 (2.6)-[1.69 (-5.79 to 9.17),0.09], Sports & Recreation: 33.1 (3.6), 26.3 (2.8)-[6.79 (-2.10 to 15.69),0.39], Quality of Life: 43.4 (2.6), 35.2 (3.0)-[8.25 (0.42 to 16.10),0.59];<br>**EQ-5D(QALYS):** 0.66(0.04), 0.61(0.04),[0.04,(0.01 to 0.07)]<br>**Total Healthcare Costs** €16181 (1174),€16313(1374), [€-132(-3668 to 3405)]<br>**LOS(days):** NR	
Walls,[46]2010, Ireland, TKR, [Some concerns]	14<br>9 Intervention<br>5 Usual care<br>4 Males<br>10 Females<br>65.4yrs	Pre-op Neuromuscular. electrical nerve stimulation vs usual care<br>Unimodal	**Pre-op**<br>Ms Strength (Data extracted from figures): Quads Peak Torque: Affected limb:110.0,95.0, Unaffected limb:125.0,92.0; **Functional measures**(Data extracted from figures):Stair-climb Test (sec):15.0,20.0, Chair-rise Test(sec):6.5,8.5, 25m times walk Test(sec):15.0,20.0<br>**WOMAC:** Pain: 11.4 ± 3.3, 11.8 ± 4.0, Function: 34.7 ± 11.0, 38.4±14.4, Stiffness: 5.1 ± 1.8, 5.4 ± 1.3, **SF-36:** Physical Health: 44.4 ± 18.7, 45.6 ± 17.8, Mental Health: 62.3 ± 17.5, 68.6 ± 23.1<br>**At Discharge**<br>LOS(days): 8.1,8.8, Discharged to Rehab facility: 2 in NMES group and 1 in usual care<br>**6 weeks post-op**<br>Ms Strength (Data extracted from figures): Quads Peak Torque:<br>Affected limb:50.0,45.0; Unaffected limb:120.0,95.0<br>**Functional measures** (Data extracted from figures):<br>Stair-climb Test(sec): 23.0,35.0, Chair-rise Test(sec):7.5,8.5, 25m times walk Test(sec):22.0,25.0<br>**WOMAC:** Pain: 7.9 ± 5.9, 5.4 ± 3.0, Function: 29.8 ± 16.9, 24.2 ± 9.9, Stiffness: 4.6 ± 2.4, 4.6 ± 1.1; **SF-36:** Physical Health: 35.7 ± 17.0, 39.0 ± 9.4; Mental Health: 58.6 ± 26.0, 58.8 ± 19.5<br>**12 weeks post-op**<br>Ms Strength (Data extracted from figures):Quads Peak Torque:<br>Affected limb:80.0,60.0, Unaffected limb:125.0,95.0<br>**Functional measures** (Data extracted from figures):<br>Stair-climb Test(sec): 17.0,25.0; Chair-rise Test (sec): 6.5,8.3, 25m times walk Test(sec):17.0,19.0<br>**WOMAC:** Pain: 5.4 ± 4.0, 5.2 ± 4.3, Function: 19.9 ± 13.9, 21.6 ± 11.3, Stiffness: 3.2 ± 2.0, 3.0 ± 1.4, **SF-36:** Physical Health: 57.1 ± 18.0, 57.0 ± 25.2, Mental Health: 77.7 ± 14.7, 71.0 ± 26.4	NR

© 2023 Punnoose A et al. *JAMA Network Open*.

Author, Year, Location, Type of Surgery, [Risk of Bias]	Number of participants, sex and mean age.	Intervention vs Usual care Mode.	Results: Between group comparison Intervention, Usual care, Mean ± SD (unless reported otherwise)	Adverse events
Williamson,[47] 2007, UK, TKR [Some Concerns]	181<br>60 Intervention1<br>60 Intervention2<br>61Usual care<br>84 Males<br>97 Females<br>71.2years	Pre-op Acupuncture vs exercises vs usual care Multimodal	Results reported as Mean±SD for Acupuncture, Physiotherapy, Control [Changes in Mean with Control(95%CI)]- Acupuncture, Physiotherapy<br>**12 weeks Pre-op**<br>**OKS:** 38.1±6.88), 38.8±8.71, 40.8±8.14, [-2.64 (-5.35,0.08), [-2 (-5.04 to 1.03)]<br>**50m Timed walking(sec):**<br>54.0±15.6/51.8±18.4/57.4±26.7 [-3.43(-11.32,4.45)],-5.66 (-13.93,2.61)]<br>**WOMAC:** 48.4±14.3, 49.4±17.3, 52.3±16.6 [-3.94 (-9.53,1.64)], [-3(9.08,3.13)], Pain: 6.58±2.29/6.36±2.6)/7.24±2.07, [-0.66 (-1.45,0.12)],[-0.88 (-1.72,–0.04)].<br>**At discharge LOS(days):** 7.73±3.96/6.49±1.99/6.6±2.62<br>**3 months Post-op**<br>**OKS**:25.6±8.95/ 28.3±9.78/ 26.7±7.45<br>[-1.18 (-6.23,3.87)],[1.61 (-3.91,7.13)], 50m Timed walking(sec): 46.9±8.93/ 46.6±11.4/44.1±6.91 [2.82 (-2.1,7.75), 2.51 (-3.48,8.51)]<br>**WOMAC:** 21.2±15.2/26±17.7/ 24.6±16.8, [-3.41(-13,6.17)], [1.33 (-9.53,12.18)] Pain:2.94±2.67/ 3.86±2.59/ 3.95±2.59, [-1.0 (-2.6,0.58)],[-0.09 (-1.71,1.53)]	0 reported in both groups
Zeng,[48]2014, China, THR, [High]	59<br>27 Intervention<br>32 Usual care<br>31 Males<br>28 Females<br>65years	Pre-op exercises vs usual care; Unimodal	**Pre-op**<br>**WOMAC:** Pain: 9.30 ± 1.97, 10.55 ± 2.02 Function: 36.28 ± 5.11, 41.08 ± 5.16<br>**6MWT(metres)**: 478.10±52.46, 419.02± 47.56 **TUG(sec):**14.61±2.60, 19.06 ±3.37 **UPST:** Data unavailable; **ROM:**<br>Flex: 97.80 ±7.97, 94.80 ± 7.20; Abd: 31.53 ±4.28, 28.79 ±4.56;<br>Further follow-up points unclear and data unavailable	0 reported in both groups

Abd = Abduction, ACL= Anterior cruciate Ligament, ADL= Activities of daily living, AIMS=Arthritis Impact Measurement Scale, APSI=Antero-Posterior Stability Index, BMRC= British Medical Research Council, CBT= Cognitive Behavioural Therapy, CG=Control Group, CRT= Chair rise test, CSA=Cross Sectional Area, CST=Chair Stand Test, DVT=Deep vein thrombosis, Ed=Education, EQ5D = EuroQol 5 Dimension, Ext = Extension, FAI= Femoroacetabular Impingement, Flex = Flexion, FS=Function score, GRCS= Global rating of change scale, HHS=Harris Hip Score, HOOS= Hip disability and Osteoarthritis Outcome Score, HRQoL-Health Related Quality of Life Measurement, IG=Intervention Group, IKDC=International Knee Documentation Committee, ILAS= Iowa Level of Assistance, IQR= Interquartile range, IS=Incentive Spirometry, ISK=Isokinetic, ISM=Isometric, KSS= Knee society score, KOOS= Knee injury and Osteoarthritis Outcome Score, LAPAQ= Longitudinal Aging Study Amsterdam Physical Activity Questionnaire LEFS=Lower Extremity Functional Scale, LOS=Length of Stay, LSI=Limb Symmetry Index, LSS=Lumbar spinal stenosis, MLSI= Medio-lateral Stability Index, Ms= Muscle, MUA= Manipulation under anesthesia, MVT= Maximal Voluntary Torque, MWT=Minute walking test NAHS=Non-Arthritic Hip Score, NMES=Neuromuscular Electrical Stimulation, NR=Not reported, OA= Osteoarthritis, OEE= Outcome expectation for exercise, OKS=Oxford Knee Score, Op= Operation, OSI= Overall Stability Index, ODI=Oswestry Disability Index, PE=Pulmonary embolism, PGIC= Patient Global Impression of Change, PCS= Pain catastrophizing score, PSC-Q=Patient specific complaints questionnaire, PSFS=Patient specific Functional Score, PWC-Physical Work Capacity, QALYS-Quality Adjusted Life Years, QOL=Quality of life, RMDQ=Roland Morris Disability Questionnaire, ROM= Range of motion, Rot = Rotation, SEE=Self efficacy to exercises, SF36 = Short Form 36, SPPB= Short physical performance battery, SPW= Self-paced walk, ST= Stair test, THR= Total hip replacement, TKR= Total knee replacement, TUG=Timed up and go, UPST=Unipedal Stance Test, VAS = Visual Analogue Scale, RM=Repeated Measures, ROM=Range of Motion, VAS=Visual Analogue Scale, TUG=Timed up and go test, WOMAC=Western Ontario and McMaster Universities Arthritis Index, 6MWT= 6 Minute Walk Test.

© 2023 Punnoose A et al. *JAMA Network Open*.

eTable 3. Details of Interventions, Dosage, and Compliance Rates

Author, Year	Surgery	Prehab (n)	Control (n)	Type of interventions	Supervision	Dosage (Frequency)	Adherence (%)
An,[1] 2021	TKR	40	20	Strengthening exercises, Stretching, Balance exercises, Patient education	Y	4 weeks (3 times/week)	NR
Beaupre,[2] 2004	TKR	65	66	Strengthening exercises, Patient education	Y	4 weeks (3 times/week)	99
Berge,[3] 2004	THR	19	21	CBT, Patient education	Y	6 weeks (1-2 times/ week)	NR
Bergin,[4] 2014	THR, TKR	50	56	Incentive Spirometry	N	1 week (10 times/day)	NR
Brown,[5] 2012	TKR	17	15	Strengthening exercises, Stretching	C	8 weeks (3 times/week)	NR
Brown,[6] 2014	TKR	17	15	CBT, strengthening exercises, Stretching	C	8 weeks (3times/week)	NR
Calatayud,[7] 2017	TKR	22	22	Strengthening exercises, Balance exercises	Y	8 weeks (3times/week)	NR
Cavill,[8] 2016	THR, TKR	32	32	Strengthening exercises, Mobility, CV exercises	C	3-4 weeks (2times/week)	NR
Crowe,[9] 2003	THR, TKR	65	68	Strengthening exercises, Patient education, counselling	C	Unclear	NR
Doiron-Cadrin,[10] 2019	THR, TKR	23	11	Strengthening exercises, ROM ex, Balance	Y	12 weeks (2 times/week)	73-86

© 2023 Punnoose A et al. *JAMA Network Open*.

Author, Year	Surgery	Prehab (n)	Control (n)	Type of interventions	Supervision	Dosage (Frequency)	Adherence (%)
				exercises, CV exercises			
Dominguez-Navarro,[11] 2020	TKR	56	26	Strengthening exercises, Balance exercises	Y	4 weeks (3 times/week)	100
Evgeniadis,[12] 2008	TKR	40	22	Strengthening exercises	Y	3 weeks (3times/week)	NR
Ferrara,[13] 2008	THR	11	12	Strengthening exercises, Stretching, CV exercises	Y	4 weeks (5 times/week)	NR
Franz,[14] 2022	TKR	20	10	Cycling with BFR	Y	6 weeks (2 times/week)	NR
Gocen,[15] 2004	THR	30	30	Strengthening exercises, Stretching, Patient education	N	8 weeks (3 times/day)	NR
Grant,[16] 2017	FAI	9	9	Strengthening exercises, Patient education	N	8 weeks (1 time/day)	NR
Gstoettner,[17] 2011	TKR	18	20	Balance exercises	N	6 weeks (1 time/day)	NR
Hermann,[18] 2015	THR	40	40	Strengthening exercises	Y	10 weeks (2 times/week)	>80
Hoogeboom,[19] 2010	THR	10	11	Strengthening exercises, CV exercises	C	3-6 weeks (2 times/week)	91
Huang,[20] 2012	TKR	126	117	Home exercise education	N	4 weeks (NR)	NR
Huber,[21] 2015	TKR	22	23	Neuromuscular training exercises, Patient education, CV exercises	Y	4-12 weeks (>8 sessions in total)	76
Jahic,[22] 2018	TKR	10	10	Strengthening exercises, Stretching	N	6 weeks (3 times/day)	NR

© 2023 Punnoose A et al. *JAMA Network Open*.

Author, Year	Surgery	Prehab (n)	Control (n)	Type of interventions	Supervision	Dosage (Frequency)	Adherence (%)
Kim,[23]2021	TKR	20	23	Aquatic exercises- Strengthening exercises, ROM exercises, CV exercises	Y	6 weeks (3 times/week)	NR
Leeuwen,[24]2014	TKR	11	11	Strengthening exercises, Patient education	C	6 weeks (2-3 times/week)	Unclear
Lindback,[25]2017	Lumbar surgery	98	99	Strengthening exercises, Motor control, Traction, CBT	Y	9 weeks (2times/week)	57
Lotzke,[26]2019	Lumbar surgery	59	59	CBT	Y	8-12 weeks (4 x 1 hr)	NR
Marchand,[27]2022	Lumbar surgery	35	33	Strengthening exercises	Y	6 weeks (3times/week)	90
Mat-Eismail,[28]2016	TKR	24	26	Strengthening exercises, CV exercises	Y	6 weeks (1-2 times/week)	100
McKay,[29] 2012	TKR	10	12	Strengthening exercises, CV exercises	Y	6 weeks (3 times/week)	98
Mitchell,[30]2005	TKR	57	57	Strengthening exercises, ROM exercises, Gait re-education	Y	8 weeks (>3 sessions in total)	NR
NCT03044028 [31]	TKR	44	22	NMES	N	4 weeks (3 times/day)	NR
NCT01844934[32]	TKR	9	10	Strengthening exercises	C	4 weeks (3 times/week)	NR
Nielsen,[33]2010	Lumbar surgery	38	35	Strengthening exercises, CV exercises	N	6-8 weeks (1 time/day)	85
Oosting,[34]2012	THR	15	15	Strengthening exercises, Functional exercises	C	3-6 weeks (6 times/ week)	99

© 2023 Punnoose A et al. *JAMA Network Open*.

**Author, Year**	**Surgery**	**Prehab (n)**	**Control (n)**	**Type of interventions**	**Supervision**	**Dosage (Frequency)**	**Adherence (%)**
Riddle,[35]2019	TKR	272	130	CBT, education	C	2 weeks (NR)	73-79
Risso,[36]2022	TKR	9	12	Neuromuscular exercise conditioning	Y	1 week (3times/day x 3times/week)	NR
Rolving,[37]2015	Lumbar surgery	59	31	CBT	Y	4 sessions in total (NR)	>50
Rooks,[38]2006	THR, TKR	54	54	Strengthening exercises, Strethching, Cv exercises (Land +Aquatic)	Y	6 weeks (3 times/week)	89
Savkin,[39]2021	TKR	20	20	NMES	N	6 weeks (5 times/week)	NR
Shaarani,[40]2013	ACLR	11	9	Strengthening exercises, Balance exercises	Y	6 weeks (4 times/week)	90
Skoffer[41], 2016	TKR	30	29	Strengthening exercises	Y	4 weeks (3 times/week)	94
Soni,[42]2012	TKR	28	28	[42], Strengthening exercises, Balance exercises, ROM exercises	Y	6-12 weeks (1time/week to 1 time/month	NR
Swank,[43]2011	TKR	35	36	Strengthening exercises, Stretching	C	4-8 weeks (3 times/ week)	90
Topp,[44]2009	TKR	26	28	Strengthening exercises, Stretching	C	4-8 weeks (3 times/week)	NR
Villadsen,[45]2013	THR, TKR	84	81	Neuromuscular exercise training	Y	8 weeks (2 times/week)	74
Walls,[46]2010	TKR	9	5	NMES	N	8 weeks (1 time/day x 5 days/week)	99
Williamson,[47]2007	TKR	120	61	Acupuncture, strengthening exercises,	Y	6 weeks (1 time/week)	NR

© 2023 Punnoose A et al. *JAMA Network Open*.

Author, Year	Surgery	Prehab (n)	Control (n)	Type of interventions	Supervision	Dosage (Frequency)	Adherence (%)
				Stretching, Balance exercises, ROM exercises			
Zeng,[48] 2014	THR	27	32	Tai-Chi training, Strengthening exercises, ROM exercises	N	12 weeks (5 times/week)	87

ACLR= Anterior cruciate ligament,reconstruction, BFR= Blood Flow Restriction therapy, C=Combination of supervised and unsupervised, CBT= Cognitive behavioural therapy, CV= Cardiovascular, N=No, NR= Not reported, NMES= Neuromuscular electrical stimulation, ROM= Range of motion, TKR= Total Knee replacement, THR= Total Hip replacement, Y= Yes

© 2023 Punnoose A et al. *JAMA Network Open*.

eFigure 1. Risk of Bias for Individual Trials

Unique ID	D1	D2	D3	D4	D5	Overall
Beaupre,2004	+	+	+	+	?	!
Berge,2004	?	+	?	−	?	−
Bergin,2014	−	+	+	−	?	−
Brown,2012	?	+	+	+	?	!
Brown,2014	?	?	+	?	?	!
Calatayud,2017	?	+	+	+	?	!
Cavill,2016	+	+	+	+	+	+
Crowe,2003	?	+	?	+	?	!
Doiron-Cadrin,2019	+	+	+	+	+	+
Dominguez-Navarro,2020	+	+	?	+	+	!
Evgeniadis,2008	+	?	+	−	?	−
Ferrara,2008	?	+	+	+	?	!
Franz,2022	?	+	+	−	?	−
Gocen,2004	−	+	+	+	?	−
Grant,2017	−	+	+	+	+	−
Gstoettner,2011	+	+	+	−	?	−
Hermann,2015	+	+	+	−	+	−
Hoogeboom,2010	+	+	+	+	?	!
Huang,2012	−	+	+	−	?	−
Huber,2015	+	+	−	+	?	−
Jahic,2018	?	?	+	+	?	!
An,2021	+	+	+	+	+	+
Leeuwen,2014	?	+	−	−	?	−
Lindback,2017	+	+	+	?	+	!
Lotzke,2019	+	+	+	+	+	+
McKay2012	?	+	+	−	?	−
Marchand,2022	+	+	+	+	+	+
Mat-Eismail,2016	?	+	−	−	?	−
Mitchell,2005	?	+	+	+	?	!
NCT01844934	−	−	−	−	?	−
NCT03044028	?	−	+	−	?	−
Nielsen,2010	+	+	+	+	+	+
Oosting,2012	?	+	+	+	?	!
Riddle,2019	+	+	+	+	+	+
Risso,2022	−	+	+	−	+	−
Rooks,2006	?	+	+	+	?	!
Rolving,2015	+	?	+	−	?	−
Savkin,2021	?	?	+	−	?	−
Shaarani,2013	+	+	+	−	?	−
Skoffer,2016	+	+	+	+	+	+
Soni,2012	+	+	+	+	+	+
Kim,2021	?	?	+	+	?	!
Swank,2011	?	+	+	−	?	−
Topp,2009	?	?	+	+	?	!
Villadsen,2013	+	+	+	+	+	+
Walls,2010	?	+	+	+	?	!
Williamson,2007	+	+	+	+	?	!
Zeng,2014	?	+	−	+	?	−

+ Low risk
? Some concerns
− High risk

D1 Randomisation process
D2 Deviations from the intended interventions
D3 Missing outcome data
D4 Measurement of the outcome
D5 Selection of the reported result

© 2023 Punnoose A et al. *JAMA Network Open*.

eFigure 2. Risk of Bias Summary Graph

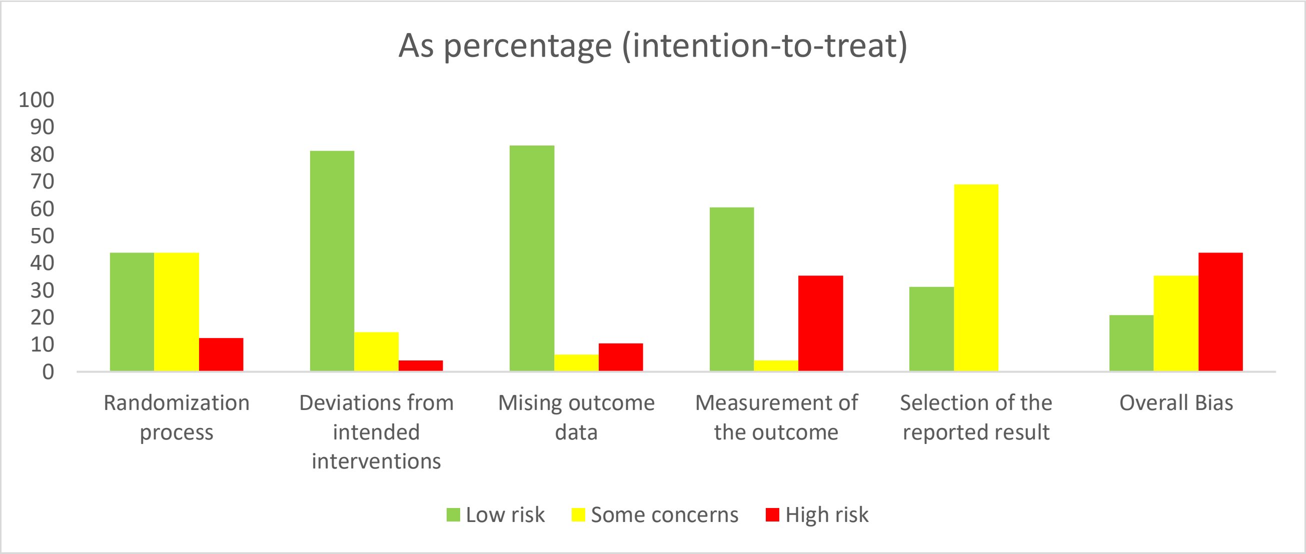

© 2023 Punnoose A et al. *JAMA Network Open*.

eTable 4. GRADE Summary of Findings: TKR

Prehabilitation compared to standard care in patients undergoing Total Knee replacement surgeries

Patient or population: patients undergoing Total Knee replacement surgeries
Setting: All
Intervention: Prehabilitation
Comparison: standard care
Outcome scorings:
High score= better outcome (HRQoL, Muscle strength, Flexion range of motion, 30 sec chair rise test, 6 Minute walk test, Functional reach)
High score= worse outcome (Pain, Anxiety and depression, Function, Extension range of motion, timed up and go test, Stair test)

Outcome № of participants (studies)	Relative effect (95% CI)	Anticipated absolute effects (95% CI) Difference	Certainty	What happens
**PRE-OPERATIVE OUTCOMES**				
Pain at Pre-op № of participants: 1138 (18 RCTs)	Not applicable	SMD **0.58 SD lower** (0.88 lower to 0.28 lower)	Low[a]	Prehabilitation results in moderate reduction in pain at Pre-op but the evidence is uncertain.
Anxiety on HADS at Pre-op № of participants: 161 (2 RCTs)	Not applicable	MD **0.04 lower** (1.39 lower to 1.32 higher)	Low[2,f]	Prehabilitation results in little to no difference in anxiety on HADS at Pre-op.
Depression on HADS at Pre-op № of participants: 161 (2 RCTs)	Not applicable	MD **0.25 lower** (1.35 lower to 0.86 higher)	Low[2,f]	Prehabilitation results in little to no difference in depression on HADS at Pre-op.
Function at pre-op № of participants: 575 (14 RCTs)	Not applicable	SMD **0.7 SD lower** (1.08 lower to 0.32 lower)	Moderate[3,g]	Prehabilitation results in moderate improvement in function at pre-op.
HRQoL on SF-36 at Pre-op № of participants: 206 (5 RCTs)	Not applicable	MD **3.39 higher** (0.65 lower to 7.42 higher)	Very low[4,l]	Prehabilitation may result in little to no effect on HRQoL on SF-36 at Pre-op.

© 2023 Punnoose A et al. *JAMA Network Open*.

eTable 4. GRADE Summary of Findings: TKR

Prehabilitation compared to standard care in patients undergoing Total Knee replacement surgeries

Patient or population: patients undergoing Total Knee replacement surgeries
Setting: All
Intervention: Prehabilitation
Comparison: standard care
Outcome scorings:
High score= better outcome (HRQoL, Muscle strength, Flexion range of motion, 30 sec chair rise test, 6 Minute walk test, Functional reach)
High score= worse outcome (Pain, Anxiety and depression, Function, Extension range of motion, timed up and go test, Stair test)

Outcome № of participants (studies)	Relative effect (95% CI)	Anticipated absolute effects (95% CI) Difference	Certainty	What happens
Knee extensor strength at pre-op № of participants: 682 (13 RCTs)	Not applicable	SMD **0.72 SD higher** (0.28 higher to 1.15 higher)	Low[6,o]	Prehabilitation may result in a moderate increase in knee extensor strength at pre-op, but evidence is uncertain.
Knee flexor strength at pre-op № of participants: 349 (7 RCTs)	Not applicable	SMD **1 SD higher** (0.23 higher to 1.77 higher)	Moderate[p]	Prehabilitation results in large increase in knee flexor strength at pre-op.
Active Knee flexion ROM at pre-op № of participants: 736 (11 RCTs)	Not applicable	MD **4.29 deg higher** (2.02 higher to 6.56 higher)	Low[7,w]	Prehabilitation may result in a slight increase in active knee flexion ROM at pre-op, but evidence is uncertain
Active Knee extension ROM at pre-op № of participants: 331 (8 RCTs)	Not applicable	MD **1.33 deg lower** (3.02 lower to 0.36 higher)	Low[7,x]	Prehabilitation may have little to no effect on active Knee extension ROM at pre-op.

© 2023 Punnoose A et al. *JAMA Network Open*.

eTable 4. GRADE Summary of Findings: TKR

Prehabilitation compared to standard care in patients undergoing Total Knee replacement surgeries

Patient or population: patients undergoing Total Knee replacement surgeries
Setting: All
Intervention: Prehabilitation
Comparison: standard care
Outcome scorings:
High score= better outcome (HRQoL, Muscle strength, Flexion range of motion, 30 sec chair rise test, 6 Minute walk test, Functional reach)
High score= worse outcome (Pain, Anxiety and depression, Function, Extension range of motion, timed up and go test, Stair test)

Outcome № of participants (studies)	Relative effect (95% CI)	Anticipated absolute effects (95% CI)	Certainty	What happens
		**Difference**		
30 sec chair rise test at Pre-op № of participants: 198 (5 RCTs)	Not applicable	MD **0.76 reps higher** (0.16 lower to 1.67 higher)	Low[8,ae]	Prehabilitation may result in little to no difference in 30 sec Sit to Stand Test at Pre-op.
6 Min Walk Test at Pre-op assessed with: meters № of participants: 231 (6 RCTs)	Not applicable	MD **29.2 meters higher** (22.2 higher to 36.2 higher)	Moderate[9,ag]	Prehabilitation results in an improvement in 6 Min Walk Test at Pre-op.
Timed Up and Go at Pre-op № of participants: 302 (7 RCTs)	Not applicable	MD **1.38 sec lower** (2.39 lower to 0.37 lower)	Low[10,aj]	Prehabilitation likely results in a slight improvement in Timed Up and Go at Pre-op but evidence is very uncertian.

© 2023 Punnoose A et al. *JAMA Network Open*.

eTable 4. GRADE Summary of Findings: TKR

Prehabilitation compared to standard care in patients undergoing Total Knee replacement surgeries

Patient or population: patients undergoing Total Knee replacement surgeries
Setting: All
Intervention: Prehabilitation
Comparison: standard care
Outcome scorings:
High score= better outcome (HRQoL, Muscle strength, Flexion range of motion, 30 sec chair rise test, 6 Minute walk test, Functional reach)
High score= worse outcome (Pain, Anxiety and depression, Function, Extension range of motion, timed up and go test, Stair test)

Outcome<br>№ of participants<br>(studies)	Relative effect<br>(95% CI)	Anticipated absolute effects (95% CI)<br>Difference	Certainty	What happens
Stair test at Pre-op<br>№ of participants: 140<br>(5 RCTs)	Not applicable	MD **3.58 sec lower**<br>(5.04 lower to 2.11 lower)	Low[am]	Prehabilitation may reduce the time taken to perform stair test at pre-op but the evidence is very uncertain.
Functional reach at Pre-op assessed with: cms<br>№ of participants: 94<br>(3 RCTs)	Not applicable	MD **1.82 cms higher**<br>(0.72 lower to 4.36 higher)	Low[ap]	Prehabilitation results in little to no difference in functional reach at Pre-op.
**POST-OPERATIVE OUTCOMES**				
Pain at 6 weeks post-op<br>№ of participants: 470<br>(11 RCTs)	Not applicable	SMD **0.23 SD lower**<br>(0.58 lower to 0.12 higher)	Very low[b]	Prehabilitation results in little to no effect on pain at 6 weeks post-op.
Function at 6 weeks post-op<br>№ of participants: 451<br>(10 RCTs)	Not applicable	SMD **0.51 SD lower**<br>(0.85 lower to 0.17 lower)	Moderate[3,h]	Prehabilitation results in moderate improvement in function at 6 weeks post-op.

© 2023 Punnoose A et al. *JAMA Network Open*.

eTable 4. GRADE Summary of Findings: TKR

Prehabilitation compared to standard care in patients undergoing Total Knee replacement surgeries

Patient or population: patients undergoing Total Knee replacement surgeries
Setting: All
Intervention: Prehabilitation
Comparison: standard care
Outcome scorings:
High score= better outcome (HRQoL, Muscle strength, Flexion range of motion, 30 sec chair rise test, 6 Minute walk test, Functional reach)
High score= worse outcome (Pain, Anxiety and depression, Function, Extension range of motion, timed up and go test, Stair test)

Outcome № of participants (studies)	Relative effect (95% CI)	Anticipated absolute effects (95% CI)	Certainty	What happens
		**Difference**		
HRQoL on KOOS at 6 weeks post-op № of participants: 330 (6 RCTs)	Not applicable	MD **5.66 higher** (2.04 higher to 9.27 higher)	Low[5,m]	Prehabilitation results in little to no difference in HRQoL on KOOS at 6 weeks post-op.
Knee extensor strength at 6 weeks post-op № of participants: 327 (8 RCTs)	Not applicable	SMD **0.45 SD higher** (0.06 higher to 0.84 higher)	Low[q]	Prehabilitation may result in an increase knee extensor strength at 6 weeks post-op but the evidence is uncertain.
Knee flexor strength at 6 weeks post-op № of participants: 69 (2 RCTs)	Not applicable	SMD **0.72 SD higher** (0.23 higher to 1.21 higher)	Low[r]	Prehabilitation may result in an increase in Knee flexor strength at 6 weeks post-op, but evidence is very uncertain
Active Knee flexion ROM at 6 weeks post-op № of participants: 260 (5 RCTs)	Not applicable	MD **4.96 deg higher** (2.51 higher to 7.4 higher)	Low[y]	Prehabilitation may result in a slight increase in active Knee flexion ROM at 6 weeks post-op, but the evidence is uncertain
Active Knee extension ROM at 6 weeks post-op № of participants: 157 (3 RCTs)	Not applicable	MD **0.07 deg higher** (1.1 lower to 1.23 higher)	Low[z]	Prehabilitation results in little to no improvement in active knee extension ROM at 6 weeks post-op.

© 2023 Punnoose A et al. *JAMA Network Open*.

eTable 4. GRADE Summary of Findings: TKR

Prehabilitation compared to standard care in patients undergoing Total Knee replacement surgeries

Patient or population: patients undergoing Total Knee replacement surgeries
Setting: All
Intervention: Prehabilitation
Comparison: standard care
Outcome scorings:
High score= better outcome (HRQoL, Muscle strength, Flexion range of motion, 30 sec chair rise test, 6 Minute walk test, Functional reach)
High score= worse outcome (Pain, Anxiety and depression, Function, Extension range of motion, timed up and go test, Stair test)

Outcome № of participants (studies)	Relative effect (95% CI)	Anticipated absolute effects (95% CI)	Certainty	What happens
		Difference		
30 sec Chair rise test at 6 weeks post-op № of participants: 165 (4 RCTs)	Not applicable	MD **1.07 reps higher** (0.14 lower to 2.29 higher)	Very low[8,af]	Prehabilitation results in little to no effect on 30 sec Chair rise test at 6 weeks post-op.
6min walk test at 6 weeks post-op № of participants: 69 (2 RCTs)	Not applicable	MD **1.43 metres higher** (103.41 lower to 106.26 higher)	Very low[ah]	Prehabilitation may have little to no effect on 6min walk test at 6 weeks post-op.
Timed Up and Go at 6 weeks post-op № of participants: 170 (3 RCTs)	Not applicable	MD **1.52 sec lower** (2.22 lower to 0.82 lower)	Low[10,ak]	Prehabilitation results in slight improvement in Timed Up and Go at 6 weeks post-op, but the evidence is uncertain
Stair test at 6 weeks post-op № of participants: 76 (3 RCTs)	Not applicable	MD **0.32 sec higher** (4.02 lower to 4.67 higher)	Low[an]	Prehabilitation may result in little to no difference in stair test time at 6 weeks post-op.
Functional reach at 6 weeks post-op № of participants: 94 (2 RCTs)	Not applicable	MD **0.97 cm higher** (1.54 lower to 3.49 higher)	Low[aq]	Prehabilitation results in little to no difference in functional reach at 6 weeks post-op.

© 2023 Punnoose A et al. *JAMA Network Open*.

eTable 4. GRADE Summary of Findings: TKR

Prehabilitation compared to standard care in patients undergoing Total Knee replacement surgeries

Patient or population: patients undergoing Total Knee replacement surgeries
Setting: All
Intervention: Prehabilitation
Comparison: standard care
Outcome scorings:
High score= better outcome (HRQoL, Muscle strength, Flexion range of motion, 30 sec chair rise test, 6 Minute walk test, Functional reach)
High score= worse outcome (Pain, Anxiety and depression, Function, Extension range of motion, timed up and go test, Stair test)

Outcome № of participants (studies)	Relative effect (95% CI)	**Anticipated absolute effects (95% CI)** **Difference**	Certainty	What happens
Pain at 3 months post-op № of participants: 1304 (18 RCTs)	Not applicable	SMD **0.11 SD lower** (0.29 lower to 0.07 higher)	Very low[c]	Prehabilitation may have little to no effect on pain at 3 months post-op.
Function at 3months post-op № of participants: 1086 (17 RCTs)	Not applicable	SMD **0.29 SD lower** (0.51 lower to 0.08 lower)	Low[i]	Prehabilitation may result in a small improvement in function at 3months post-op, but the evidence is very uncertain.
Anxiety on HADS at 3 months post-op № of participants: 111 (2 RCTs)	Not applicable	MD **0.67 higher** (0.64 lower to 1.98 higher)	Low[2,f]	Prehabilitation results in little to no difference in anxiety on HADS at 3 months post-op.
Depression on HADS at 3 months post-op № of participants: 111 (2 RCTs)	Not applicable	MD **0.59 lower** (1.86 lower to 0.68 higher)	Low[2,f]	Prehabilitation results in little to no difference in depression on HADS at 3 months post-op.
HQoL on SF-36 at 3 months post-op (Higher score=better QoL) № of participants: 177 (4 RCTs)	Not applicable	MD **1.89 higher** (0.64 higher to 3.14 higher)	Low[4,n]	Prehabilitation results in little to no difference in HRQoL on SF-36 at 3 months post-op.

© 2023 Punnoose A et al. *JAMA Network Open*.

eTable 4. GRADE Summary of Findings: TKR

Prehabilitation compared to standard care in patients undergoing Total Knee replacement surgeries

Patient or population: patients undergoing Total Knee replacement surgeries
Setting: All
Intervention: Prehabilitation
Comparison: standard care
Outcome scorings:
High score= better outcome (HRQoL, Muscle strength, Flexion range of motion, 30 sec chair rise test, 6 Minute walk test, Functional reach)
High score= worse outcome (Pain, Anxiety and depression, Function, Extension range of motion, timed up and go test, Stair test)

Outcome № of participants (studies)	Relative effect (95% CI)	Anticipated absolute effects (95% CI)	Certainty	What happens
		Difference		
Knee extensor strength at 3 months post-op № of participants: 402 (10 RCTs)	Not applicable	SMD **0.27 SD higher** (0.04 lower to 0.58 higher)	Low[s]	Prehabilitation may have little to no effect on isometric knee extensor strength at 3 months post-op.
Knee flexor strength at 3 months post-op № of participants: 272 (6 RCTs)	Not applicable	SMD **0.59 SD higher** (0.11 lower to 1.28 higher)	Very low[t]	Prehabilitation may have little or no effect in Knee flexor strength at 3 months post-op.
Active Knee flexion ROM at 3 months post-op № of participants: 460 (10 RCTs)	Not applicable	MD **2.17 deg higher** (0.52 lower to 4.86 higher)	Very low[7,aa]	Prehabilitation results in little to no effect on active Knee flexion ROM at 3 months post-op.
Active Knee extension ROM at 3 months post-op № of participants: 301 (8 RCTs)	Not applicable	MD **1.32 deg lower** (3.16 lower to 0.53 higher)	Very low[7,ab]	Prehabilitation results in little to no effect on active Knee extension ROM at 3 months post-op.
30 sec Sit to Stand at 3 months post-op № of participants: 193 (5 RCTs)	Not applicable	MD **0.98 reps higher** (0.47 lower to 2.43 higher)	Low[8,ae]	Prehabilitation may result in little to no difference in 30 sec Sit to Stand at 3 months post-op.

© 2023 Punnoose A et al. *JAMA Network Open*.

eTable 4. GRADE Summary of Findings: TKR

Prehabilitation compared to standard care in patients undergoing Total Knee replacement surgeries

Patient or population: patients undergoing Total Knee replacement surgeries
Setting: All
Intervention: Prehabilitation
Comparison: standard care
Outcome scorings:
High score= better outcome (HRQoL, Muscle strength, Flexion range of motion, 30 sec chair rise test, 6 Minute walk test, Functional reach)
High score= worse outcome (Pain, Anxiety and depression, Function, Extension range of motion, timed up and go test, Stair test)

Outcome<br>№ of participants<br>(studies)	Relative effect<br>(95% CI)	**Anticipated absolute effects (95% CI)**<br>**Difference**	Certainty	What happens
6 Min Walk Test at 3 months post-op<br>№ of participants: 169<br>(5 RCTs)	Not applicable	MD **17.03 metres higher**<br>(14.21 lower to 48.27 higher)	Low[9,ai]	Prehabilitation results in little to no effect on 6 Min Walk Test at 3 months post-op.
Timed Up and Go at 3 months post-op<br>№ of participants: 161<br>(4 RCTs)	Not applicable	MD **0.67 sec lower**<br>(1.66 lower to 0.32 higher)	Low[10,ak]	Prehabilitation results in little to no difference in timed Up and Go at 3 months post-op.
Stair Test at 3 months post-op assessed with: sec<br>№ of participants: 117<br>(4 RCTs)	Not applicable	MD **3.16 sec lower**<br>(5.05 lower to 1.26 lower)	Low[ao]	Prehabilitation may reduce the time taken to perform Stair test at 3 months post-op but the evidence is very uncertain.
Pain at 6 months post-op (high score= worse pain)<br>№ of participants: 503<br>(4 RCTs)	Not applicable	SMD **0.09 SD lower**<br>(0.27 lower to 0.09 higher)	Low[1,d]	Prehabilitation may have little to no effect on pain at 6 months post-op.
Function at 6 months post-op<br>№ of participants: 523<br>(5 RCTs)	Not applicable	SMD **0.01 SD higher**<br>(0.35 lower to 0.37 higher)	Very low[j]	Prehabilitation may have little to no effect on function at 6 months post-op.

© 2023 Punnoose A et al. *JAMA Network Open*.

eTable 4. GRADE Summary of Findings: TKR

Prehabilitation compared to standard care in patients undergoing Total Knee replacement surgeries

Patient or population: patients undergoing Total Knee replacement surgeries
Setting: All
Intervention: Prehabilitation
Comparison: standard care
Outcome scorings:
High score= better outcome (HRQoL, Muscle strength, Flexion range of motion, 30 sec chair rise test, 6 Minute walk test, Functional reach)
High score= worse outcome (Pain, Anxiety and depression, Function, Extension range of motion, timed up and go test, Stair test)

Outcome № of participants (studies)	Relative effect (95% CI)	Anticipated absolute effects (95% CI) Difference	Certainty	What happens
Pain at 1 year post-op № of participants: 600 (5 RCTs)	Not applicable	SMD **0.04 SD lower** (0.21 lower to 0.12 higher)	Low[e]	Prehabilitation results in little to no difference in pain at 1 year post-op.
Function at 1 year post-op № of participants: 620 (6 RCTs)	Not applicable	SMD **0.03 SD higher** (0.13 lower to 0.2 higher)	Low[k]	Prehabilitation results in little to no difference in function at 1 year post-op.
Knee extensor strength at 1 yr post-op № of participants: 203 (3 RCTs)	Not applicable	SMD **0.22 higher** (0.06 lower to 0.5 higher)	Low[u]	Prehabilitation results in little to no difference in knee extensor strength at 1 yr post-op.
Knee flexor strength at 1 year post-op № of participants: 153 (2 RCTs)	Not applicable	SMD **0.12 higher** (0.23 lower to 0.48 higher)	Low[v]	Prehabilitation results in little to no increase in knee flexor strength at 1 year post-op.
Active knee flexion ROM at 1 year post-op № of participants: 203 (3 RCTs)	Not applicable	MD **0.34 deg lower** (3.97 lower to 3.3 higher)	Very low[7,ac]	Prehabilitation results in little to no difference in active knee flexion ROM at 1 year post-op.

© 2023 Punnoose A et al. *JAMA Network Open*.

eTable 4. GRADE Summary of Findings: TKR

Prehabilitation compared to standard care in patients undergoing Total Knee replacement surgeries

Patient or population: patients undergoing Total Knee replacement surgeries
Setting: All
Intervention: Prehabilitation
Comparison: standard care
Outcome scorings:
High score= better outcome (HRQoL, Muscle strength, Flexion range of motion, 30 sec chair rise test, 6 Minute walk test, Functional reach)
High score= worse outcome (Pain, Anxiety and depression, Function, Extension range of motion, timed up and go test, Stair test)

Outcome № of participants (studies)	Relative effect (95% CI)	Anticipated absolute effects (95% CI)	Certainty	What happens
		**Difference**		
Active knee extension ROM at 1 year post-op № of participants: 94 (2 RCTs)	Not applicable	MD **0.75 deg higher** (1.03 lower to 2.54 higher)	Very low[7,ad]	Prehabilitation results in little to no improvement in active knee extension ROM at 1 year post-op.
6 Min walk test at 1 year post-op № of participants: 390 (2 RCTs)	Not applicable	MD **7.24 metres lower** (32.39 lower to 17.92 higher)	Low[9,ah]	Prehabilitation results in little to no difference in 6 Min walk test at 1 year post-op.
Timed Up and Go test at 1 year post-op № of participants: 94 (3 RCTs)	Not applicable	MD **0.8 sec lower** (2.31 lower to 0.7 higher)	Low[10,al]	Prehabilitation results in little to no difference in Timed Up and Go test at 1 year post-op.
Length of stay № of participants: 713 (6 RCTs)	Not applicable	MD **0.16 days lower** (0.74 lower to 0.41 higher)	Low[ar]	Prehabilitation results in little to no difference in length of stay after Total Knee replacement
Complication rates № of participants: 1003 (10 RCTs)	**RR 1.10** (0.76 to 1.61)	**0.9% more** (2.3 fewer to 5.8 more)	Very low[as]	Prehabilitation may increase complication rates but the evidence is very uncertain.

© 2023 Punnoose A et al. *JAMA Network Open*.

eTable 4. GRADE Summary of Findings: TKR

Prehabilitation compared to standard care in patients undergoing Total Knee replacement surgeries

Patient or population: patients undergoing Total Knee replacement surgeries
Setting: All
Intervention: Prehabilitation
Comparison: standard care
Outcome scorings:
High score= better outcome (HRQoL, Muscle strength, Flexion range of motion, 30 sec chair rise test, 6 Minute walk test, Functional reach)
High score= worse outcome (Pain, Anxiety and depression, Function, Extension range of motion, timed up and go test, Stair test)

Outcome № of participants (studies)	Relative effect (95% CI)	Anticipated absolute effects (95% CI)	Certainty	What happens
		Difference		
Healthcare costs № of participants: 466 (3 RCTs)	Not applicable	SMD **0 SD** (0.19 lower to 0.18 higher)	Low[at]	Prehabilitation does not have any effect on healthcare costs.
Readmission rates № of participants: 544 (3 RCTs)	**RR 0.83** (0.38 to 1.79)	**0.9% fewer** (3.2 fewer to 4 more)	Low[au]	Prehabilitation may result in little to no difference in readmission rates.

***The risk in the intervention group** (and its 95% confidence interval) is based on the assumed risk in the comparison group and the **relative effect** of the intervention (and its 95% CI).

CI: confidence interval; **MD:** mean difference; **RR:** risk ratio; **SMD:** standardised mean difference

GRADE Working Group grades of evidence
High certainty: we are very confident that the true effect lies close to that of the estimate of the effect.
Moderate certainty: we are moderately confident in the effect estimate: the true effect is likely to be close to the estimate of the effect, but there is a possibility that it is substantially different.
Low certainty: our confidence in the effect estimate is limited: the true effect may be substantially different from the estimate of the effect.
Very low certainty: we have very little confidence in the effect estimate: the true effect is likely to be substantially different from the estimate of effect.

Explanations

a. Downgraded as 14/18 studies showed moderate to high ROB. Downgraded as showing substantial level of heterogeneity.
b. Downgraded as 8 out of 11 studies showed moderate to high ROB. Downgraded as showing substantial heterogeneity and 95% CI crossing the line of no effect.
c. Downgraded as 14/18 studies showed moderate to high ROB. Downgraded as showing substantial level of heterogeneity and 95% CI crossing the line of no effect.
d. Downgraded as 3/4 studies showed moderate ROB. Downgraded as 95% CI crossing the line of no effect.
e. Downgraded as 2 out of 5 studies showed moderate and 1 study showed high ROB. Downgraded as 95% CI crosses the line of no effect.
f. Downgraded by 1 level as 1 out of 2 studies showed moderate ROB (selective reporting). Downgraded for imprecision as small sample size and 95% CI crosses the line of no effect. MD does not meet the MCID for HADS.
g. Downgraded as 11 out of 14 studies showed moderate to high ROB. Fair amount of CI overlap noted and therefore not downgraded for inconsistency.
h. Downgraded as 5 studies showed high ROB and 4 showed moderate ROB out of a total of 10 studies. Fair amount go overlap in CI's and therefore not downgraded for heterogeneity
i. Downgraded as 11/15 studies showed moderate to high ROB. Downgraded as showing substantial heterogeneity and 95% CI crosses the line of no effect. Publication bias suspected on funnel plot

© 2023 Punnoose A et al. *JAMA Network Open*.

j. Downgraded as 3/5 studies showed moderate ROB and 1 study showed high ROB. Downgraded as showing high heterogeneity and 95% CI crossing the line of no effect.
k. Downgraded as 4 out of six studies showed moderate to high ROB. Downgraded for imprecision as 95% CI crosses the line of no effect.
l. Downgraded as 3 studies showed moderate ROB and 1 showed high ROB. Downgraded as showing substantial heterogeneity and 95% CI crosses line of no effect. MD does not meet the MCID of 5 points
m. Downgraded as 4 out of 6 studies showed moderate to high ROB. Downgraded for imprecision as small sample size. MD does not meet the MCID of 15 points for KOOS
n. Downgraded as 3 out of 4 studies showed moderate ROB. Downgraded for imprecision as small sample size (<400). MD not meeting the MCID of 5 points
o. Downgraded as 10/13 studies showed moderate to high ROB (allocation concealment, missing outcome data, assessor blinding, selective reporting). Downgraded as substantial heterogeneity.
p. Downgraded as 5/7 studies showed moderate to high ROB(allocation concealment, missing outcome data, assessor blinding, possible selective reporting). Downgraded for substantial heterogeneity and small sample size. Upgraded for very large effect size.
q. Downgraded as 4 out of 8 studies showed high ROB and 2 showed moderate. Downgraded for imprecision as small sample size (<400).
r. Downgraded as 1 out of 2 studies showed moderate ROB. Downgraded for imprecision as small sample size.
s. Downgraded as 8 out of 10 studies showed moderate to high ROB (allocation concealment, missing outcome data, assessor blinding, possible selective reporting). Downgraded as 95% CI crosses the line of no effect.
t. Downgraded as 5 out of 6 studies showed moderate to high ROB (allocation concealment, missing outcome data, assessor blinding, possible selective reporting). Downgraded for substantial heterogeneity and 95% CI crosses the line of no effect.
u. Downgraded as 2 out of 3 studies showed moderate ROB (allocation concealment, possible selective reporting). Downgraded as 95% CI crosses the line of no effect.
v. Downgraded as 1 out of 2 studies showed moderate ROB (selective reporting). Downgraded as 95% CI crosses the line of no effect.
w. Downgraded as 9/11 studies showed moderate to high ROB. Moderate level of heterogeneity and therefore downgraded for heterogeneity. MD does not meet the MCID of 9.6 deg for knee flexion
x. Downgraded as 7/8 studies showed moderate to high ROB(allocation concealment, assessor blinding, missing outcome data, selective reporting). Moderate level of heterogeneity noted, however, fair amount of CI overlap seen, therefore not downgraded for inconsistency. Downgraded as 95% CI crossing the line of no effect. MD does not meet the MCID of 6.9 deg for knee extension.
y. Downgraded as 4 out of 5 studies showed moderate to high ROB. Downgraded for imprecision as small sample size <400. MD does not meet the MCID of 9.6 deg for knee flexion
z. Downgraded as 1 out of 3 studies showed moderate ROB and 1 showed high ROB. Downgraded for imprecision as small sample size.MD does not meet the MCID of 5 points.
aa. Downgraded as 6 out of 10 studies showed high ROB and 2 moderate ROB. Downgraded as showing moderate heterogeneity and 95% CI crossing the line of no effect. MD does not meet the MCID for knee flexion
ab. Downgraded as 4 out of 6 studies showed moderate to high ROB. Downgraded as showing substantial heterogeneity and 95% CI crossing the line of no effect. MD does not meet the MCID for knee extension
ac. Downgraded as 2 out of 3 studies showed moderate ROB. Downgraded for imprecision as 95% CI crosses the line of no effect.MD does not meet the MCID fo knee flexion
ad. Downgraded as one of the two studies showed moderate ROB. Downgraded as showed moderate heterogeneity and 95% CI crossing the line of no effect. MD does not meet the MCID for knee extension
ae. Downgraded as 2 out of 3 studies showed moderate to high ROB. Downgraded for imprecision as small sample size. MD does not meet the MCID of 2.0- 2.6 repetitions.
af. Downgraded as 2 showed high ROB and 1 study showed moderate ROB. Downgraded for moderate heterogeneity and small sample size. The MD does not meet the mCID of 2.0-2.6
ag. Downgraded as 5 out of 6 studies showed moderate to high ROB. Downgraded for small sample size. MD meets the MCID of 20 metres
ah. Downgraded 2 levels for imprecision as 95% CI crosses the line of no effect and is shown to be extremely wide. MD does not meet the MCID of 20 metres
ai. Downgraded as 4 out of 5 studies showed moderate to high ROB. Downgraded for imprecision as small sample size and 95% CI crosses the line of no effect. MD does not meet the MCID of 20 metres.
aj. Downgraded one level as 5 out of 7 studies showed moderate to high ROB. Downgraded for imprecision as small sample size. MD does not meet the MCID of 3.4 sec
ak. Downgraded as 2 out of 4 studies showed moderate to high ROB. Downgraded for imprecision as 95% CI crosses the line of no effect. MD does not meet the MCID of 3.4 sec
al. Downgraded as 1 out 2 studies showed moderate ROB. Downgraded for imprecision as the 95% CI crosses the line of no effect. MD does not meet the MCID 3.4 sec
am. Downgraded as 4 out of 5 studies showed moderate to high ROB. No information of the MCID for Stair test available in literature.
an. Downgraded by 2 levels as both studies showed high ROB. Downgraded for imprecision as 95% CI crosses the line of no effect
ao. Downgraded as 3 out of 4 studies showed high ROB and 1 study showed moderate ROB. Downgraded for small sample size. No information on MCID for stair test available in the literature.
ap. Downgraded as both studies showed moderate ROB and 95% CI crosses the line of no effect.
aq. Downgraded as both studies showed moderate ROB. Downgraded for imprecision as small sample size
ar. Downgraded as all studies showed moderate to high ROB(allocation concealment, blinding of the outcome assessors and possible selective reporting). Downgraded for high level of heterogeneity and 95% CI crossing the line of no effect.
as. Downgraded by 2 levels as 6/10 studies showed high ROB and 3/10 studies showed moderate ROB (allocation concealment, blinding of the outcome assessor and possible selective reporting). Downgraded for imprecision as the 95% CI crosses the line of no effect.
at. Downgraded as all 3 studies showed moderate or high ROB. Downgraded for imprecision as 95% CI crossed the line of no effect.
au. Downgraded as 2/3 studies showed moderate to high ROB. Downgraded for imprecision as 95% CI crossed the line of no effect

References

1.Man Soo Kim, In Jun Koh,Keun Young Choi,Yong Gyu Sung,Dong Chul Park,Hyo Jin Lee,Yong In. The Minimal Clinically Important Difference (MCID) for the WOMAC and Factors Related to Achievement of the MCID After Medial Opening Wedge High Tibial Osteotomy for Knee Osteoarthritis. American Journal of sports medicine; 2021.
2.Milo A Puhan, Martin Frey,Stefan Büchi and Holger J Schünemann. The minimal important difference of the hospital anxiety and depression scale in patients with chronic obstructive pulmonary disease. Health and Quality of Life Outcomes; 2008.

© 2023 Punnoose A et al. *JAMA Network Open*.

3.Guyatt GH, Oxman AD,Kunz R,Woodcock J,Brozek J,Helfand M,Alonso-Coello P,Glasziou P,Jaeschke R,Akl EA,Norris S,Vist G,Dahm P,Shukla VK,Higgins J,Falck-Ytter Y,Schünemann HJ, Group., GRADE,Working. GRADE guidelines: 7. Rating the quality of evidence--inconsistency. 2011.
4.Koichi Ogura, Mohamed A. Yakoub,Alexander B. Christ,Tomohiro Fujiwara,Zarko Nikolic,Patrick J. Boland,John H. Healey. What Are the Minimum Clinically Important Differences in SF-36 Scores in Patients with Orthopaedic Oncologic Conditions?. Clinical orthopaedics and related research; 2020.
5.Man Hung, Jerry Bounsanga,Maren W Voss,and Charles L Saltzman. Establishing minimum clinically important difference values for the Patient-Reported Outcomes Measurement Information System Physical Function, hip disability and osteoarthritis outcome score for joint reconstruction, and knee injury and osteoarthritis outcome score for joint reconstruction in orthopaedics. 2018.
6.Cohen, J. Statistical Power Analysis for the Behavioral Sciences (2nd ed.). Hillsdale, NJ: Lawrence Erlbaum Associates, Publishers.; 1988.
7.Stratford, P. W.,Kennedy,D. M. & Robarts,S. F. Modelling knee range of motion post arthroplasty: Clinical applications. Physiotherapy Canada; 2010.
8.Alexis A Wright, Chad E Cook,G David Baxter,John D Dockerty,J Haxby Abbott. A comparison of 3 methodological approaches to defining major clinically important improvement of 4 performance measures in patients with hip osteoarthritis. JOSPT; 2011.
9.Bennell K, Dobson F. Measures of physical performance assessments. Patient outcomes in rheumatology, A review of measures. Arth Care Res.2011;63(11):S350-S370.; 2011.
10.Oliver P Gautschi, Martin N Stienen,Marco V Corniola,Holger Joswig,Karl Schaller,Gerhard Hildebrandt,Nicolas R Smoll. Assessment of the Minimum Clinically Important Difference in the Timed Up and Go Test After Surgery for Lumbar Degenerative Disc Disease. Neurosurgery; 2017.

© 2023 Punnoose A et al. *JAMA Network Open*.

eTable 5. GRADE Summary of Findings: THR

Prehabilitation compared to standard care in patients undergoing Total Hip replacement surgery

Patient or population: patients undergoing Total Hip replacement surgery
Setting: All
Intervention: Prehabilitation
Comparison: standard care
Outcome scorings:
High score= better outcome (HRQoL, Function on HOOS, Pain on HOOS, Muscle strength, Flexion range of motion, 30 sec chair rise test, 6 Minute walk test, Functional reach)
High score= worse outcome (Pain, Anxiety and depression, Function, Extension range of motion, timed up and go test, Stair test)

Outcome № of participants (studies)	Relative effect (95% CI)	Anticipated absolute effects (95% CI)<br>Difference	Certainty	What happens
**PRE-OPERATIVE OUTCOMES**				
Pain at Pre-op № of participants: 340 (8 RCTs)	Not applicable	SMD **0.47 SD lower** (0.69 lower to 0.25 lower)	Low[1,a]	Prehabilitation may reduce pain pre-operatively but the evidence is very uncertain.
Function at Pre-op № of participants: 359 (8 RCTs)	Not applicable	SMD **0.54 SD lower** (0.81 lower to 0.28 lower)	Low[e]	Prehabilitation may improve function moderately at Pre-op but the evidence is very uncertain.
HRQoL at Pre-op on SF-36 (High score=better QoL) № of participants: 40 (2 RCTs)	Not applicable	MD **7.35 higher** (3.15 higher to 11.54 higher)	Moderate[3,i]	Prehabilitation results in large improvement in HRQoL at Pre-op.
Ms strength-Hip Abductor at Pre-op № of participants: 107 (2 RCTs)	Not applicable	SMD **1.03 higher** (0.03 higher to 2.02 higher)	Moderate[1,k]	Prehabilitation likely results in a large increase in Hip abductor strength at Pre-op.
Ms strength-Hip extensor at Pre-op № of participants: 163 (2 RCTs)	Not applicable	SMD **0.26 higher** (0.27 lower to 0.8 higher)	Very low[l]	Prehabilitation may have little to no effect on hip extensor muscle strength at Pre-op.

© 2023 Punnoose A et al. *JAMA Network Open*.

eTable 5. GRADE Summary of Findings: THR

Prehabilitation compared to standard care in patients undergoing Total Hip replacement surgery

Patient or population: patients undergoing Total Hip replacement surgery
Setting: All
Intervention: Prehabilitation
Comparison: standard care
Outcome scorings:
High score= better outcome (HRQoL, Function on HOOS, Pain on HOOS, Muscle strength, Flexion range of motion, 30 sec chair rise test, 6 Minute walk test, Functional reach)
High score= worse outcome (Pain, Anxiety and depression, Function, Extension range of motion, timed up and go test, Stair test)

Outcome № of participants (studies)	Relative effect (95% CI)	Anticipated absolute effects (95% CI)<br>Difference	Certainty	What happens
Timed Up and Go at Pre-op (High score= worse outcome) № of participants: 201 (5 RCTs)	Not applicable	MD **2.01 sec lower** (4.49 lower to 0.47 higher)	Very low[4,m]	Prehabilitation results in little to no effect on Timed Up and Go at Pre-op.
6 Min Walk Test at Pre-op (High score= better outcome) № of participants: 135 (3 RCTs)	Not applicable	MD **40.65 meters higher** (-1.01 higher to 82.32 higher)	Low[5,o]	Prehabilitation results in an improvement in 6 Min Walk Test at Pre-op.
**POST-OPERATIVE OUTCOMES**				
Pain on HOOS at 6 weeks post-op № of participants: 110 (2 RCTs)	Not applicable	MD **1.82 higher** (2.61 lower to 6.43 higher)	Low[2,b]	Prehabilitation results in little to no difference in pain on HOOS at 6 weeks post-op.
Function on HOOS at 6 weeks post-op (High score=better outcome) № of participants: 110 (2 RCTs)	Not applicable	MD **1.46 higher** (5.08 lower to 7.99 higher)	Low[2,b]	Prehabilitation may have little to no effect on function at 6 weeks post-op.

© 2023 Punnoose A et al. *JAMA Network Open*.

eTable 5. GRADE Summary of Findings: THR

Prehabilitation compared to standard care in patients undergoing Total Hip replacement surgery

Patient or population: patients undergoing Total Hip replacement surgery
Setting: All
Intervention: Prehabilitation
Comparison: standard care
Outcome scorings:
High score= better outcome (HRQoL, Function on HOOS, Pain on HOOS, Muscle strength, Flexion range of motion, 30 sec chair rise test, 6 Minute walk test, Functional reach)
High score= worse outcome (Pain, Anxiety and depression, Function, Extension range of motion, timed up and go test, Stair test)

Outcome № of participants (studies)	Relative effect (95% CI)	Anticipated absolute effects (95% CI) Difference	Certainty	What happens
HRQoL on HOOS at 6 weeks post-op (High score=better outcome) № of participants: 109 (2 RCTs)	Not applicable	MD **5.5 higher** (0.55 lower to 11.54 higher)	Low[2,b]	Prehabilitation results in little to no difference in HQoL at 6 weeks post-op.
Timed Up and Go at 6 weeks post-op № of participants: 75 (2 RCTs)	Not applicable	MD **0.58 higher** (0.86 lower to 2.03 higher)	Low[n]	Prehabilitation may result in little to no difference in timed Up and Go at Pre-op - TUG at 6 weeks post-op.
Pain at 3 months post-op № of participants: 146 (3 RCTs)	Not applicable	SMD **0.17 SD lower** (0.5 lower to 0.15 higher)	Low[c]	Prehabilitation results in little to no difference in pain at 3 months post-op.
Function at 3 months post-op № of participants: 310 (6 RCTs)	Not applicable	SMD **0.38 SD lower** (0.61 lower to 0.16 lower)	Low[f]	Prehabilitation may result in small improvement in function at 3 months post-op, but evidence is uncertain.
HRQoL at 3 months post-op № of participants: 171 (3 RCTs)	Not applicable	SMD **0.21 SD higher** (0.09 lower to 0.51 higher)	Low[1,j]	Prehabilitation likely results in little to no difference in hQoL at 3 months post-op.
Pain at 6 months post-op № of participants: 115 (2 RCTs)	Not applicable	SMD **0.16 SD lower** (0.53 lower to 0.21 higher)	Low[d]	Prehabilitation results in little to no effect on pain at 6 months post-op.

© 2023 Punnoose A et al. *JAMA Network Open*.

eTable 5. GRADE Summary of Findings: THR

Prehabilitation compared to standard care in patients undergoing Total Hip replacement surgery

Patient or population: patients undergoing Total Hip replacement surgery
Setting: All
Intervention: Prehabilitation
Comparison: standard care
Outcome scorings:
High score= better outcome (HRQoL, Function on HOOS, Pain on HOOS, Muscle strength, Flexion range of motion, 30 sec chair rise test, 6 Minute walk test, Functional reach)
High score= worse outcome (Pain, Anxiety and depression, Function, Extension range of motion, timed up and go test, Stair test)

Outcome № of participants (studies)	Relative effect (95% CI)	Anticipated absolute effects (95% CI)	Certainty	What happens
		**Difference**		
Function at 6 months post-op № of participants: 115 (2 RCTs)	Not applicable	SMD **0.15 lower** (0.52 lower to 0.22 higher)	Low[g]	Prehabilitation results in little to no difference in function at 6 months post-op.
Function at 12 months post-op № of participants: 160 (3 RCTs)	Not applicable	SMD **0.34 SD lower** (0.65 lower to 0.02 lower)	Low[h]	Prehabilitation may result in a small improvement function s at 12 months post-op but the evidence is very uncertain.
Length of Stay assessed with: days № of participants: 66 (3 RCTs)	Not applicable	MD **0.16 days higher** (0.89 lower to 1.21 higher)	Very low[p]	Prehabilitation may have little to no effect on length of stay after Total Hip replacement surgery
Complication rates № of participants: 156 (4 RCTs)	**RR 0.49** (0.18 to 1.17)	**10.6% fewer** (17 fewer to 3.5 more)	Low[q]	Prehabilitation results in little to no difference in complication rates.

***The risk in the intervention group** (and its 95% confidence interval) is based on the assumed risk in the comparison group and the **relative effect** of the intervention (and its 95% CI).

CI: confidence interval; **MD:** mean difference; **RR:** risk ratio; **SMD:** standardised mean difference

GRADE Working Group grades of evidence
High certainty: we are very confident that the true effect lies close to that of the estimate of the effect.
Moderate certainty: we are moderately confident in the effect estimate: the true effect is likely to be close to the estimate of the effect, but there is a possibility that it is substantially different.
Low certainty: our confidence in the effect estimate is limited: the true effect may be substantially different from the estimate of the effect.
Very low certainty: we have very little confidence in the effect estimate: the true effect is likely to be substantially different from the estimate of effect.

Explanations

a. Downgraded due to moderate to high risk in 7/8 studies (blinding, missing outcome data, selective reporting). Downgraded for small sample size (<400).

© 2023 Punnoose A et al. *JAMA Network Open*.

b. Downgraded as 1 out of 2 studies showed moderate risk of bias(allocation concealment, selective reporting). Downgraded for imprecision as 95% CI crosses the line of no effect. MD does not meet the MCID for HOOS
c. All 3 studies showed moderate to high ROB (allocation concealment, blinding, selective reporting). Downgraded for imprecision due to small sample size (<400).
d. Downgraded as both studies showed moderate to high ROB (allocation concealment, blinding of the assessor). Downgraded due to moderate heterogeneity and small sample size (<400).
e. Downgraded due to moderate to high risk in 7/8 studies (blinding, missing outcome data, selective reporting). Downgraded for imprecision as small sample size (<400).
f. 4 out of 6 studies showed moderate to high ROB (allocation concealment, blinding of the assessor, selective reporting). Downgraded for substantial heterogeneity and small sample size (<400).
g. Downgraded as both studies showed moderate to high ROB. Downgraded for imprecision as small sample size (<400).
h. All 3 studies showed high ROB (allocation concealment, blinding of the assessor, missing outcome data). Downgraded for small sample size (<400).
i. Downgraded as one study showed moderate ROB (allocation concealment, selective reporting). Downgraded for imprecision as small sample size (<400). MD meets the MCID for SF-36 of 5 points.
j. Downgraded as 1 out of 3 studies showed high ROB. Downgraded for small sample size (<400) and 95% CI crossing the line of no effect.
k. Downgraded as 1 study showed moderate ROB (Possible allocation concealment and selective reporting). Downgraded due to moderate level of heterogeneity and small sample size (<400). Upgraded for very large effect size (SMD>1.0).
l. Downgraded as 1 study showed high ROB (blinding of assessor, elective reporting). Downgraded for moderate heterogeneity and small number of participants.
m. Downgraded as 4 out of 5 paper showed moderate to high ROB (allocation concealment, selective reporting). Substantial heterogeneity noted and small sample size(<400). The MD does not meet MCID
n. Downgraded as both studies showed moderate RoB. Downgraded for small number of participants and 95% CI crossing the line of no effect.
o. Downgraded as all studies showed moderate to high ROB (allocation concealment, selective reporting) and small sample size (<400).
p. Downgraded as 4 out of 5 studies showed moderate ROB (allocation concealment, possible selective reporting). Downgraded for substantial heterogeneity, small sample size and 95% CI crosses the line of no effect.
q. Downgraded as all studies showed moderate ROB (allocation concealment, possible selective reporting). Downgraded for small sample size and 95% CI crossing the line of no effect.

References

1.Cohen, J. Statistical Power Analysis for the Behavioral Sciences (2nd ed.). Hillsdale, NJ: Lawrence Erlbaum Associates, Publishers.; 1988.
2.Stephen Lyman, Yuo-Yu Lee,Alexander S McLawhorn,Wasif Islam,Catherine H MacLean. What Are the Minimal and Substantial Improvements in the HOOS and KOOS and JR Versions After Total Joint Replacement?. 2018.
3.Koichi Ogura, Mohamed A. Yakoub,Alexander B. Christ,Tomohiro Fujiwara,Zarko Nikolic,Patrick J. Boland,John H. Healey. What Are the Minimum Clinically Important Differences in SF-36 Scores in Patients with Orthopaedic Oncologic Conditions?. Clinical orthopaedics and related research; 2020.
4.Oliver P Gautschi, Martin N Stienen,Marco V Corniola,Holger Joswig,Karl Schaller,Gerhard Hildebrandt,Nicolas R Smoll. Assessment of the Minimum Clinically Important Difference in the Timed Up and Go Test After Surgery for Lumbar Degenerative Disc Disease. Neurosurgery; 2017.
5.Bennell K, Dobson F. Measures of physical performance assessments. Patient outcomes in rheumatology, A review of measures. Arth Care Res.2011;63(11):S350-S370.; 2011.

eTable 6. GRADE Summary of Findings: Lumbar Surgery

Prehabilitation compared to standard care in patients undergoing Lumbar surgery

Patient or population: Patients undergoing Lumbar surgery
Setting: All
Intervention: Prehabilitation
Comparison: standard care

Outcome № of participants (studies)	Relative effect (95% CI)	Anticipated absolute effects (95% CI) Difference	Certainty	What happens
**PRE-OPERATIVE OUTCOMES**				
Back pain pre-operatively (High score=worse pain) № of participants: 402 (4 RCTs)	Not applicable	- MD **8.2 lower** (8.85 lower to 7.55 lower)	High[1,a]	Prehabilitation likely results in a slight reduction in back pain pre-operatively.
Leg pain pre-operatively (High score= worse pain) № of participants: 401 (4 RCTs)	Not applicable	MD **4.23 lower** (10.14 lower to 1.67 higher)	Low[1,e]	Prehabilitation may have little to no effect on leg pain pre-operatively.
Depression at Pre-op (High score=worse outcome) № of participants: 217 (2 RCTs)	Not applicable	SMD **0.58 SD lower** (1.28 lower to 0.12 higher)	Very low[h]	Prehabilitation may have little to no effect on depression at pre-op.
Function pre-operatively (High score=poor function) № of participants: 391 (4 RCTs)	Not applicable	SMD **0.74 SD lower** (1.11 lower to 0.69 lower)	Low[j]	Prehabilitation may result in moderate improvement in function pre-operatively, but the evidence is uncertain.

© 2023 Punnoose A et al. *JAMA Network Open*.

eTable 6. GRADE Summary of Findings: Lumbar Surgery

Prehabilitation compared to standard care in patients undergoing Lumbar surgery

Patient or population: Patients undergoing Lumbar surgery
Setting: All
Intervention: Prehabilitation
Comparison: standard care

Outcome<br>№ of participants (studies)	Relative effect (95% CI)	Anticipated absolute effects (95% CI)<br>Difference	Certainty	What happens
HRQoL at pre-op (High score= better outcome)<br>№ of participants: 149<br>(2 RCTs)	Not applicable	SMD **0.46 SD higher**<br>(0.13 higher to 0.78 higher)	Moderate[n]	The evidence suggests prehabilitation results in moderate improvement in HRQoL at pre-op, but the evidence is uncertain.
**POST-OPERATIVE OUTCOMES**				
Function at 6 weeks post-op<br>№ of participants: 132<br>(2 RCTs)	Not applicable	SMD **0.41 SD lower**<br>(1.07 lower to 0.24 higher)	Low[k]	Prehabilitation may result in little to no difference in function at 6 weeks post-op.
Back pain at 3 months post-op<br>№ of participants: 280<br>(4 RCTs)	Not applicable	MD **5.93 lower**<br>(10.55 lower to 1.31 lower)	Low[1,b]	Prehabilitation may result in a slight reduction in back pain at 3 months post-op, but the evidence is uncertain
Leg pain at 3 months post-op<br>№ of participants: 279<br>(4 RCTs)	Not applicable	MD **1.83 lower**<br>(9.71 lower to 6.04 higher)	Very low[2,c]	Prehabilitation may have little to no effect on leg pain at 3 months post-op.
Depression at 3 month post-op<br>№ of participants: 211<br>(2 RCTs)	Not applicable	SMD **0.02 SD lower**<br>(0.35 lower to 0.32 higher)	Low[i]	Prehabilitation may have little to no effect on depression at 3 months post-op.
Function at 3 months post-op<br>№ of participants: 448<br>(5 RCTs)	Not applicable	SMD **0.94 SD lower**<br>(2.07 lower to 0.18 higher)	Low[l]	Prehabilitation may result in a large improvement in function at 3 months post-op, but the evidence is uncertain

© 2023 Punnoose A et al. *JAMA Network Open*.

eTable 6. GRADE Summary of Findings: Lumbar Surgery

Prehabilitation compared to standard care in patients undergoing Lumbar surgery

Patient or population: Patients undergoing Lumbar surgery
Setting: All
Intervention: Prehabilitation
Comparison: standard care

Outcome № of participants (studies)	Relative effect (95% CI)	**Anticipated absolute effects (95% CI)**	Certainty	What happens
		**Difference**		
HRQoL at 3 months post-op (High score=better QoL) № of participants: 337 (3 RCTs)	Not applicable	SMD **0.11 SD higher** (0.11 lower to 0.32 higher)	Low[o]	Prehabilitation results in little to no difference in HRQoL at 3 months post-op.
Back pain at 6 months post-op № of participants: 276 (4 RCTs)	Not applicable	MD **3.27 lower** (7.89 lower to 1.35 higher)	Low[c]	Prehabilitation results in little to no difference in back pain at 6 months post-op.
Leg pain at 6 months post-op № of participants: 275 (4 RCTs)	Not applicable	MD **4.37 lower** (9.89 lower to 1.15 higher)	Low[f]	Prehabilitation may have little to no effect on leg pain at 6 months post-op.
Function at 6 months post-op № of participants: 448 (5 RCTs)	Not applicable	SMD **2.35 SD lower** (3.92 lower to 0.79 lower)	Moderate[3,m]	Prehabilitation results in large improvement in function at 6 months post-op.
Back pain at 12 months post-op № of participants: 280 (2 RCTs)	Not applicable	MD **1.22 higher** (0.37 lower to 2.81 higher)	Low[d]	Prehabilitation results in little to no difference in back pain at 12 months post-op.
Leg pain at 12 months post-op № of participants: 280 (2 RCTs)	Not applicable	MD **6.09 lower** (23.53 lower to 11.35 higher)	Low[2,g]	Prehabilitation may have little to no effect on leg pain at 12 months post-op.

© 2023 Punnoose A et al. *JAMA Network Open*.

eTable 6. GRADE Summary of Findings: Lumbar Surgery

Prehabilitation compared to standard care in patients undergoing Lumbar surgery

Patient or population: Patients undergoing Lumbar surgery
Setting: All
Intervention: Prehabilitation
Comparison: standard care

Outcome № of participants (studies)	Relative effect (95% CI)	Anticipated absolute effects (95% CI)	Certainty	What happens
		Difference		
Length of stay assessed with: days № of participants: 97 (2 RCTs)	Not applicable	MD **1.88 days fewer** (3.89 fewer to 0.13 more)	Low[p]	Prehabilitation may have little effect to no effect on length of stay after spinal surgery
Complications № of participants: 127 (2 RCTs)	**RR 2.69** (0.44 to 13.41)	**3.3% more** (1.1 fewer to 29.3 more)	Low[q]	Prehabilitation may result in an increase in complications, but evidence is very uncertain

***The risk in the intervention group** (and its 95% confidence interval) is based on the assumed risk in the comparison group and the **relative effect** of the intervention (and its 95% CI).

CI: confidence interval; **MD:** mean difference; **RR:** risk ratio; **SMD:** standardised mean difference

GRADE Working Group grades of evidence
High certainty: we are very confident that the true effect lies close to that of the estimate of the effect.
Moderate certainty: we are moderately confident in the effect estimate: the true effect is likely to be close to the estimate of the effect, but there is a possibility that it is substantially different.
Low certainty: our confidence in the effect estimate is limited: the true effect may be substantially different from the estimate of the effect.
Very low certainty: we have very little confidence in the effect estimate: the true effect is likely to be substantially different from the estimate of effect.

Explanations

a. Only 2 out of 4 studies showed moderate ROB and sensitivity analysis showed the results to remain significant. Mean difference does not meet the MCID of pain of 12 points on a 0-100 scale
b. Downgraded for moderate to high ROB (possible selective reporting, assessor blinding) in 2 out of 4 studies and sensitivity analysis showed the results to become non-significant. Downgraded for imprecision as small number of participants (<400). Mean difference does not meet the threshold for MCID of 12 points on 0-100 scale
c. Downgraded for moderate to high ROB (possible selective reporting, assessor blinding) in 2 out of 4 studies and sensitivity analysis did not alter the significance. Downgraded for imprecision as small number of participants (<400) and CI crossed the line of no effect. Mean difference does not meet the threshold for MCID of pain on 0-100 scale
d. Downgraded as both studies showed moderate to high ROB (possible selective reporting, assessor blinding). Mean difference does not meet the threshold for MCID of pain on 0-100 scale.
e. Downgraded as 2 out of 4 studies showed moderate ROB (possible selective reporting, assessor blinding). Downgraded as 95% CI crossed the line of no effect
f. Downgraded as 2 studies showed moderate to high ROB .Downgraded for imprecision as small number of participants (<400). 95% CI crosses line of no effect.Mean difference does not meet the threshold for MCID of pain on 0-100 scale
g. Downgraded as both studies showed moderate to high ROB. Mean difference does not meet the threshold for MCID of pain on 0-100 scale
h. Downgraded as 1 out 2 studies showed moderate ROB. Downgraded for imprecision as small sample size.
i. Downgraded as 1 out 2 studies showed moderate ROB. Downgraded for imprecision as small sample size
j. Downgraded as 2 out of 4 studies showed moderate ROB (possible selective reporting, assessor blinding).Although interventions differed in one study (CBT vs exercises), unexplained heterogeneity remained in subgroup analysis; therefore downgraded. Downgraded due to substantial levels of heterogeneity and small number of participants(<400).
k. Downgraded for imprecision as small sample size and 95% CI crosses line of no effect. MD does not meet the MCID for ODI

© 2023 Punnoose A et al. *JAMA Network Open*.

l. Downgraded as 3 out of 5 studies showed moderate to high ROB (possible selective reporting, assessor blinding). Downgraded for substantial heterogeneity and imprecision due to 95% CI crossing the line of no effect. Upgraded due to a very large effect size (SMD >0.80)
m. Downgraded as 3 out of 5 studies showed moderate to high ROB (possible selective reporting, assessor blinding). Considerable heterogeneity noted and therefore downgraded by 2. Upgraded by 2 levels due to very large effect size (SMD >1.0)
n. Downgraded for imprecision as small sample size.
o. Downgraded as 2 out of 3 studies showed moderate to high ROB (possible selective reporting, assessor blinding). Downgraded for imprecision as small sample size(<400) and 95% CI crosses the line of no effect.
p. Downgraded for moderate ROB in 1 study (possible selective reporting).Overall reduction of 1.88 days which was significant. However, downgraded for imprecision for small sample size and 95% CI crossed the line of no effect.
q. Downgraded as 1 study showed high ROB and the other moderate (possible selective reporting, assessor blinding). Upgraded as Large effect size(as per GRADE) favouring standard arm.

References

1.Copay AG, Glassman SD,Subach BR,Berven S,Schuler TC,Carreon LY. Minimum clinically important difference in lumbar spine surgery patients: a choice of methods using the Oswestry Disability Index, Medical Outcomes Study questionnaire Short Form 36, and Pain Scales. Spine..J; 2008.
2.Deeks JJ, Higgins JPT,Altman DG (editors). Chapter 10: Analysing data and undertaking meta-analyses. . Higgins JPT, Thomas J, Chandler J, Cumpston M, Li T, Page MJ, Welch VA (editors). ; Feb 2002.
3.Cohen, J. Statistical Power Analysis for the Behavioral Sciences (2nd ed.). Hillsdale, NJ: Lawrence Erlbaum Associates, Publishers.; 1988.

© 2023 Punnoose A et al. *JAMA Network Open*.

eTable 7. GRADE Summary of Findings: THR and TKR

Prehabilitation compared to standard care for Total Hip or Total Knee replacements

Patient or population: Total Hip or Total Knee replacements
Setting: All
Intervention: Prehabilitation
Comparison: standard care

Outcome № of participants (studies)	Relative effect (95% CI)	Anticipated absolute effects (95% CI)	Certainty	What happens
		**Difference**		
Complications № of participants: 1563 (17 RCTs)	**RR 0.85** (0.68 to 1.06)	**2.8% fewer** (5.3 fewer to 0.2 more)	Low[a]	Prehabilitation may result in little to no difference in complications.

***The risk in the intervention group** (and its 95% confidence interval) is based on the assumed risk in the comparison group and the **relative effect** of the intervention (and its 95% CI).

CI: confidence interval; **RR:** risk ratio

GRADE Working Group grades of evidence
High certainty: we are very confident that the true effect lies close to that of the estimate of the effect.
Moderate certainty: we are moderately confident in the effect estimate: the true effect is likely to be close to the estimate of the effect, but there is a possibility that it is substantially different.
Low certainty: our confidence in the effect estimate is limited: the true effect may be substantially different from the estimate of the effect.
Very low certainty: we have very little confidence in the effect estimate: the true effect is likely to be substantially different from the estimate of effect.

Explanations

a. Downgraded as majority of studies included showed moderate to high ROB. Downgraded as the 95% Ci crossed the line of no effect. Did not demonstrate a large magnitude of effect of >2 or <0.5 as per the GRADE handbook

eFigure 3. Pain: Preoperative and 3 Months and 6 Months Postoperative for THR

	Prehab			Standard care				Std. Mean Difference
Study or Subgroup	Mean	SD	Total	Mean	SD	Total	Weight	IV, Random, 95% CI
**1.7.1 Pain at Pre-op**								
Berge 2004	44.7	19.7	19	66.5	29.7	18	10.4%	−0.85 [−1.53, −0.17]
Doiron-Cadrin 2019 (Inperson prehab vs control)	40	25	6	45	14.5	3	2.5%	−0.20 [−1.59, 1.19]
Doiron-Cadrin 2019 (Teleprehab vs control)	45	15.5	6	45	14.5	2	1.9%	0.00 [−1.60, 1.60]
Ferrara 2015	40	19	11	55	18	12	6.5%	−0.78 [−1.64, 0.07]
Hermann 2015	−55.4	16.7	39	−46.1	14.1	40	23.4%	−0.60 [−1.05, −0.15]
Hoogeboom 2010	−55.3	12	10	−49.3	17	10	6.1%	−0.39 [−1.28, 0.50]
Oosting 2012	−66.8	9.2	12	−67.8	12.2	14	8.0%	0.09 [−0.68, 0.86]
Rooks 2006	39	205	25	49.5	14.5	24	15.2%	−0.07 [−0.63, 0.49]
Zeng 2014	46.5	9.85	40	52.75	10.1	49	26.0%	−0.62 [−1.05, −0.19]
**Subtotal (95% CI)**			**168**			**172**	**100.0%**	**−0.47 [−0.69, −0.25]**
Heterogeneity: $Tau^2 = 0.00$; $Chi^2 = 6.98$, df = 8 (P = 0.54); $I^2 = 0\%$								
Test for overall effect: Z = 4.25 (P < 0.0001)								
**1.7.3 Pain at 3 months post-op**								
Ferrara 2015	8.5	11.75	11	11	8.75	12	15.7%	−0.23 [−1.06, 0.59]
Hermann 2015	−85.23	15.58	36	−81.44	16.16	38	50.6%	−0.24 [−0.69, 0.22]
Rooks 2006	13	13	25	13.5	10	24	33.7%	−0.04 [−0.60, 0.52]
**Subtotal (95% CI)**			**72**			**74**	**100.0%**	**−0.17 [−0.50, 0.15]**
Heterogeneity: $Tau^2 = 0.00$; $Chi^2 = 0.30$, df = 2 (P = 0.86); $I^2 = 0\%$								
Test for overall effect: Z = 1.03 (P = 0.30)								
**1.7.4 Pain at 6 months post-op**								
Hermann 2015	−86.83	15.3	30	−80.69	20.23	36	56.1%	−0.33 [−0.82, 0.15]
Rooks 2006	5.5	8.5	25	5	6	24	43.9%	0.07 [−0.49, 0.63]
**Subtotal (95% CI)**			**55**			**60**	**100.0%**	**−0.16 [−0.55, 0.23]**
Heterogeneity: $Tau^2 = 0.01$; $Chi^2 = 1.12$, df = 1 (P = 0.29); $I^2 = 10\%$								
Test for overall effect: Z = 0.80 (P = 0.43)								

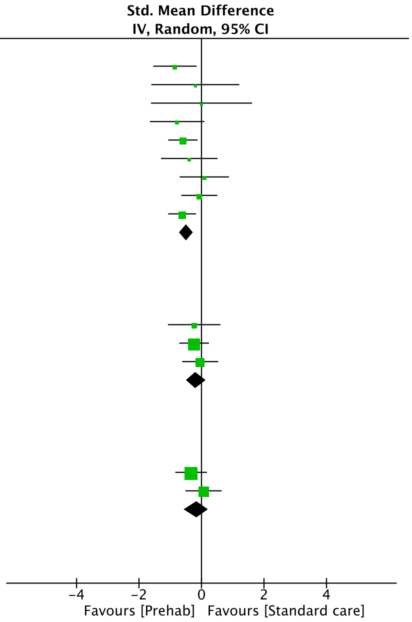

© 2023 Punnoose A et al. *JAMA Network Open*.

eFigure 4. Pain: Preoperative and 3 Months, 6 Months, and 12 Months Postoperative for Total Knee Replacement Surgery (TKR)

Study or Subgroup	Prehabilitation Mean	Prehabilitation SD	Prehabilitation Total	Standard care Mean	Standard care SD	Standard care Total	Weight	Std. Mean Difference IV, Random, 95% CI
**1.9.1 Pain at pre-op**								
An 2021 (Pre-op education vs standard care)	57.65	12.85	17	63.35	8	9	4.2%	-0.48 [-1.30, 0.34]
An 2021 (Tele-prehab vs standard care)	55	5.7	18	63.35	8	9	4.0%	-1.24 [-2.12, -0.36]
Beaupre 2004	-48	13	51	-49	17	58	5.6%	0.07 [-0.31, 0.44]
Calatayud 2017	34	4.5	22	51.5	4.5	22	3.6%	-3.82 [-4.84, -2.80]
Doiron-Cadrin 2019 (In-person prehab vs Control)	32.5	23.5	5	49	24	3	2.4%	-0.61 [-2.10, 0.88]
Doiron-Cadrin 2019 (Tele-prehab vs Control)	38	15.5	6	49	24	2	2.1%	-0.56 [-2.20, 1.09]
Dominguez-Navarro 2020 (ST vs Control)	-61.8	9.9	24	-49.3	13	10	4.3%	-1.12 [-1.91, -0.33]
Dominguez-Navarro 2020 (STB vs Control)	-57.6	11.1	20	-49.3	13	11	4.4%	-0.69 [-1.44, 0.07]
Franz 2022 (Prehab+BFR vs Standard care)	-52.8	4.1	10	-48	4.1	5	3.2%	-1.10 [-2.27, 0.07]
Franz 2022 (Prehab+Sham BFR vs Standard care	-50	4.5	10	-48	4.1	5	3.4%	-0.43 [-1.52, 0.66]
Huang 2012	64	10	126	65	10	117	5.9%	-0.10 [-0.35, 0.15]
Huber 2015	-48.9	17.14	21	-50.3	16.24	19	4.9%	0.08 [-0.54, 0.70]
McKay 2012	43.5	18.85	10	45	22.05	12	4.1%	-0.07 [-0.91, 0.77]
NCT01844934	31.7	28.1	9	21.5	12	10	3.9%	0.46 [-0.46, 1.38]
Rooks 2006	36.5	3.5	14	37.5	25	15	4.5%	-0.05 [-0.78, 0.67]
Savkin 2021	-53.06	17.93	20	-37.08	15.35	20	4.7%	-0.94 [-1.59, -0.28]
Skoffer 2016	-59.2	15.7	29	-55.2	18.5	26	5.2%	-0.23 [-0.76, 0.30]
Soni 2012	60	27	25	58	30	28	5.1%	0.07 [-0.47, 0.61]
Swank 2011	43	3.8	36	51	4.2	35	5.0%	-1.98 [-2.55, -1.40]
Topp 2009	47.7	22.9	26	68	22.7	28	5.1%	-0.88 [-1.44, -0.32]
Walls 2010	57	16.5	9	59	20	5	3.4%	-0.11 [-1.20, 0.99]
Williamson 2007 - (Acupuncture vs Control)	65.8	22.9	60	72.4	20.7	30	5.4%	-0.29 [-0.74, 0.15]
Williamson 2007- (Exercises vs Control)	63.6	26	60	72.4	20.7	31	5.4%	-0.36 [-0.80, 0.08]
**Subtotal (95% CI)**			**628**			**510**	**100.0%**	**-0.58 [-0.88, -0.28]**
Heterogeneity: Tau² = 0.38; Chi² = 108.45, df = 22 (P < 0.00001); I² = 80%								
Test for overall effect: Z = 3.82 (P = 0.0001)								
**1.9.2 Pain at 6 weeks post-op**								
An 2021 (Pre-op education vs standard care)	30	5	17	35.25	12.75	9	7.1%	-0.60 [-1.43, 0.22]
An 2021 (Tele-prehab vs standard care)	17.5	6.65	18	35.25	12.75	9	6.2%	-1.90 [-2.87, -0.93]
Dominguez-Navarro 2020 (ST vs Control)	-79.1	17.5	24	-77.3	12	10	7.7%	-0.11 [-0.85, 0.63]
Dominguez-Navarro 2020 (STB vs Control)	-74.3	17.5	20	-77.3	12	11	7.7%	0.18 [-0.55, 0.92]
Gstoettner 2011	13	11	15	9.8	9.9	20	8.1%	0.30 [-0.37, 0.97]
Huber 2015	-61.3	17.26	21	-66.9	17.69	21	8.6%	0.31 [-0.29, 0.92]
Mat Eil-Ismail 2016	-86.94	13.92	24	-82.95	18.98	26	9.0%	-0.23 [-0.79, 0.32]
McKay 2012	28	13.6	9	24.6	22.5	10	6.6%	0.17 [-0.73, 1.08]
NCT01844934	27.2	20	9	19.5	17.2	10	6.5%	0.40 [-0.52, 1.31]
Savkin 2021	-73.06	17.2	20	-66.53	17.67	20	8.5%	-0.37 [-0.99, 0.26]
Skoffer 2016	-70.9	16.7	29	-67.3	13	23	9.1%	-0.23 [-0.78, 0.32]
Villadsen 2013	-67.5	12.46	41	-51.5	14.27	40	9.6%	-1.18 [-1.66, -0.71]
Walls 2010	39.5	29.5	9	27	15	5	5.4%	0.46 [-0.65, 1.57]
**Subtotal (95% CI)**			**256**			**214**	**100.0%**	**-0.23 [-0.58, 0.12]**
Heterogeneity: Tau² = 0.27; Chi² = 38.42, df = 12 (P = 0.0001); I² = 69%								
Test for overall effect: Z = 1.28 (P = 0.20)								
**1.9.3 Pain at 3 months post-op**								
Beaupre 2004	-74	18	51	-73	14	58	6.8%	-0.06 [-0.44, 0.31]
Calatayud 2017	14.5	4.5	22	19	4.5	22	4.5%	-0.98 [-1.61, -0.35]
Franz 2022 (Prehab+BFR vs Standard care)	-67.8	3.5	10	-60.8	6.9	5	1.8%	-1.37 [-2.59, -0.15]
Franz 2022 (Prehab+Sham BFR vs Standard care	-61.7	6.8	10	-60.8	6.9	5	2.2%	-0.12 [-1.20, 0.95]
Huber 2015	-71.4	17.14	21	-74	17.23	20	4.6%	0.15 [-0.46, 0.76]
Mat Eil-Ismail 2016	-94.35	9.16	24	-88.06	9.19	26	4.9%	-0.67 [-1.25, -0.10]
McKay 2012	22	16	7	17.9	22	10	2.6%	0.20 [-0.77, 1.17]
Mitchell 2005	34	18.5	56	34.5	21.5	56	6.9%	-0.02 [-0.40, 0.35]
NCT01844934	13.9	13.2	9	4	9.7	10	2.6%	0.82 [-0.12, 1.77]
NCT03044028	-72.22	19.64	22	-80.38	15.43	22	4.7%	0.45 [-0.15, 1.05]
Riddle 2019 (Education vs Control)	30.5	24.7	116	30.5	23.75	60	7.5%	0.00 [-0.31, 0.31]
Riddle 2019-(CBT vs Control)	32	24.15	111	30.5	23.75	61	7.5%	0.06 [-0.25, 0.37]
Rooks 2006	23.5	12	14	25	17	15	3.8%	-0.10 [-0.83, 0.63]
Savkin 2021	-83.89	15.2	20	-86.39	12.96	20	4.5%	0.17 [-0.45, 0.79]
Skoffer 2016	-78.1	16.3	29	-79.9	14.2	21	5.0%	0.11 [-0.45, 0.68]
Soni 2012	47	28	21	39	30	20	4.6%	0.27 [-0.34, 0.89]
Topp 2009	15.3	17.3	26	13.8	17.4	28	5.2%	0.09 [-0.45, 0.62]
Villadsen 2013	-72.7	12.46	41	-59.1	14.27	40	5.9%	-1.01 [-1.47, -0.54]
Walls 2010	27	20	9	26	21.5	5	2.1%	0.05 [-1.05, 1.14]
Williamson 2007 - (Acupuncture vs Control)	29.4	26.7	60	39.5	25.9	30	6.1%	-0.38 [-0.82, 0.06]
Williamson 2007- (Exercises vs Control)	38.6	25.9	60	39.5	25.9	31	6.2%	-0.03 [-0.47, 0.40]
**Subtotal (95% CI)**			**739**			**565**	**100.0%**	**-0.11 [-0.29, 0.07]**
Heterogeneity: Tau² = 0.09; Chi² = 44.71, df = 20 (P = 0.001); I² = 55%								
Test for overall effect: Z = 1.17 (P = 0.24)								
**1.9.4 Pain at 6 months post-op**								
Beaupre 2004	-16	15	51	-15	15	58	23.1%	-0.07 [-0.44, 0.31]
Franz 2022 (Prehab+BFR vs Standard care)	-70	4.7	10	-71.1	7.9	5	2.8%	0.18 [-0.90, 1.25]
Franz 2022 (Prehab+Sham BFR vs Standard care	-76.2	3.6	10	-71.1	7.9	5	2.5%	-0.90 [-2.04, 0.23]
Riddle 2019 (Education vs Control)	19	23.6	106	22	23.65	59	32.3%	-0.13 [-0.45, 0.19]
Riddle 2019-(CBT vs Control)	20.5	24.05	110	22	23.65	60	33.1%	-0.06 [-0.38, 0.25]
Rooks 2006	12	13.5	14	11.5	10	15	6.2%	0.04 [-0.69, 0.77]
**Subtotal (95% CI)**			**301**			**202**	**100.0%**	**-0.09 [-0.27, 0.09]**
Heterogeneity: Tau² = 0.00; Chi² = 2.42, df = 5 (P = 0.79); I² = 0%								
Test for overall effect: Z = 1.00 (P = 0.32)								
**1.9.5 Pain at 12 months post-op**								
Beaupre 2004	-82	13	51	-80	16	58	19.4%	-0.14 [-0.51, 0.24]
Dominguez-Navarro 2020 (ST vs Control)	-90.2	5.7	24	-88.7	7.8	10	5.0%	-0.23 [-0.97, 0.51]
Dominguez-Navarro 2020 (STB vs Control)	-92.2	4.4	20	-88.7	7.8	11	4.9%	-0.59 [-1.34, 0.16]
Huber 2015	-86.6	15.9	17	-86.9	17.32	19	6.4%	0.02 [-0.64, 0.67]
Riddle 2019 (Education vs Control)	15	25.15	113	14.5	26.85	60	28.1%	0.02 [-0.29, 0.33]
Riddle 2019-(CBT vs Control)	16.5	24.7	111	14.5	26.85	62	28.5%	0.08 [-0.23, 0.39]
Skoffer 2016	-89.9	13.2	24	-89	10.1	20	7.8%	-0.07 [-0.67, 0.52]
**Subtotal (95% CI)**			**360**			**240**	**100.0%**	**-0.04 [-0.21, 0.12]**
Heterogeneity: Tau² = 0.00; Chi² = 3.27, df = 6 (P = 0.77); I² = 0%								
Test for overall effect: Z = 0.51 (P = 0.61)								

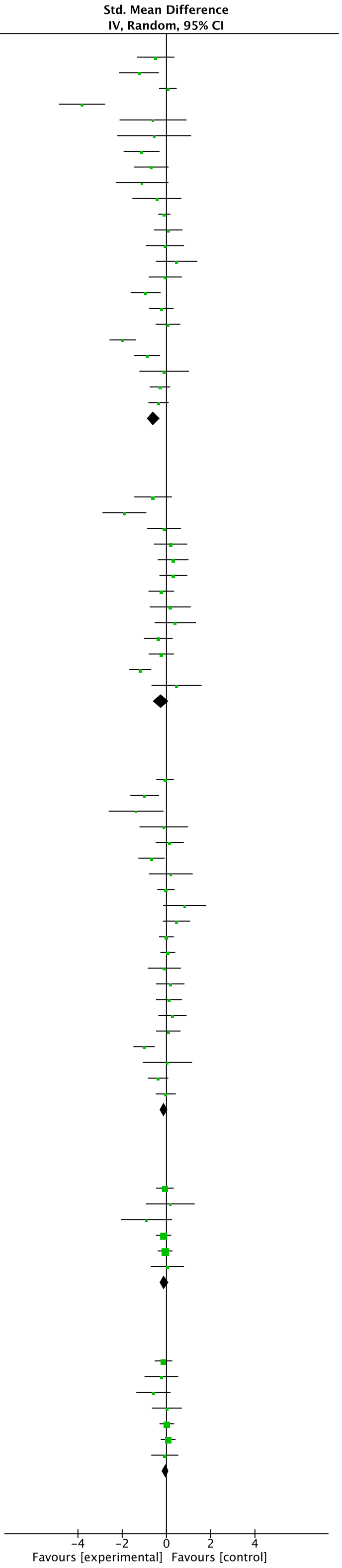

© 2023 Punnoose A et al. *JAMA Network Open*.

eFigure 5. Function: Preoperative and 3 Months, 6 Months, and 12 Months Postoperative for THR

Study or Subgroup	Prehab Mean	Prehab SD	Prehab Total	Standard care Mean	Standard care SD	Standard care Total	Weight	Std. Mean Difference IV, Random, 95% CI
**1.11.1 Function at Pre-op**								
Doiron-Cadrin 2019 (Inperson prehab vs control)	24.55	19.7	6	46.76	11.61	3	2.7%	-1.11 [-2.66, 0.43]
Doiron-Cadrin 2019 (Teleprehab vs control)	42.64	10.73	6	46.76	11.61	2	2.5%	-0.33 [-1.95, 1.29]
Ferrara 2015	49.55	20.29	11	63.97	13.97	12	7.8%	-0.80 [-1.66, 0.05]
Gocen 2004	-51.48	18.32	29	-45.3	12.98	30	16.7%	-0.39 [-0.90, 0.13]
Hermann 2014	-59.9	17.1	37	-48.7	13.9	39	18.9%	-0.71 [-1.18, -0.25]
Hoogeboom 2010	-51	10.5	10	-52.3	21.2	10	7.5%	0.07 [-0.80, 0.95]
Oosting 2012	-65.2	10.6	12	-68.7	14.9	14	9.2%	0.26 [-0.52, 1.03]
Rooks 2006	39.55	17.5	25	49.55	16.02	24	14.5%	-0.59 [-1.16, -0.01]
Zeng 2014	53.35	7.51	40	60.41	7.58	49	20.2%	-0.93 [-1.37, -0.49]
**Subtotal (95% CI)**			**176**			**183**	**100.0%**	**-0.54 [-0.81, -0.28]**
Heterogeneity: $Tau^2 = 0.04$; $Chi^2 = 10.70$, df = 8 (P = 0.22); $I^2 = 25\%$								
Test for overall effect: Z = 4.05 (P < 0.0001)								
**1.11.2 Function at 3 months post-op**								
Cavill 2016	-50	20	11	-55	26	12	8.3%	0.21 [-0.61, 1.03]
Ferrara 2015	26.9	18.52	11	41.91	14.72	10	6.9%	-0.86 [-1.76, 0.05]
Gocen 2004	-85.3	11.78	29	-78.7	9.41	30	18.8%	-0.61 [-1.14, -0.09]
Hermann 2015	-86.44	13.37	36	-79.61	16.62	38	23.3%	-0.45 [-0.91, 0.02]
Rooks 2006	18.82	13.23	25	18.97	11.76	24	16.7%	-0.01 [-0.57, 0.55]
Villadsen 2013	-79.7	10.75	43	-75.8	5.48	41	25.9%	-0.45 [-0.88, -0.02]
**Subtotal (95% CI)**			**155**			**155**	**100.0%**	**-0.38 [-0.62, -0.14]**
Heterogeneity: $Tau^2 = 0.01$; $Chi^2 = 5.62$, df = 5 (P = 0.34); $I^2 = 11\%$								
Test for overall effect: Z = 3.06 (P = 0.002)								
**1.11.3 Function at 6 months post-op**								
Hermann 2015	-86.3	14.34	30	-81.44	19.26	36	56.9%	-0.28 [-0.77, 0.21]
Rooks 2006	7.94	8.52	25	7.79	7.94	24	43.1%	0.02 [-0.54, 0.58]
**Subtotal (95% CI)**			**55**			**60**	**100.0%**	**-0.15 [-0.52, 0.22]**
Heterogeneity: $Tau^2 = 0.00$; $Chi^2 = 0.62$, df = 1 (P = 0.43); $I^2 = 0\%$								
Test for overall effect: Z = 0.81 (P = 0.42)								
**1.11.4 Function at 1-2 years post-op**								
Berge 2004	47.65	9.31	18	54.61	9.31	15	19.5%	-0.73 [-1.44, -0.02]
Gocen 2004	-97.14	4.32	29	-95.66	6.08	30	37.3%	-0.28 [-0.79, 0.24]
Hermann 2015	-86.5	13.64	34	-82.47	22.68	34	43.2%	-0.21 [-0.69, 0.26]
**Subtotal (95% CI)**			**81**			**79**	**100.0%**	**-0.34 [-0.65, -0.02]**
Heterogeneity: $Tau^2 = 0.00$; $Chi^2 = 1.49$, df = 2 (P = 0.48); $I^2 = 0\%$								
Test for overall effect: Z = 2.11 (P = 0.04)								

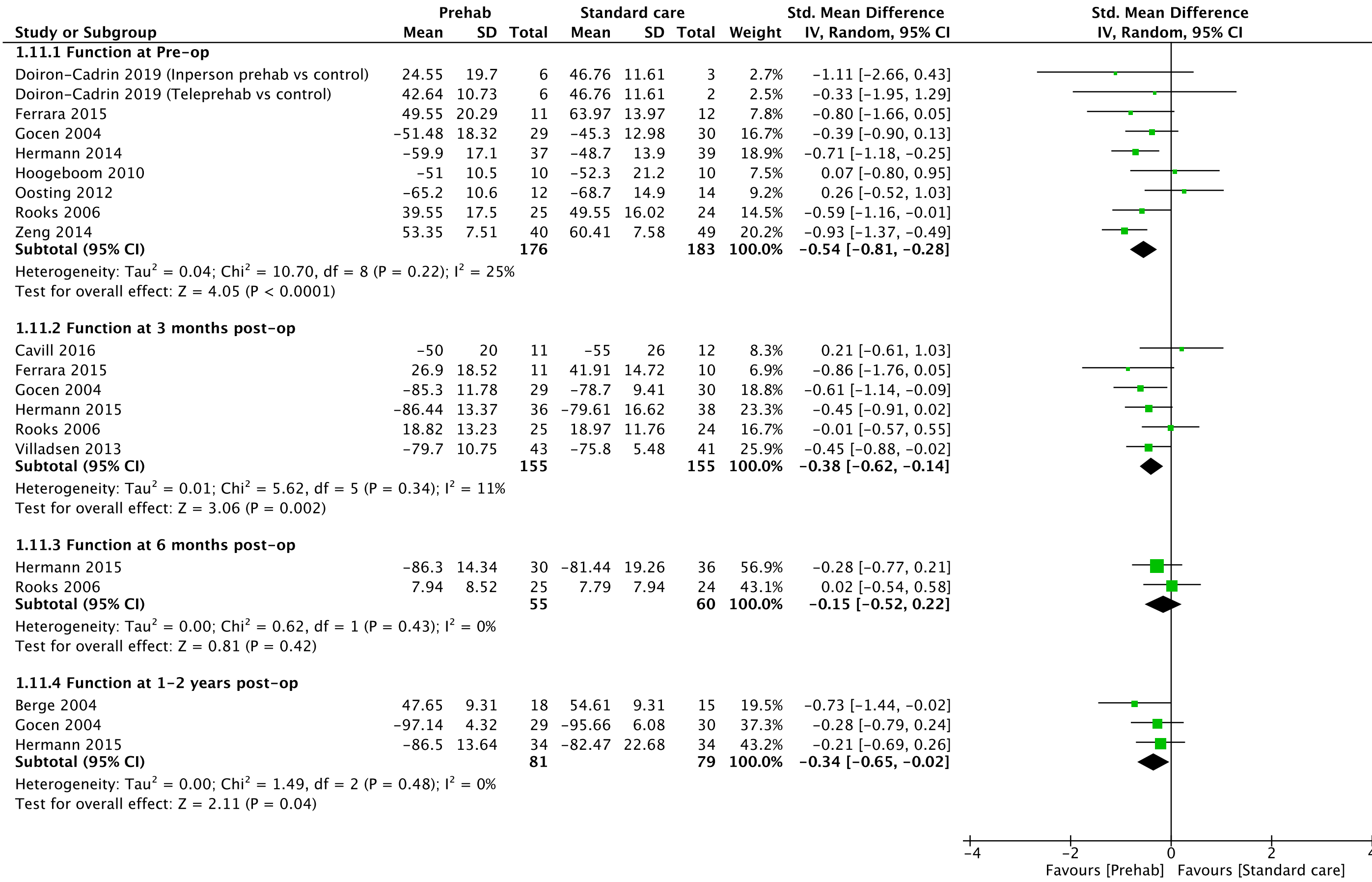

© 2023 Punnoose A et al. *JAMA Network Open*.

eFigure 6. Function: Preoperative and 3 Months, 6 Months, and 12 Months Postoperative for TKR

Study or Subgroup	Prehabilitation Mean	Prehabilitation SD	Prehabilitation Total	Standard care Mean	Standard care SD	Standard care Total	Weight	Std. Mean Difference IV, Random, 95% CI
**1.10.1 Function at pre-op**								
Beaupre 2004	−50	14	51	−51	17	58	7.4%	0.06 [−0.31, 0.44]
Calatayud 2017	−49	2.48	22	−40.2	2.48	22	5.3%	−3.48 [−4.45, −2.52]
Doiron−Cadrin 2019 (In−person prehab vs Control)	34.5	11.61	5	54.7	20	3	3.2%	−1.18 [−2.82, 0.47]
Doiron−Cadrin 2019 (Tele−prehab vs Control)	37.9	11.32	6	54.7	20	2	2.9%	−1.11 [−2.88, 0.66]
Dominguez−Navarro 2020 (ST vs Control)	−62.4	10.1	24	−51.3	11.7	10	6.0%	−1.02 [−1.81, −0.24]
Dominguez−Navarro 2020 (STB vs Control)	−61.1	10.8	20	−51.3	11.7	11	6.0%	−0.86 [−1.63, −0.09]
Evgeniadis 2008	−19.9	2.77	18	−19.6	3.4	20	6.5%	−0.09 [−0.73, 0.54]
Franz 2022 (Prehab+BFR vs Standard care)	−53.5	6.1	10	−49	4.4	5	4.7%	−0.75 [−1.87, 0.37]
Franz 2022 (Prehab+Sham BFR vs Standard care	−50.5	3.8	10	−49	4.4	5	4.9%	−0.35 [−1.44, 0.73]
Huber 2015	−45.8	24.48	21	−39.4	21.48	19	6.6%	−0.27 [−0.90, 0.35]
Jahic 2018	−40.5	7.25	10	−29.5	7.25	10	5.1%	−1.45 [−2.46, −0.44]
Jungae 2021 (Pre−op education vs standard care)	47.94	4.52	17	48.16	6.34	9	5.9%	−0.04 [−0.85, 0.77]
Jungae 2021 (Tele−prehab vs standard care)	40.47	4.81	18	48.16	6.34	9	5.5%	−1.39 [−2.29, −0.50]
McKay 2012	41.9	18.48	10	44.1	20.11	12	5.8%	−0.11 [−0.95, 0.73]
Rooks 2006	40.7	17.05	14	36.7	17.5	15	6.2%	0.22 [−0.51, 0.96]
Savkin 2021	−61.47	15.89	20	−42.65	11.31	20	6.3%	−1.34 [−2.03, −0.65]
Skoffer 2016	−64.1	16.1	29	−60.6	15.9	26	6.9%	−0.22 [−0.75, 0.32]
Walls 2010	51	16.17	9	56.4	21.17	5	4.8%	−0.28 [−1.38, 0.82]
**Subtotal (95% CI)**			**314**			**261**	**100.0%**	**−0.70 [−1.08, −0.32]**
Heterogeneity: Tau² = 0.48; Chi² = 71.31, df = 17 (P < 0.00001); I² = 76%								
Test for overall effect: Z = 3.57 (P = 0.0004)								
**1.10.2 Function at 6 weeks post-op**								
Dominguez−Navarro 2020 (ST vs Control)	−73.3	16.4	24	−75.7	9.1	10	8.2%	0.16 [−0.58, 0.90]
Dominguez−Navarro 2020 (STB vs Control)	−77.6	9	20	−75.7	9.1	11	8.2%	−0.20 [−0.94, 0.53]
Gstoettner 2011	12	12	15	19	10	20	8.7%	−0.63 [−1.32, 0.06]
Huber 2015	−53.6	24.48	21	−53.9	29.9	21	9.4%	0.01 [−0.59, 0.62]
Jungae 2021 (Pre−op education vs standard care)	41	6.57	17	46.89	9.72	9	7.4%	−0.73 [−1.57, 0.10]
Jungae 2021 (Tele−prehab vs standard care)	32.1	6.33	18	46.89	9.72	9	6.4%	−1.89 [−2.86, −0.92]
Mat Eil−Ismail 2016	−81.5	14.69	24	−74.5	14.75	26	9.8%	−0.47 [−1.03, 0.09]
McKay 2012	26.6	17.42	9	28.1	22.07	10	6.9%	−0.07 [−0.97, 0.83]
Savkin 2021	−83.68	13.22	20	−73.53	17.76	20	9.1%	−0.64 [−1.27, 0.00]
Skoffer 2016	−76.1	13.8	29	−70.6	11.4	23	9.9%	−0.42 [−0.98, 0.13]
Villadsen 2013	−76	11.07	41	−59.2	12.57	40	10.5%	−1.41 [−1.89, −0.92]
Walls 2010	43.8	24.85	9	35.5	14.55	5	5.5%	0.35 [−0.75, 1.46]
**Subtotal (95% CI)**			**247**			**204**	**100.0%**	**−0.51 [−0.85, −0.17]**
Heterogeneity: Tau² = 0.22; Chi² = 31.07, df = 11 (P = 0.001); I² = 65%								
Test for overall effect: Z = 2.96 (P = 0.003)								
**1.10.3 Function at 3 months post-op**								
Beaupre 2004	−73	17	51	−73	15	58	7.6%	0.00 [−0.38, 0.38]
Brown 2012	−66.4	20.7	11	−39.3	30.2	7	3.1%	−1.05 [−2.07, −0.02]
Calatayud 2017	−55.7	2.48	22	−53	2.48	22	5.3%	−1.07 [−1.70, −0.43]
Cavill 2016	−47	21	20	−55	23	19	5.3%	0.36 [−0.28, 0.99]
Franz 2022 (Prehab+BFR vs Standard care)	−63.7	5.1	10	−58.7	4.5	5	2.6%	−0.96 [−2.10, 0.19]
Franz 2022 (Prehab+Sham BFR vs Standard care	−59	3.5	10	−58.7	4.5	5	2.9%	−0.07 [−1.15, 1.00]
Huber 2015	−65.8	24.36	21	−73.1	22.68	20	5.5%	0.30 [−0.31, 0.92]
Jahic 2018	−68	2.58	10	−67.5	2.64	10	3.7%	−0.18 [−1.06, 0.70]
Mat Eil−Ismail 2016	−89.5	10.57	24	−80.5	10.6	26	5.8%	−0.84 [−1.42, −0.26]
McKay 2012	19.2	17	7	21	22.67	10	3.3%	−0.08 [−1.05, 0.88]
Mitchell 2005	36.6	19.7	56	38.8	21.91	56	7.6%	−0.10 [−0.48, 0.27]
NCT03044028	−33.43	14.76	22	−26.6	9.81	22	5.6%	−0.54 [−1.14, 0.07]
Riddle 2019 (Education vs Control)	28.6	25.85	116	31.1	25.88	60	8.2%	−0.10 [−0.41, 0.22]
Riddle 2019−(CBT vs Control)	33	25.67	111	31.1	25.88	61	8.2%	0.07 [−0.24, 0.39]
Rooks 2006	23.9	10.44	14	22.5	16.76	15	4.6%	0.10 [−0.63, 0.83]
Savkin 2021	−89.85	12.35	20	−87.21	11.19	20	5.4%	−0.22 [−0.84, 0.40]
Skoffer 2016	−82.9	11.7	29	−78.2	12.9	21	5.9%	−0.38 [−0.95, 0.19]
Villadsen 2013	−80.5	11.34	41	−65.6	12.57	40	6.7%	−1.23 [−1.71, −0.76]
Walls 2010	29.2	20.44	9	31.7	16.61	5	2.8%	−0.12 [−1.22, 0.97]
**Subtotal (95% CI)**			**604**			**482**	**100.0%**	**−0.29 [−0.51, −0.08]**
Heterogeneity: Tau² = 0.12; Chi² = 46.49, df = 18 (P = 0.0003); I² = 61%								
Test for overall effect: Z = 2.66 (P = 0.008)								
**1.10.4 Function at 6 months post-op**								
Beaupre 2004	−78	15	51	−74	15	58	20.4%	−0.26 [−0.64, 0.11]
Franz 2022 (Prehab+BFR vs Standard care)	−71.9	3.1	10	−65.6	4.6	5	6.1%	−1.63 [−2.91, −0.36]
Franz 2022 (Prehab+Sham BFR vs Standard care	−67.8	3.1	10	−65.6	4.6	5	7.6%	−0.57 [−1.67, 0.53]
Jahic 2018	−77.5	2.64	10	−77.5	2.64	10	10.2%	0.00 [−0.88, 0.88]
Riddle 2019 (Education vs Control)	21.6	24.7	106	15.4	24.3	59	21.8%	0.25 [−0.07, 0.57]
Riddle 2019−(CBT vs Control)	22.3	25.17	110	15.4	24.3	60	21.9%	0.28 [−0.04, 0.59]
Rooks 2006	14.5	13.23	14	2.05	17.5	15	12.1%	0.78 [0.02, 1.54]
**Subtotal (95% CI)**			**311**			**212**	**100.0%**	**0.01 [−0.35, 0.37]**
Heterogeneity: Tau² = 0.13; Chi² = 17.31, df = 6 (P = 0.008); I² = 65%								
Test for overall effect: Z = 0.07 (P = 0.95)								
**1.10.5 Function at 12 months post-op**								
Beaupre 2004	−77	14	51	−77	16	58	19.4%	0.00 [−0.38, 0.38]
Dominguez−Navarro 2020 (ST vs Control)	−88.1	6.8	24	−87.8	4.6	10	5.0%	−0.05 [−0.78, 0.69]
Dominguez−Navarro 2020 (STB vs Control)	−88.2	5.5	20	−87.8	4.6	11	5.1%	−0.07 [−0.81, 0.66]
Huber 2015	−70.8	15.41	17	−73.6	19.17	19	6.4%	0.16 [−0.50, 0.81]
Jahic 2018	−92	4.22	10	−90	0	10		Not estimable
Riddle 2019 (Education vs Control)	17.2	25.11	113	15.4	25.67	60	28.0%	0.07 [−0.24, 0.38]
Riddle 2019−(CBT vs Control)	17.9	25.29	111	15.4	25.67	62	28.4%	0.10 [−0.21, 0.41]
Skoffer 2016	−87.6	12.3	24	−84.4	11.8	20	7.7%	−0.26 [−0.86, 0.34]
**Subtotal (95% CI)**			**370**			**250**	**100.0%**	**0.03 [−0.13, 0.20]**
Heterogeneity: Tau² = 0.00; Chi² = 1.45, df = 6 (P = 0.96); I² = 0%								
Test for overall effect: Z = 0.37 (P = 0.71)								

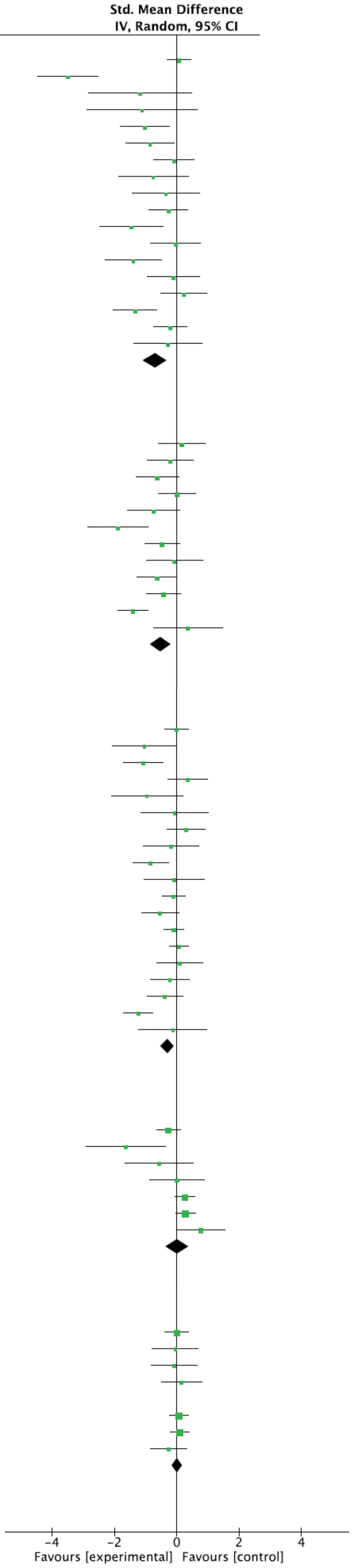

© 2023 Punnoose A et al. *JAMA Network Open*.

eFigure 7. Health-Related Quality of Life (HRQOL): Preoperative for THR on SF-36

	Prehab			Standard care				Mean Difference
**Study or Subgroup**	**Mean**	**SD**	**Total**	**Mean**	**SD**	**Total**	**Weight**	**IV, Random, 95% CI**
**1.17.1 HRQoL at pre-op on SF-36 PCS**								
Doiron-Cadrin 2019 (Inperson prehab vs control)	35.5	8.2	6	33.9	8.8	3	12.4%	1.60 [−10.33, 13.53]
Doiron-Cadrin 2019 (Teleprehab vs control)	38.2	6.3	6	33.9	8.8	2	10.1%	4.30 [−8.90, 17.50]
Ferrara 2015	34.4	4.05	11	27.3	10.3	12	44.3%	7.10 [0.80, 13.40]
**Subtotal (95% CI)**			**23**			**17**	**66.8%**	**5.66 [0.53, 10.79]**
Heterogeneity: $Tau^2 = 0.00$; $Chi^2 = 0.69$, df = 2 (P = 0.71); $I^2 = 0\%$								
Test for overall effect: Z = 2.16 (P = 0.03)								
**1.17.2 HRQoL at Pre-op on SF-36 MCS**								
Doiron-Cadrin 2019 (Inperson prehab vs control)	42.8	10.7	6	35.7	11.6	3	7.2%	7.10 [−8.57, 22.77]
Doiron-Cadrin 2019 (Teleprehab vs control)	52.8	8.2	6	35.7	11.6	2	5.8%	17.10 [−0.26, 34.46]
Ferrara 2015	51.1	11.2	11	40.9	11.6	12	20.2%	10.20 [0.88, 19.52]
**Subtotal (95% CI)**			**23**			**17**	**33.2%**	**10.74 [3.47, 18.02]**
Heterogeneity: $Tau^2 = 0.00$; $Chi^2 = 0.74$, df = 2 (P = 0.69); $I^2 = 0\%$								
Test for overall effect: Z = 2.89 (P = 0.004)								
**Total (95% CI)**			**46**			**34**	**100.0%**	**7.35 [3.15, 11.54]**
Heterogeneity: $Tau^2 = 0.00$; $Chi^2 = 2.68$, df = 5 (P = 0.75); $I^2 = 0\%$								
Test for overall effect: Z = 3.43 (P = 0.0006)								
Test for subgroup differences: $Chi^2 = 1.25$, df = 1 (P = 0.26), $I^2 = 20.2\%$								

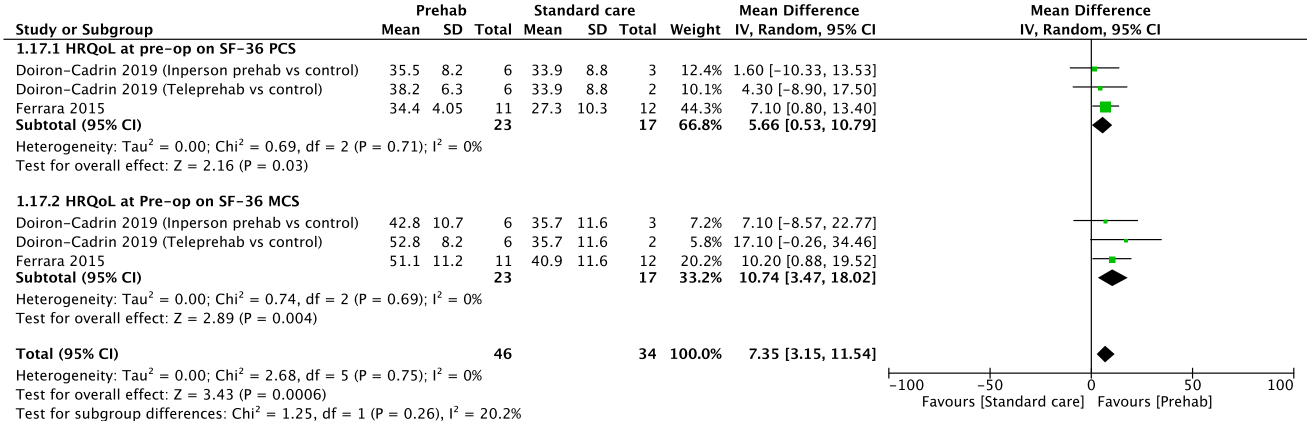

© 2023 Punnoose A et al. *JAMA Network Open*.

eFigure 8. HRQOL: Preoperative and 3 Months Postoperative for Spinal Surgery

	Experimental			Control				Std. Mean Difference
Study or Subgroup	Mean	SD	Total	Mean	SD	Total	Weight	IV, Random, 95% CI
**6.1.1 HRQoL at pre-op**								
Lotzke 2019	0.6	0.32	48	0.42	0.42	41	59.5%	0.48 [0.06, 0.91]
Nielsen 2010	0.85	0.06	28	0.82	0.08	32	40.5%	0.41 [−0.10, 0.93]
**Subtotal (95% CI)**			**76**			**73**	**100.0%**	**0.46 [0.13, 0.78]**
Heterogeneity: $Tau^2 = 0.00$; $Chi^2 = 0.04$, df = 1 (P = 0.84); $I^2 = 0\%$								
Test for overall effect: Z = 2.73 (P = 0.006)								
**6.1.2 HRQoL at 3 months**								
Lindback 2017	0.64	0.26	99	0.64	0.25	98	58.7%	0.00 [−0.28, 0.28]
Lotzke 2019	0.71	0.21	43	0.67	0.23	41	24.9%	0.18 [−0.25, 0.61]
Nielsen 2010	0.88	0.07	28	0.85	0.09	28	16.4%	0.37 [−0.16, 0.90]
**Subtotal (95% CI)**			**170**			**167**	**100.0%**	**0.11 [−0.11, 0.32]**
Heterogeneity: $Tau^2 = 0.00$; $Chi^2 = 1.60$, df = 2 (P = 0.45); $I^2 = 0\%$								
Test for overall effect: Z = 0.96 (P = 0.34)								

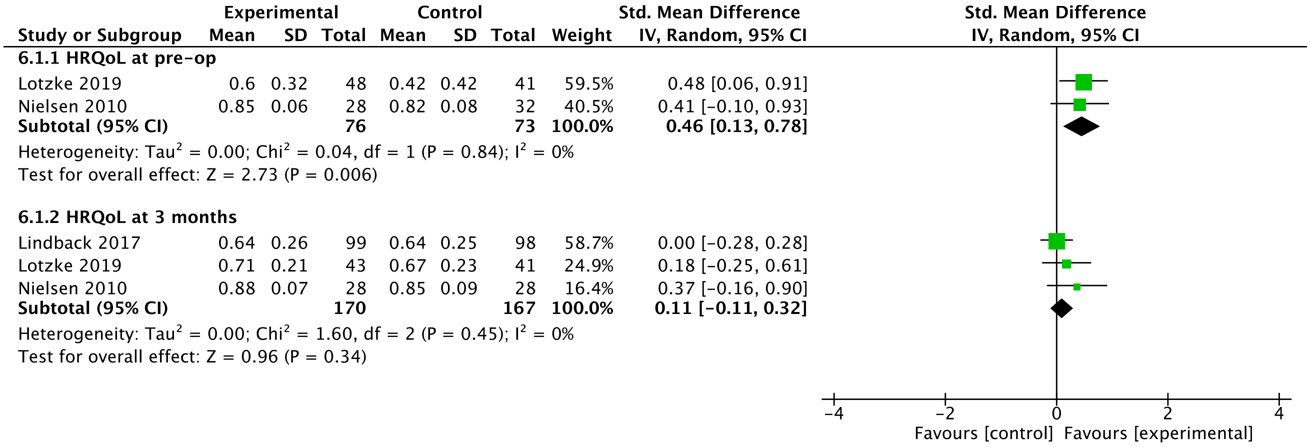

© 2023 Punnoose A et al. *JAMA Network Open*.

eFigure 9. Isometric Hip Abductor Strength: Preoperative for THR

Study or Subgroup	Prehab Mean	Prehab SD	Prehab Total	Standard care Mean	Standard care SD	Standard care Total	Weight	Std. Mean Difference IV, Random, 95% CI
Ferrara 2015	4.68	0.46	11	3.9	0.46	12	40.8%	1.63 [0.67, 2.60]
Villadsen 2013	20	3.6	43	16.9	6.26	41	59.2%	0.61 [0.17, 1.04]
**Total (95% CI)**			**54**			**53**	**100.0%**	**1.03 [0.03, 2.02]**

Heterogeneity: $Tau^2 = 0.38$; $Chi^2 = 3.60$, df = 1 (P = 0.06); $I^2 = 72\%$
Test for overall effect: Z = 2.03 (P = 0.04)

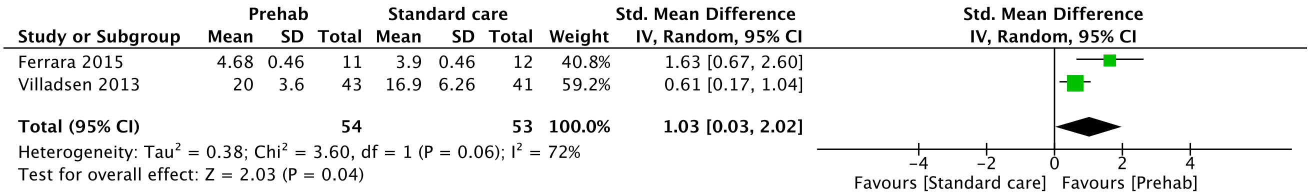

© 2023 Punnoose A et al. *JAMA Network Open*.

eFigure 10. Knee Flexor Strength: Preoperative for TKR

Study or Subgroup	Prehabilitation Mean	SD	Total	Standard care Mean	SD	Total	Weight	Std. Mean Difference IV, Random, 95% CI
**1.11.1 Isometric Knee flexor strength at pre-op**								
Beaupre 2004	7.25	2.72	42	7.71	3.17	49	12.5%	−0.15 [−0.57, 0.26]
Calatayud 2017	17.6	1.24	22	8.2	1.35	22	8.1%	7.12 [5.45, 8.79]
Huber 2015	175.6	81.16	21	153.3	82.37	19	12.0%	0.27 [−0.36, 0.89]
Skoffer 2016	0.8	0.3	29	0.6	0.2	26	12.2%	0.77 [0.22, 1.31]
Van Leeuwen 2014	43	19	10	50	24	8	10.9%	−0.31 [−1.25, 0.62]
**Subtotal (95% CI)**			**124**			**124**	**55.7%**	**1.28 [−0.01, 2.57]**
Heterogeneity: Tau² = 1.96; Chi² = 73.23, df = 4 (P < 0.00001); I² = 95%								
Test for overall effect: Z = 1.94 (P = 0.05)								
**1.11.2 Isokinetic Knee flexor strength at pre-op**								
Franz 2022 (Prehab+BFR vs Standard care)	19.1	4.8	10	11	2.9	5	9.5%	1.77 [0.47, 3.07]
Franz 2022 (Prehab+Sham BFR vs Standard care	9.8	3.6	10	11	2.9	5	10.3%	−0.33 [−1.41, 0.75]
Skoffer 2016	0.6	0.3	29	0.5	0.2	26	12.2%	0.38 [−0.15, 0.92]
Swank 2011	32.3	3.7	35	27.1	3.8	36	12.3%	1.37 [0.85, 1.89]
**Subtotal (95% CI)**			**84**			**72**	**44.3%**	**0.78 [−0.02, 1.59]**
Heterogeneity: Tau² = 0.49; Chi² = 13.29, df = 3 (P = 0.004); I² = 77%								
Test for overall effect: Z = 1.91 (P = 0.06)								
**Total (95% CI)**			**208**			**196**	**100.0%**	**1.00 [0.23, 1.77]**
Heterogeneity: Tau² = 1.19; Chi² = 91.31, df = 8 (P < 0.00001); I² = 91%								
Test for overall effect: Z = 2.53 (P = 0.01)								
Test for subgroup differences: Chi² = 0.40, df = 1 (P = 0.53), I² = 0%								

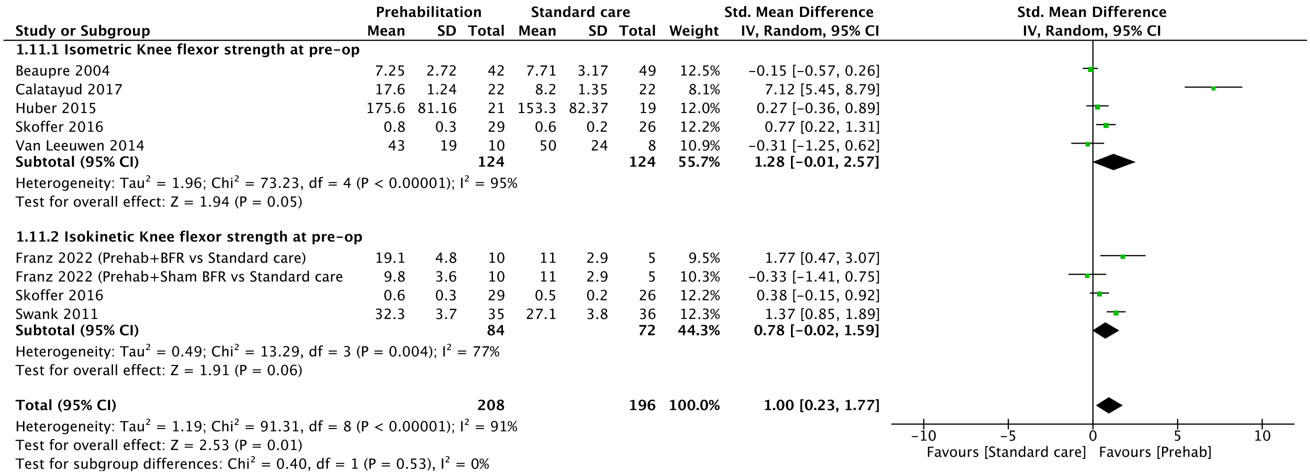

© 2023 Punnoose A et al. *JAMA Network Open*.

eFigure 11. Knee Extensor Strength: Preoperative for TKR

	Prehabilitation			Standard care				Std. Mean Difference
**Study or Subgroup**	**Mean**	**SD**	**Total**	**Mean**	**SD**	**Total**	**Weight**	**IV, Random, 95% CI**
**1.17.1 Isometric Knee extensor strength at pre-op**								
Beaupre 2004	9.97	3.62	42	10.88	4.98	49	6.3%	−0.20 [−0.62, 0.21]
Calatayud 2017	37.8	7	22	22	7.21	22	5.5%	2.18 [1.42, 2.94]
Dominguez-Navarro 2020 (ST vs Control)	141.9	42	24	107.2	33.1	10	5.5%	0.85 [0.08, 1.62]
Dominguez-Navarro 2020 (STB vs Control)	152.3	40.8	20	107.2	33.1	11	5.5%	1.15 [0.35, 1.94]
Huber 2015	255	45.81	21	249	31.94	19	5.9%	0.15 [−0.47, 0.77]
NCT01844934	33.36	41.91	9	23.9	33.04	10	5.2%	0.24 [−0.66, 1.15]
Risso 2022	211.9	62.8	9	171.7	44.9	12	5.2%	0.73 [−0.17, 1.62]
Savkin 2021	103.66	14.39	20	95.08	22.9	20	5.9%	0.44 [−0.19, 1.07]
Skoffer 2016	1.2	0.4	29	1	0.4	26	6.1%	0.49 [−0.05, 1.03]
Van Leeuwen 2014	111	50	10	121	50	8	5.1%	−0.19 [−1.12, 0.74]
Villadsen 2013	43.8	3.61	41	33.3	3.37	40	5.8%	2.98 [2.34, 3.62]
**Subtotal (95% CI)**			**247**			**227**	**61.9%**	**0.80 [0.19, 1.42]**
Heterogeneity: $Tau^2 = 0.95$; $Chi^2 = 91.61$, df = 10 (P < 0.00001); $I^2 = 89\%$								
Test for overall effect: Z = 2.55 (P = 0.01)								
**1.17.2 Isokinetic Knee extensor strength at Pre-op**								
An 2021 (Pre-op education vs standard care)	42.77	9.72	17	37.94	15.61	9	5.4%	0.39 [−0.43, 1.21]
An 2021 (Tele-prehab vs standard care)	53.07	9.71	18	37.94	15.61	9	5.3%	1.23 [0.36, 2.11]
Franz 2022 (Prehab+BFR vs Standard care)	31.5	10.4	10	20.3	7.1	5	4.5%	1.11 [−0.06, 2.28]
Franz 2022 (Prehab+Sham BFR vs Standard care	16	10.2	10	20.3	7.1	5	4.7%	−0.43 [−1.52, 0.66]
Skoffer 2016	0.9	0.4	29	0.9	0.4	26	6.1%	0.00 [−0.53, 0.53]
Swank 2011	60	5.4	35	50.7	5.5	36	6.0%	1.69 [1.14, 2.23]
Topp 2009	56.5	31.4	26	54.02	31.43	28	6.1%	0.08 [−0.46, 0.61]
**Subtotal (95% CI)**			**145**			**118**	**38.1%**	**0.58 [−0.03, 1.20]**
Heterogeneity: $Tau^2 = 0.53$; $Chi^2 = 30.26$, df = 6 (P < 0.0001); $I^2 = 80\%$								
Test for overall effect: Z = 1.86 (P = 0.06)								
**Total (95% CI)**			**392**			**345**	**100.0%**	**0.72 [0.28, 1.15]**
Heterogeneity: $Tau^2 = 0.73$; $Chi^2 = 122.22$, df = 17 (P < 0.00001); $I^2 = 86\%$								
Test for overall effect: Z = 3.24 (P = 0.001)								
Test for subgroup differences: $Chi^2 = 0.24$, df = 1 (P = 0.62), $I^2 = 0\%$								

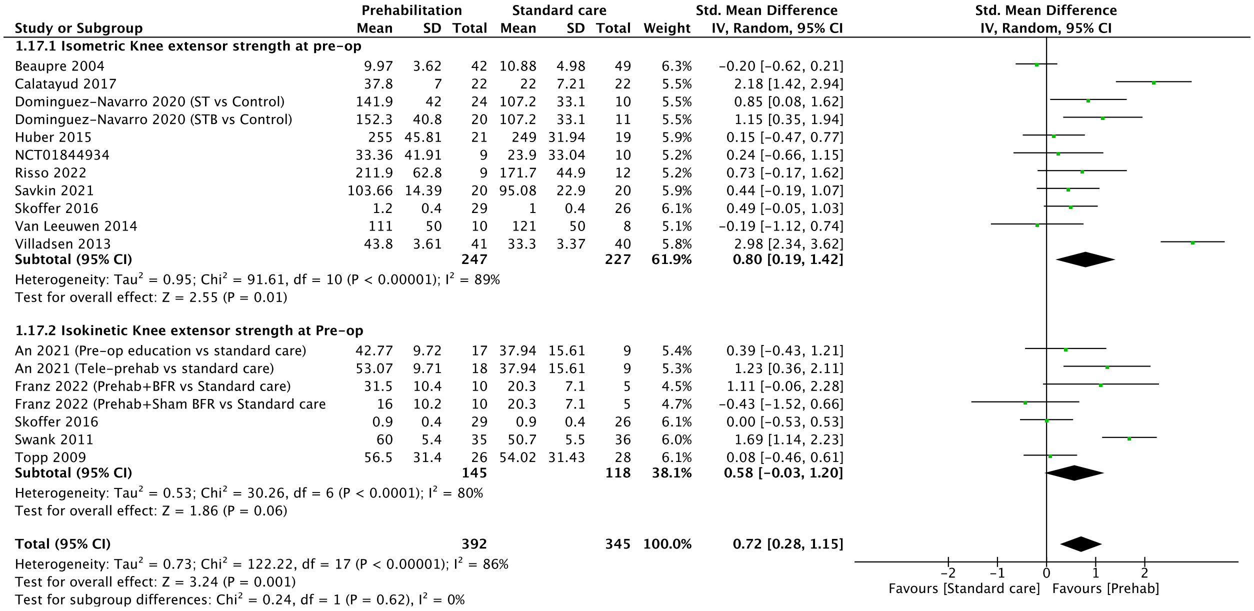

© 2023 Punnoose A et al. *JAMA Network Open*.

eFigure 12. Leg Pain: Preoperative and 3 Months, 6 Months, and 12 Months Postoperative for Spinal Surgery

	Prehabilitation			Control				Mean Difference
**Study or Subgroup**	**Mean**	**SD**	**Total**	**Mean**	**SD**	**Total**	**Weight**	**IV, Random, 95% CI**
**4.5.1 Pre-op leg pain**								
Lindback 2017	54.8	4.3	99	59	7	98	47.8%	-4.20 [-5.82, -2.58]
Lotzke 2019	38.4	27.8	48	31.3	25.5	41	18.1%	7.10 [-3.98, 18.18]
Marchand 2022	55	23	29	65	24	26	15.5%	-10.00 [-22.46, 2.46]
Nielsen 2010	40	19.4	28	50.5	23.2	32	18.7%	-10.50 [-21.28, 0.28]
**Subtotal (95% CI)**			**204**			**197**	**100.0%**	**-4.23 [-10.14, 1.67]**
Heterogeneity: Tau^2 = 18.33; Chi^2 = 6.14, df = 3 (P = 0.11); I^2 = 51%								
Test for overall effect: Z = 1.40 (P = 0.16)								
**4.5.2 Leg pain t 3 months post-op**								
Lotzke 2019	12.2	18.3	48	11	16.1	41	28.2%	1.20 [-5.95, 8.35]
Marchand 2022	29	31	24	22	23	23	15.1%	7.00 [-8.56, 22.56]
Nielsen 2010	28.3	23.1	28	28.3	20.1	28	20.8%	0.00 [-11.34, 11.34]
Rolving 2015	31	6.7	58	40	2.7	29	35.9%	-9.00 [-10.98, -7.02]
**Subtotal (95% CI)**			**158**			**121**	**100.0%**	**-1.83 [-9.71, 6.04]**
Heterogeneity: Tau^2 = 44.02; Chi^2 = 12.79, df = 3 (P = 0.005); I^2 = 77%								
Test for overall effect: Z = 0.46 (P = 0.65)								
**4.5.3 Leg pain at 6 months post-op**								
Lotzke 2019	18.7	24.1	48	15.3	19.1	41	22.1%	3.40 [-5.58, 12.38]
Marchand 2022	24	28	24	31	25	19	9.9%	-7.00 [-22.87, 8.87]
Nielsen 2010	22.3	17.2	28	26	15.4	28	23.4%	-3.70 [-12.25, 4.85]
Rolving 2015	35	3.5	58	43	8.9	29	44.6%	-8.00 [-11.36, -4.64]
**Subtotal (95% CI)**			**158**			**117**	**100.0%**	**-4.37 [-9.89, 1.15]**
Heterogeneity: Tau^2 = 14.87; Chi^2 = 5.83, df = 3 (P = 0.12); I^2 = 49%								
Test for overall effect: Z = 1.55 (P = 0.12)								
**4.5.4 Leg pain at 12 months post-op**								
Lindback 2017	30.3	14.5	99	27.5	16.6	98	50.1%	2.80 [-1.55, 7.15]
Rolving 2015	35	3.5	55	50	11.9	28	49.9%	-15.00 [-19.50, -10.50]
**Subtotal (95% CI)**			**154**			**126**	**100.0%**	**-6.09 [-23.53, 11.35]**
Heterogeneity: Tau^2 = 153.31; Chi^2 = 31.01, df = 1 (P < 0.00001); I^2 = 97%								
Test for overall effect: Z = 0.68 (P = 0.49)								

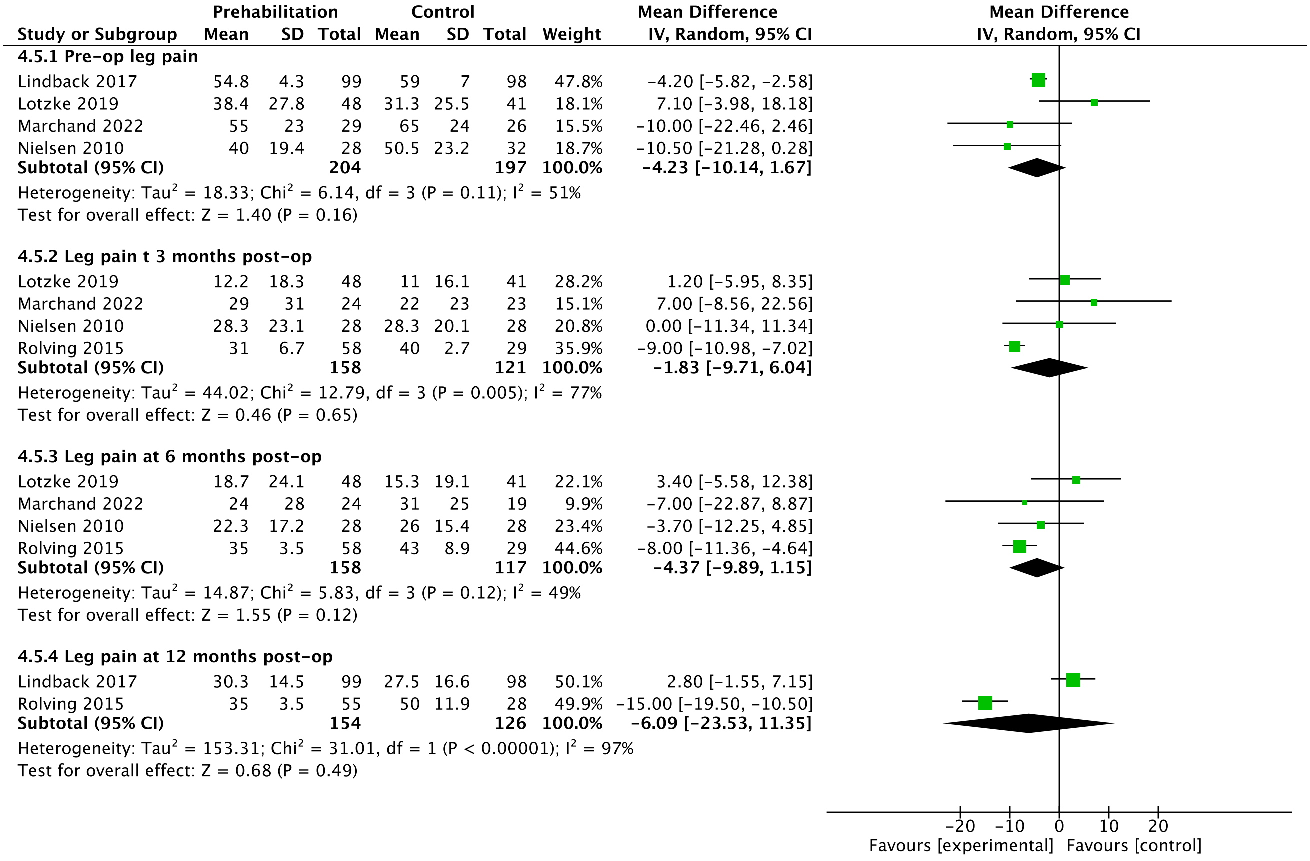

© 2023 Punnoose A et al. *JAMA Network Open*.

eFigure 13. Anxiety and Depression on Hospital Anxiety and Depression Scale (HADS): Preoperative and 3 Months Postoperative for TKR

Study or Subgroup	Prehabilitation Mean	Prehabilitation SD	Prehabilitation Total	Standard care Mean	Standard care SD	Standard care Total	Weight	Mean Difference IV, Random, 95% CI
**1.11.1 Anxiety on HADS at Pre-op**								
Soni 2012	6.7	4.3	21	6.4	3.9	20	29.1%	0.30 [-2.21, 2.81]
Williamson 2007 - (Acupuncture vs Control)	6.45	4.52	44	6.56	3.88	17	35.4%	-0.11 [-2.39, 2.17]
Williamson 2007- (Exercises vs Control)	6.32	4.59	41	6.56	3.88	18	35.4%	-0.24 [-2.52, 2.04]
**Subtotal (95% CI)**			**106**			**55**	**100.0%**	**-0.04 [-1.39, 1.32]**
Heterogeneity: $Tau^2 = 0.00$; $Chi^2 = 0.10$, df = 2 (P = 0.95); $I^2 = 0\%$								
Test for overall effect: Z = 0.05 (P = 0.96)								
**1.11.2 Depression on HADS at Pre-op**								
Soni 2012	6.4	4.8	21	6.2	2.9	20	21.0%	0.20 [-2.21, 2.61]
Williamson 2007 - (Acupuncture vs Control)	6.8	3.32	44	7.08	3.01	17	40.7%	-0.28 [-2.01, 1.45]
Williamson 2007- (Exercises vs Control)	6.62	3.68	41	7.08	3.01	18	38.3%	-0.46 [-2.25, 1.33]
**Subtotal (95% CI)**			**106**			**55**	**100.0%**	**-0.25 [-1.35, 0.86]**
Heterogeneity: $Tau^2 = 0.00$; $Chi^2 = 0.19$, df = 2 (P = 0.91); $I^2 = 0\%$								
Test for overall effect: Z = 0.44 (P = 0.66)								
**1.11.3 Anxiety on HADS at 3 months**								
Soni 2012	5	4.8	21	5.2	4	20	23.5%	-0.20 [-2.90, 2.50]
Williamson 2007 - (Acupuncture vs Control)	2.67	3.56	28	2.42	2.39	10	43.5%	0.25 [-1.73, 2.23]
Williamson 2007- (Exercises vs Control)	4.26	4.04	23	2.42	2.39	9	33.1%	1.84 [-0.43, 4.11]
**Subtotal (95% CI)**			**72**			**39**	**100.0%**	**0.67 [-0.64, 1.98]**
Heterogeneity: $Tau^2 = 0.00$; $Chi^2 = 1.59$, df = 2 (P = 0.45); $I^2 = 0\%$								
Test for overall effect: Z = 1.01 (P = 0.31)								
**1.11.4 Depression on HADS at 3 months**								
Soni 2012	4.9	4.1	21	5.2	4.2	20	24.9%	-0.30 [-2.84, 2.24]
Williamson 2007 - (Acupuncture vs Control)	2.63	2.1	28	3.68	2.93	10	41.2%	-1.05 [-3.03, 0.93]
Williamson 2007- (Exercises vs Control)	3.43	2.54	23	3.68	2.93	9	33.9%	-0.25 [-2.43, 1.93]
**Subtotal (95% CI)**			**72**			**39**	**100.0%**	**-0.59 [-1.86, 0.68]**
Heterogeneity: $Tau^2 = 0.00$; $Chi^2 = 0.35$, df = 2 (P = 0.84); $I^2 = 0\%$								
Test for overall effect: Z = 0.92 (P = 0.36)								

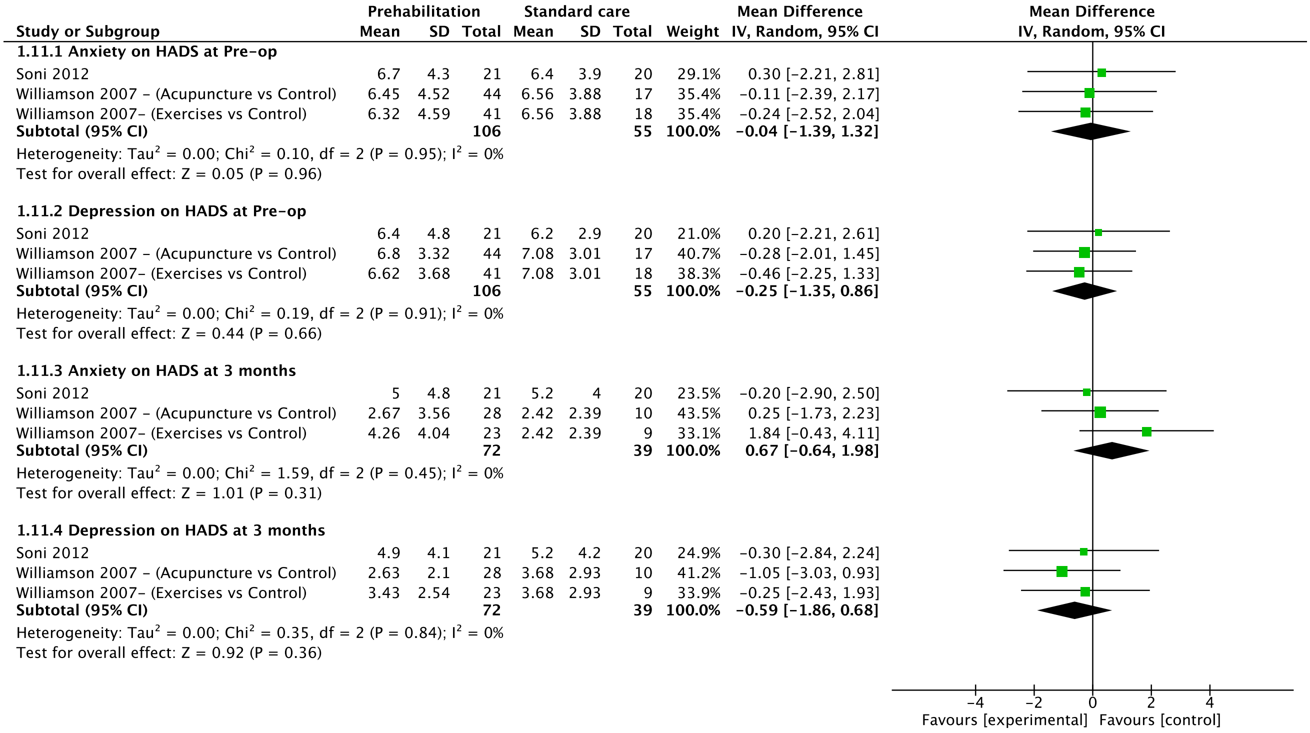

© 2023 Punnoose A et al. *JAMA Network Open*.

eFigure 14. Depression: Preoperative and 3 Months Postoperative for Spinal Surgery

	Experimental			Control				Std. Mean Difference
**Study or Subgroup**	**Mean**	**SD**	**Total**	**Mean**	**SD**	**Total**	**Weight**	**IV, Random, 95% CI**
**7.1.1 Depression at pre-op**								
Lindback 2017	4.4	0.74	90	5.4	1.36	93	54.8%	-0.91 [-1.21, -0.60]
Marchand 2022	4.3	5.3	29	5.3	5.2	26	45.2%	-0.19 [-0.72, 0.34]
**Subtotal (95% CI)**			**119**			**119**	**100.0%**	**-0.58 [-1.28, 0.12]**
Heterogeneity: $Tau^2 = 0.21$; $Chi^2 = 5.29$, df = 1 (P = 0.02); $I^2 = 81\%$								
Test for overall effect: Z = 1.63 (P = 0.10)								
**7.1.2 Depression at 3 months post-op**								
Lindback 2017	3.5	0.74	91	3.4	1.36	91	72.1%	0.09 [-0.20, 0.38]
Marchand 2022	2.7	3.5	24	3.9	4.6	23	27.9%	-0.29 [-0.86, 0.29]
**Subtotal (95% CI)**			**115**			**114**	**100.0%**	**-0.02 [-0.35, 0.32]**
Heterogeneity: $Tau^2 = 0.02$; $Chi^2 = 1.34$, df = 1 (P = 0.25); $I^2 = 25\%$								
Test for overall effect: Z = 0.09 (P = 0.93)								

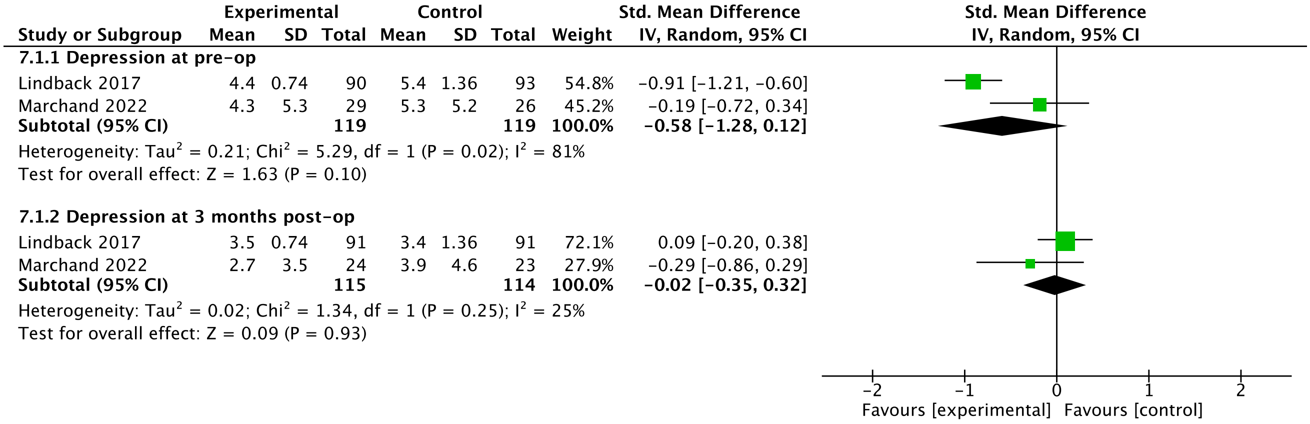

© 2023 Punnoose A et al. *JAMA Network Open*.

eFigure 15. HRQOL: Preoperative for TKR on SF-36

Study or Subgroup	Prehabilitation Mean	SD	Total	Standard care Mean	SD	Total	Weight	Mean Difference IV, Random, 95% CI
**1.12.1 SF-36 PCS at Pre-op**								
Beaupre 2004	32	6	51	31	6	58	14.9%	1.00 [-1.26, 3.26]
Calatayud 2017	49	2.48	22	40	2.48	22	15.3%	9.00 [7.53, 10.47]
Doiron-Cadrin 2019 (In-person prehab vs Control)	34.1	5.3	5	35.4	6	3	9.5%	-1.30 [-9.53, 6.93]
Doiron-Cadrin 2019 (Tele-prehab vs Control)	38.4	5.4	6	35.4	6	3	9.6%	3.00 [-5.05, 11.05]
McKay 2012	29.66	7.99	10	25.61	5.77	12	11.7%	4.05 [-1.88, 9.98]
Walls 2010	44.4	18.7	9	45.6	17.8	5	3.3%	-1.20 [-21.02, 18.62]
**Subtotal (95% CI)**			**103**			**103**	**64.2%**	**3.36 [-1.46, 8.17]**
Heterogeneity: $Tau^2 = 24.21$; $Chi^2 = 39.24$, df = 5 (P < 0.00001); $I^2 = 87\%$								
Test for overall effect: Z = 1.37 (P = 0.17)								
**1.12.2 SF-36 MCS at Pre-op**								
Beaupre 2004	53	10	51	56	11	58	13.6%	-3.00 [-6.94, 0.94]
Doiron-Cadrin 2019 (In-person prehab vs Control)	43	12.3	5	33.6	8.1	3	5.3%	9.40 [-4.75, 23.55]
Doiron-Cadrin 2019 (Tele-prehab vs Control)	41.3	10.5	6	33.6	8.1	3	6.3%	7.70 [-4.73, 20.13]
McKay 2012	52.76	7.79	10	42.28	15.28	12	8.0%	10.48 [0.58, 20.38]
Walls 2010	62.3	17.5	9	68.6	23.1	5	2.5%	-6.30 [-29.55, 16.95]
**Subtotal (95% CI)**			**81**			**81**	**35.8%**	**3.82 [-3.77, 11.41]**
Heterogeneity: $Tau^2 = 40.16$; $Chi^2 = 9.98$, df = 4 (P = 0.04); $I^2 = 60\%$								
Test for overall effect: Z = 0.99 (P = 0.32)								
**Total (95% CI)**			**184**			**184**	**100.0%**	**3.39 [-0.65, 7.42]**
Heterogeneity: $Tau^2 = 27.17$; $Chi^2 = 61.04$, df = 10 (P < 0.00001); $I^2 = 84\%$								
Test for overall effect: Z = 1.65 (P = 0.10)								
Test for subgroup differences: $Chi^2 = 0.01$, df = 1 (P = 0.92), $I^2 = 0\%$								

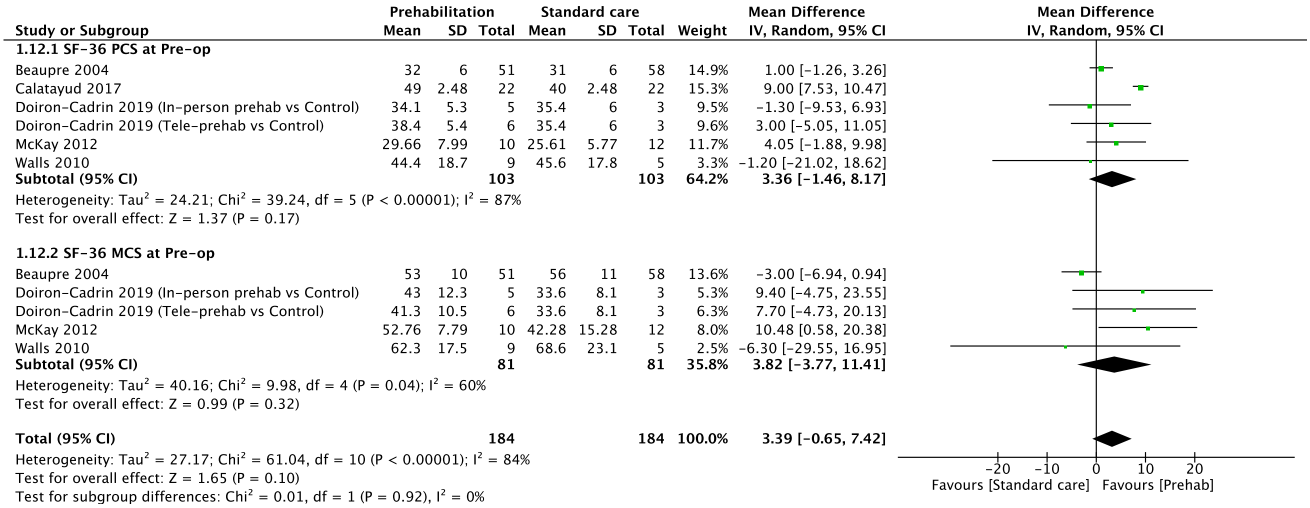

© 2023 Punnoose A et al. *JAMA Network Open*.

eFigure 16. Isometric Hip Extensor Strength: Preoperative for THR

	Prehab			Standard care				Std. Mean Difference
Study or Subgroup	Mean	SD	Total	Mean	SD	Total	Weight	IV, Random, 95% CI
Hermann 2015	143.98	52.87	39	115.78	50.79	40	49.2%	0.54 [0.09, 0.99]
Villadsen 2013	20.1	16.28	43	20.2	11.04	41	50.8%	-0.01 [-0.43, 0.42]
**Total (95% CI)**			**82**			**81**	**100.0%**	**0.26 [-0.27, 0.80]**

Heterogeneity: $Tau^2 = 0.10$; $Chi^2 = 2.97$, df = 1 (P = 0.08); $I^2 = 66\%$
Test for overall effect: Z = 0.96 (P = 0.34)

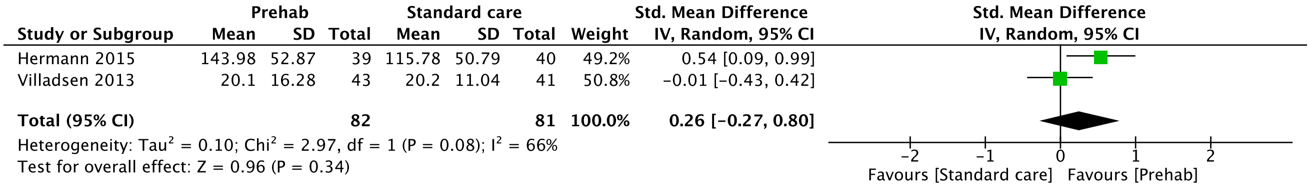

© 2023 Punnoose A et al. *JAMA Network Open*.

eFigure 17. HRQOL on KOOS: 6 Weeks Postoperative for TKR

Study or Subgroup	Prehabilitation Mean	Prehabilitation SD	Prehabilitation Total	Standard care Mean	Standard care SD	Standard care Total	Weight	Mean Difference IV, Random, 95% CI
Dominguez-Navarro 2020 (ST vs Control)	56	8.1	24	55.4	17.9	10	9.8%	0.60 [−10.96, 12.16]
Dominguez-Navarro 2020 (STB vs Control)	57.6	14	20	55.4	17.9	11	8.8%	2.20 [−10.03, 14.43]
Huber 2015	51.9	28.29	21	46.2	27.96	21	4.5%	5.70 [−11.31, 22.71]
Mat Eil-Ismail 2016	55.49	13.16	24	51.76	13.22	26	24.4%	3.73 [−3.59, 11.05]
Savkin 2021	62.81	23.95	20	54.06	25.54	20	5.6%	8.75 [−6.59, 24.09]
Skoffer 2016	56.3	20.6	29	50	13.3	23	15.3%	6.30 [−2.96, 15.56]
Villadsen 2013	50.1	16.31	41	41.3	13.06	40	31.7%	8.80 [2.37, 15.23]
**Total (95% CI)**			**179**			**151**	**100.0%**	**5.66 [2.04, 9.27]**

Heterogeneity: $Tau^2 = 0.00$; $Chi^2 = 2.40$, df = 6 (P = 0.88); $I^2 = 0\%$

Test for overall effect: Z = 3.06 (P = 0.002)

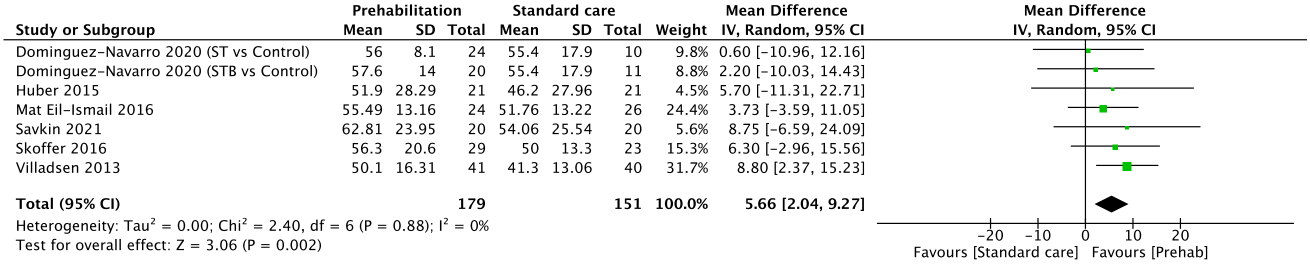

© 2023 Punnoose A et al. *JAMA Network Open*.

eFigure 18. HRQOL: 3 Months Postoperative for TKR on SF-36

Study or Subgroup	Prehabilitation Mean	Prehabilitation SD	Prehabilitation Total	Standard care Mean	Standard care SD	Standard care Total	Weight	Mean Difference IV, Random, 95% CI
**1.14.1 HRQoL on SF-36 PCS**								
Beaupre 2004	35	9	51	35	8	58	15.1%	0.00 [−3.22, 3.22]
Calatayud 2017	55.7	2.48	22	53	2.48	22	72.6%	2.70 [1.23, 4.17]
McKay 2012	41.25	10.06	5	34.83	9.78	5	1.0%	6.42 [−5.88, 18.72]
Walls 2010	35.7	17	9	39	9.4	5	0.8%	−3.30 [−17.13, 10.53]
**Subtotal (95% CI)**			**87**			**90**	**89.5%**	**2.05 [0.37, 3.73]**
Heterogeneity: $Tau^2 = 0.44$; $Chi^2 = 3.30$, df = 3 (P = 0.35); $I^2 = 9\%$								
Test for overall effect: Z = 2.40 (P = 0.02)								
**1.14.2 HRQoL on SF-36 MCS at 3 months post-op**								
Beaupre 2004	54	10	51	55	11	58	10.0%	−1.00 [−4.94, 2.94]
McKay 2012	48.02	17.45	4	51.46	16.37	5	0.3%	−3.44 [−25.76, 18.88]
Walls 2010	58.6	26	4	58.8	19.5	2	0.1%	−0.20 [−37.34, 36.94]
**Subtotal (95% CI)**			**59**			**65**	**10.5%**	**−1.06 [−4.93, 2.80]**
Heterogeneity: $Tau^2 = 0.00$; $Chi^2 = 0.05$, df = 2 (P = 0.98); $I^2 = 0\%$								
Test for overall effect: Z = 0.54 (P = 0.59)								
**Total (95% CI)**			**146**			**155**	**100.0%**	**1.89 [0.64, 3.14]**
Heterogeneity: $Tau^2 = 0.00$; $Chi^2 = 5.86$, df = 6 (P = 0.44); $I^2 = 0\%$								
Test for overall effect: Z = 2.96 (P = 0.003)								
Test for subgroup differences: $Chi^2 = 2.10$, df = 1 (P = 0.15), $I^2 = 52.4\%$								

Mean Difference
IV, Random, 95% CI

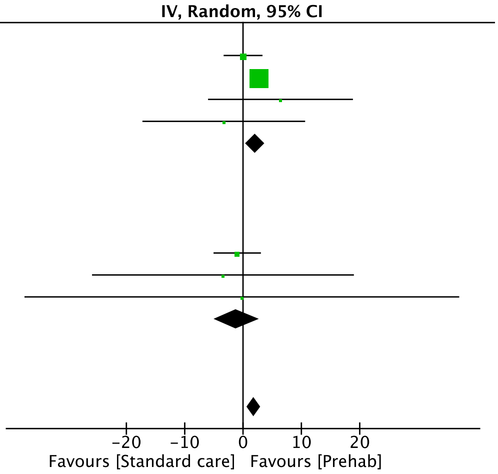

© 2023 Punnoose A et al. *JAMA Network Open*.

eFigure 19. Knee Flexor Strength: 6 Weeks Postoperative for TKR

Study or Subgroup	Prehabilitation Mean	SD	Total	Standard care Mean	SD	Total	Weight	Std. Mean Difference IV, Random, 95% CI
Skoffer 2016	0.7	0.3	29	0.5	0.2	23	75.4%	0.76 [0.19, 1.32]
Van Leeuwen 2014	37	18	10	26	16	7	24.6%	0.61 [−0.39, 1.60]
**Total (95% CI)**			**39**			**30**	**100.0%**	**0.72 [0.23, 1.21]**

Heterogeneity: $Tau^2 = 0.00$; $Chi^2 = 0.07$, df = 1 (P = 0.80); $I^2 = 0\%$
Test for overall effect: Z = 2.86 (P = 0.004)

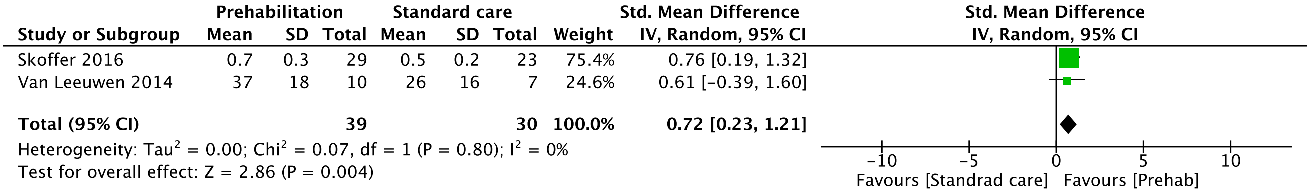

© 2023 Punnoose A et al. *JAMA Network Open*.

eFigure 20. Knee Extensor Strength: 6 Weeks Postoperative

Study or Subgroup	Prehabilitation Mean	SD	Total	Standard care Mean	SD	Total	Weight	Std. Mean Difference IV, Random, 95% CI
**1.19.1 Isometric Knee extensor strength at 6 weeks post-op**								
Dominguez-Navarro 2020 (ST vs Control)	135.7	53.3	24	118.5	34	10	9.2%	0.35 [−0.40, 1.09]
Dominguez-Navarro 2020 (STB vs Control)	118.7	37.9	20	118.5	34	11	9.2%	0.01 [−0.73, 0.74]
McKay 2012	0.6	0.39	9	0.57	0.29	10	7.9%	0.08 [−0.82, 0.99]
NCT03044028	1.51	20.7	11	−2.48	16.53	16	8.9%	0.21 [−0.56, 0.98]
Savkin 2021	93.41	24.69	20	95.84	19.96	20	10.2%	−0.11 [−0.73, 0.51]
Skoffer 2016	0.9	0.3	29	0.6	0.3	23	10.5%	0.98 [0.40, 1.57]
Van Leeuwen 2014	63	30	10	70	35	8	7.7%	−0.21 [−1.14, 0.73]
**Subtotal (95% CI)**			**123**			**98**	**63.7%**	**0.24 [−0.10, 0.58]**
Heterogeneity: $Tau^2 = 0.07; Chi^2 = 8.94, df = 6 (P = 0.18); I^2 = 33\%$								
Test for overall effect: Z = 1.36 (P = 0.17)								
**1.19.2 Isokinetic knee extensor strength at 6 weeks post-op**								
An 2021 (Pre-op education vs standard care)	31.01	6.88	17	23.32	5.15	9	8.1%	1.17 [0.29, 2.05]
An 2021 (Tele-prehab vs standard care)	37.34	5.12	18	23.32	5.15	9	6.5%	2.65 [1.54, 3.76]
Skoffer 2016	0.7	0.2	29	0.6	0.2	23	10.7%	0.49 [−0.06, 1.05]
Topp 2009	43.15	18.5	26	44.41	18.52	28	10.9%	−0.07 [−0.60, 0.47]
**Subtotal (95% CI)**			**90**			**69**	**36.3%**	**0.96 [0.01, 1.91]**
Heterogeneity: $Tau^2 = 0.77; Chi^2 = 20.91, df = 3 (P = 0.0001); I^2 = 86\%$								
Test for overall effect: Z = 1.99 (P = 0.05)								
**Total (95% CI)**			**213**			**167**	**100.0%**	**0.45 [0.06, 0.84]**
Heterogeneity: $Tau^2 = 0.29; Chi^2 = 31.76, df = 10 (P = 0.0004); I^2 = 69\%$								
Test for overall effect: Z = 2.25 (P = 0.02)								
Test for subgroup differences: $Chi^2 = 2.00, df = 1 (P = 0.16), I^2 = 49.9\%$								

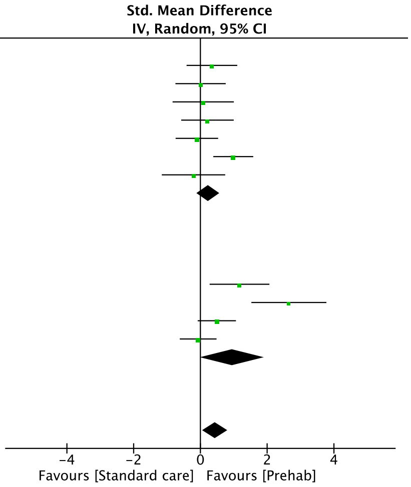

© 2023 Punnoose A et al. *JAMA Network Open*.

eFigure 21. Pain on HOOS: 6 Weeks Postoperative for THR

	Prehab			Standard care				Mean Difference
**Study or Subgroup**	**Mean**	**SD**	**Total**	**Mean**	**SD**	**Total**	**Weight**	**IV, Random, 95% CI**
Oosting 2012	34.5	8.6	12	37.3	16.8	14	21.8%	−2.80 [−12.86, 7.26]
Villadsen 2013	82.2	12.45	43	79.1	11.19	41	78.2%	3.10 [−1.96, 8.16]
**Total (95% CI)**			**55**			**55**	**100.0%**	**1.82 [−2.95, 6.59]**

Heterogeneity: $Tau^2 = 0.91$; $Chi^2 = 1.06$, df = 1 (P = 0.30); $I^2 = 5\%$

Test for overall effect: Z = 0.75 (P = 0.46)

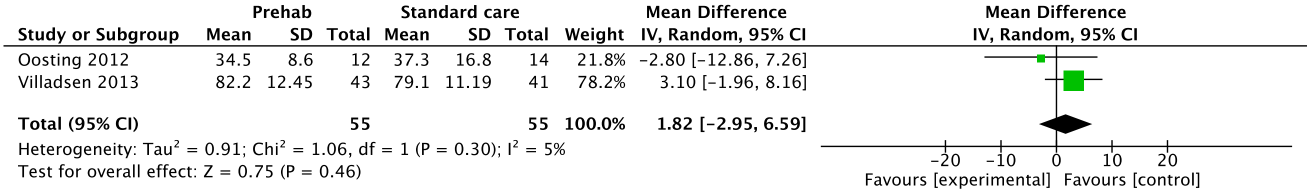

© 2023 Punnoose A et al. *JAMA Network Open*.

eFigure 22. Function: 6 months and 12 months for TKR

Study or Subgroup	Prehabilitation Mean	Prehabilitation SD	Prehabilitation Total	Standard care Mean	Standard care SD	Standard care Total	Weight	Std. Mean Difference IV, Random, 95% CI
**1.5.4 Function at 6 months post-op**								
Beaupre 2004	−78	15	51	−74	15	58	20.4%	−0.26 [−0.64, 0.11]
Franz 2022 (Prehab+BFR vs Standard care)	−71.9	3.1	10	−65.6	4.6	5	6.1%	−1.63 [−2.91, −0.36]
Franz 2022 (Prehab+Sham BFR vs Standard care	−67.8	3.1	10	−65.6	4.6	5	7.6%	−0.57 [−1.67, 0.53]
Jahic 2018	−77.5	2.64	10	−77.5	2.64	10	10.2%	0.00 [−0.88, 0.88]
Riddle 2019 (Education vs Control)	21.6	24.7	106	15.4	24.3	59	21.8%	0.25 [−0.07, 0.57]
Riddle 2019-(CBT vs Control)	22.3	25.17	110	15.4	24.3	60	21.9%	0.28 [−0.04, 0.59]
Rooks 2006	14.5	13.23	14	2.05	17.5	15	12.1%	0.78 [0.02, 1.54]
**Subtotal (95% CI)**			**311**			**212**	**100.0%**	**0.01 [−0.35, 0.37]**
Heterogeneity: $Tau^2 = 0.13$; $Chi^2 = 17.31$, df = 6 (P = 0.008); $I^2 = 65\%$								
Test for overall effect: Z = 0.07 (P = 0.95)								
**1.5.5 Function at 12 months post-op**								
Beaupre 2004	−77	14	51	−77	16	58	19.4%	0.00 [−0.38, 0.38]
Dominguez-Navarro 2020 (ST vs Control)	−88.1	6.8	24	−87.8	4.6	10	5.0%	−0.05 [−0.78, 0.69]
Dominguez-Navarro 2020 (STB vs Control)	−88.2	5.5	20	−87.8	4.6	11	5.1%	−0.07 [−0.81, 0.66]
Huber 2015	−70.8	15.41	17	−73.6	19.17	19	6.4%	0.16 [−0.50, 0.81]
Jahic 2018	−92	4.22	10	−90	0	10		Not estimable
Riddle 2019 (Education vs Control)	17.2	25.11	113	15.4	25.67	60	28.0%	0.07 [−0.24, 0.38]
Riddle 2019-(CBT vs Control)	17.9	25.29	111	15.4	25.67	62	28.4%	0.10 [−0.21, 0.41]
Skoffer 2016	−87.6	12.3	24	−84.4	11.8	20	7.7%	−0.26 [−0.86, 0.34]
**Subtotal (95% CI)**			**370**			**250**	**100.0%**	**0.03 [−0.13, 0.20]**
Heterogeneity: $Tau^2 = 0.00$; $Chi^2 = 1.45$, df = 6 (P = 0.96); $I^2 = 0\%$								
Test for overall effect: Z = 0.37 (P = 0.71)								

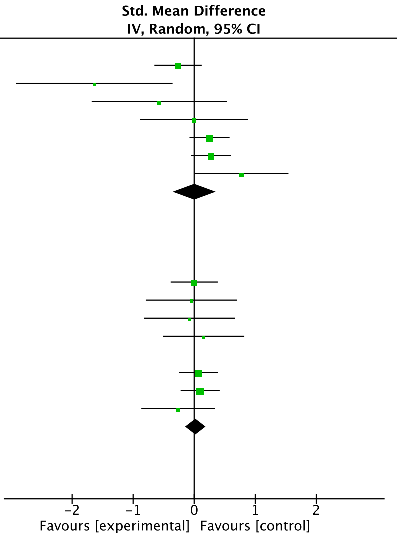

© 2023 Punnoose A et al. *JAMA Network Open*.

eFigure 23. Function on HOOS: 6 Weeks Postoperative for THR

	Prehab			Standard care				Mean Difference
Study or Subgroup	Mean	SD	Total	Mean	SD	Total	Weight	IV, Random, 95% CI
Oosting 2012	36.5	10.8	14	40	13.3	12	31.1%	−3.50 [−12.91, 5.91]
Villadsen 2013	77.7	10.75	41	74	5.48	43	68.9%	3.70 [0.02, 7.38]
**Total (95% CI)**			**55**			**55**	**100.0%**	**1.46 [−5.08, 7.99]**

Heterogeneity: $Tau^2 = 12.63$; $Chi^2 = 1.95$, df = 1 (P = 0.16); $I^2 = 49\%$
Test for overall effect: Z = 0.44 (P = 0.66)

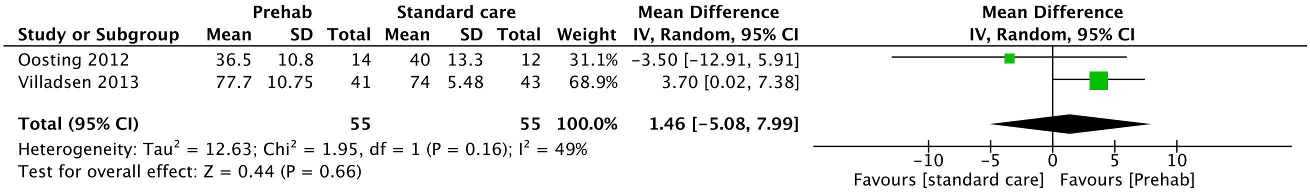

© 2023 Punnoose A et al. *JAMA Network Open*.

eFigure 24. HRQOL on HOOS: 6 Weeks Postoperative for THR

	Prehab			Standard care				Mean Difference
Study or Subgroup	Mean	SD	Total	Mean	SD	Total	Weight	IV, Random, 95% CI
Oosting 2012	53.2	16.4	15	49.2	16.1	13	25.1%	4.00 [−8.06, 16.06]
Villadsen 2013	60.2	18.05	41	54.2	13.8	40	74.9%	6.00 [−0.99, 12.99]
**Total (95% CI)**			**56**			**53**	**100.0%**	**5.50 [−0.55, 11.54]**

Heterogeneity: $Tau^2 = 0.00$; $Chi^2 = 0.08$, $df = 1$ ($P = 0.78$); $I^2 = 0\%$
Test for overall effect: $Z = 1.78$ ($P = 0.07$)

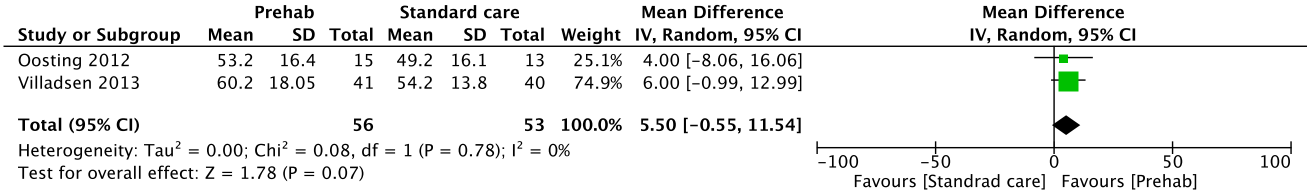

© 2023 Punnoose A et al. *JAMA Network Open*.

eFigure 25. HRQOL: 3 Months Postoperative for THR

Study or Subgroup	Prehab Mean	Prehab SD	Prehab Total	Standard care Mean	Standard care SD	Standard care Total	Weight	Std. Mean Difference IV, Random, 95% CI
Cavill 2016	69	23	11	64	23	12	13.5%	0.21 [−0.61, 1.03]
Hermann 2015	74.58	18.15	36	70.3	22.79	28	37.1%	0.21 [−0.29, 0.70]
Villadsen 2013	63.3	18.05	43	59.8	13.8	41	49.4%	0.22 [−0.21, 0.64]
**Total (95% CI)**			**90**			**81**	**100.0%**	**0.21 [−0.09, 0.51]**

Heterogeneity: $Tau^2 = 0.00$; $Chi^2 = 0.00$, df = 2 (P = 1.00); $I^2 = 0\%$
Test for overall effect: Z = 1.38 (P = 0.17)

Std. Mean Difference
IV, Random, 95% CI

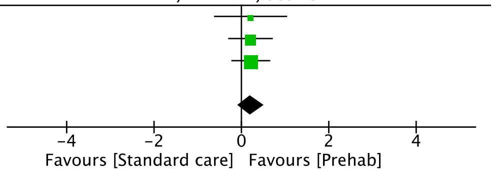

© 2023 Punnoose A et al. *JAMA Network Open*.

eFigure 26. Knee Flexor Strength: 3 Months Postoperative

Study or Subgroup	Prehabilitation Mean	SD	Total	Standard care Mean	SD	Total	Weight	Std. Mean Difference IV, Random, 95% CI
**1.20.1 Isometric Knee flexor strength at 3 months post-op**								
Beaupre 2004	8.16	7	42	8.16	6	49	14.4%	0.00 [−0.41, 0.41]
Calatayud 2017	9.4	1.24	22	4.4	1.35	22	11.4%	3.79 [2.77, 4.81]
Huber 2015	118.3	83.43	21	108.4	86.15	20	13.6%	0.11 [−0.50, 0.73]
Skoffer 2016	0.7	0.3	29	0.6	0.2	21	13.8%	0.37 [−0.19, 0.94]
Van Leeuwen 2014	42	17	9	50	23	7	11.5%	−0.38 [−1.38, 0.62]
**Subtotal (95% CI)**			**123**			**119**	**64.7%**	**0.73 [−0.30, 1.75]**
**1.20.2 Isokinetic knee flexor strength at 3 months post-op**								
Franz 2022 (Prehab+BFR vs Standard care)	14.6	5.3	10	8.8	2.7	5	10.5%	1.17 [−0.01, 2.35]
Franz 2022 (Prehab+Sham BFR vs Standard care	6.4	4.6	10	8.8	2.7	5	11.0%	−0.55 [−1.65, 0.55]
Skoffer 2016	0.5	0.2	29	0.4	0.2	21	13.8%	0.49 [−0.08, 1.06]
**Subtotal (95% CI)**			**49**			**31**	**35.3%**	**0.38 [−0.44, 1.19]**
**Total (95% CI)**			**172**			**150**	**100.0%**	**0.59 [−0.11, 1.28]**

1.20.1 subtotal: Heterogeneity: $Tau^2 = 1.21$; $Chi^2 = 49.18$, df = 4 ($P < 0.00001$); $I^2 = 92\%$
Test for overall effect: Z = 1.39 (P = 0.16)

1.20.2 subtotal: Heterogeneity: $Tau^2 = 0.29$; $Chi^2 = 4.62$, df = 2 (P = 0.10); $I^2 = 57\%$
Test for overall effect: Z = 0.90 (P = 0.37)

Total: Heterogeneity: $Tau^2 = 0.83$; $Chi^2 = 53.86$, df = 7 ($P < 0.00001$); $I^2 = 87\%$
Test for overall effect: Z = 1.66 (P = 0.10)
Test for subgroup differences: $Chi^2 = 0.28$, df = 1 (P = 0.60), $I^2 = 0\%$

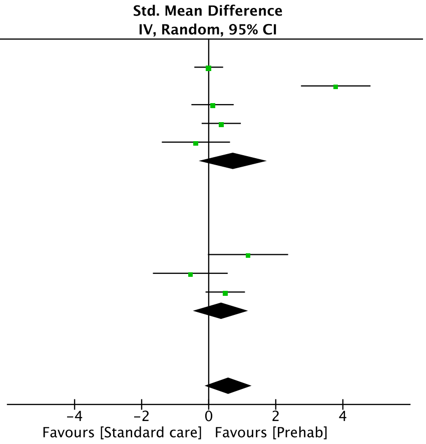

© 2023 Punnoose A et al. *JAMA Network Open*.

eFigure 27. Knee Extensor Strength: 3 Months Postoperative

Study or Subgroup	Prehabilitation Mean	Prehabilitation SD	Prehabilitation Total	Standard care Mean	Standard care SD	Standard care Total	Weight	Std. Mean Difference IV, Random, 95% CI
**1.17.1 Isometric knee extensor strength at 3 months post-op**								
Beaupre 2004	12.24	4.53	42	12.24	3.62	49	12.2%	0.00 [−0.41, 0.41]
Calatayud 2017	22.8	7	22	14.3	7.2	22	9.3%	1.18 [0.53, 1.82]
Huber 2015	197.7	48.32	21	204.1	36.6	20	9.7%	−0.15 [−0.76, 0.47]
McKay 2012	0.6	0.39	7	0.57	0.29	10	6.2%	0.09 [−0.88, 1.05]
NCT01844934	31.82	34.89	9	30.3	31.04	10	6.7%	0.04 [−0.86, 0.94]
Savkin 2021	96.34	25.75	20	98.01	22.79	20	9.6%	−0.07 [−0.69, 0.55]
Skoffer 2016	1	0.3	29	0.8	0.3	21	10.1%	0.66 [0.08, 1.23]
Van Leeuwen 2014	76	34	9	97	40	7	5.9%	−0.54 [−1.55, 0.47]
**Subtotal (95% CI)**			**159**			**159**	**69.7%**	**0.19 [−0.17, 0.55]**
Heterogeneity: $Tau^2 = 0.15$; $Chi^2 = 16.27$, df = 7 (P = 0.02); $I^2 = 57\%$								
Test for overall effect: Z = 1.03 (P = 0.30)								
**1.17.2 Isokinetic Knee extensor strength at 3 months post-op**								
Franz 2022 (Prehab+BFR vs Standard care)	19.1	4.8	10	11	2.9	5	4.2%	1.77 [0.47, 3.07]
Franz 2022 (Prehab+Sham BFR vs Standard care	9.8	3.6	10	11	2.9	5	5.4%	−0.33 [−1.41, 0.75]
Skoffer 2016	0.9	0.3	29	0.7	0.2	21	10.1%	0.75 [0.17, 1.33]
Topp 2009	62.27	25.49	26	60.74	25.45	28	10.7%	0.06 [−0.47, 0.59]
**Subtotal (95% CI)**			**75**			**59**	**30.3%**	**0.48 [−0.21, 1.16]**
Heterogeneity: $Tau^2 = 0.30$; $Chi^2 = 8.95$, df = 3 (P = 0.03); $I^2 = 66\%$								
Test for overall effect: Z = 1.36 (P = 0.17)								
**Total (95% CI)**			**234**			**218**	**100.0%**	**0.27 [−0.04, 0.58]**
Heterogeneity: $Tau^2 = 0.16$; $Chi^2 = 26.16$, df = 11 (P = 0.006); $I^2 = 58\%$								
Test for overall effect: Z = 1.70 (P = 0.09)								
Test for subgroup differences: $Chi^2 = 0.52$, df = 1 (P = 0.47), $I^2 = 0\%$								

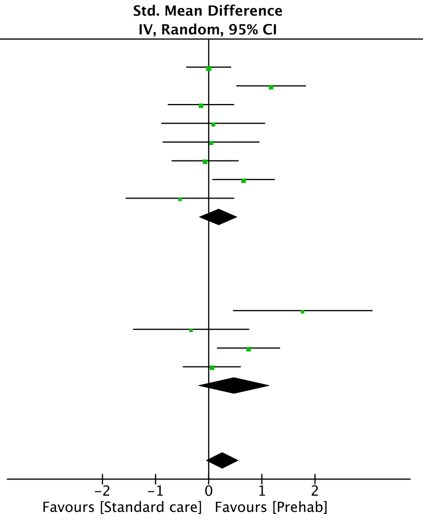

© 2023 Punnoose A et al. *JAMA Network Open*.

eFigure 28. Knee Flexor Strength: 12 Months Postoperative for TRK

	Prehabilitation			Standard care				Std. Mean Difference
**Study or Subgroup**	**Mean**	**SD**	**Total**	**Mean**	**SD**	**Total**	**Weight**	**IV, Random, 95% CI**
Beaupre 2004	9.52	3.62	51	9.52	2.72	58	68.4%	0.00 [−0.38, 0.38]
Skoffer 2016	0.9	0.2	24	0.8	0.3	20	31.6%	0.39 [−0.21, 0.99]
**Total (95% CI)**			**75**			**78**	**100.0%**	**0.12 [−0.23, 0.48]**

Heterogeneity: $Tau^2 = 0.01$; $Chi^2 = 1.18$, df = 1 (P = 0.28); $I^2 = 15\%$
Test for overall effect: Z = 0.68 (P = 0.50)

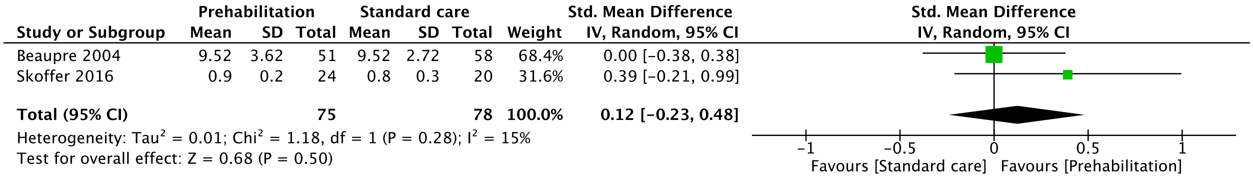

© 2023 Punnoose A et al. *JAMA Network Open*.

eFigure 29. Knee Extensor Strength: 12 Months Postoperative for TRK

Study or Subgroup	Prehabilitation Mean	SD	Total	Standard care Mean	SD	Total	Weight	Std. Mean Difference IV, Random, 95% CI
Beaupre 2004	13.6	4.53	51	13.15	3.62	58	55.9%	0.11 [−0.27, 0.49]
Dominguez-Navarro 2020 (ST vs Control)	158.3	67.2	19	128.3	32.7	8	11.3%	0.49 [−0.35, 1.33]
Dominguez-Navarro 2020 (STB vs Control)	142.8	38.9	15	128.3	32.7	8	10.6%	0.38 [−0.49, 1.24]
Skoffer 2016	1.4	0.3	24	1.3	0.4	20	22.3%	0.28 [−0.32, 0.88]
**Total (95% CI)**			**109**			**94**	**100.0%**	**0.22 [−0.06, 0.50]**

Heterogeneity: $Tau^2 = 0.00$; $Chi^2 = 0.89$, df = 3 (P = 0.83); $I^2 = 0\%$
Test for overall effect: Z = 1.53 (P = 0.13)

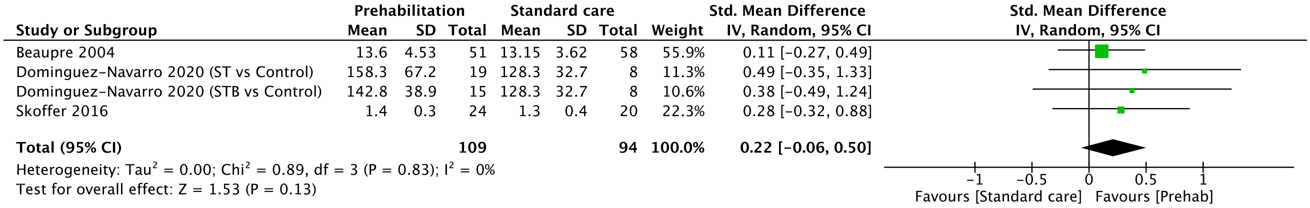

© 2023 Punnoose A et al. *JAMA Network Open*.

eResults. Association of Prehabilitation With Secondary Outcomes

Pre-operative phase (after prehabilitation)

On performing meta-analysis, there was statistically significant difference between groups only for TKR flexion range of motion (ROM) (11 trials, n=736, WMD 4.29, 95% CI 2.02, 6.56; GRADE low (eFigure 30)
Functional performance was measured during various tasks including stair test, timed up and go test (TUG), 30-second sit to stand test, 6-minute walk test (6MWT) and functional reach. Pre-operatively, a statistically significant difference was observed for TKR for: 6MWT (6 trials, n=231, WMD 29.2, 95% CI 22.2,36.2; GRADE moderate (eFigure 31); TUG (7 trials, n=302, WMD -1.38, 95% CI -2.39,-0.37; GRADE low (eFigure 32); Stair test (5 trials, n=140, WMD -3.58, 95% CI -5.04, -2.11 GRADE low (eFigure 33). There were no significant between group differences in any other measures on meta-analysis (eFigure 34 to eFigure 38).

Post-operative phase (after surgery)

A statistically significant difference was only noted in TKR for flexion ROM at 6 weeks post-operatively (5 trials, n=260, WMD 4.96,95% CI 2.51, 7.40; GRADE low (eFigure 30). Post-operatively, there was also a statistically significant difference favoring prehabilitation for TKR for: TUG at 6 weeks (3 trials, n=170, WMD -1.52, 95% CI -2.22,-0.82; GRADE low eFigure 32) and stair test at 3 months (4 trials, n=117, WMD -3.16, 95% CI -5.05, -1.26; GRADE low (eFigure 33).There were no significant between group differences in any other measures on meta-analysis (eFigure 33, eFigure 34-38).

Health economic data

On meta-analysis, there were no statistically significant differences in: Length of stay in THR, TKR or spinal surgeries (eFigure 39 and eFigure 40); healthcare costs in TKR (3 trials, 466 patients, SMD 0.0, 95% CI -0.19,0.18; GRADE low (eFigure 41) and readmission rates in TKR surgery (4 trials, 544 patients, RR 0.83, 95% CI -0.38,1.79; GRADE low (eFigure 42).

Overall complications

There was no statistically significant difference between groups in complication rates in THR, TKR or spinal surgeries (eFigure 43 and eFigure 44).

© 2023 Punnoose A et al. *JAMA Network Open*.

eFigure 30. Active Knee Flexion Range of Motion (ROM): Preoperative and 6 Weeks, 3 Months, and 12 Months Postoperative for TKR

Study or Subgroup	Prehabilitation Mean	Prehabilitation SD	Prehabilitation Total	Standard care Mean	Standard care SD	Standard care Total	Weight	Mean Difference IV, Random, 95% CI
**1.35.1 Knee flexion at Pre-op**								
An 2021 (Pre-op education vs standard care)	110.52	8.8	17	107.18	10.23	9	5.6%	3.34 [−4.54, 11.22]
An 2021 (Tele-prehab vs standard care)	114.41	6.26	18	107.18	10.23	9	6.2%	7.23 [−0.05, 14.51]
Beaupre 2004	109	12	51	105	18	58	8.3%	4.00 [−1.68, 9.68]
Calatayud 2017	114.4	8.79	22	102.8	7.89	22	9.5%	11.60 [6.66, 16.54]
Dominguez-Navarro 2020 (ST vs Control)	112.1	11.5	24	104.1	10.5	10	5.5%	8.00 [0.03, 15.97]
Dominguez-Navarro 2020 (STB vs Control)	110.9	10.6	20	104.1	10.5	11	5.7%	6.80 [−0.95, 14.55]
Evgeniadis 2008	112.5	9.11	18	109.3	12.4	20	6.7%	3.20 [−3.67, 10.07]
Franz 2022 (Prehab+BFR vs Standard care)	116.2	10.8	10	116.4	14.3	5	2.2%	−0.20 [−14.41, 14.01]
Franz 2022 (Prehab+Sham BFR vs Standard care	112.7	3.5	10	116.4	14.3	5	2.7%	−3.70 [−16.42, 9.02]
Huang 2012	92	7	126	91	7	117	15.5%	1.00 [−0.76, 2.76]
Huber 2015	117.9	6.96	21	115.2	5.36	19	11.5%	2.70 [−1.13, 6.53]
NCT01844934	129.44	10.43	9	116.6	12.83	10	3.7%	12.84 [2.37, 23.31]
Savkin 2021	109.55	8.29	20	105.05	10.81	20	7.8%	4.50 [−1.47, 10.47]
Skoffer 2016	120.76	10.8	29	120.86	8.8	26	9.1%	−0.10 [−5.29, 5.09]
**Subtotal (95% CI)**			**395**			**341**	**100.0%**	**4.29 [2.02, 6.56]**
Heterogeneity: Tau² = 7.84; Chi² = 26.72, df = 13 (P = 0.01); I² = 51%								
Test for overall effect: Z = 3.70 (P = 0.0002)								
**1.35.2 Knee flexion at 6 weeks post-op**								
An 2021 (Pre-op education vs standard care)	131.7	3.18	17	127.91	6.73	9	19.7%	3.79 [−0.86, 8.44]
An 2021 (Tele-prehab vs standard care)	138.32	1.79	18	127.91	6.73	9	20.8%	10.41 [5.94, 14.88]
Dominguez-Navarro 2020 (ST vs Control)	103.2	10.4	24	101.6	9.1	10	10.3%	1.60 [−5.41, 8.61]
Dominguez-Navarro 2020 (STB vs Control)	105.2	9.1	20	101.6	9.1	11	11.1%	3.60 [−3.10, 10.30]
Mat Eil-Ismail 2016	94.58	15.47	24	89.23	15.54	26	7.2%	5.35 [−3.25, 13.95]
Savkin 2021	116.25	8.05	20	112.15	6.52	20	20.3%	4.10 [−0.44, 8.64]
Skoffer 2016	108.66	11	29	106.16	13.8	23	10.6%	2.50 [−4.42, 9.42]
**Subtotal (95% CI)**			**152**			**108**	**100.0%**	**4.96 [2.51, 7.40]**
Heterogeneity: Tau² = 2.27; Chi² = 7.60, df = 6 (P = 0.27); I² = 21%								
Test for overall effect: Z = 3.98 (P < 0.0001)								
**1.35.3 Knee flexion at 3 months post-op**								
Beaupre 2004	93	16	51	93	15	58	9.6%	0.00 [−5.85, 5.85]
Calatayud 2017	101.2	7.78	22	96.4	7.89	22	11.6%	4.80 [0.17, 9.43]
Cavill 2016	110.8	10.6	20	98.2	11.1	19	8.2%	12.60 [5.78, 19.42]
Evgeniadis 2008	80.73	6.7	18	80.42	10.2	20	10.2%	0.31 [−5.13, 5.75]
Franz 2022 (Prehab+BFR vs Standard care)	117.9	6	10	114.7	7.1	5	7.7%	3.20 [−4.05, 10.45]
Franz 2022 (Prehab+Sham BFR vs Standard care	113.3	10.1	10	114.7	7.1	5	6.1%	−1.40 [−10.23, 7.43]
Huber 2015	113.1	6.83	21	116.3	5.95	20	12.8%	−3.20 [−7.12, 0.72]
Mat Eil-Ismail 2016	108.75	9.63	24	102.31	9.68	26	10.4%	6.44 [1.08, 11.80]
NCT01844934	117	15	9	118	12.33	10	3.7%	−1.00 [−13.43, 11.43]
Savkin 2021	117.1	8.48	20	116	7.81	20	10.8%	1.10 [−3.95, 6.15]
Skoffer 2016	113.06	14.8	29	112.56	7.81	21	8.9%	0.50 [−5.84, 6.84]
**Subtotal (95% CI)**			**234**			**226**	**100.0%**	**2.17 [−0.52, 4.86]**
Heterogeneity: Tau² = 10.69; Chi² = 22.13, df = 10 (P = 0.01); I² = 55%								
Test for overall effect: Z = 1.58 (P = 0.11)								
**1.35.4 Knee flexion at 12 months post-op**								
Beaupre 2004	99	16	51	103	16	58	36.5%	−4.00 [−10.02, 2.02]
Dominguez-Navarro 2020 (ST vs Control)	105.1	10.2	19	102.9	8.5	8	23.7%	2.20 [−5.27, 9.67]
Dominguez-Navarro 2020 (STB vs Control)	104.8	8.7	15	102.9	8.5	8	24.5%	1.90 [−5.45, 9.25]
Skoffer 2016	117.3	8.4	24	116.4	19.8	20	15.3%	0.90 [−8.41, 10.21]
**Subtotal (95% CI)**			**109**			**94**	**100.0%**	**−0.34 [−3.97, 3.30]**
Heterogeneity: Tau² = 0.00; Chi² = 2.29, df = 3 (P = 0.51); I² = 0%								
Test for overall effect: Z = 0.18 (P = 0.86)								

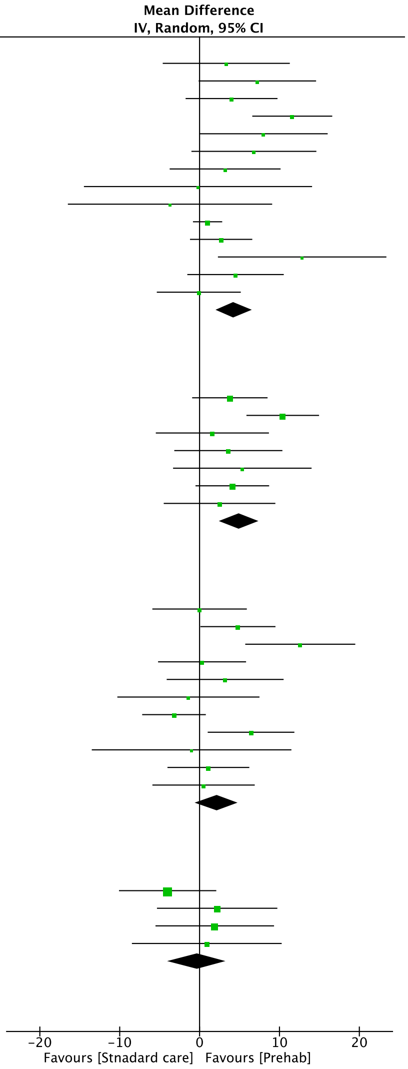

© 2023 Punnoose A et al. *JAMA Network Open*.

eFigure 31. 6-Minute Walk Test (6MWT): Preoperative and 6 Weeks, 3 Months, and 12 Months Postoperative for TKR

Study or Subgroup	Prehabilitation Mean	Prehabilitation SD	Prehabilitation Total	Standard care Mean	Standard care SD	Standard care Total	Weight	Mean Difference IV, Random, 95% CI
**1.71.1 6MWT at Pre-op**								
Franz 2022 (Prehab+BFR vs Standard care)	431	69	10	395	85	5	0.7%	36.00 [-49.91, 121.91]
Franz 2022 (Prehab+Sham BFR vs Standard care	440	70	10	395	85	5	0.7%	45.00 [-41.22, 131.22]
NCT01844934	442.23	93.47	9	386.15	78.12	10	0.8%	56.08 [-21.85, 134.01]
Skoffer 2016	434	101	29	427	76	10	1.4%	7.00 [-52.75, 66.75]
Swank 2011	392.8	15.2	36	363.3	15.6	35	95.4%	29.50 [22.33, 36.67]
Topp 2009	1,282	300.84	26	1,185	296.32	28	0.2%	97.00 [-62.43, 256.43]
Van Leeuwen 2014	471	92	8	493	55	10	0.9%	-22.00 [-94.29, 50.29]
**Subtotal (95% CI)**			**128**			**103**	**100.0%**	**29.20 [22.20, 36.20]**
Heterogeneity: Tau² = 0.00; Chi² = 3.77, df = 6 (P = 0.71); I² = 0%								
Test for overall effect: Z = 8.18 (P < 0.00001)								
**1.71.2 6MWT at 6 weeks post-op**								
Skoffer 2016	424	103	29	376	83	23	56.9%	48.00 [-2.56, 98.56]
Van Leeuwen 2014	380	109	10	440	87	7	43.1%	-60.00 [-153.37, 33.37]
**Subtotal (95% CI)**			**39**			**30**	**100.0%**	**1.43 [-103.41, 106.26]**
Heterogeneity: Tau² = 4364.63; Chi² = 3.97, df = 1 (P = 0.05); I² = 75%								
Test for overall effect: Z = 0.03 (P = 0.98)								
**1.71.4 6MWT at 3 months post-op**								
Franz 2022 (Prehab+BFR vs Standard care)	426	73	10	370	70	5	17.4%	56.00 [-20.23, 132.23]
Franz 2022 (Prehab+Sham BFR vs Standard care	420	60	10	370	70	5	19.5%	50.00 [-21.75, 121.75]
NCT01844934	443.95	365.1	9	422.94	301.93	10	1.2%	21.01 [-282.16, 324.18]
Skoffer 2016	449	94	29	433	74	21	42.9%	16.00 [-30.61, 62.61]
Topp 2009	1,337	295.74	26	1,365	296.32	28	4.2%	-28.00 [-186.02, 130.02]
Van Leeuwen 2014	456	62	9	513	97	7	14.9%	-57.00 [-139.49, 25.49]
**Subtotal (95% CI)**			**93**			**76**	**100.0%**	**16.87 [-15.72, 49.46]**
Heterogeneity: Tau² = 79.82; Chi² = 5.22, df = 5 (P = 0.39); I² = 4%								
Test for overall effect: Z = 1.01 (P = 0.31)								
**1.71.5 6MWT at 12 months post-op**								
Riddle 2019 (Education vs Control)	337	132.87	113	363	132.43	61	37.1%	-26.00 [-67.29, 15.29]
Riddle 2019-(CBT vs Control)	366	134.38	111	363	132.43	61	36.6%	3.00 [-38.59, 44.59]
Skoffer 2016	479	92	24	474	74	20	26.3%	5.00 [-44.06, 54.06]
**Subtotal (95% CI)**			**248**			**142**	**100.0%**	**-7.24 [-32.39, 17.92]**
Heterogeneity: Tau² = 0.00; Chi² = 1.27, df = 2 (P = 0.53); I² = 0%								
Test for overall effect: Z = 0.56 (P = 0.57)								

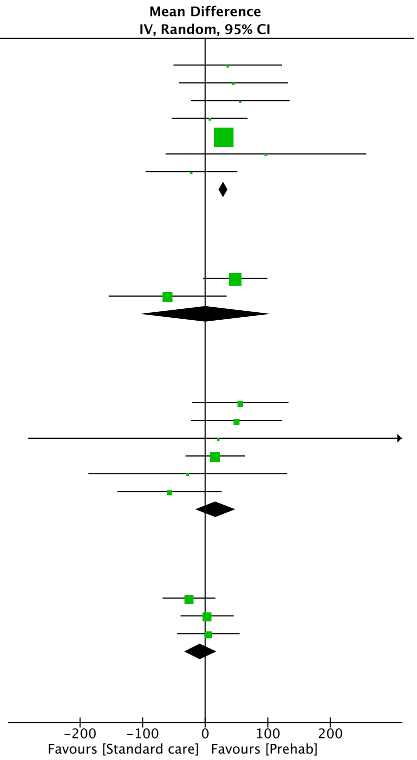

© 2023 Punnoose A et al. *JAMA Network Open*.

eFigure 32. TUG: Preoperative and 6 Weeks, 3 Months, and 12 Months Postoperative for TKR

	Prehabilitation			Standard care				Mean Difference
Study or Subgroup	Mean	SD	Total	Mean	SD	Total	Weight	IV, Random, 95% CI
**1.58.1 TUG at Pre-op**								
An 2021 (Pre-op education vs standard care)	12.16	1.6	17	13.34	2.13	9	13.2%	-1.18 [-2.77, 0.41]
An 2021 (Tele-prehab vs standard care)	10.61	1.26	18	13.34	2.13	9	13.6%	-2.73 [-4.24, -1.22]
Calatayud 2017	6.7	0.78	22	9	0.78	22	18.7%	-2.30 [-2.76, -1.84]
Doiron-Cadrin 2019 (In-person prehab vs Control)	11.5	2.7	5	13.2	5.4	3	2.1%	-1.70 [-8.25, 4.85]
Doiron-Cadrin 2019 (Tele-prehab vs Control)	9.4	1.3	6	13.2	5.4	2	1.6%	-3.80 [-11.36, 3.76]
Dominguez-Navarro 2020 (ST vs Control)	13.4	7.6	24	15.7	6.1	10	3.5%	-2.30 [-7.15, 2.55]
Dominguez-Navarro 2020 (STB vs Control)	11.9	2.6	20	15.7	6.1	11	5.2%	-3.80 [-7.58, -0.02]
Huber 2015	9.1	2.65	21	10.7	0.26	19	15.6%	-1.60 [-2.74, -0.46]
Rooks 2006	12.15	2.65	14	10.38	1.77	15	12.8%	1.77 [0.12, 3.42]
Skoffer 2016	9.1	2.6	29	9.3	3	26	13.7%	-0.20 [-1.69, 1.29]
**Subtotal (95% CI)**			**176**			**126**	**100.0%**	**-1.38 [-2.39, -0.37]**
Heterogeneity: Tau² = 1.36; Chi² = 30.28, df = 9 (P = 0.0004); I² = 70%								
Test for overall effect: Z = 2.69 (P = 0.007)								
**1.58.2 TUG at 6 weeks post-op**								
An 2021 (Pre-op education vs standard care)	10.45	0.67	17	11.65	1.85	9	31.4%	-1.20 [-2.45, 0.05]
An 2021 (Tele-prehab vs standard care)	9.81	1.06	18	11.65	1.85	9	28.9%	-1.84 [-3.14, -0.54]
Dominguez-Navarro 2020 (ST vs Control)	13.1	6.8	20	13.7	3.8	10	3.4%	-0.60 [-4.40, 3.20]
Dominguez-Navarro 2020 (STB vs Control)	12.4	3.7	24	13.7	3.8	11	6.8%	-1.30 [-3.99, 1.39]
Skoffer 2016	8.3	2.3	29	10	2.4	23	29.5%	-1.70 [-2.99, -0.41]
**Subtotal (95% CI)**			**108**			**62**	**100.0%**	**-1.52 [-2.22, -0.82]**
Heterogeneity: Tau² = 0.00; Chi² = 0.81, df = 4 (P = 0.94); I² = 0%								
Test for overall effect: Z = 4.25 (P < 0.0001)								
**1.58.3 TUG at 3 months post-op**								
Cavill 2016	11.8	6.8	20	10.5	5.2	19	6.3%	1.30 [-2.49, 5.09]
Huber 2015	10.1	2.77	21	10.3	0.46	20	38.4%	-0.20 [-1.40, 1.00]
Rooks 2006	12.03	2.05	14	12.4	3.22	15	19.9%	-0.37 [-2.32, 1.58]
Skoffer 2016	8.3	2.3	29	10	2.4	23	35.4%	-1.70 [-2.99, -0.41]
**Subtotal (95% CI)**			**84**			**77**	**100.0%**	**-0.67 [-1.66, 0.32]**
Heterogeneity: Tau² = 0.28; Chi² = 4.15, df = 3 (P = 0.25); I² = 28%								
Test for overall effect: Z = 1.33 (P = 0.18)								
**1.58.4 TUG at 12 months post-op**								
Dominguez-Navarro 2020 (ST vs Control)	11.1	3.1	19	12.1	2.9	8	37.8%	-1.00 [-3.45, 1.45]
Dominguez-Navarro 2020 (STB vs Control)	11.1	2.8	15	12.1	2.9	8	37.4%	-1.00 [-3.46, 1.46]
Skoffer 2016	7.5	2.2	24	7.7	6.6	20	24.8%	-0.20 [-3.22, 2.82]
**Subtotal (95% CI)**			**58**			**36**	**100.0%**	**-0.80 [-2.31, 0.70]**
Heterogeneity: Tau² = 0.00; Chi² = 0.20, df = 2 (P = 0.90); I² = 0%								
Test for overall effect: Z = 1.05 (P = 0.30)								

Mean Difference
IV, Random, 95% CI

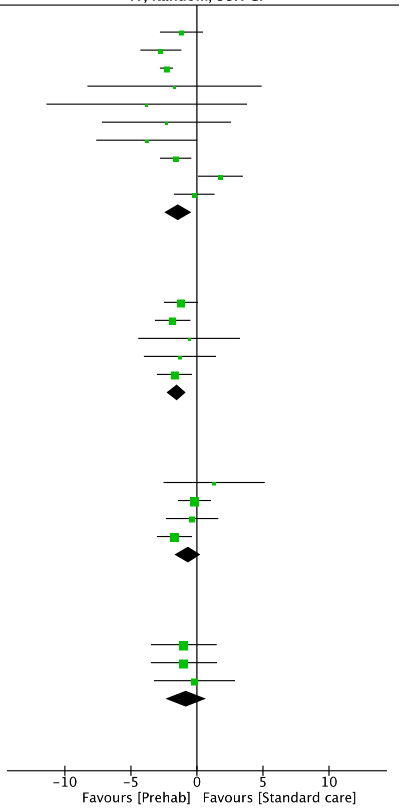

© 2023 Punnoose A et al. *JAMA Network Open*.

eFigure 33. Stair Test: Preoperative and 6 Weeks and 3 Months Postoperative for TKR

Study or Subgroup	Prehabilitation Mean	Prehabilitation SD	Prehabilitation Total	Standard care Mean	Standard care SD	Standard care Total	Weight	Mean Difference IV, Random, 95% CI
**1.52.1 Stair test at Pre-op**								
Calatayud 2017	7.2	1.46	22	11.4	1.46	22	74.6%	-4.20 [-5.06, -3.34]
Doiron-Cadrin 2019 (In-person prehab vs Control)	23.4	10.9	5	28.6	18.5	3	0.4%	-5.20 [-28.21, 17.81]
Doiron-Cadrin 2019 (Tele-prehab vs Control)	15.6	7.6	6	28.6	18.5	2	0.3%	-13.00 [-39.35, 13.35]
McKay 2012	26.86	24.89	10	23.28	11.7	12	0.8%	3.58 [-13.21, 20.37]
Savkin 2021	23.99	10.8	20	28.61	8.3	20	5.7%	-4.62 [-10.59, 1.35]
Van Leeuwen 2014	11.6	3.4	10	12.4	3.3	8	18.2%	-0.80 [-3.91, 2.31]
**Subtotal (95% CI)**			**73**			**67**	**100.0%**	**-3.58 [-5.04, -2.11]**
Heterogeneity: $Tau^2 = 0.56$; $Chi^2 = 5.55$, df = 5 (P = 0.35); $I^2 = 10\%$								
Test for overall effect: Z = 4.77 (P < 0.00001)								
**1.52.2 Stair test at 6 weeks post-op**								
McKay 2012	30.53	24.84	9	26.72	12.05	10	5.9%	3.81 [-14.05, 21.67]
Savkin 2021	24.36	9.41	20	25.41	7.33	20	69.1%	-1.05 [-6.28, 4.18]
Van Leeuwen 2014	20.9	10.8	10	17.6	7.5	7	25.0%	3.30 [-5.40, 12.00]
**Subtotal (95% CI)**			**39**			**37**	**100.0%**	**0.32 [-4.02, 4.67]**
Heterogeneity: $Tau^2 = 0.00$; $Chi^2 = 0.86$, df = 2 (P = 0.65); $I^2 = 0\%$								
Test for overall effect: Z = 0.15 (P = 0.88)								
**1.52.3 Stair test at 3 months post-op**								
Calatayud 2017	7.9	1.46	22	12.1	1.91	22	54.3%	-4.20 [-5.20, -3.20]
McKay 2012	26.99	26.73	7	22.18	10.98	10	0.8%	4.81 [-16.13, 25.75]
Savkin 2021	18.88	6.59	20	22.43	7.35	20	14.8%	-3.55 [-7.88, 0.78]
Van Leeuwen 2014	12.8	3.4	7	14.1	0.1	9	30.1%	-1.30 [-3.82, 1.22]
**Subtotal (95% CI)**			**56**			**61**	**100.0%**	**-3.16 [-5.05, -1.26]**
Heterogeneity: $Tau^2 = 1.46$; $Chi^2 = 5.05$, df = 3 (P = 0.17); $I^2 = 41\%$								
Test for overall effect: Z = 3.27 (P = 0.001)								

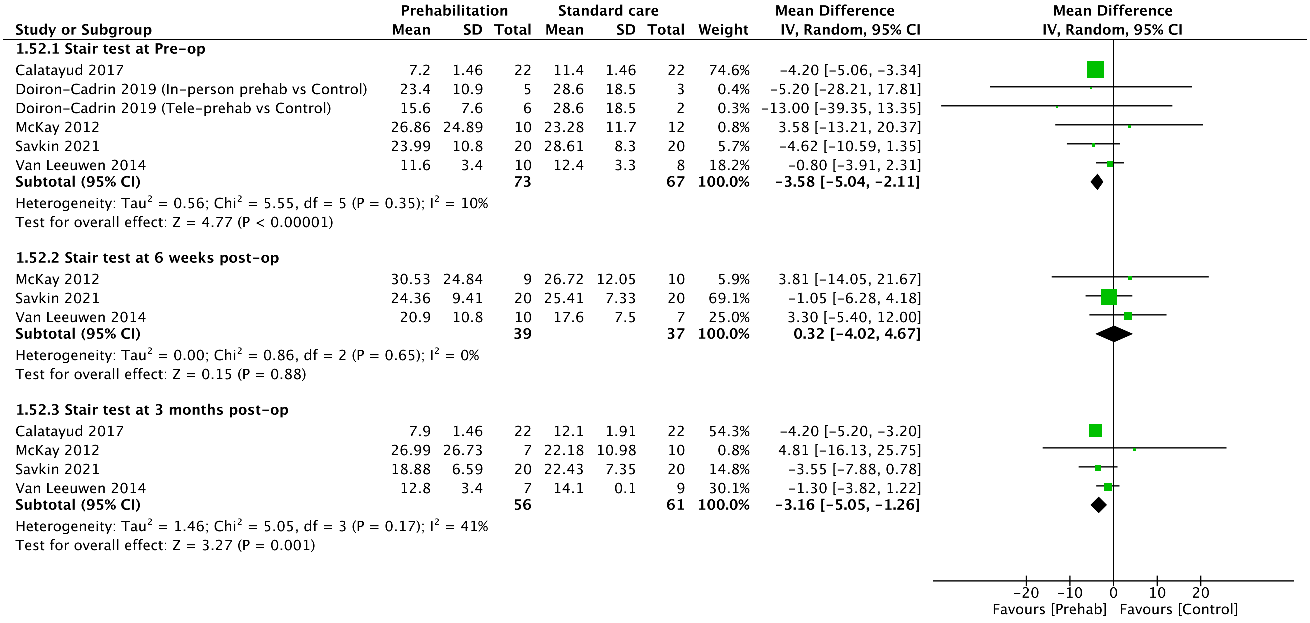

© 2023 Punnoose A et al. *JAMA Network Open*.

eFigure 34. Active Knee Extension ROM: Preoperative and 6 Weeks, 3 Months, and 12 Months Postoperative for TKR

Study or Subgroup	Prehabilitation Mean	SD	Total	Standard care Mean	SD	Total	Weight	Mean Difference IV, Random, 95% CI
**1.39.1 Knee extension at Pre-op**								
Calatayud 2017	6.6	2.36	22	14.9	5.84	22	10.9%	-8.30 [-10.93, -5.67]
Dominguez-Navarro 2020 (ST vs Control)	3	3.5	24	4.3	5.1	11	9.4%	-1.30 [-4.62, 2.02]
Dominguez-Navarro 2020 (STB vs Control)	4.7	4.2	20	4.3	5.1	10	8.7%	0.40 [-3.26, 4.06]
Evgeniadis 2008	2.46	2.46	18	3.87	3.93	20	12.1%	-1.41 [-3.47, 0.65]
Franz 2022 (Prehab+BFR vs Standard care)	2.7	2.83	10	2.6	1.9	5	11.4%	0.10 [-2.32, 2.52]
Franz 2022 (Prehab+Sham BFR vs Standard care	2.3	2.83	10	2.6	1.9	5	11.4%	-0.30 [-2.72, 2.12]
Huber 2015	7.2	4.54	21	6.4	4.56	19	10.5%	0.80 [-2.02, 3.62]
NCT01844934	3.22	5.24	9	3.5	7.04	10	5.7%	-0.28 [-5.83, 5.27]
Savkin 2021	13.45	5.7	20	14.8	8.32	20	7.4%	-1.35 [-5.77, 3.07]
Skoffer 2016	5.76	3.8	29	6.66	3.2	26	12.6%	-0.90 [-2.75, 0.95]
**Subtotal (95% CI)**			**183**			**148**	**100.0%**	**-1.33 [-3.02, 0.36]**
Heterogeneity: Tau² = 5.05; Chi² = 32.37, df = 9 (P = 0.0002); I² = 72%								
Test for overall effect: Z = 1.54 (P = 0.12)								
**1.39.2 Knee extension at 6 weeks post-op**								
Dominguez-Navarro 2020 (ST vs Control)	4.2	4.1	24	3.6	2.9	11	24.0%	0.60 [-1.77, 2.97]
Dominguez-Navarro 2020 (STB vs Control)	3	4.7	20	3.6	2.9	10	18.1%	-0.60 [-3.33, 2.13]
Savkin 2021	12.5	6.15	20	12.2	5.62	20	10.1%	0.30 [-3.35, 3.95]
Skoffer 2016	5.26	2.9	29	5.26	3.2	23	47.8%	0.00 [-1.68, 1.68]
**Subtotal (95% CI)**			**93**			**64**	**100.0%**	**0.07 [-1.10, 1.23]**
Heterogeneity: Tau² = 0.00; Chi² = 0.44, df = 3 (P = 0.93); I² = 0%								
Test for overall effect: Z = 0.11 (P = 0.91)								
**1.39.3 Knee extension at 3 months post-op**								
Calatayud 2017	8.2	2.36	22	13.9	2.36	22	14.4%	-5.70 [-7.09, -4.31]
Cavill 2016	12.9	8.5	20	13.3	6.4	19	7.8%	-0.40 [-5.11, 4.31]
Evgeniadis 2008	5.7	4.27	18	6.42	3.6	20	12.2%	-0.72 [-3.25, 1.81]
Franz 2022 (Prehab+BFR vs Standard care)	1.5	3.24	10	3.3	2.98	5	10.5%	-1.80 [-5.09, 1.49]
Franz 2022 (Prehab+Sham BFR vs Standard care	2	1.63	10	3.3	2.98	5	11.6%	-1.30 [-4.10, 1.50]
Huber 2015	3.9	4.66	21	2.7	4.7	20	11.4%	1.20 [-1.67, 4.07]
NCT01844934	3.67	4.36	9	3.8	6.16	10	7.7%	-0.13 [-4.89, 4.63]
Savkin 2021	9.05	5.27	20	9.25	5.88	20	10.1%	-0.20 [-3.66, 3.26]
Skoffer 2016	3.36	2.8	29	4.36	2.4	21	14.4%	-1.00 [-2.45, 0.45]
**Subtotal (95% CI)**			**159**			**142**	**100.0%**	**-1.32 [-3.16, 0.53]**
Heterogeneity: Tau² = 5.63; Chi² = 36.57, df = 8 (P < 0.0001); I² = 78%								
Test for overall effect: Z = 1.40 (P = 0.16)								
**1.39.4 Knee extension at 12 months post-op**								
Dominguez-Navarro 2020 (ST vs Control)	2.2	2.3	19	0.1	3.3	8	28.4%	2.10 [-0.41, 4.61]
Dominguez-Navarro 2020 (STB vs Control)	1.6	2.2	15	0.1	3.3	8	27.9%	1.50 [-1.04, 4.04]
Skoffer 2016	1.8	2.3	24	2.4	2.8	20	43.7%	-0.60 [-2.13, 0.93]
**Subtotal (95% CI)**			**58**			**36**	**100.0%**	**0.75 [-1.03, 2.54]**
Heterogeneity: Tau² = 1.29; Chi² = 4.11, df = 2 (P = 0.13); I² = 51%								
Test for overall effect: Z = 0.83 (P = 0.41)								

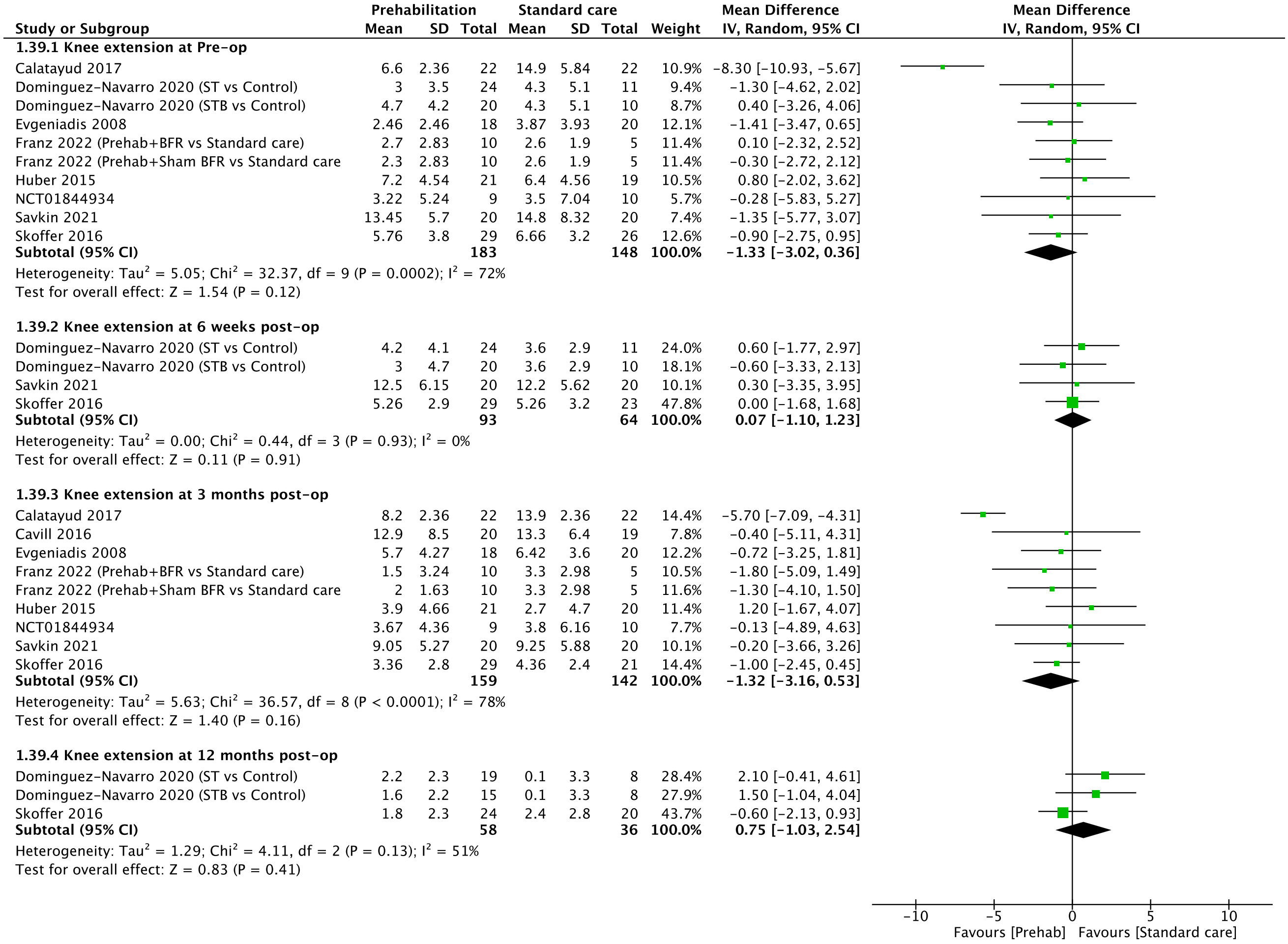

© 2023 Punnoose A et al. *JAMA Network Open*.

eFigure 35. 6MWT: Preoperative for THR

	Prehab			Standard care				Mean Difference
**Study or Subgroup**	**Mean**	**SD**	**Total**	**Mean**	**SD**	**Total**	**Weight**	**IV, Random, 95% CI**
Hoogeboom 2010	363	126.3	10	342.7	133.7	10	11.5%	20.30 [−93.69, 134.29]
Oosting 2012	288	88	14	296	113	12	20.8%	−8.00 [−86.82, 70.82]
Zeng 2014	478.1	52.46	40	419.02	47.56	49	67.7%	59.08 [38.07, 80.09]
**Total (95% CI)**			**64**			**71**	**100.0%**	**40.65 [−1.01, 82.32]**

Heterogeneity: Tau^2 = 552.76; Chi^2 = 2.94, df = 2 (P = 0.23); I^2 = 32%
Test for overall effect: Z = 1.91 (P = 0.06)

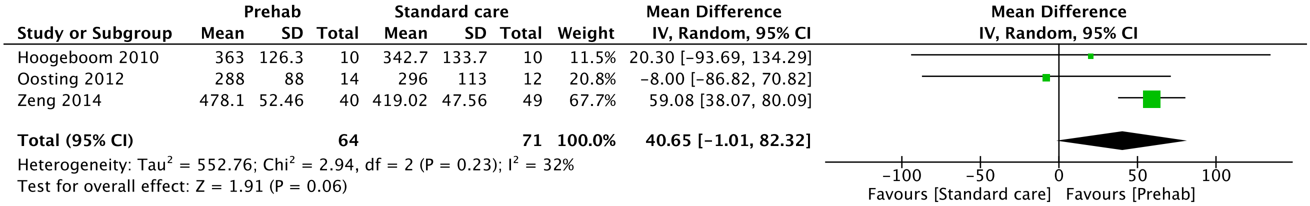

© 2023 Punnoose A et al. *JAMA Network Open*.

eFigure 36. 30-Second Chair Rise Test: Preoperative and 6 Weeks and 3 Months Postoperative for TKR

Study or Subgroup	Prehabilitation Mean	Prehabilitation SD	Prehabilitation Total	Standard care Mean	Standard care SD	Standard care Total	Weight	Mean Difference IV, Random, 95% CI
**1.66.1 30 sec CRT at Pre-op**								
Franz 2022 (Prehab+BFR vs Standard care)	11.2	2.35	10	10.5	4.5	5	6.1%	0.70 [−3.50, 4.90]
Franz 2022 (Prehab+Sham BFR vs Standard care	11	3	10	10.5	4.5	5	5.7%	0.50 [−3.86, 4.86]
NCT01844934	14.89	6.07	9	16.8	8.23	10	2.7%	−1.91 [−8.37, 4.55]
Savkin 2021	9.75	1.86	20	9.8	2.14	20	46.3%	−0.05 [−1.29, 1.19]
Skoffer 2016	13.3	5.1	29	11.1	2.9	26	20.3%	2.20 [0.03, 4.37]
Topp 2009	12.08	4.23	26	9.82	4.23	28	18.9%	2.26 [0.00, 4.52]
**Subtotal (95% CI)**			**104**			**94**	**100.0%**	**0.87 [−0.19, 1.94]**
Heterogeneity: $Tau^2 = 0.24$; $Chi^2 = 5.70$, df = 5 (P = 0.34); $I^2 = 12\%$								
Test for overall effect: Z = 1.60 (P = 0.11)								
**1.66.2 30 sec CRT at 6 weeks post-op**								
NCT01844934	11.99	5.51	9	13.2	6.56	10	4.5%	−1.21 [−6.64, 4.22]
Savkin 2021	10	2.22	20	9.95	2.14	20	31.2%	0.05 [−1.30, 1.40]
Skoffer 2016	13.3	5	29	9.6	4.4	23	15.6%	3.70 [1.14, 6.26]
Topp 2009	11.46	0.69	26	10.36	0.67	28	48.7%	1.10 [0.74, 1.46]
**Subtotal (95% CI)**			**84**			**81**	**100.0%**	**1.07 [−0.14, 2.29]**
Heterogeneity: $Tau^2 = 0.75$; $Chi^2 = 6.95$, df = 3 (P = 0.07); $I^2 = 57\%$								
Test for overall effect: Z = 1.73 (P = 0.08)								
**1.66.3 30 sec CRT at 3 months post-op**								
Franz 2022 (Prehab+BFR vs Standard care)	10.9	2.77	10	10.25	3.75	5	11.5%	0.65 [−3.06, 4.36]
Franz 2022 (Prehab+Sham BFR vs Standard care	10	2.98	10	10.25	3.75	5	11.2%	−0.25 [−4.02, 3.52]
NCT01844934	16	6.38	9	17.5	8.63	10	4.2%	−1.50 [−8.28, 5.28]
Savkin 2021	11.4	2.14	20	11.6	2.3	20	32.3%	−0.20 [−1.58, 1.18]
Skoffer 2016	14.7	4.7	29	11	4.4	21	19.0%	3.70 [1.16, 6.24]
Topp 2009	12.87	4.18	26	11.25	4.18	28	21.9%	1.62 [−0.61, 3.85]
**Subtotal (95% CI)**			**104**			**89**	**100.0%**	**0.98 [−0.47, 2.43]**
Heterogeneity: $Tau^2 = 1.20$; $Chi^2 = 8.28$, df = 5 (P = 0.14); $I^2 = 40\%$								
Test for overall effect: Z = 1.32 (P = 0.19)								

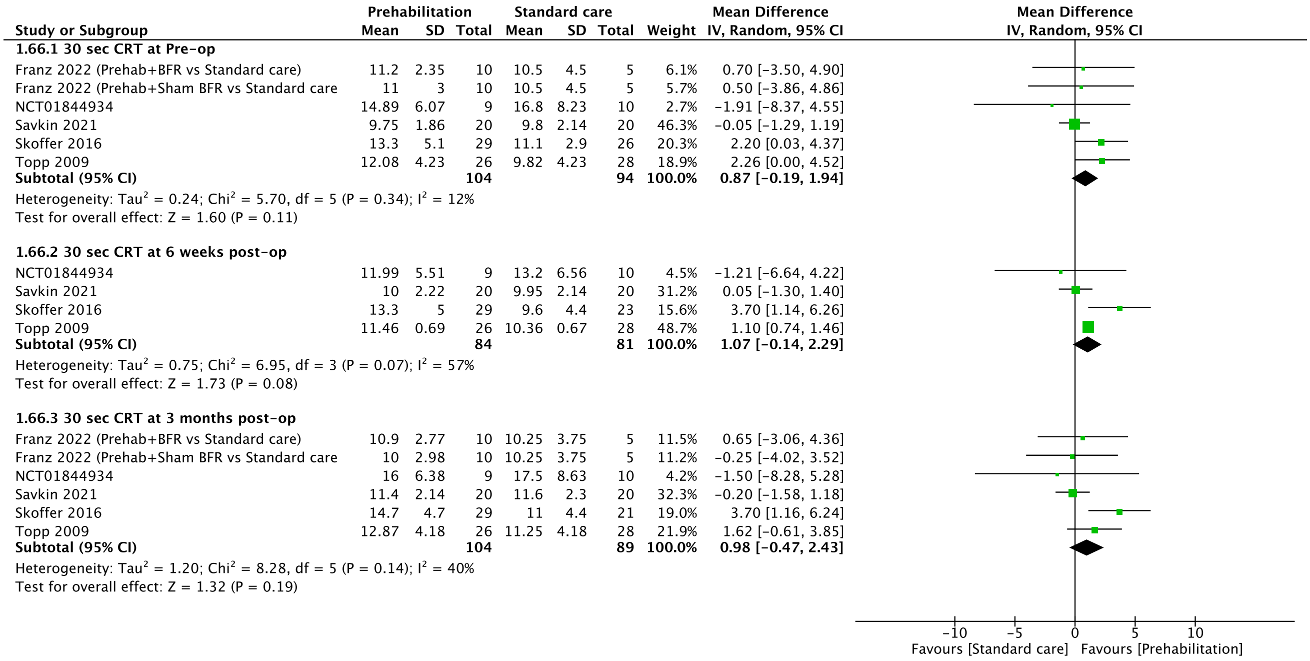

© 2023 Punnoose A et al. *JAMA Network Open*.

eFigure 37. Functional Reach: Preoperative and 6 Weeks Postoperative for TKR

	Prehabilitation			Standard care				Mean Difference
Study or Subgroup	Mean	SD	Total	Mean	SD	Total	Weight	IV, Random, 95% CI
**1.69.1 Functional reach at Pre-op**								
Dominguez-Navarro 2020 (ST vs Control)	30.8	5.4	24	28	5.8	10	36.6%	2.80 [-1.39, 6.99]
Dominguez-Navarro 2020 (STB vs Control)	30.9	5.1	20	28	5.8	11	38.5%	2.90 [-1.19, 6.99]
Rooks 2006	26.7	7.5	14	28	6.4	15	24.9%	-1.30 [-6.39, 3.79]
**Subtotal (95% CI)**			**58**			**36**	**100.0%**	**1.82 [-0.72, 4.36]**
Heterogeneity: Tau^2 = 0.00; Chi^2 = 1.92, df = 2 (P = 0.38); I^2 = 0%								
Test for overall effect: Z = 1.40 (P = 0.16)								
**1.69.2 Functional reach at 6 weeks post-op**								
Dominguez-Navarro 2020 (ST vs Control)	31.6	4.8	24	29.9	5.2	10	39.2%	1.70 [-2.05, 5.45]
Dominguez-Navarro 2020 (STB vs Control)	32.3	5.8	20	29.9	5.2	11	35.2%	2.40 [-1.59, 6.39]
Rooks 2006	30.7	6.9	14	32.8	6.1	15	25.6%	-2.10 [-6.85, 2.65]
**Subtotal (95% CI)**			**58**			**36**	**100.0%**	**0.97 [-1.54, 3.49]**
Heterogeneity: Tau^2 = 0.54; Chi^2 = 2.24, df = 2 (P = 0.33); I^2 = 11%								
Test for overall effect: Z = 0.76 (P = 0.45)								

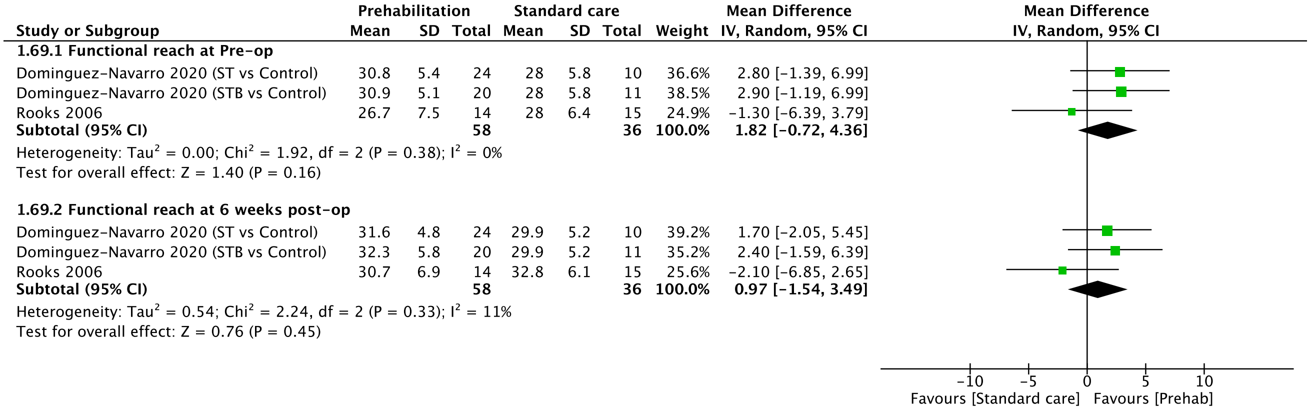

© 2023 Punnoose A et al. *JAMA Network Open*.

eFigure 38. Timed Up and Go Test (TUG): Preoperative and 6 Weeks Posterative for THR

Study or Subgroup	Prehab Mean	Prehab SD	Prehab Total	Standard care Mean	Standard care SD	Standard care Total	Weight	Mean Difference IV, Random, 95% CI
**1.21.1 TUG at pre-op**								
Doiron-Cadrin 2019 (Inperson prehab vs control)	9.8	3.1	6	10	2.3	3	17.1%	-0.20 [-3.80, 3.40]
Doiron-Cadrin 2019 (Teleprehab vs control)	7.7	1.1	6	10	2.3	2	18.2%	-2.30 [-5.61, 1.01]
Hoogeboom 2010	9.6	2.8	10	13.4	11.5	10	8.0%	-3.80 [-11.14, 3.54]
Oosting 2012	14.9	7.6	12	17.2	13.8	14	6.6%	-2.30 [-10.71, 6.11]
Rooks 2006	11.35	2.35	25	11.3	2.25	24	25.0%	0.05 [-1.24, 1.34]
Zeng 2014	14.61	2.6	40	19.06	3.37	49	25.1%	-4.45 [-5.69, -3.21]
**Subtotal (95% CI)**			**99**			**102**	**100.0%**	**-2.01 [-4.49, 0.47]**
Heterogeneity: $Tau^2 = 5.97$; $Chi^2 = 25.73$, df = 5 (P = 0.0001); $I^2 = 81\%$								
Test for overall effect: Z = 1.59 (P = 0.11)								
**1.21.2 TUG at 6 weeks post-op**								
Oosting 2012	13.4	5.5	14	13.8	11.1	12	4.4%	-0.40 [-7.31, 6.51]
Rooks 2006	11.53	2.42	25	10.9	2.83	24	95.6%	0.63 [-0.85, 2.11]
**Subtotal (95% CI)**			**39**			**36**	**100.0%**	**0.58 [-0.86, 2.03]**
Heterogeneity: $Tau^2 = 0.00$; $Chi^2 = 0.08$, df = 1 (P = 0.78); $I^2 = 0\%$								
Test for overall effect: Z = 0.79 (P = 0.43)								

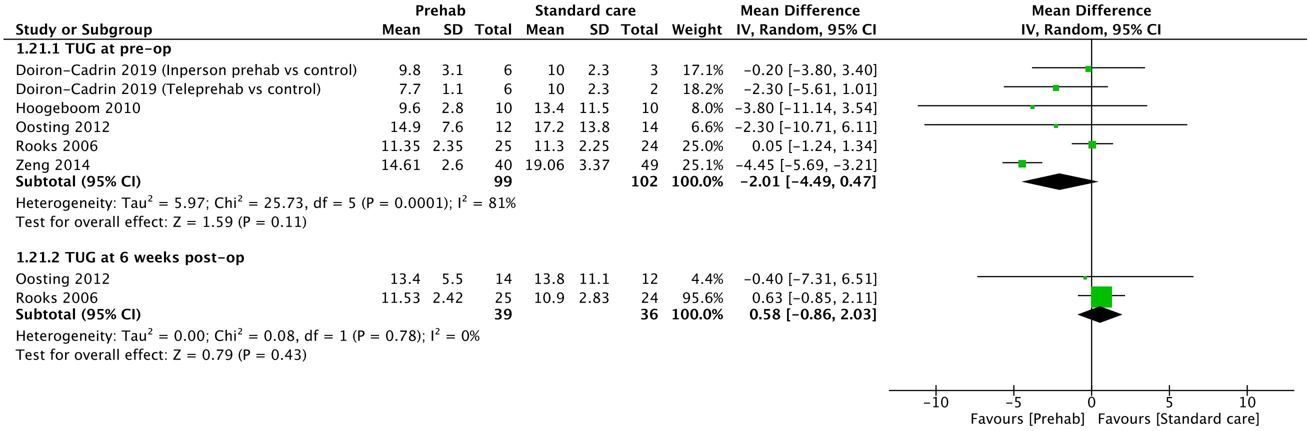

© 2023 Punnoose A et al. *JAMA Network Open*.

eFigure 39. Total Length of Stay for THR and TKR

Study or Subgroup	Prehabilitation Mean	Prehabilitation SD	Prehabilitation Total	Standard care Mean	Standard care SD	Standard care Total	Weight	Mean Difference IV, Random, 95% CI
**1.2.1 THR**								
Cavill 2016	6.2	1.8	9	7.2	3.8	11	2.5%	−1.00 [−3.53, 1.53]
Hoogeboom 2010	9.75	4.93	10	5.75	0.82	10	1.8%	4.00 [0.90, 7.10]
Oosting 2012	5.1	1	12	5.4	2.1	14	7.4%	−0.30 [−1.54, 0.94]
**Subtotal (95% CI)**			**31**			**35**	**11.7%**	**0.63 [−1.82, 3.09]**
Heterogeneity: $Tau^2 = 3.33$; $Chi^2 = 7.21$, df = 2 (P = 0.03); $I^2 = 72\%$								
Test for overall effect: Z = 0.51 (P = 0.61)								
**1.2.2 TKR**								
Beaupre 2004	6.7	2.2	55	7.3	2.5	60	10.6%	−0.60 [−1.46, 0.26]
Cavill 2016	7.3	2.6	20	6.7	1.9	19	6.2%	0.60 [−0.82, 2.02]
Huang 2012	7	2	126	8	1	117	15.8%	−1.00 [−1.39, −0.61]
Mitchell 2005	9.2	3.4	57	9.6	3	57	7.8%	−0.40 [−1.58, 0.78]
NCT03044028	1.83	0.72	12	1.68	0.78	22	14.4%	0.15 [−0.37, 0.67]
Williamson 2007 – (Acupuncture vs Control)	7.73	3.96	57	6.6	2.62	30	6.4%	1.13 [−0.26, 2.52]
Williamson 2007– (Exercises vs Control)	6.49	1.99	50	6.6	2.62	31	8.6%	−0.11 [−1.18, 0.96]
**Subtotal (95% CI)**			**377**			**336**	**69.7%**	**−0.16 [−0.74, 0.41]**
Heterogeneity: $Tau^2 = 0.37$; $Chi^2 = 20.14$, df = 6 (P = 0.003); $I^2 = 70\%$								
Test for overall effect: Z = 0.56 (P = 0.58)								
**1.2.3 THR+TKR**								
Bergin 2014	2.5	0.6	50	2.7	0.6	56	17.2%	−0.20 [−0.43, 0.03]
Crowe 2003	6.55	4.2	65	10.5	14.2	68	1.4%	−3.95 [−7.48, −0.42]
**Subtotal (95% CI)**			**115**			**124**	**18.7%**	**−1.65 [−5.22, 1.93]**
Heterogeneity: $Tau^2 = 5.41$; $Chi^2 = 4.33$, df = 1 (P = 0.04); $I^2 = 77\%$								
Test for overall effect: Z = 0.90 (P = 0.37)								
**Total (95% CI)**			**523**			**495**	**100.0%**	**−0.20 [−0.63, 0.24]**

Heterogeneity: $Tau^2 = 0.27$; $Chi^2 = 33.77$, df = 11 (P = 0.0004); $I^2 = 67\%$
Test for overall effect: Z = 0.89 (P = 0.37)
Test for subgroup differences: $Chi^2 = 1.07$, df = 2 (P = 0.59), $I^2 = 0\%$

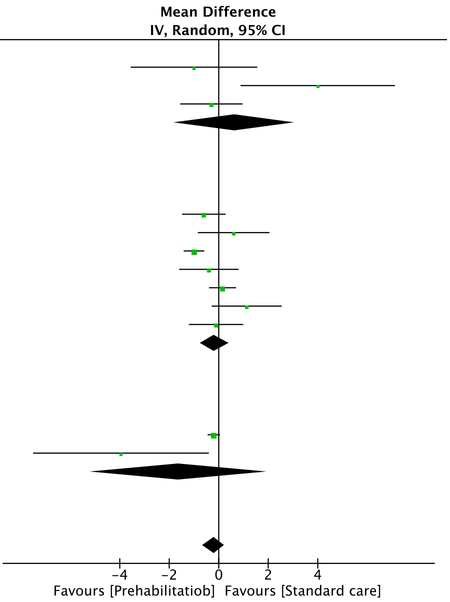

© 2023 Punnoose A et al. *JAMA Network Open*.

eFigure 40. Length of Stay for Spinal Surgical Procedures

	Prehabilitation			Control				Mean Difference
Study or Subgroup	Mean	SD	Total	Mean	SD	Total	Weight	IV, Random, 95% CI
Marchand 2019	5.8	1.2	28	8.5	2.4	32	61.0%	−2.70 [−3.64, −1.76]
Nielsen 2010	3.6	4	20	4.2	2.6	17	39.0%	−0.60 [−2.74, 1.54]
**Total (95% CI)**			**48**			**49**	**100.0%**	**−1.88 [−3.89, 0.13]**

Heterogeneity: $Tau^2 = 1.49$; $Chi^2 = 3.09$, df = 1 (P = 0.08); $I^2 = 68\%$
Test for overall effect: Z = 1.84 (P = 0.07)

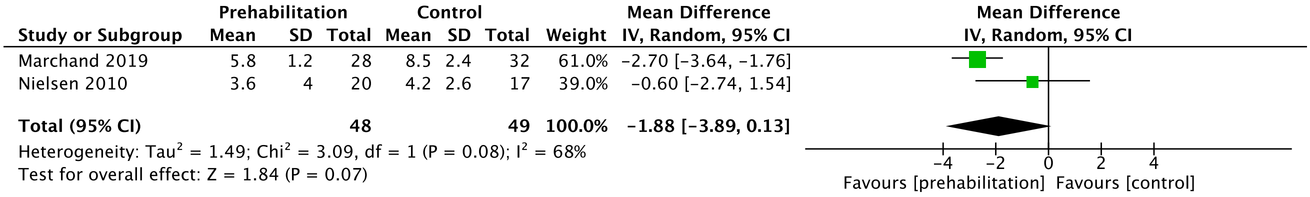

© 2023 Punnoose A et al. *JAMA Network Open*.

eFigure 41. Health Care Costs for TKR

	Prehabilitation			Standard care				Std. Mean Difference	Std. Mean Difference
**Study or Subgroup**	**Mean**	**SD**	**Total**	**Mean**	**SD**	**Total**	**Weight**	**IV, Random, 95% CI**	**IV, Random, 95% CI**
Beaupre 2004	982.17	274	51	980.02	1,415	58	23.3%	0.00 [−0.37, 0.38]	
Huang 2012	3,156.62	5,204	126	3,210.5	4,428	117	52.2%	−0.01 [−0.26, 0.24]	
Mitchell 2005	5,376	916	57	5,372	832	57	24.5%	0.00 [−0.36, 0.37]	
**Total (95% CI)**			**234**			**232**	**100.0%**	**−0.00 [−0.19, 0.18]**	

Heterogeneity: $Tau^2 = 0.00$; $Chi^2 = 0.01$, df = 2 (P = 1.00); $I^2 = 0\%$
Test for overall effect: Z = 0.05 (P = 0.96)

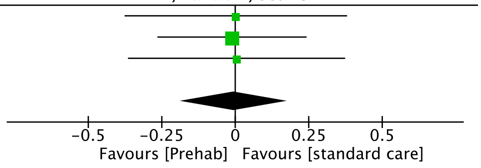

© 2023 Punnoose A et al. *JAMA Network Open*.

eFigure 42. Readmission Rates for TKR

	Prehabilitation		Standard care			Risk Ratio
**Study or Subgroup**	**Events**	**Total**	**Events**	**Total**	**Weight**	**M-H, Random, 95% CI**
Beaupre 2004	1	51	2	58	10.6%	0.57 [0.05, 6.09]
NCT03044028	0	12	1	22	6.1%	0.59 [0.03, 13.46]
Riddle 2019 (Education vs Control)	7	135	4	68	41.7%	0.88 [0.27, 2.91]
Riddle 2019-(CBT vs Control)	7	130	4	68	41.7%	0.92 [0.28, 3.02]
**Total (95% CI)**		**328**		**216**	**100.0%**	**0.83 [0.39, 1.80]**
Total events	15		11			

Heterogeneity: $Tau^2 = 0.00$; $Chi^2 = 0.18$, df = 3 (P = 0.98); $I^2 = 0\%$
Test for overall effect: Z = 0.46 (P = 0.65)

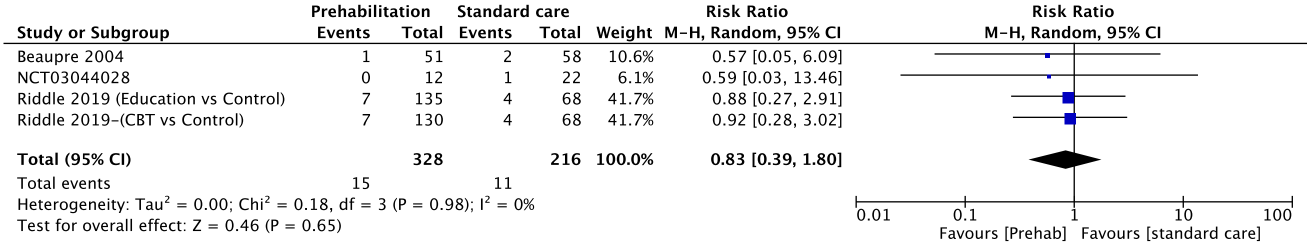

© 2023 Punnoose A et al. *JAMA Network Open*.

eFigure 43. Total Complication Rates for THR and TKR

Study or Subgroup	Prehabilitation Events	Prehabilitation Total	Standard care Events	Standard care Total	Weight	Risk Ratio M-H, Random, 95% CI
**1.1.1 THR+TKR**						
Bergin 2014	27	50	37	56	44.8%	0.82 [0.60, 1.12]
Crowe 2003	7	65	16	68	6.7%	0.46 [0.20, 1.04]
Villadsen 2013	1	84	2	81	0.8%	0.48 [0.04, 5.21]
**Subtotal (95% CI)**		**199**		**205**	**52.2%**	**0.74 [0.54, 1.02]**
Total events	35		55			
Heterogeneity: $Tau^2 = 0.01$; $Chi^2 = 2.07$, df = 2 (P = 0.36); $I^2 = 3\%$						
Test for overall effect: Z = 1.82 (P = 0.07)						
**1.1.2 THR**						
Gocen 2004	1	29	1	30	0.6%	1.03 [0.07, 15.77]
Hoogeboom 2010	2	10	0	10	0.5%	5.00 [0.27, 92.62]
Oosting 2012	8	15	11	13	16.2%	0.63 [0.37, 1.07]
Rooks 2006	0	25	4	24	0.5%	0.11 [0.01, 1.88]
**Subtotal (95% CI)**		**79**		**77**	**17.9%**	**0.67 [0.28, 1.61]**
Total events	11		16			
Heterogeneity: $Tau^2 = 0.19$; $Chi^2 = 3.52$, df = 3 (P = 0.32); $I^2 = 15\%$						
Test for overall effect: Z = 0.89 (P = 0.37)						
**1.1.3 TKR**						
Beaupre 2004	9	51	13	58	7.8%	0.79 [0.37, 1.69]
Brown 2014	5	16	6	15	4.9%	0.78 [0.30, 2.03]
Calatayud 2017	0	22	3	22	0.5%	0.14 [0.01, 2.61]
Gstoettner 2011	0	18	1	20	0.5%	0.37 [0.02, 8.51]
Huang 2012	7	126	4	117	3.1%	1.63 [0.49, 5.41]
Huber 2015	1	17	0	19	0.5%	3.33 [0.14, 76.75]
Mat Eil-Ismail 2016	2	24	4	26	1.8%	0.54 [0.11, 2.69]
McKay 2012	1	7	0	10	0.5%	4.13 [0.19, 88.71]
NCT03044028	0	12	1	22	0.5%	0.59 [0.03, 13.46]
Riddle 2019 (Education vs Control)	15	135	5	68	4.8%	1.51 [0.57, 3.98]
Riddle 2019-(CBT vs Control)	19	130	5	68	5.1%	1.99 [0.78, 5.09]
**Subtotal (95% CI)**		**558**		**445**	**29.8%**	**1.08 [0.73, 1.60]**
Total events	59		42			
Heterogeneity: $Tau^2 = 0.00$; $Chi^2 = 8.03$, df = 10 (P = 0.63); $I^2 = 0\%$						
Test for overall effect: Z = 0.40 (P = 0.69)						
**Total (95% CI)**		**836**		**727**	**100.0%**	**0.82 [0.66, 1.01]**
Total events	105		113			
Heterogeneity: $Tau^2 = 0.00$; $Chi^2 = 16.64$, df = 17 (P = 0.48); $I^2 = 0\%$						
Test for overall effect: Z = 1.88 (P = 0.06)						
Test for subgroup differences: $Chi^2 = 2.43$, df = 2 (P = 0.30), $I^2 = 17.6\%$						

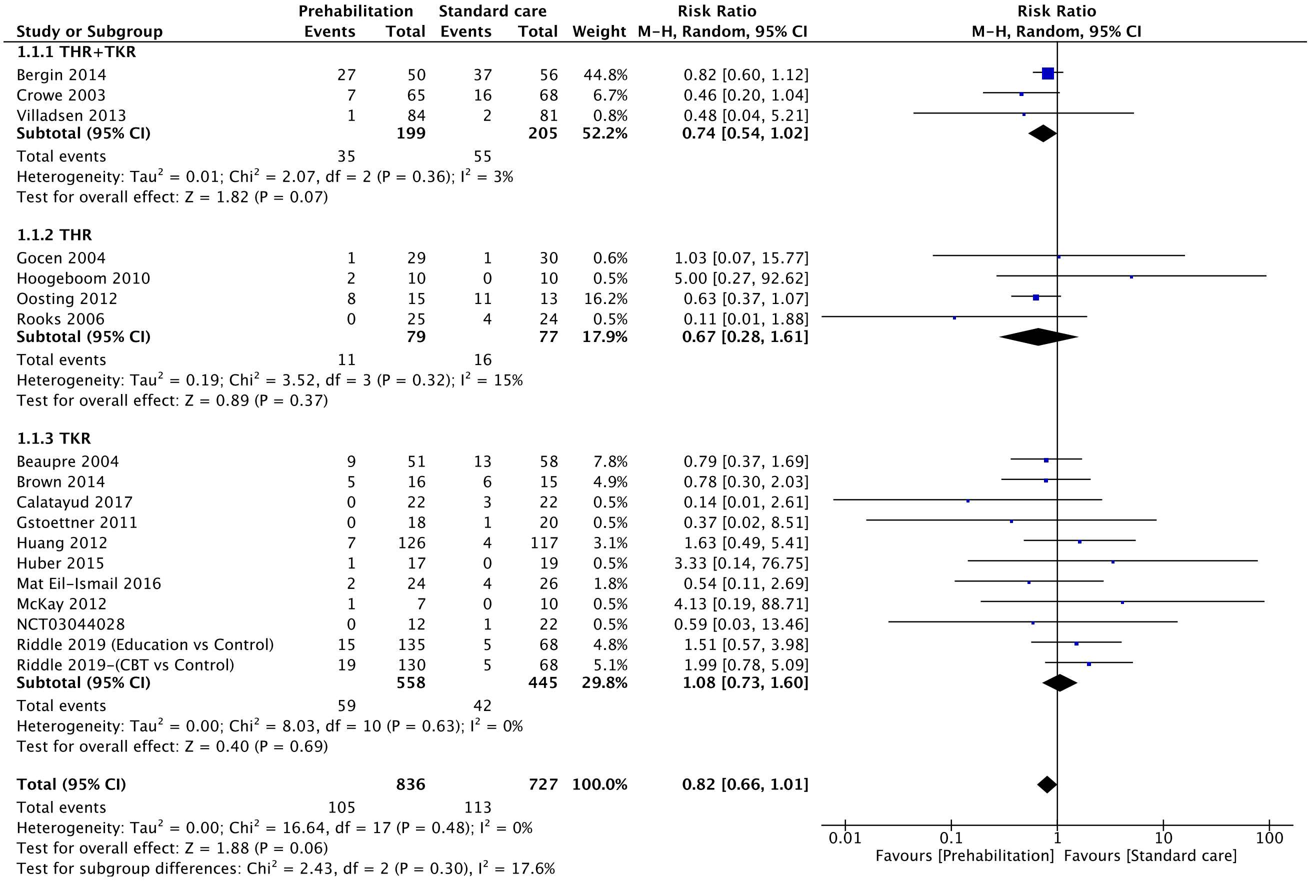

© 2023 Punnoose A et al. *JAMA Network Open*.

eFigure 44. Complication Rates for Spinal Surgery

Study or Subgroup	Experimental Events	Experimental Total	Control Events	Control Total	Weight	Risk Ratio M-H, Random, 95% CI
Nielsen 2010	3	20	1	17	65.8%	2.55 [0.29, 22.31]
Rolving 2015	2	59	0	31	34.2%	2.67 [0.13, 53.88]
**Total (95% CI)**		**79**		**48**	**100.0%**	**2.59 [0.45, 15.03]**
Total events	5		1			

Heterogeneity: $Tau^2 = 0.00$; $Chi^2 = 0.00$, df = 1 (P = 0.98); $I^2 = 0\%$
Test for overall effect: Z = 1.06 (P = 0.29)

Risk Ratio
M-H, Random, 95% CI

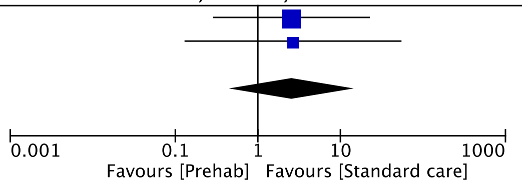

© 2023 Punnoose A et al. *JAMA Network Open*.

eReferences

1. An J, Ryu HK, Lyu SJ, Yi HJ, Lee BH. Effects of Preoperative Telerehabilitation on Muscle Strength, Range of Motion, and Functional Outcomes in Candidates for Total Knee Arthroplasty: A Single-Blind Randomized Controlled Trial. *Int J Environ Res Public Health.* 2021;18(11).
2. Beaupre LA, Lier D, Davies DM, Johnston DB. The effect of a preoperative exercise and education program on functional recovery, health related quality of life, and health service utilization following primary total knee arthroplasty. *J Rheumatol.* 2004;31(6):1166-1173.
3. Berge DJ, Dolin SJ, Williams AC, Harman R. Pre-operative and post-operative effect of a pain management programme prior to total hip replacement: a randomized controlled trial. *Pain.* 2004;110(1-2):33-39.
4. Bergin C, Speroni KG, Travis T, et al. Effect of preoperative incentive spirometry patient education on patient outcomes in the knee and hip joint replacement population. *J Perianesth Nurs.* 2014;29(1):20-27.
5. Brown K, Topp R, Brosky JA, Lajoie AS. Prehabilitation and quality of life three months after total knee arthroplasty: a pilot study. *Percept Mot Skills.* 2012;115(3):765-774.
6. Brown K, Loprinzi PD, Brosky JA, Topp R. Prehabilitation influences exercise-related psychological constructs such as self-efficacy and outcome expectations to exercise. *J Strength Cond Res.* 2014;28(1):201-209.
7. Calatayud J, Casana J, Ezzatvar Y, Jakobsen MD, Sundstrup E, Andersen LL. High-intensity preoperative training improves physical and functional recovery in the early post-operative periods after total knee arthroplasty: a randomized controlled trial. *Knee Surg Sports Traumatol Arthrosc.* 2017;25(9):2864-2872.
8. Cavill S, McKenzie K, Munro A, et al. The effect of prehabilitation on the range of motion and functional outcomes in patients following the total knee or hip arthroplasty: A pilot randomized trial. *Physiother Theory Pract.* 2016;32(4):262-270.
9. Crowe J, Henderson J. Pre-arthroplasty rehabilitation is effective in reducing hospital stay. *Can J Occup Ther.* 2003;70(2):88-96.
10. Doiron-Cadrin P, Kairy D, Vendittoli PA, Lowry V, Poitras S, Desmeules F. Feasibility and preliminary effects of a tele-prehabilitation program and an in-person prehablitation program compared to usual care for total hip or knee arthroplasty candidates: a pilot randomized controlled trial. *Disabil Rehabil.* 2020;42(7):989-998.
11. Dominguez-Navarro F, Silvestre-Munoz A, Igual-Camacho C, et al. A randomized controlled trial assessing the effects of preoperative strengthening plus balance training on balance and functional outcome up to 1 year following total knee replacement. *Knee Surg Sports Traumatol Arthrosc.* 2021;29(3):838-848.
12. Evgeniadis G, Beneka A, Malliou P, Mavromoustakos S, Godolias G. Effects of pre- or postoperative therapeutic exercise on the quality of life, before and after total knee arthroplasty for osteoarthritis. *Journal of Back and Musculoskeletal Rehabilitation.* 2008;21:161-169.
13. Ferrara PE, Rabini A, Maggi L, et al. Effect of pre-operative physiotherapy in patients with end-stage osteoarthritis undergoing hip arthroplasty. *Clin Rehabil.* 2008;22(10-11):977-986.
14. Franz A, Ji S, Bittersohl B, Zilkens C, Behringer M. Impact of a Six-Week Prehabilitation With Blood-Flow Restriction Training on Pre- and Postoperative Skeletal Muscle Mass

© 2023 Punnoose A et al. *JAMA Network Open*.

and Strength in Patients Receiving Primary Total Knee Arthroplasty. *Front Physiol.* 2022;13:881484.
15. Gocen Z, Sen A, Unver B, Karatosun V, Gunal I. The effect of preoperative physiotherapy and education on the outcome of total hip replacement: a prospective randomized controlled trial. *Clin Rehabil.* 2004;18(4):353-358.
16. Grant LF, Cooper DJ, Conroy JL. The HAPI 'Hip Arthroscopy Pre-habilitation Intervention' study: does pre-habilitation affect outcomes in patients undergoing hip arthroscopy for femoro-acetabular impingement? *J Hip Preserv Surg.* 2017;4(1):85-92.
17. Gstoettner M, Raschner C, Dirnberger E, Leimser H, Krismer M. Preoperative proprioceptive training in patients with total knee arthroplasty. *Knee.* 2011;18(4):265-270.
18. Hermann A, Holsgaard-Larsen A, Zerahn B, Mejdahl S, Overgaard S. Preoperative progressive explosive-type resistance training is feasible and effective in patients with hip osteoarthritis scheduled for total hip arthroplasty--a randomized controlled trial. *Osteoarthritis Cartilage.* 2016;24(1):91-98.
19. Hoogeboom TJ, Dronkers JJ, van den Ende CH, Oosting E, van Meeteren NL. Preoperative therapeutic exercise in frail elderly scheduled for total hip replacement: a randomized pilot trial. *Clin Rehabil.* 2010;24(10):901-910.
20. Huang SW, Chen PH, Chou YH. Effects of a preoperative simplified home rehabilitation education program on length of stay of total knee arthroplasty patients. *Orthop Traumatol Surg Res.* 2012;98(3):259-264.
21. Huber EO, de Bie RA, Roos EM, Bischoff-Ferrari HA. Effect of pre-operative neuromuscular training on functional outcome after total knee replacement: a randomized-controlled trial. *BMC Musculoskelet Disord.* 2013;14:157.
22. Jahic D, Omerovic D, Tanovic AT, Dzankovic F, Campara MT. The Effect of Prehabilitation on Postoperative Outcome in Patients Following Primary Total Knee Arthroplasty. *Med Arch.* 2018;72(6):439-443.
23. Kim SA-O, Hsu FC, Groban L, Williamson J, Messier S. A pilot study of aquatic prehabilitation in adults with knee osteoarthritis undergoing total knee arthroplasty - short term outcome. (1471-2474 (Electronic)).
24. van Leeuwen DM, de Ruiter CJ, Nolte PA, de Haan A. Preoperative strength training for elderly patients awaiting total knee arthroplasty. *Rehabil Res Pract.* 2014;2014:462750.
25. Lindback Y, Tropp H, Enthoven P, Abbott A, Oberg B. PREPARE: presurgery physiotherapy for patients with degenerative lumbar spine disorder: a randomized controlled trial. *Spine J.* 2018;18(8):1347-1355.
26. Lotzke H, Brisby H, Gutke A, et al. A Person-Centered Prehabilitation Program Based on Cognitive-Behavioral Physical Therapy for Patients Scheduled for Lumbar Fusion Surgery: A Randomized Controlled Trial. *Phys Ther.* 2019;99(8):1069-1088.
27. Marchand AA, Suitner M, O'Shaughnessy J, Chatillon CE, Cantin V, Descarreaux M. Feasibility of conducting an active exercise prehabilitation program in patients awaiting spinal stenosis surgery: a randomized pilot study. *Sci Rep.* 2019;9(1):12257.
28. Mat Eil Ismail MS, Sharifudin MA, Shokri AA, Ab Rahman S. Preoperative physiotherapy and short-term functional outcomes of primary total knee arthroplasty. *Singapore Med J.* 2016;57(3):138-143.
29. McKay C, Prapavessis H, Doherty T. The effect of a prehabilitation exercise program on quadriceps strength for patients undergoing total knee arthroplasty: a randomized controlled pilot study. *PM R.* 2012;4(9):647-656.

© 2023 Punnoose A et al. *JAMA Network Open*.

30. Mitchell C, Walker J, Walters S, Morgan AB, Binns T, Mathers N. Costs and effectiveness of pre- and post-operative home physiotherapy for total knee replacement: randomized controlled trial. *J Eval Clin Pract.* 2005;11(3):283-292.
31. Nct. Neuromuscular Electrical Stimulation (NMES) for Improving Outcomes Following Total Knee Arthroplasty (TKA). *Https://clinicaltrialsgov/show/nct03044028.* 2016.
32. Nct. Impact of Prehabilitation in Total Knee Replacement. *Https://clinicaltrialsgov/show/nct01844934.* 2013.
33. Nielsen PR, Jorgensen LD, Dahl B, Pedersen T, Tonnesen H. Prehabilitation and early rehabilitation after spinal surgery: randomized clinical trial. *Clin Rehabil.* 2010;24(2):137-148.
34. Oosting E, Jans MP, Dronkers JJ, et al. Preoperative home-based physical therapy versus usual care to improve functional health of frail older adults scheduled for elective total hip arthroplasty: a pilot randomized controlled trial. *Arch Phys Med Rehabil.* 2012;93(4):610-616.
35. Riddle DL, Keefe FJ, Ang DC, et al. Pain Coping Skills Training for Patients Who Catastrophize About Pain Prior to Knee Arthroplasty: A Multisite Randomized Clinical Trial. *J Bone Joint Surg Am.* 2019;101(3):218-227.
36. Risso AM, van der Linden ML, Bailey A, Gallacher P, Gleeson N. Exploratory insights into novel prehabilitative neuromuscular exercise-conditioning in total knee arthroplasty. *BMC Musculoskeletal Disorders.* 2022;23(1):547.
37. Rolving N, Nielsen Cv Fau - Christensen FB, Christensen Fb Fau - Holm R, Holm R Fau - Bünger CE, Bünger Ce Fau - Oestergaard LG, Oestergaard LG. Does a preoperative cognitive-behavioral intervention affect disability, pain behavior, pain, and return to work the first year after lumbar spinal fusion surgery? (1528-1159 (Electronic)).
38. Rooks DS, Huang J, Bierbaum BE, et al. Effect of preoperative exercise on measures of functional status in men and women undergoing total hip and knee arthroplasty. *Arthritis Rheum.* 2006;55(5):700-708.
39. Savkin R, Buker N, Gungor HR. The effects of preoperative neuromuscular electrical stimulation on the postoperative quadriceps muscle strength and functional status in patients with fast-track total knee arthroplasty. *Acta Orthop Belg.* 2021;87(4):735-744.
40. Shaarani SR, O'Hare C, Quinn A, Moyna N, Moran R, O'Byrne JM. Effect of prehabilitation on the outcome of anterior cruciate ligament reconstruction. *Am J Sports Med.* 2013;41(9):2117-2127.
41. Skoffer B, Maribo T, Mechlenburg I, Hansen PM, Soballe K, Dalgas U. Efficacy of Preoperative Progressive Resistance Training on Postoperative Outcomes in Patients Undergoing Total Knee Arthroplasty. *Arthritis Care Res (Hoboken).* 2016;68(9):1239-1251.
42. Soni A, Joshi A, Mudge N, Wyatt M, Williamson L. Supervised exercise plus acupuncture for moderate to severe knee osteoarthritis: a small randomised controlled trial. *Acupunct Med.* 2012;30(3):176-181.
43. Swank AM, Kachelman Jb Fau - Bibeau W, Bibeau W Fau - Quesada PM, et al. Prehabilitation before total knee arthroplasty increases strength and function in older adults with severe osteoarthritis. (1533-4287 (Electronic)).
44. Topp R, Swank AM, Quesada PM, Nyland J, Malkani A. The effect of prehabilitation exercise on strength and functioning after total knee arthroplasty. *PM R.* 2009;1(8):729-735.

© 2023 Punnoose A et al. *JAMA Network Open*.

45. Villadsen A, Overgaard S, Holsgaard-Larsen A, Christensen R, Roos EM. Postoperative effects of neuromuscular exercise prior to hip or knee arthroplasty: a randomised controlled trial. *Ann Rheum Dis.* 2014;73(6):1130-1137.
46. Walls RJ, Mchugh G, O'Gorman D, Moyna NM, O byrne JM. Effects of preoperative neuromuscular electrical stimulation on quadriceps strength and functional recovery in total knee arthroplasty. A pilot study. *BMC Musculoskeletal Disorders.* 2010;11:119 - 119.
47. Williamson L, Wyatt MR, Yein K, Melton JT. Severe knee osteoarthritis: a randomized controlled trial of acupuncture, physiotherapy (supervised exercise) and standard management for patients awaiting knee replacement. *Rheumatology (Oxford).* 2007;46(9):1445-1449.
48. Zeng R, Lin J, Wu S, et al. A randomized controlled trial: preoperative home-based combined Tai Chi and Strength Training (TCST) to improve balance and aerobic capacity in patients with total hip arthroplasty (THA). *Arch Gerontol Geriatr.* 2015;60(2):265-271.

© 2023 Punnoose A et al. *JAMA Network Open*.
