# Supplementary material for: Prehabilitation for Patients Undergoing Orthopedic Surgery: A Systematic Review and Meta-analysis
Source: JAMA Netw Open. 2023 Apr 13;6(4):e238050. doi: 10.1001/jamanetworkopen.2023.8050 (PMC10102876; doi:10.1001/jamanetworkopen.2023.8050)
Supplement: Supplement 2. — Data Sharing Statement [file jamanetwopen-e238050-s002.pdf]

Data Sharing Statement

Punnoose. Prehabilitation for Patients Undergoing Orthopedic Surgery. *JAMA Netw Open*. Published April 13, 2023. doi:10.1001/jamanetworkopen.2023.8050

Data
Data available: Yes
Data types: Data (not involving human participants)
How to access data: All data used in the study are available within the article or as supplementary evidence.
When available: With publication

Supporting Documents
Document types: None

Additional Information
Who can access the data: N/A
Types of analyses: For any purpose
Mechanisms of data availability: N/A
Any additional restrictions: none
